# Synthesis of a Thiazole Library via an Iridium-Catalyzed Sulfur Ylide Insertion Reaction

**DOI:** 10.1021/acs.orglett.2c02996

**Published:** 2022-10-20

**Authors:** Storm Hassell-Hart, Elisa Speranzini, Sirihathai Srikwanjai, Euan Hossack, S. Mark Roe, Daren Fearon, Daniel Akinbosede, Stephen Hare, John Spencer

**Affiliations:** † Department of Chemistry, School of Life Sciences, University of Sussex, Brighton BN1 9QJ, U.K.; ‡ Department of Biochemistry, School of Life Sciences, University of Sussex, Brighton BN1 9QG, U.K.; § Diamond LightSource (DLS), Harwell Science and Innovation Campus, Didcot OX11 0DE, U.K.

## Abstract

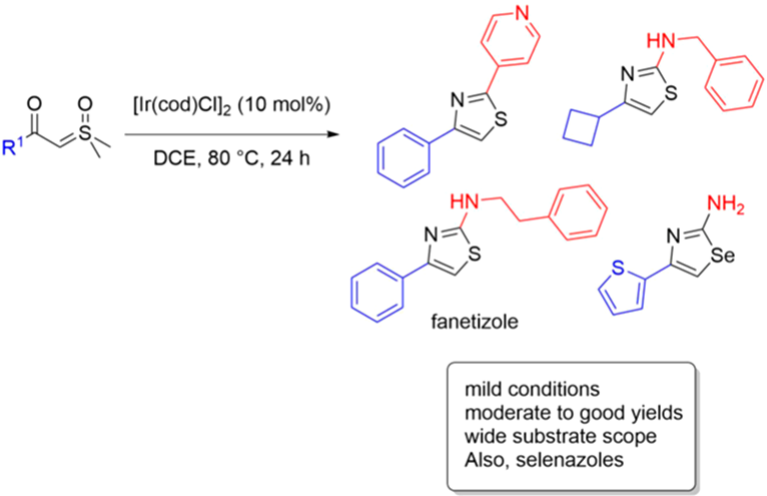

A library of thiazoles and selenothiazoles were synthesized via Ir-catalyzed ylide insertion chemistry. This process is a functional group, particularly heterocycle-substituent tolerant. This was applied to the synthesis of fanetizole, an anti-inflammatory drug, and a thiazole-containing drug fragment that binds to the peptidyl-tRNA hydrolase (Pth) in Neisseria gonorrheae bacteria.

Thiazoles are important motifs in natural and bioactive compounds.^1−5^ Unsurprisingly, a myriad of synthetic routes to these important heterocycles are documented, for example, Hantzsch’s seminal thiazole synthesis.^6,7^ However, the latter has limitations, which include the concomitant formation of 1 equiv of strong acid (HX) (Scheme 1). Moreover, it has a limited synthetic scope in terms of forming the α-halo or equivalent (X) ketone precursor, which can be unstable, be a potential alkylating agent, and not tolerate, e.g., heterocyclic, notably pyridyl, substituents. This has prompted the development of a range of methods to circumvent some of these shortcomings including, but not limited to, Cu-mediated reactions of oximes, anhydrides, and KSCN;^8^ oxidative amine and aldehyde couplings;^9^ Pd-mediated reactions to diversify thiazole cores via direct arylation;^10,11^ transformations using Lawesson’s reagent;^12^ and three-component reactions of enaminoesters, sulfur, and bromodifluoroacetamides.^13^ However, many of these often require complex or unstable starting materials or are limited in scope.

**Scheme 1:**
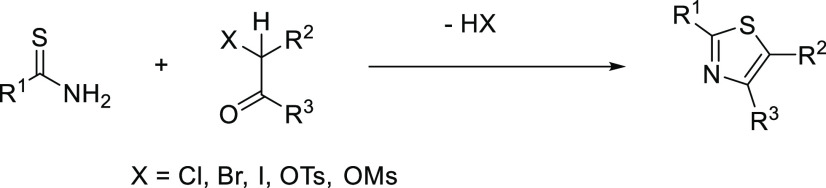
Hantzch Thiazole Synthesis

In 1993, Baldwin et al. demonstrated the use of rhodium-catalyzed carbenoid formation from sulfoxonium ylides, followed by intramolecular N–H insertion. Although this was somewhat limited, due to potential catalyst deactivation by the DMSO byproduct, it demonstrated that carbenoids could be synthesized from sulfoxonium ylides.^14^ More recent research has demonstrated the value of sulfoxonium ylides as diazo surrogates.^15^ In a key early example of this reactivity, sulfoxonium ylides were treated with HCl (or MsOH) to access α-halo carbonyls (or equivalents). A major advantage is that ylides are easy to prepare and are stable compared to their diazo congeners,^16^ which are generally found to be thermally unstable, presenting a potential explosion risk.^17^

Significant improvements to the insertion reaction were published by Mangion et al., who undertook a new catalyst screen and identified an iridium catalyst for the N–H insertion of sulfoxonium ylides.^18^ This occurs via loss of DMSO and the formation of an iridium carbene intermediate and was utilized by Merck in the synthesis of MK-7246, a CRTH2 antagonist,^19^ and MK-7655, a β-lactamase inhibitor.^20^ Sulfoxonium ylides have also recently been utilized in C–H activation, C–C bond formation, and asymmetric reactions, and this area is expected to grow as a scaleable, industrially viable alternative to the use of diazonium compounds.^21−24^

Here, we disclose a convenient, scalable, broad substrate tolerant route to a large thiazole library, mainly for biological evaluation. Central to this is an efficient synthesis of novel sulfoxinium ylides **1** as precursors to thiazoles with excellent substituent tolerance, operating under mild conditions.

After preliminary optimization studies (Tables S1 and S2) an iridium-catalyzed C–H insertion was applied to the synthesis of a library of thiazoles containing a R^1^ = Ph group (Scheme 2). Key observations include the reaction tolerance of free primary amine (**3c**), heterocycle (**3f**, **3l**), amide (**3h**), phenolic OH (**3k**), Boc (**3e**), and alkyl (**3o**, **3p**) groups. The yields were, generally, good to excellent. Of note, many products have high Csp^3^ character and are attractive as drug discovery scaffolds.^25^ Compound **3b**, fanetizole, an anti-inflammatory drug,^26^ was made in excellent yield. Its ^1^H NMR spectrum matched the one in the literature,^27^ verifying the regiochemistry of the reaction, which is known to be adversely affected under strong acidic conditions.^28^

**Scheme 2:**
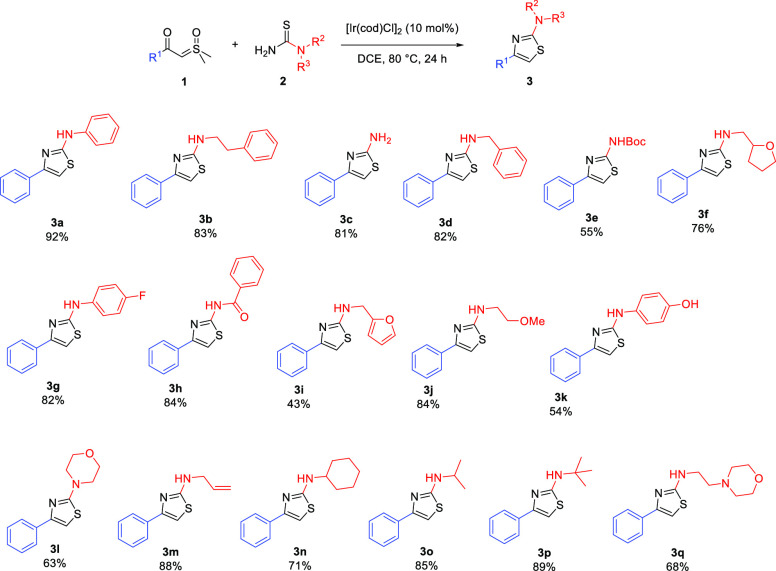
Thiazole Synthesis from Sulfonium Ylides

The scope of this reaction was significantly broadened by next changing the R^1^ group. Hence, this procedure tolerates a wide range of substituted aryl and heterocyclic groups (Scheme 3). Notably, a pyridyl substituent, incompatible with previous Hanztch chemistry (Scheme 1) yet readily synthesized as an ylide precursor, was tolerated, as well as alkyl and cycloalkyl groups, which tend to be harder to introduce at a later stage using standard chemistry.^29^

**Scheme 3:**
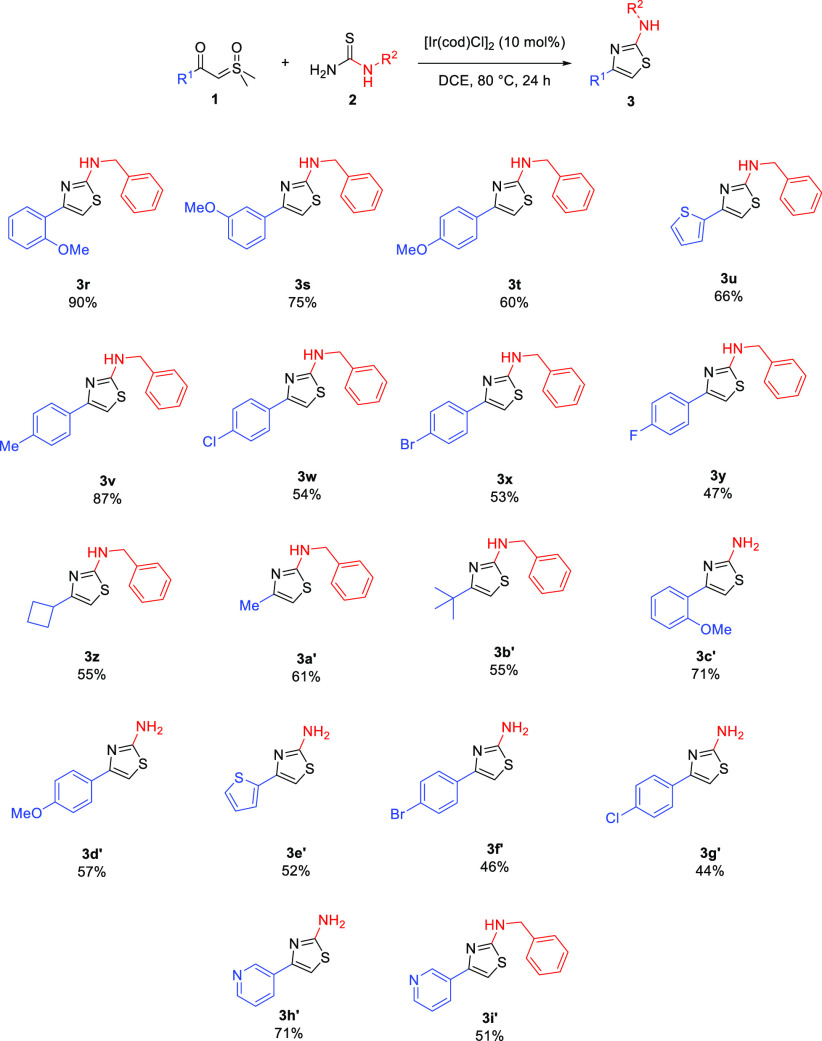
Further Cyclization Chemistry

Moreover, primary amines (**3c′**–**3h′**) and aryl groups, substituted with a range of electron-donating and electron-withdrawing groups, are tolerated. Those of the type “Ar–X” (e.g., **3w**, **3x**, **3f′**, and **3g′**) are especially attractive for further elaboration such as in Pd-catalyzed couplings.

Next, the related insertion reactions of thioamides **4** were attempted, enabling the synthesis of thiazoles devoid of a direct amine linker. These used the previously found conditions, as our goal was to make a broad selection of analogues **5** relatively quickly (Scheme 4). Despite this, yields tended to be moderate to good. Reactions are tolerant of alkyl (**5a**), aryl (**5b**–**5e** and **5k**), and heterocyclic substituents (**5g**–**5j**). Protected amines, such as **5f**, will be useful “handles”, once deprotected, for further library elaboration.

**Scheme 4:**
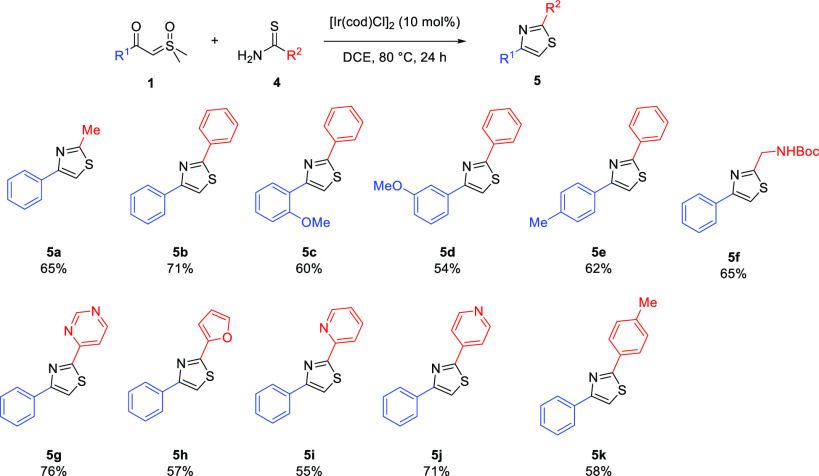
Thioamide Insertions

Buoyed by the successful implementation of these protocols, we shifted our attention to the corresponding selenazoles, **7**,^30^ which were made in moderate yields, starting from selenourea **6** (Scheme 5). All analogues **7** should be useful building blocks for further elaboration, such as amide, sulfonamide, reductive amination, and heterocyclization chemistry.^31,32^

**Scheme 5:**
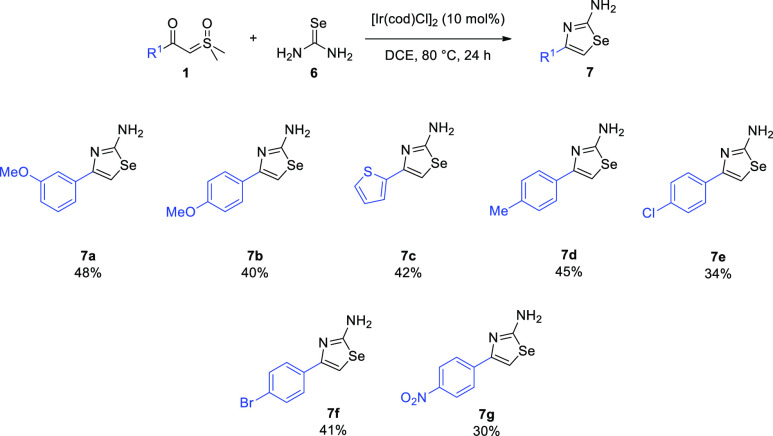
Selenazole Synthesis

Finally, we have applied this chemistry to the synthesis of a small library of analogues related to and including **3r′**. The latter was found as a crystallographic hit (PDB: 8AXP) from a structural screen of a fragment library vs the peptidyl-tRNA hydrolase (Pth) in Neisseria gonorrheae bacteria (Scheme 6).^33−35^

**Scheme 6:**
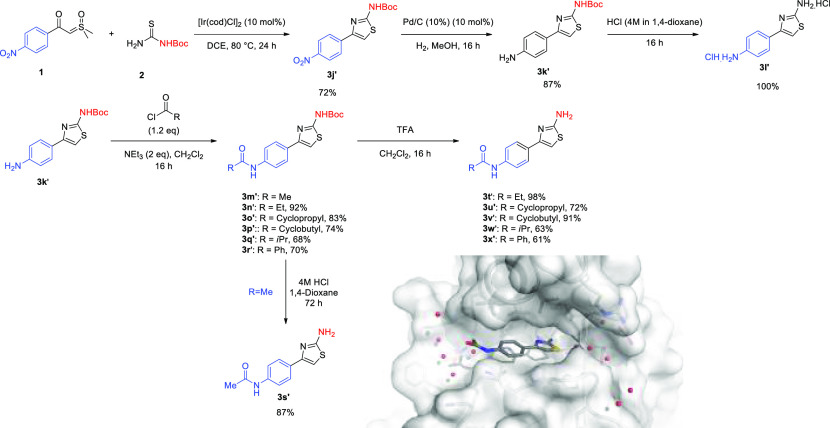
PTH Hit Based Library

In conclusion, Ir-catalyzed insertions of sulfoxonium ylides are very versatile reactions in the synthesis of a range of S, N, and Se heterocycles. This is a useful, substrate-tolerant approach to thiazoles and selenazoles and should have high value in library diversification in medicinal chemistry.

## Notes

The data underlying this study are openly available at https://pubs.acs.org/doi/10.1021/acs.orglett.2c02996.

## Supporting Information Available

Optimization procedures, synthetic and analytical procedures for ylides and final heterocyclic products, and scanned spectra (^1^H, ^13^C NMR, and HPLC-MS) (PDF)

## Dedication

In memory of Dr. Stephen Hare. A fabulous colleague, scientist, and teacher.
