# Supplementary material for: Synthesis of a Thiazole Library via an Iridium-Catalyzed Sulfur Ylide Insertion Reaction
Source: Org Lett. 2022 Oct 20;24(43):7924–7. doi: 10.1021/acs.orglett.2c02996 (PMC9641659; doi:10.1021/acs.orglett.2c02996)
Supplement: Supplementary file 1 — ol2c02996_si_001.pdf [file ol2c02996_si_001.pdf]

Supporting Information

Synthesis of a Thiazole Library via an Iridium Catalyzed Sulfur Ylide Insertion Reaction.

Storm Hassell-Hart,* Elisa Speranzini, Sirihathai Srikwanjai, Euan Hossack, S. Mark Roe, Daren Fearon, Daniel Akinbosede, Stephen Hare[ʃ] and John Spencer*

AUTHOR INFORMATION

Corresponding authors

Storm Hassell-Hart – Department of Chemistry, School of Life Sciences, University of Sussex, BN1 9QJ, U.K.

John Spencer – Department of Chemistry, School of Life Sciences, University of Sussex, BN1 9QJ, U.K. ORCID 0000-0001-5231-8836.

Authors

Elisa Speranzini - Department of Chemistry, School of Life Sciences, University of Sussex, BN1 9QJ, U.K.

Sirihathai Srikwanjai - Department of Chemistry, School of Life Sciences, University of Sussex, BN1 9QJ, U.K.

Euan Hossack - Department of Biochemistry, School of Life Sciences, University of Sussex, BN1 9QG, U.K.

S. Mark Roe - Department of Biochemistry, School of Life Sciences, University of Sussex, BN1 9QG, U.K.

Daren Fearon – Diamond LightSource (DLS), Harwell Science and Innovation Campus, Didcot, OX11 0DE, U.K.

Daniel Akinbosede - Department of Biochemistry, School of Life Sciences, University of Sussex, BN1 9QG, U.K.

Stephen Hare[ʃ] - Department of Biochemistry, School of Life Sciences, University of Sussex, BN1 9QG, U.K.

[ʃ]deceased December 2021.

Contents

General Experimental 2

Preparation of Sulfoxonium Ylides 3

Reaction Optimisation of the Iridium-Mediated Cyclisation.. 10

Substrate Scope of the Iridium-Mediated Cyclisation 12

Insertion/Cyclisation of Thioamides.. 12

Insertion/Cyclisation of Thioureas.. 18

Insertion/Cyclisation of Selenoureas 34

Synthesis of Prospective peptidyl-tRNA hydrolase (Pth) Inhibitors 37

Crystallographic Table 184

General Experimental

Solvents, reagents, and consumables, including intermediates **2**, **4** and **6** (Sigma Aldrich), such as TLC plates and column material, were purchased from commercial suppliers and solvents/reagents were subsequently used without purification. ^{1}H, ^{13}C NMR spectroscopy was performed on Varian 500 MHz or 600 MHz spectrometers and chemical shifts are reported in ppm, usually referenced to TMS as an internal standard. LCMS measurements were performed on a Shimadzu LCMS-2020 equipped with a Gemini® 5 µm C18 110 Å column and percentage purity measurements were run over 30 minutes in water/acetonitrile with 0.1% formic acid (5 min at 5%, 5–95% over 20 min, 5 min at 95%) with the UV detector set at 254 nm. High-Resolution Accurate Mass Spectrometry measurements were taken using a Waters Xevo G2 Q-ToF HRMS (Wilmslow, Cheshire, UK), equipped with an ESI source and MassLynx software. Experimental parameters were: (1)—ESI source: capillary voltage 3.0 kV, sampling cone 35 au, extraction cone 4 au, source temperature 120 °C and desolvation gas 450 °C with a desolvation gas flow of 650 L/h and no cone gas; (2)—MS conditions: MS in resolution mode between 100 and 1500 Da. Additionally, a Waters (Wilmslow, Cheshire, UK) Acquity H-Class UHPLC chromatography pumping system with column oven was used, connected to a Waters Synapt G2 HDMS high-resolution mass spectrometer.

Preparation of Sulfoxonium Ylides

Protocol for drying trimethylsulfoxonium chloride

Trimethylsulfoxonium chloride was dried under vacuum at 50 °C for 72 h prior to use. The resulting salts were cooled to ambient temperature and stored in a covered vessel under argon until required. *Note – analysis of the mass before and after suggested that the untreated salts could contain up to 5% water by mass.*

General Protocol for the Preparation of Sulfoxonium Ylides

To a mixture of dried trimethylsulfoxonium chloride (3 eq) and anhydrous THF (0.75 M) was added potassium *tert*-butoxide (4 eq). The resulting mixture was heated to reflux (using a dry block heating vessel) and stirred for 3 h under an argon atmosphere. The reaction was cooled to ambient temperature and, to the mixture, was added the acid chloride substrate (1 eq). The resulting mixture was stirred for 16 h under argon and then concentrated under reduced pressure. To the mixture were added water (50 mL) and a solution of DCM:IPA (dichloromethane, isopropanol) (3:1) (50 mL). The resulting mixture was separated and the aqueous phase extracted with DCM:IPA (3:1) (5 x 50 mL). The combined organic extracts were dried over anhydrous $MgSO_4$, filtered, and concentrated under reduced pressure. Purification by dissolving in the minimum amount of boiling ethyl acetate, followed by precipitation with *n*-hexane. The mixture was filtered, washed with a mixture of ethyl acetate:*n*-hexane (1:1) and dried under reduced pressure to give the title compound.

Representative Example of Ylide Synthesis: Benzoyl(dimethyloxosulphonio)methanide (1a)

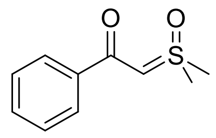

To a mixture of dried trimethylsulfoxonium chloride (5.67 g, 44.09 mmol) and anhydrous THF (60 mL) was added potassium *tert*-butoxide (6.60 g, 58.79 mmol). The resulting mixture was heated to reflux and stirred for 3 h under an argon atmosphere. The reaction was cooled to 0 °C and to the mixture was added dropwise benzoyl chloride (1.71 mL, 14.70 mmol). The reaction was warmed to ambient

temperature and stirred for 16 h under argon. The resulting mixture was concentrated under reduced pressure. To the resulting residue was added water (50 mL) and a solution of DCM:IPA (3:1) (50 mL). The resulting mixture was separated and the aqueous phase extracted with DCM:IPA (3:1) (5 x 50 mL). The combined organic extracts were dried over anhydrous $MgSO_4$, filtered, and concentrated under reduced pressure to give an off-white solid (4.2 g). The solid was dissolved in the minimum amount of boiling ethyl acetate and the product precipitated by the addition of *n*-hexane. The mixture was filtered, washing with a mixture of ethyl acetate:*n*-hexane (1:1) and dried under reduced pressure to give benzoyl(dimethyloxosulphonio)methanide (**1a**) as a white solid (2.61 g, 91%). LCMS (UV, ESI) R_t = 7.19 min, $[M-H]^+$ *m/z* = 196.8, 99% purity. ^{1}H NMR (600 MHz, d_6-DMSO): δ = 7.78-7.73 (2H, m), 7.46-7.36 (3H, m), 5.59 (1H, s), 3.55 (6H, s). ^{13}C NMR (151 MHz, d_6-DMSO): δ = 180.3, 139.4, 130.3, 128.0, 126.3, 72.2, 40.5. HRMS (ESI-[+H]) *m/z*: Calcd for $C_{10}H_{13}O_2S$ 197.0636; Found 197.0646. Data in agreement with previously reported data.[1]

4-Methoxybenzoyl(dimethyloxosulphonio)methanide (1b)

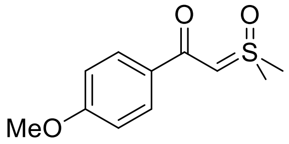

Preformed according to the general protocol with 4-methoxybenzoyl chloride (853 mg, 5.00 mmol) to give 4-methoxybenzoyl(dimethyloxosulphonio)methanide (**1b**) as a pale-yellow solid (644 mg, 57%). LCMS (UV, ESI) R_t = 7.16 min, $[M-H]^+$ *m/z* = 226.8, 91% purity. ^{1}H NMR (600 MHz, d_6-DMSO): δ = 7.72 (2H, d, J = 8.8 Hz), 6.92 (2H, d, J = 8.8 Hz), 5.52 (1H, s), 3.78 (3H, s), 3.53 (6H, s). ^{13}C NMR (151 MHz, d_6-DMSO): δ = 179.9, 160.9, 132.0, 128.1, 113.2, 71.2, 55.2, 40.7. HRMS (ESI-[+H]) *m/z*: Calcd for $C_{11}H_{15}O_3S$ 227.0742; Found 227.0728. Data in agreement with previously reported data.[1]

4-Methylbenzoyl(dimethyloxosulphonio)methanide (1c)

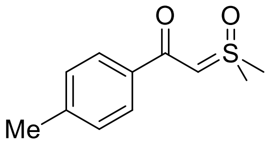

Preformed according to the general protocol with *p*-toluoyl chloride (666 µL, 5.00 mmol) to give 4-methylbenzoyl (dimethyloxosulphonio)methanide (**1c**) as an off-white solid (976 mg, 93%). LCMS (UV, ESI) R_t = 7.20 min, $[M-H]^+$ *m/z* = 210.8, 95% purity. ^{1}H NMR (600 MHz, d_6-DMSO): δ = 7.65 (2H, d, J = 8.3 Hz), 7.19 (2H, d, J = 7.9 Hz), 5.56 (1H, s), 3.54 (6H, s), 2.32 (3H, s). ^{13}C NMR (151

MHz, d_6-DMSO): δ = 180.3, 139.9, 136.8, 128.6, 126.4, 71.7, 42.1, 21.0. HRMS (ESI-[+H]) *m/z*: Calcd for $C_{11}H_{15}O_2S$ 211.0793; Found 211.0792. Data in agreement with previously reported data.[2]

4-Chlorobenzoyl(dimethyloxosulphonio)methanide (1d)

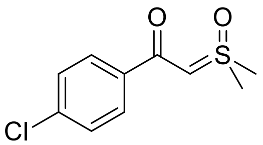

Preformed according to the general protocol with 4-chlorobenzoyl chloride (641 µL, 5.00 mmol) to give 4-chlorobenzoyl(dimethyloxosulphonio)methanide (**1d**) as an off-white solid (873 mg, 73%). LCMS (UV, ESI) R_t = 9.99 min, $[M\text{-}H]^+$ *m/z* = 230.8, 97% purity. ^{1}H NMR (600 MHz, d_6-DMSO): δ = 7.77 (2H, d, J = 8.5 Hz), 7.46 (2H, d, J = 8.5 Hz), 5.65 (1H, s), 3.55 (6H, s). ^{13}C NMR (151 MHz, d_6 -DMSO): δ = 178.7, 138.2, 134.9, 128.2, 128.1, 72.6, 40.5. HRMS (ESI-[+H]) *m/z*: Calcd for $C_{10}H_{12}O_2SCl$ 231.0247; Found 231.0234. Data in agreement with previously reported data.[2]

4-Bromobenzoyl(dimethyloxosulphonio)methanide (1e)

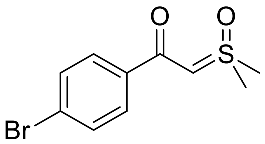

Preformed according to the general protocol with 4-bromobenzoyl chloride (1.10 g, 5.00 mmol) to give 4-bromobenzoyl(dimethyloxosulphonio)methanide (**1e**) as an off-white solid (1.00 g, 73%). LCMS (UV, ESI) R_t = 10.66 min, $[M\text{-}H]^+$ *m/z* = 274.8, 95% purity. ^{1}H NMR (600 MHz, d_6-DMSO): δ = 7.72-7.69 (2H, m), 7.61-7.58 (2H, m), 5.65 (1H, s), 3.55 (6H, s). NMR (151 MHz, d_6 -DMSO): δ = 178.8, 138.5, 131.0, 128.5, 123.9, 72.7, 42.1. HRMS (ESI-[+H]) *m/z*: Calcd for $C_{10}H_{12}O_2SBr$ 274.9741; Found 274.9733. Data in agreement with previously reported data.[1]

4-Nitrobenzoyl(dimethyloxosulphonio)methanide (1f)

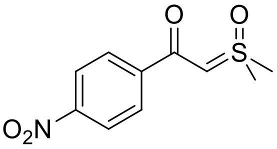

Preformed according to the general protocol with 4-nitrobenzoyl chloride (2.74 g, 14.70 mmol) to give 4-nitrobenzoyl(dimethyloxosulphonio)methanide (**1f**) as a yellow solid (1.96 g, 55%). LCMS (UV, ESI) R_t = 10.28 min, $[M\text{-}H]^+$ *m/z* = 242.0, 97% purity. ^{1}H NMR (600 MHz, d_6-DMSO): δ = 8.28-8.23 (2H, m), 8.02-7.97 (2H, m), 5.80 (1H, s), 3.59 (6H, s). NMR (151 MHz, d_6-DMSO): δ = 177.9, 148.8, 145.5, 128.0, 123.8, 74.9, 40.7. HRMS (ESI-[+H]) *m/z*: Calcd for $C_{10}H_{12}NO_4S$ 242.0487; Found 242.0481. Data in agreement with previously reported data.[2]

2-Methoxybenzoyl(dimethyloxosulphonio)methanide (1g)

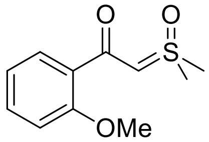

Preformed according to the general protocol with 2-methoxybenzoyl chloride (853 mg, 5.00 mmol) to give 2-methoxybenzoyl (dimethyloxosulphonio)methanide (**1g**) as a pale-yellow solid (842 mg, 74%). LCMS (UV, ESI) R_t = 7.29 min, $[M\text{-}H]^+$ *m/z* = 226.8, 99% purity. ^{1}H NMR (600 MHz, d_6-DMSO): δ = 7.68 (1H, dd, J = 7.6, 1.9 Hz), 7.35 (1H, ddd, J = 8.3, 7.3, 1.9 Hz), 7.03 (1H, dd, J = 8.3, 1.0 Hz), 6.97-6.93 (1H, m), 5.45 (1H, s), 3.81 (3H, s), 3.52 (6H, s). ^{13}C NMR (151 MHz, d_6-DMSO): δ = 178.9, 157.1, 131.0, 129.3, 129.0, 120.0, 111.8, 76.2, 55.5, 40.6. HRMS (ESI-[+H]) *m/z*: Calcd for $C_{11}H_{15}O_3S$ 227.0742; Found 227.0728.

3-Methoxybenzoyl(dimethyloxosulphonio)methanide (1h)

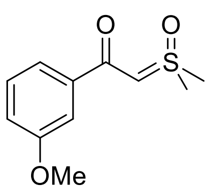

Preformed according to the general protocol with 3-methoxybenzoyl chloride (853 mg, 5.00 mmol) to give 3-methoxybenzoyl (dimethyloxosulphonio)methanide (**1h**) as an off-white solid (796 mg, 70%). LCMS (UV, ESI) R_t = 7.42 min, $[M\text{-}H]^+$ *m/z* = 226.8, 99% purity. ^{1}H NMR (600 MHz, d_6-DMSO): δ = 7.34-7.28 (3H, m), 6.99 (1H, ddd, J = 7.8, 2.6, 1.3 Hz), 5.60 (1H, s), 3.78 (3H, s), 3.55 (6H, s). ^{13}C NMR (151 MHz, d_6-DMSO): δ = 179.9, 159.1, 141.1, 129.1, 118.7, 116.1, 111.3, 72.4, 55.1, 40.5. HRMS (ESI-[+H]) *m/z*: Calcd for $C_{11}H_{15}O_3S$ 227.0742; Found 227.0728.

2-Thiophenecarbonyl(dimethyloxosulphonio)methanide (1i)

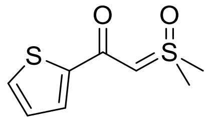

Preformed according to the general protocol with 2-thiophenecarbonyl chloride (535 µL, 5.00 mmol) to to give 2-thiophenecarbonyl (dimethyloxosulphonio)methanide (**1i**) as an off-white solid (694 mg, 69%). LCMS (UV, ESI) R_t = 7.01 min, $[M\text{-}H]^+$ *m/z* = 202.8, 95% purity. ^{1}H NMR (600 MHz, d_6-DMSO): δ = 7.62 (1H, dd, J = 4.9, 1.1 Hz), 7.46 (1H, dd, J = 3.7, 1.2 Hz), 7.07 (1H, dd, J = 4.9, 3.6 Hz), 5.51 (1H, s), 3.55 (6H, s). ^{13}C NMR (151 MHz, d_6-DMSO): δ = 174.2, 147.1, 129.2, 127.7, 126.6, 71.3, 40.7. HRMS (ESI-[+H]) *m/z*: Calcd for $C_8H_{11}O_2S_2Na$ 225.0020; Found 225.0014. Data in agreement with previously reported data.[1]

2-(Dimethyl(oxo)-λ6-sulfaneylidene)-1-(4-fluorophenyl)ethan-1-one (1j)

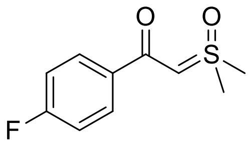

Preformed according to the general protocol with 4-fluorobenzoyl chloride (0.90 mL, 6.70 mmol) to give 2-(dimethyl(oxo)-λ^6-sulfaneylidene)-1-(4-fluorophenyl)ethan-1-one (**1j**) as a white solid (1.29 g, 90%). LCMS (UV, ESI) R_t = 7.98 min, $[M\text{-}H]^+$ *m/z* = 214.8, 96% purity. ^{1}H NMR (600 MHz, d_6-DMSO): δ = 7.85-7.79 (2H, m), 7.24-7.17 (2H, m), 5.62 (1H, s), 3.55 (6H, s). ^{13}C NMR (151 MHz, d_6 -DMSO): δ = 178.9, 163.4 (d, $^1J_{C\text{-}F}$ = 246.9 Hz), 135.9 (d, $^4J_{C\text{-}F}$ = 2.8 Hz), 128.8 (d, $^3J_{C\text{-}F}$ = 8.7 Hz), 114.8 (d, $^2J_{C\text{-}F}$ = 21.4 Hz), 72.2, 40.5. ^{19}F NMR (376 MHz, d_6-DMSO) δ = -111.26. HRMS (ESI-[+H]) *m/z*: Calcd for $C_{10}H_{12}O_2SF$ 215.0542; Found 215.0536. Data in agreement with previously reported data.[2]

1-(Dimethyl(oxo)-λ^6-sulfanylidene)-3,3-dimethylbutan-2-one (1k)

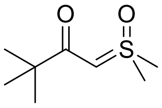

Preformed according to the general protocol with pivaloyl chloride (838 µL, 6.80 mmol) to give 1-(dimethyl(oxo)-λ^6-sulfanylidene)-3,3-dimethylbutan-2-one (**1k**) as a white solid (1.06 g, 89%). ^{1}H NMR (600 MHz, d_6-DMSO): δ = 4.83 (1H, s), 3.40 (6H, s), 1.00 (9H, s). ^{13}C NMR (151 MHz, d_6 -DMSO): δ = 195.3, 69.4, 42.1, 40.5, 27.9. HRMS (ESI-[+H]) *m/z*: Calcd for $C_8H_{17}O_2S$ 177.0949; Found 177.0958. Data in agreement with previously reported data.[3]

1-Cyclobutyl-2-(dimethyl(oxo)-λ^6-sulfanylidene)ethan-1-one (1l)

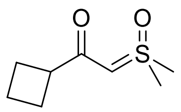

Preformed according to the general protocol with cyclobutanecarbonyl chloride (776 µL, 6.80 mmol) to give 1-cyclobutyl-2-(dimethyl(oxo)-λ^6-sulfanylidene)ethan-1-one (**1l**) as a white solid (680 mg, 57%). ^{1}H NMR (600 MHz, d_6-DMSO): δ = 4.68 (1H, s), 3.42 (6H, s), 2.92-2.84 (1H, m), 2.10-2.00 (2H, m), 1.98-1.87 (2H, m), 1.86-1.77 (1H, m), 1.71-1.64 (1H, m). ^{13}C NMR (151 MHz, d_6-DMSO): δ = 70.8, 43.2, 40.4, 25.0, 17.5. HRMS (ESI-[+H]) *m/z*: Calcd for $C_8H_{15}O_2S$ 175.0793; Found 175.0805. Data in agreement with previously reported data.[4]

2-(Dimethyl(oxo)-λ^6-sulfanylidene)-*N*-phenylacetamide (1m)

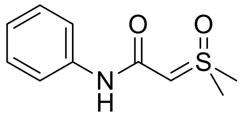

Preformed according to the general protocol but with phenyl isocyanate (739 µL, 6.80 mmol) to give 2-(dimethyl(oxo)-λ^6-sulfanylidene)-*N*-phenylacetamide (**1m**) as a white solid (1.02 g, 71%). LCMS (UV, ESI) R_t = 7.06 min, $[M-H]^+$ *m/z* = 212.0, 99% purity. ^{1}H NMR (600 MHz, d_6-DMSO): δ = 8.73 (1H, s), 7.49-7.44 (2H, m), 7.19-7.13 (2H, m), 6.85-6.79 (1H, m), 4.14 (1H, s), 3.44 (6H, s). ^{13}C NMR (151 MHz, d_6-DMSO): δ = 165.8, 141.4, 128.4, 120.5, 117.9, 59.1, 41.2. HRMS (ESI-[+H]) *m/z*: Calcd for $C_{10}H_{14}NO_2S$ 212.0764; Found 212.0723. Data in agreement with previously reported data.[5]

1-(Dimethyl-(oxo)-λ^6-sulfanylidene)propan-2-one (1n)

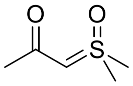

Preformed according to the general protocol with acetyl chloride (485 µL, 6.80 mmol) to give 1-(dimethyl-(oxo)-λ^6-sulfanylidene)propan-2-on (**1n**) as a yellow oil (456 mg, 50%). ^{1}H NMR (600 MHz, d_6-DMSO): δ = 4.73 (1H, s), 3.41 (6H, s), 1.73 (3H, s). ^{13}C NMR (151 MHz, d_6-DMSO): δ = 173.4, 38.6, 26.0. Data in agreement with previously reported data.[1]

2-(Dimethyl(oxo)-λ^6-sulfaneylidene)-1-(pyridin-3-yl)ethan-1-oneone (1o)

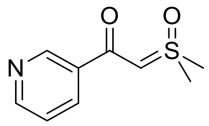

To a mixture of potassium *tert*-butoxide (3.03 g, 27.00 mmol) and anhydrous THF (55 mL) was added dried trimethylsulfoxonium chloride (3.47 g, 27.00 mmol) and the resulting mixture refluxed for 3 h under argon. The resulting mixture was cooled to 0 °C and to the mixture was added dropwise a solution of nicotinoyl chloride hydrochloride (1.60 mg, 9.00 mmol), NEt_3 (1.26 mL, 9.00 mmol), and anhydrous THF (10 mL) which had been pre-stirred for 1 h at ambient temperature. The resulting mixture was warmed to ambient temperature and stirred for 72 h under argon. The resulting mixture was filtered through Celite® and concentrated under reduced pressure to give an orange oil (3.30 g). The resulting residue was purified by automated flash column chromatography (EtOAc/MeOH, 100:0 – 60:40, 40 g SiO_2). The appropriate fractions were combined and concentrated to give 2-(dimethyl(oxo)-λ^6-sulfaneylidene)-1-(pyridin-3-yl)ethan-1-oneone (**1o**) as a off-white solid (460 mg) of sufficient purity to be used in the subsequent reactions without purification.

Reaction Optimisation of the Iridium-Mediated Cyclisation

Solvent Screen for the Iridium-mediated cyclisation

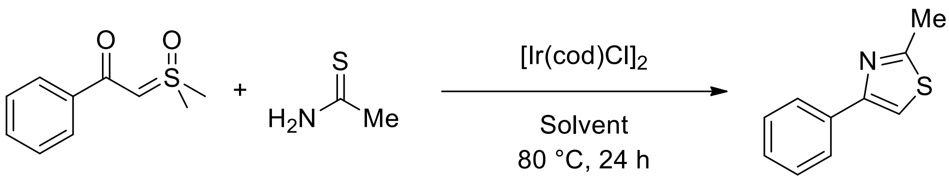

Scheme S1. Solvent Screen for the Iridium Cyclisation.

General protocol:

A mixture of thioacetamide (23 mg, 0.306 mmol), benzoyl(dimethyloxosulphonio)methanide (40 mg, 0.204 mmol), and solvent (1 mL) was heated to 80 °C. To the mixture was added $[Ir(cod)Cl]_2$ (13.7 mg, 0.0204 mmol) and the reaction stirred at 80 °C for 24 h. The reaction was cooled to ambient temperature, concentrated under reduced pressure, and to the mixture was added a known amount of durene as an external standard. The % yield/conversion was calculated by the integration of the durene and product signals in the proton NMR.

Solvent	^{1}H NMR Yield (%)[a]
**Toluene**	13
**THF**	3
**DMA**	32
**DCM**[b]	26
**DCE**	47
**1,4-Dioxane**	6
**IPA**	9
**HFIP**	9
**MeCN**	44

[a] durene as an external standard. **[b]** Conducted in a sealed tube at 80 °C.

Table S1. Results of the Solvent Screen for the Iridium Cyclisation.

Equivalents, concentration, and catalyst loading for the Iridium-mediated cyclisation

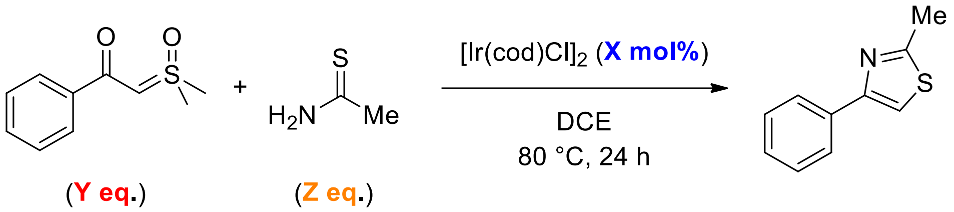

Scheme S2. Optimisation of the Iridium Cyclisation.

General protocol:

A mixture of thioacetamide (**Y eq.**), benzoyl(dimethyloxosulphonio)methanide (**Z eq.**), and solvent (**X** mL) was heated to 80 °C. To the mixture was added $[Ir(cod)Cl]_2$ (**X mol%**) and the reaction stirred at 80 °C for 24 h. The reaction was cooled to ambient temperature, concentrated under reduced pressure, and to the mixture was added a known amount of durene as an external standard. The % yield/conversion was calculated by the integration of the durene and product signals in the proton NMR.

Entry	Thioacetamide (eq)	Ylide (eq)	Catalyst loading (mol%)	Solvent volume (mL)	Concentration of Thioacetamide (M)	NMR yield (%)
1	2.0	1.0	10	1	0.4	21
2	1.0	3.0	10	1	0.2	0[a]
3	1.0	3.0	10	1	0.2	61
4	1.0	1.5	5	1	0.2	11[a]
5	1.0	1.5	5	1	0.2	0[b]
6	1.0	1.5	5	1	0.2	66
7	1.0	1.5	1	1	0.2	57
8	1.0	1.5	0	1	0.2	0
9	1.0	1.5	5	0.5	0.4	58
10	1.0	1.5	1	0.5	0.4	35
11	1.0	1.5	10	0.5	0.4	79

[a]All reactants were added at ambient temperature (including the catalyst) before heating to 80 °C.

[b]Preincubation of the catalyst, solvent, and thioacetamide at 80 °C for 15 mins, prior to addition of the ylide.

Table S2. Results of the Optimisation Screen for the Iridium Cyclisation.

Substrate Scope of the Iridium-Mediated Cyclisation

General Procedure for the Synthesis of 1,3-Thiazoles/Selenazoles

A mixture of respective β-ketosulfoxonium ylide (1.0 eq, 0.408 mmol), urea/thioamide/selenourea (1.5 eq, 0.612 mmol) and DCE (1 mL) was heated to 80 °C and to the mixture was added [Ir(cod)Cl]$_2$ (27.4 mg, 10 mol%, 0.041 mmol). The resulting mixture was stirred for 24 h at 80 °C. The reaction was cooled to ambient temperature and concentrated under reduced pressure. The resulting residue was purified by automated column chromatography to give the title compound.

Insertion/Cyclisation of Thioamides

Representative Example of the Insertion/Cyclisation: 2-Methyl-4-phenylthiazole (5a)

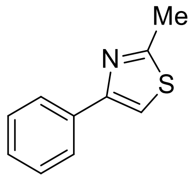

A mixture of benzoyl(dimethyloxosulphonio)methanide (80 mg, 0.408 mmol), thioacetamide (46 mg, 0.612 mmol), and DCE (1 mL) was heated to 80 °C and to the mixture was added [IrCODCl]$_2$ (27.4 mg, 0.041 mmol). The resulting mixture was stirred for 24 h at 80 °C. The reaction was cooled to ambient temperature and concentrated under reduced pressure. The resulting residue was purified by automated flash column chromatography (hexane/EtOAc, 100:0 – 90:10, 24 g SiO_2) to give 2-methyl-4-phenylthiazole (**5a**) as a cloudy gum (42 mg, 65%). LCMS (UV, ESI) R_t = 18.38 min, $[M-H]^+$ *m/z* = 175.8, 94% purity. ^{1}H NMR (600 MHz, d_6-DMSO): δ = 7.95-7.92 (3H, m), 7.44-7.40 (2H, m), 7.34-7.31 (1H, m), 2.72 (3H, s). ^{13}C NMR (151 MHz, d_6 -DMSO): δ = 165.5, 153.8, 134.2, 128.8, 127.9, 126.0, 113.8, 19.0. HRMS (ESI-[+H]) *m/z*: Calcd for $C_{10}H_{10}NS$ 176.0534; Found 176.0518.

2,4-Diphenylthiazole (5b)

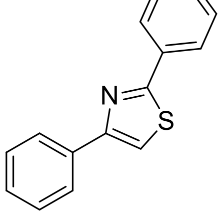

Preformed according to the general protocol with benzoyl(dimethyloxosulphonio)methanide (80 mg, 0.408 mmol) and thiobenzamide (84 mg, 0.612 mmol). The product was purified by automated flash column chromatography (hexane/EtOAc, 100:0 – 85:15, 24 g SiO_2) to give 2,4-diphenylthiazole (**5b**) as a white solid (64 mg, 71%). LCMS (UV, ESI) R_t = 25.89 min, $[M-H]^+$ *m/z* = 237.8, 93% purity. ^{1}H NMR (600 MHz, d_6-DMSO): δ = 8.20 (1H, s), 8.08-8.02 (5H, m), 7.57-7.46 (4H, m), 7.40-7.36 (1H, m). ^{13}C NMR (151 MHz, d_6-DMSO): δ = 167.0, 155.2, 134.0, 133.0, 130.5, 129.4, 128.9, 128.3, 126.3, 126.2, 114.7. HRMS (ESI-[+H]) *m/z*: Calcd for $C_{15}H_{12}NS$ 238.0690; Found 238.0702.

4-(2-Methoxyphenyl)-2-phenylthiazole (5c)

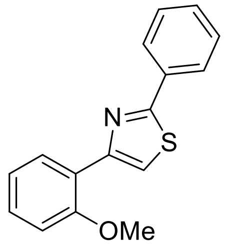

Preformed according to the general protocol with 2-methoxybenzoyl (dimethyloxosulphonio)methanide (92 mg, 0.408 mmol) and thiobenzamide (84 mg, 0.612 mmol). The product was purified by automated flash column chromatography (hexane/EtOAc, 100:0 – 95:5, 12 g SiO_2) to give 4-(2-methoxyphenyl)-2-phenylthiazole (**5c**) as an off-white solid (65 mg, 60%). LCMS (UV, ESI) R_t = 26.38 min, $[M-H]^+$ *m/z* = 268.0, 97% purity. ^{1}H NMR (600 MHz, d_6-DMSO): δ = 8.31 (1H, dd, J = 7.7, 1.8 Hz), 8.15 (1H, s), 8.06 – 8.01 (2H, m), 7.57 – 7.48 (3H, m), 7.37 (1H, ddd, J = 8.3, 7.3, 1.8 Hz), 7.17 (1H, dd, J = 8.3, 1.1 Hz), 7.11-7.07 (1H, m), 3.95 (3H, s). ^{13}C NMR (151 MHz, d_6-DMSO): δ = 165.1, 156.6, 151.1, 133.1, 130.3, 129.5, 129.4, 129.3, 126.3, 122.3, 120.6, 118.1, 111.8, 55.6. HRMS (ESI-[+H]) *m/z*: Calcd for $C_{16}H_{14}NOS$ 268.0796; Found 268.0785.

4-(3-Methoxyphenyl)-2-phenylthiazole (5d)

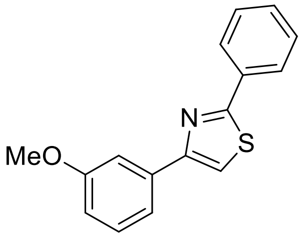

Preformed according to the general protocol with 3-methoxybenzoyl (dimethyloxosulphonio)methanide (92 mg, 0.408 mmol) and thiobenzamide (84 mg, 0.612 mmol). The product was purified by automated flash column chromatography (hexane/EtOAc, 100:0 – 95:5, 12 g SiO_2) to give 4-(3-methoxyphenyl)-2-phenylthiazole (**5d**) as an off-white solid (59 mg, 54%). LCMS

(UV, ESI) R_t = 25.40 min, $[M-H]^+$ *m/z* = 268.0, 99% purity. ^{1}H NMR (600 MHz, d_6-DMSO): δ = 8.23 (1H, s), 8.06 – 8.01 (2H, m), 7.65-7.63 (1H, m), 7.61 (1H, dd, J = 2.6, 1.5 Hz), 7.57 – 7.49 (3H, m), 7.41-7.38 (1H, m), 6.96 (1H, ddd, J = 8.2, 2.6, 1.0 Hz), 3.84 (3H, s). ^{13}C NMR (151 MHz, d_6-DMSO): δ = 166.9, 159.7, 155.0, 135.3, 133.0, 130.5, 130.0, 129.4, 126.3, 118.6, 115.1, 113.9, 111.6, 55.2. HRMS (ESI-[+H]) *m/z*: Calcd for $C_{16}H_{14}NOS$ 268.0796; Found 268.0785.

2-Phenyl-4-(*p*-tolyl)thiazole (5e)

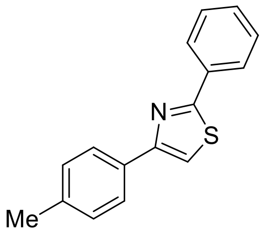

Preformed according to the general protocol with 4-methylbenzoyl (dimethyloxosulphonio)methanide (88 mg, 0.408 mmol) and thiobenzamide (84 mg, 0.612 mmol). The product was purified by automated flash column chromatography (hexane/EtOAc, 100:0 – 95:5, 12 g SiO_2) to give 2-phenyl-4-(*p*-tolyl)thiazole (**5e**) as a white solid (64 mg, 62%). LCMS (UV, ESI) R_t = 27.01 min, $[M-H]^+$ *m/z* = 252.0, 99% purity. ^{1}H NMR (600 MHz, d_6-DMSO): δ = 8.11 (1H, s), 8.05 – 8.00 (2H, m), 7.97 – 7.92 (2H, m), 7.57 – 7.48 (3H, m), 7.31 – 7.26 (2H, m), 2.35 (3H, s). ^{13}C NMR (151 MHz, d_6-DMSO): δ = 166.8, 155.3, 137.7, 133.1, 131.4, 130.4, 129.4, 129.3, 126.2, 126.1, 113.8, 20.9. HRMS (ESI-[+H]) *m/z*: Calcd for $C_{16}H_{14}NS$ 252.0847; Found 252.0852.

4-Phenyl-2-(pyrimidin-4-yl)thiazole (5g)

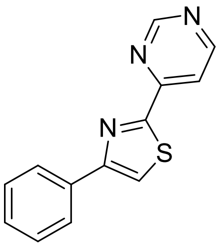

Preformed according to the general protocol with benzoyl(dimethyloxosulphonio)methanide (80 mg, 0.408 mmol) and pyrazine-2-thiocarboxamide (85 mg, 0.612 mmol). The product was purified by automated flash column chromatography (hexane/EtOAc, 100:0 – 80:20, 24 g SiO_2) to give 4-phenyl-2-(pyrimidin-4-yl)thiazole (**5g**) as a yellow solid (74 mg, 76%). LCMS (UV, ESI) R_t = 21.02 min, $[M-H]^+$ *m/z* = 239.8, 91% purity. ^{1}H NMR (600 MHz, d_6-DMSO): δ = 9.46 (1H, d, J = 1.5 Hz), 8.78 (1H, d, J = 2.6 Hz), 8.75 (1H, dd, J = 2.5, 1.5 Hz), 8.40 (1H, s), 8.12-8.08 (2H, m), 7.53-7.49 (2H, m), 7.42-

7.39 (1H, m). ^{13}C NMR (151 MHz, d_6-DMSO): δ = 165.6, 156.1, 146.0, 145.8, 144.6, 140.7, 133.6, 129.0, 128.6, 126.2, 117.9. HRMS (ESI-[+H]) *m/z*: Calcd for $C_{13}H_{10}N_3S$ 240.0595; Found 240.0582.

2-(Furan-2-yl)-4-phenylthiazole (5h)

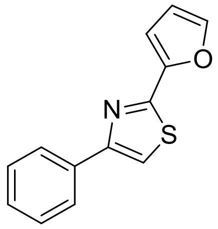

Preformed according to the general protocol with benzoyl(dimethyloxosulphonio)methanide (80 mg, 0.408 mmol) and furan-2-thiocarboxamide (79 mg, 0.612 mmol). The product was purified by automated flash column chromatography (hexane/EtOAc, 100:0 – 90:10, 12 g SiO_2) to give 2-(furan-2-yl)-4-phenylthiazole (**5h**) as a yellow gum (54 mg, 57%). LCMS (UV, ESI) R_t = 22.35 min, $[M-H]^+$ *m/z* = 228.1, 98% purity. ^{1}H NMR (600 MHz, d_6-DMSO): δ = 8.16 (1H, s), 8.01 (2H, dd, *J* = 8.2, 1.2 Hz), 7.92 (1H, dd, *J* = 1.8, 0.8 Hz), 7.47 (2H, dd, *J* = 8.3, 7.1 Hz), 7.40-7.38 (1H, m), 7.19 (1H, dd, *J* = 3.5, 0.8 Hz), 6.74 (1H, dd, *J* = 3.4, 1.8 Hz). ^{13}C NMR (151 MHz, d_6-DMSO): δ = 157.0, 155.1, 148.2, 144.9, 133.7, 128.9, 128.4, 126.2, 113.8, 112.7, 109.5. HRMS (ESI-[+H]) *m/z*: Calcd for $C_{13}H_{10}NOS$ 228.0483; Found 228.0473.

4-Phenyl-2-(pyridin-2-yl)thiazole (5i)

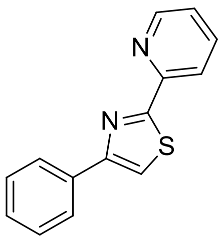

Preformed according to the general protocol with benzoyl(dimethyloxosulphonio)methanide (80 mg, 0.408 mmol) and 2-pyridinethioamide (85 mg, 0.612 mmol). The product was purified by automated flash column chromatography (hexane/EtOAc, 100:0 – 95:5, 12 g SiO_2) to give 4-phenyl-2-(pyridin-2-yl)thiazole (**5i**) as an off-white solid (54 mg, 55%). LCMS (UV, ESI) R_t = 23.21 min, $[M-H]^+$ *m/z* = 239.0, 98% purity. ^{1}H NMR (600 MHz, d_6-DMSO): δ = 8.66 (1H, ddd, *J* = 4.8, 1.7, 0.9 Hz), 8.28 (1H, s), 8.27-8.25 (1H, m), 8.09-8.07 (2H, m), 8.03-7.99 (1H, m), 7.53 (1H, ddd, *J* = 7.6, 4.8, 1.2 Hz), 7.51-7.47 (2H, m), 7.40-7.37 (1H, m). ^{13}C NMR (151 MHz, d_6-DMSO): δ = 168.7, 156.0, 150.8, 150.2,

138.3, 134.4, 129.3 128.7, 125.7, 119.7, 117.4. HRMS (ESI-[+H]) *m/z*: Calcd for $C_{14}H_{11}N_2S$ 239.0643; Found 239.0647.

4-Phenyl-2-(pyridin-4-yl)thiazole (5j)

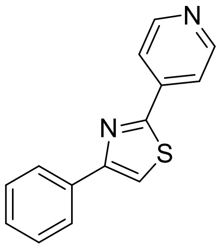

Preformed according to the general protocol with benzoyl(dimethyloxosulphonio)methanide (80 mg, 0.408 mmol) and 4-pyridinethioamide (85 mg, 0.612 mmol). The product was purified by automated flash column chromatography (hexane/EtOAc, 100:0 – 0:100, 12 g SiO_2) to give 4-phenyl-2-(pyridin-4-yl)thiazole (**5j**) as a yellow gum (69 mg, 71%). LCMS (UV, ESI) R_t = 13.46 min, $[M-H]^+$ *m/z* = 239.2, 99% purity. ^{1}H NMR (600 MHz, d_6-DMSO): δ = 8.75-8.73 (2H, m), 8.37 (1H, s), 8.11-8.05 (2H, m), 8.00-7.96 (2H, m), 7.54-7.47 (2H, m), 7.42-7.38 (1H, m). ^{13}C NMR (151 MHz, d_6 −DMSO): δ = 164.4, 155.9, 150.8, 139.5, 133.6, 129.0, 128.6, 126.3, 120.1, 116.8. HRMS (ESI-[+H]) *m/z*: Calcd for $C_{14}H_{11}N_2S$ 239.0643; Found 239.0458.

4-Phenyl-2-(*p*-tolyl)thiazole (5k)

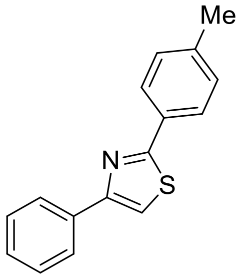

Preformed according to the general protocol with benzoyl(dimethyloxosulphonio)methanide (80 mg, 0.408 mmol) and 4-methylthiobenzamide (92 mg, 0.612 mmol). The product was purified by automated flash column chromatography (hexane/EtOAc, 100:0 – 90:10, 12 g SiO_2) to give 4-phenyl-2-(*p*-tolyl)thiazole (**5k**) as a white solid (59 mg, 58%). LCMS (UV, ESI) R_t = 26.64 min, $[M-H]^+$ *m/z* = 252.1, 97% purity. ^{1}H NMR (600 MHz, d_6-DMSO): δ = 8.15 (1H, s), 8.07-8.03 (2H, m), 7.95-7.90 (2H, m), 7.50-7.45 (2H, m), 7.40-7.33 (3H, m), 2.37 (3H, s). ^{13}C NMR (151 MHz, d_6 -DMSO): δ = 167.1, 155.0, 140.3, 134.0, 130.5, 129.9, 128.9, 128.2, 126.2, 126.2, 114.2, 21.0. HRMS (ESI-[+H]) *m/z*: Calcd for $C_{16}H_{14}NS$ 252.0847; Found 252.0852.

***tert*-Butyl ((4-phenylthiazol-2-yl)methyl)carbamate (5f)**

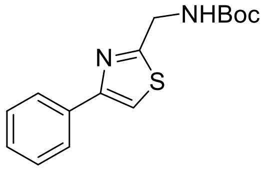

Preformed according to the general protocol with benzoyl(dimethyloxosulphonio)methanide (80 mg, 0.408 mmol) and *tert*-butyl(2-amino-2-thioxoethyl)carbamate (116 mg, 0.612 mmol). The product was purified by automated flash column chromatography (hexane/EtOAc, 100:0 – 80:20, 24 g SiO_2) to give *tert*-butyl ((4-phenylthiazol-2-yl)methyl)carbamate (**5f**) as a yellow gum (77 mg, 65%). LCMS (UV, ESI) R_t = 19.77 min, $[M-H]^+$ *m/z* = did not ionise, 99% purity. ^{1}H NMR (600 MHz, d_6-DMSO): δ = 8.00 (1H, s), 7.93 (2H, d, J = 7.7 Hz), 7.83 (1H, t, J = 6.2 Hz), 7.46-7.40 (2H, m), 7.33 (1H, t, J = 7.4 Hz), 4.44 (2H, d, J = 6.0 Hz), 1.42 (9H, s). ^{13}C NMR (151 MHz, d_6-DMSO): δ = 171.7, 156.2, 154.3, 134.6, 129.2, 128.4, 126.3, 114.4, 79.0, 42.5, 28.6. HRMS (ESI-[+H]) *m/z*: Calcd for $C_{15}H_{19}N_2O_2S$ 291.1167; Found 291.1181.

Insertion/Cyclisation of Thioureas

N,4-Diphenylthiazol-2-amine (3a)

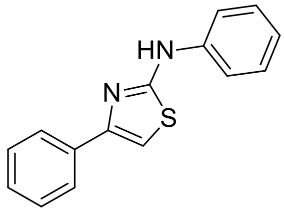

Preformed according to the general protocol with benzoyl(dimethyloxosulphonio)methanide (80 mg, 0.408 mmol) and *N*-phenylthiourea (93 mg, 0.612 mmol). The product was purified by automated flash column chromatography (hexane/EtOAc, 100:0 – 85:15, 24 g SiO_2) to give *N*,4-diphenylthiazol-2-amine (**3a**) as a light orange solid (95 mg, 92%). LCMS (UV, ESI) R_t = 22.78 min, $[M\text{-}H]^+$ *m/z* = 253.0, 97% purity. ^{1}H NMR (600 MHz, d_6-DMSO): δ = 10.29 (1H, s), 7.93 (2H, dd, J = 8.2, 1.3 Hz), 7.76-7.71 (2H, m), 7.46-7.41 (2H, m), 7.38-7.29 (4H, m), 6.96 (1H, tt, J = 7.3, 1.2 Hz). ^{13}C NMR (151 MHz, d_6-DMSO): δ = 163.1, 151.1, 141.2, 134.6, 129.1, 128.7, 127.6, 125.7, 121.2, 116.8, 103.0. HRMS (ESI-[+H]) *m/z*: Calcd for $C_{15}H_{13}N_2S$ 253.0799; Found 253.0793.

N-Phenethyl-4-phenylthiazol-2-amine (Fanetizole) (3b)

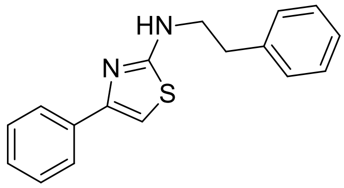

Preformed according to the general protocol with benzoyl(dimethyloxosulphonio)methanide (80 mg, 0.408 mmol) and 2-phenylethylthiourea (110 mg, 0.612 mmol). The product was purified by automated flash column chromatography (hexane/EtOAc, 100:0 – 90:10, 24 g SiO_2) to give *N*-phenethyl-4-phenylthiazol-2-amine (**3b**) as a white solid (95 mg, 83%). LCMS (UV, ESI) R_t = 19.44 min, $[M\text{-}H]^+$ *m/z* = 280.9, 91% purity. ^{1}H NMR (600 MHz, d_6-DMSO): δ = 7.85-7.82 (2H, m), 7.78 (1H, t, J = 5.4 Hz), 7.40-7.34 (2H, m), 7.34-7.24 (5H, m), 7.23-7.19 (1H, m), 7.06 (1H, s), 3.49 (2H, td, J = 7.5, 5.5 Hz), 2.91 (2H, t, J = 7.4 Hz). ^{13}C NMR (151 MHz, d_6-DMSO): δ = 168.2, 150.0, 139.5, 135.0, 128.8, 128.6, 128.4, 127.3, 126.2, 125.6, 100.9, 46.2, 34.8. HRMS (ESI-[+H]) *m/z*: Calcd for $C_{17}H_{17}N_2S$ 281.1112; Found 281.1120.

4-Phenylthiazol-2-amine (3c)

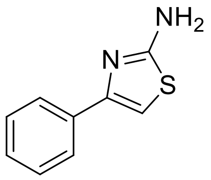

Preformed according to the general protocol with benzoyl(dimethyloxosulphonio)methanide (80 mg, 0.408 mmol) and thiourea (31 mg, 0.612 mmol). The product was purified by automated flash column chromatography (hexane/EtOAc, 100:0 – 0:100, 24 g SiO_2) to give 4-phenylthiazol-2-amine (**3c**) as a white solid (58 mg, 81%). LCMS (UV, ESI) R_t = 7.23 min, $[M-H]^+$ *m/z* = 176.8, 92% purity. ^{1}H NMR (600 MHz, d_6-DMSO): δ = 7.81 – 7.76 (2H, m), 7.38 – 7.33 (2H, m), 7.26-7.23 (1H, m), 7.06 (2H, s), 7.01 (1H, s). ^{13}C NMR (151 MHz, d_6-DMSO): δ = 168.2, 149.9, 134.9, 128.5, 127.2, 125.5, 101.5. HRMS (ESI-[+H]) *m/z*: Calcd for $C_9H_9N_2S$ 177.0486; Found 177.0486.

N-Benzyl-4-phenylthiazol-2-amine (3d)

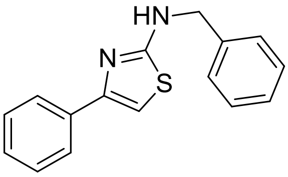

Preformed according to the general protocol with benzoyl(dimethyloxosulphonio)methanide (80 mg, 0.408 mmol) and benzyl thiourea (102 mg, 0.612 mmol). The product was purified by automated flash column chromatography (hexane/EtOAc, 100:0 – 85:15, 24 g SiO_2) to give *N*-benzyl-4-phenylthiazol-2-amine (**3d**) as a yellow solid (94 mg, 82%). LCMS (UV, ESI) R_t = 19.48 min, $[M-H]^+$ *m/z* = 266.9, 96% purity. ^{1}H NMR (600 MHz, d_6-DMSO): δ = 8.20 (1H, t, J = 5.9 Hz), 7.84-7.79 (2H, m), 7.42-7.38 (2H, m), 7.38-7.32 (4H, m), 7.28-7.22 (2H, m), 7.06 (1H, s), 4.50 (2H, d, J = 5.9 Hz). ^{13}C NMR (151 MHz, d_6-DMSO): δ = 168.3, 149.9, 139.3, 134.9, 128.5, 128.4, 127.6, 127.3, 127.0, 125.6, 101.2, 47.8. HRMS (ESI-[+H]) *m/z*: Calcd for $C_{16}H_{15}N_2S$ 267.0956; Found 267.0954.

tert-Butyl (4-phenylthiazol-2-yl)carbamate (3e)

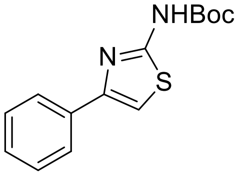

Preformed according to the general protocol with benzoyl(dimethyloxosulphonio)methanide (80 mg, 0.408 mmol) and *N*-boc-thiourea (108 mg, 0.612 mmol). The product was purified by automated flash column chromatography (hexane/EtOAc, 100:0 – 85:15, 24 g SiO_2) to give *tert*-butyl (4-phenylthiazol-2-yl)carbamate (**3e**) as an off-white solid (62 mg, 55%). LCMS (UV, ESI) R_t = 22.22 min, $[M-H]^+$ *m/z*

= did not ionise, 93% purity. ^{1}H NMR (600 MHz, $CDCl_3$): δ = 9.86 (1H, s), 7.82 (2H, d, J = 7.0 Hz), 7.43-7.38 (2H, m), 7.33-7.29 (1H, m), 7.11 (1H, s), 1.35 (9H, s). ^{13}C NMR (151 MHz, $CDCl_3$): δ = 160.5, 150.1, 134.5, 128.8, 128.6, 128.0, 126.2, 106.8, 82.4, 28.0. HRMS (ESI-[+H]) *m/z*: Calcd for $C_{14}H_{17}N_2O_2S$ 277.1011; Found 277.1028.

4-Phenyl-*N*-((tetrahydrofuran-2-yl)methyl)thiazol-2-amine (3f)

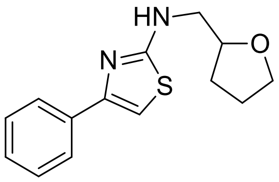

Preformed according to the general protocol with benzoyl(dimethyloxosulphonio)methanide (80 mg, 0.408 mmol) and 1-(2-tetrahydrofurfuryl)-2-thiourea (101 mg, 0.612 mmol). The product was purified by automated flash column chromatography (hexane/EtOAc, 100:0 – 90:10, 12 g SiO_2) to give 4-phenyl-*N*-((tetrahydrofuran-2-yl)methyl)thiazol-2-amine (**3f**) as a yellow solid (81 mg, 76%). LCMS (UV, ESI) R_t = 12.67 min, $[M-H]^+$ *m/z* = 261.2, 93% purity. ^{1}H NMR (600 MHz, d_6-DMSO): δ = 7.84-7.79 (2H, m), 7.73 (1H, t, J = 5.7 Hz), 7.39-7.33 (2H, m), 7.27-7.23 (1H, m), 7.03 (1H, s), 4.07-4.00 (1H, m), 3.79 (1H, td, J = 7.6, 6.0 Hz), 3.64 (1H, td, J = 7.8, 6.4 Hz), 3.41-3.30 (2H, m), 1.97-1.91 (1H, m), 1.90-1.77 (2H, n), 1.60 (1H, ddt, J = 12.0, 8.7, 6.8 Hz). ^{13}C NMR (151 MHz, d_6-DMSO): δ = 168.5, 149.8, 135.0, 128.5, 127.3, 125.6, 100.9, 76.9, 67.2, 48.5, 28.7, 25.2. HRMS (ESI-[+H]) *m/z*: Calcd for $C_{14}H_{17}N_2OS$ 261.1062; Found 261.1059.

N-(4-Fluorophenyl)-4-phenylthiazol-2-amine (3g)

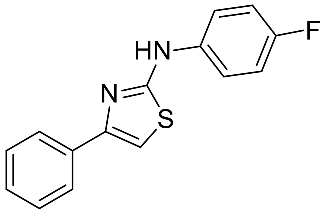

Preformed according to the general protocol with benzoyl(dimethyloxosulphonio)methanide (80 mg, 0.408 mmol) and (4-fluorophenyl)thiourea (104 mg, 0.612 mmol). The product was purified by automated flash column chromatography (hexane/EtOAc, 100:0 – 90:10, 5 g SiO_2) to give *N*-(4-fluorophenyl)-4-phenylthiazol-2-amine (**3g**) as a yellow solid (94 mg, 82%). LCMS (UV, ESI) R_t = 23.00 min, $[M-H]^+$ *m/z* = 271.0, 94% purity. ^{1}H NMR (600 MHz, d_6-DMSO): δ = 10.31 (s, 1H), 7.92 (2H, dd, J = 8.3, 1.4 Hz), 7.79-7.73 (2H, m), 7.46-7.40 (2H, m), 7.36-7.28 (2H, m), 7.23-7.16 (2H, m). ^{13}C NMR (151 MHz, d_6-DMSO): δ = 163.1, 156.8 (d, $^1J_{C-F}$ = 237.4 Hz), 150.0, 137.7 (d, $^4J_{C-F}$ = 2.2 Hz), 134.5, 128.6, 127.6, 125.7, 118.3 (d, $^3J_{C-F}$ = 7.5 Hz), 115.5 (d, $^2J_{C-F}$ = 22.4 Hz), 102.9. ^{19}F NMR

(376 MHz, d_6-DMSO) δ = -122.12. HRMS (ESI-[+H]) *m/z*: Calcd for $C_{15}H_{12}N_2FS$ 271.0705; Found 271.0722.

N-(4-Phenylthiazol-2-yl)benzamide (3h)

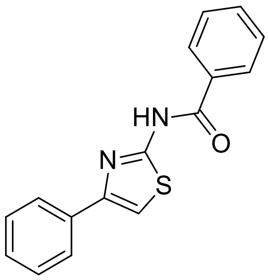

Preformed according to the general protocol with benzoyl(dimethyloxosulphonio)methanide (80 mg, 0.408 mmol) and benzoylthiourea (110 mg, 0.612 mmol). The product was purified by automated flash column chromatography (hexane/EtOAc, 100:0 – 90:10, 5 g SiO_2) to give *N*-(4-phenylthiazol-2-yl)benzamide (**3h**) as a white solid (96 mg, 84%). LCMS (UV, ESI) R_t = 21.59 min, $[M-H]^+$ *m/z* = 281.0, 99% purity. ^{1}H NMR (600 MHz, d_6-DMSO): δ = 12.81 (1H, s), 8.16-8.11 (2H, m), 7.71 (1H, s), 7.67-7.63 (1H, m), 7.59-7.53 (2H, m), 7.48-7.42 (2H, m), 7.36-7.32 (1H, m). ^{13}C NMR (151 MHz, d_6-DMSO): δ = 165.3, 158.6, 149.2, 134.4, 132.7, 132.0, 128.8, 128.6, 128.2, 127.9, 125.8, 108.6. HRMS (ESI-[+H]) *m/z*: Calcd for $C_{16}H_{13}N_2OS$ 281.0749; Found 281.0743.

N-(Furan-2-ylmethyl)-4-phenylthiazol-2-amine (3i)

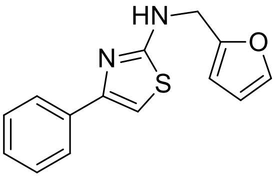

Preformed according to the general protocol with benzoyl(dimethyloxosulphonio)methanide (80 mg, 0.408 mmol) and 1-(2-furfuryl)-2-thiourea (96 mg, 0.612 mmol). The product was purified by automated flash column chromatography (hexane/EtOAc, 100:0 – 90:10, 12 g SiO_2) to give *N*-(furan-2-ylmethyl)-4-phenylthiazol-2-amine (**3i**) a white solid (46 mg, 43%). LCMS (UV, ESI) R_t = 18.64 min, $[M-H]^+$ *m/z* = did not ionise, 97% purity. ^{1}H NMR (600 MHz, d_6-DMSO): δ = 8.08 (1H, t, *J* = 5.7 Hz), 7.86-7.81 (2H, m), 7.61 (1H, dd, *J* = 1.9, 0.9 Hz), 7.40-7.34 (2H, m), 7.29-7.24 (1H, m), 7.09 (1H, s), 6.41 (1H, dd, *J* = 3.2, 1.9 Hz), 6.37 (1H, dd, *J* = 3.2, 0.9 Hz), 4.50 (2H, d, *J* = 5.9 Hz). ^{13}C NMR (151 MHz, d_6-DMSO): δ = 167.8, 152.1, 149.8, 142.3, 134.8, 128.5, 127.3, 125.6, 110.5, 107.5, 101.5, 40.8. HRMS (ESI-[+H]) *m/z*: Calcd for $C_{14}H_{13}N_2OS$ 257.0749; Found 257.0753.

N-(2-Methoxyethyl)-4-phenylthiazol-2-amine (3j)

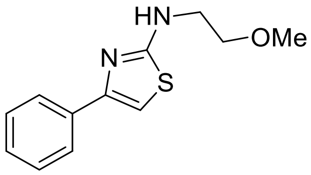

Preformed according to the general protocol with benzoyl(dimethyloxosulphonio)methanide (80 mg, 0.408 mmol) and 1-(2-methoxyethyl)-2-thiourea (82 mg, 0.612 mmol). The product was purified by automated flash column chromatography (hexane/EtOAc, 100:0 – 90:10, 12 g SiO_2) to give *N*-(2-methoxyethyl)-4-phenylthiazol-2-amine (**3j**) as a yellow gum (80 mg, 84%). LCMS (UV, ESI) R_t = 11.82 min, $[M-H]^+$ *m/z* = 235.0, 93% purity. ^{1}H NMR (600 MHz, d_6-DMSO): δ = 7.84-7.80 (2H, m), 7.72 (1H, t, J = 5.6 Hz), 7.39-7.34 (2H, m), 7.27-7.24 (1H, m), 7.04 (1H, s), 3.52 (2H, t, J = 5.7 Hz), 3.46 (2H, q, J = 5.3 Hz), 3.28 (3H, s). ^{13}C NMR (151 MHz, d_6-DMSO): δ = 168.2, 149.8, 134.9, 128.5, 127.2, 125.6, 100.9, 70.3, 58.0, 43.9. HRMS (ESI-[+H]) *m/z*: Calcd for $C_{12}H_{15}N_2OS$ 235.0905; Found 235.0896.

4-((4-Phenylthiazol-2-yl)amino)phenol (3k)

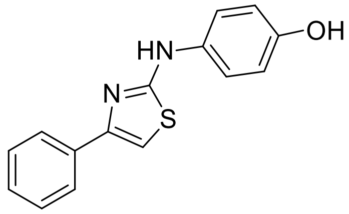

Preformed according to the general protocol with benzoyl(dimethyloxosulphonio)methanide (80 mg, 0.408 mmol) and 1-(4-hydroxyphenyl)thiourea (103 mg, 0.612 mmol). The product was purified by automated reverse phase chromatography (H_2O (1% formic acid)/Acetonitrile (1% formic acid), 95:5 – 5:95, 12 g C28) to give 4-((4-phenylthiazol-2-yl)amino)phenol (**3k**) as a light brown solid (59 mg, 54%). LCMS (UV, ESI) R_t = 17.63 min, $[M-H]^+$ *m/z* = 269.3, 99% purity. ^{1}H NMR (600 MHz, d_6-DMSO): δ = 9.92 (1H, s), 9.14 (1H, s), 7.89 (2H, d, J = 7.0 Hz), 7.51-7.45 (2H, m), 7.43-7.39 (2H, m), 7.31-7.28 (1H, m), 7.22 (1H, s), 6.78-6.73 (2H, m). ^{13}C NMR (151 MHz, d_6-DMSO): δ = 164.2, 152.3, 150.3, 134.7, 133.3, 128.6, 127.5, 125.6, 119.2, 115.5, 101.9. HRMS (ESI-[+H]) *m/z*: Calcd for $C_{15}H_{13}N_2OS$ 269.0749; Found 269.0734.

4-(4-Phenylthiazol-2-yl)morpholine (3l)

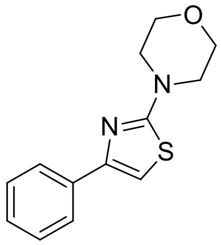

Preformed according to the general protocol with benzoyl(dimethyloxosulphonio)methanide (80 mg, 0.408 mmol) and morpholine-4-carbothioamide (89 mg, 0.612 mmol). The product was purified by automated flash column chromatography (hexane/EtOAc, 100:0 – 90:10, 12 g SiO_2) to give 4-(4-phenylthiazol-2-yl)morpholine (**3l**) as a pale yellow solid (63 mg, 63%). LCMS (UV, ESI) R_t = 20.62 min, $[M-H]^+$ *m/z* = 246.9, 97% purity. ^1H NMR (600 MHz, d_6-DMSO): δ = 7.88-7.83 (2H, m), 7.41-7.36 (2H, m), 7.32 (1H, s), 7.30-7.27 (1H, m), 3.76-3.71 (4H, m), 3.46-3.41 (4H, m). ^{13}C NMR (151 MHz, d_6-DMSO): δ = 170.8, 150.6, 134.6, 128.6, 127.6, 125.7, 102.8, 65.5, 48.2. HRMS (ESI-[+H]) *m/z*: Calcd for $C_{13}H_{15}N_2OS$ 247.0905; Found 247.0894.

N-Allyl-4-phenylthiazol-2-amine (3m)

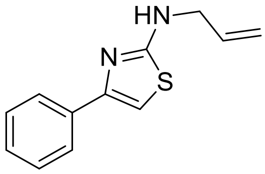

Preformed according to the general protocol with benzoyl(dimethyloxosulphonio)methanide (80 mg, 0.408 mmol) and 1-allyl-2-thiourea (71 mg, 0.612 mmol). The product was purified by automated flash column chromatography (hexane/EtOAc, 100:0 – 90:10, 12 g SiO_2) to give *N*-allyl-4-phenylthiazol-2-amine (**3m**) as a yellow gum (78 mg, 88%). LCMS (UV, ESI) R_t = 14.71 min, $[M-H]^+$ *m/z* = 217.2, 92% purity. ^1H NMR (600 MHz, d_6-DMSO): δ = 7.85-7.80 (3H, m), 7.39-7.34 (2H, m), 7.28-7.24 (1H, m), 7.07 (1H, s), 5.93 (1H, ddt, J = 17.2, 10.5, 5.4 Hz), 5.27 (1H, dq, J = 17.1, 1.8 Hz), 5.13 (1H, dq, J = 10.3, 1.7 Hz), 3.96-3.90 (2H, m). ^{13}C NMR (151 MHz, d_6-DMSO): δ = 168.3, 149.9, 135.0, 134.9, 128.5, 127.3, 125.6, 115.9, 101.1, 46.7. HRMS (ESI-[+H]) *m/z*: Calcd for $C_{12}H_{13}N_2S$ 217.0799; Found 217.0788.

N-Cyclohexyl-4-phenylthiazol-2-amine (3n)

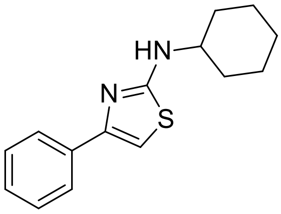

Preformed according to the general protocol with benzoyl(dimethyloxosulphonio)methanide (80 mg, 0.408 mmol) and 1-cyclohexyl-2-thiourea (97 mg, 0.612 mmol). The product was purified by automated flash column chromatography (hexane/EtOAc, 100:0 – 80:20, 12 g SiO_2) to give *N*-cyclohexyl-4-phenylthiazol-2-amine (**3n**) as a yellow solid (75 mg, 71%). LCMS (UV, ESI) R_t = 18.30 min, $[M-H]^+$ *m/z* = 259.3, 90% purity. ^{1}H NMR (600 MHz, d_6-DMSO): δ = 7.82-7.79 (2H, m), 7.59 (1H, d, *J* = 7.5 Hz), 7.39-7.33 (2H, m), 7.27-7.23 (1H, m), 7.00 (1H, s), 3.50 (1H, tdt, *J* = 10.5, 7.4, 3.6 Hz), 1.98 (2H, dt, *J* = 11.7, 4.0 Hz), 1.72 (2H, dq, *J* = 12.9, 3.9 Hz), 1.57 (1H, dt, *J* = 12.9, 3.9 Hz), 1.38-1.12 (5H, m). ^{13}C NMR (151 MHz, d_6-DMSO): δ = 167.5, 150.0, 135.0, 128.5, 127.2, 125.6, 100.4, 53.4, 32.3, 25.4, 24.5. HRMS (ESI-[+H]) *m/z*: Calcd for $C_{15}H_{19}N_2S$ 259.1269; Found 259.1278.

***N*-Isopropyl-4-phenylthiazol-2-amine (3o)**

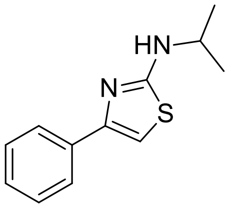

Preformed according to the general protocol with benzoyl(dimethyloxosulphonio)methanide (80 mg, 0.408 mmol) and 1-isopropylthiourea (72 mg, 0.612 mmol). The product was purified by automated flash column chromatography (hexane/EtOAc, 100:0 – 90:10, 12 g SiO_2) to give *N*-isopropyl-4-phenylthiazol-2-amine (**3o**) as a yellow solid (76 mg, 85%). LCMS (UV, ESI) R_t = 17.75 min, $[M-H]^+$ *m/z* = 219.1, 96% purity. ^{1}H NMR (600 MHz, $CDCl_3$): δ = 7.80-7.75 (2H, m), 7.37 (2H, dd, *J* = 8.4, 7.0 Hz), 7.31-7.27 (1H, m), 6.67 (1H, s), 3.68 (1H, h, *J* = 6.6 Hz), 1.32 (6H, *d*, J = 6.4 Hz) (One proton not observed). ^{13}C NMR (151 MHz, $CDCl_3$): δ = 168.8, 129.2, 128.8, 128.0, 127.4, 126.1, 100.3, 48.6, 22.9. HRMS (ESI-[+H]) *m/z*: Calcd for $C_{12}H_{15}N_2S$ 219.0956; Found 219.0977.

***N-(tert*-Butyl)-4-phenylthiazol-2-amine (3p)**

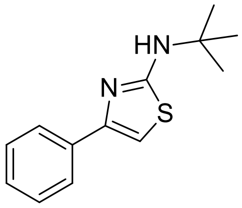

Preformed according to the general protocol with benzoyl(dimethyloxosulphonio)methanide (80 mg, 0.408 mmol) and *tert*-butylthiourea (81 mg, 0.612 mmol). The product was purified by automated flash column chromatography (hexane/EtOAc, 100:0 – 50:50, 24 g SiO_2) to give *N-(tert*-butyl)-4-phenylthiazol-2-amine (**3p**) as a yellow gum (84 mg, 89%). LCMS (UV, ESI) R_t = 20.54 min, $[M-H]^+$

m/z = 233.2, 99% purity. ^{1}H NMR (600 MHz, d_6-DMSO): δ = 7.85-7.80 (2H, m), 7.40-7.35 (3H, m), 7.27-7.23 (1H, m), 7.00 (1H, s), 1.42 (9H, s). ^{13}C NMR (151 MHz, d_6-DMSO): δ = 166.1, 149.8, 135.1, 128.5, 127.2, 125.6, 100.7, 52.5, 28.5. HRMS (ESI-[+H]) m/z: Calcd for $C_{13}H_{17}N_2S$ 233.1112; Found 233.1113.

N-(2-Morpholinoethyl)-4-phenylthiazol-2-amine (3q)

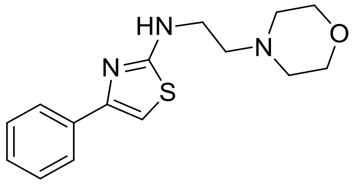

Preformed according to the general protocol with benzoyl(dimethyloxosulphonio)methanide (80 mg, 0.408 mmol) and 1-(2-morpholinoethyl)-2-thiourea (116 mg, 0.612 mmol). The product was purified by automated flash column chromatography (hexane/EtOAc, 100:0 – 0:100, 24 g SiO_2) to give *N*-(2-morpholinoethyl)-4-phenylthiazol-2-amine (**3q**) as a yellow gum (80 mg, 68%). LCMS (UV, ESI) R_t = 6.89 min, $[M-H]^+$ m/z = 290.3, 98% purity. ^{1}H NMR (600 MHz, d_6-DMSO): δ = 7.82 (2H, d, J = 7.7 Hz), 7.57 (1H, t, J = 5.5 Hz), 7.39-7.33 (2H, m), 7.25 (1H, t, J = 7.3 Hz), 7.04 (1H, s), 3.58 (4H, t, J = 4.5 Hz), 3.44-3.38 (2H, m), 2.53 (2H, t, J = 6.7 Hz), 2.47-2.39 (4H, m). ^{13}C NMR (151 MHz, d_6-DMSO): δ = 168.3, 149.9, 135.0, 128.0, 127.3, 125.6, 100.8, 66.2, 57.1, 53.4, 41.6. HRMS (ESI-[+H]) m/z: Calcd for $C_{15}H_{20}N_3OS$ 290.1327; Found 290.1284.

N-Benzyl-4-(2-methoxyphenyl)thiazol-2-amine (3r)

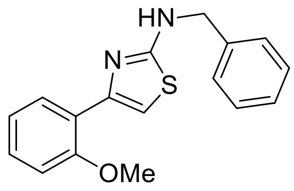

Preformed according to the general protocol with 2-methoxybenzoyl(dimethyloxosulphonio)methanide (92 mg, 0.408 mmol) and *N*-benzyllthiourea (93 mg, 0.612 mmol). The product was purified by automated flash column chromatography (hexane/EtOAc, 100:0 – 90:10, 12 g SiO_2) to give *N*-benzyl-4-(2-methoxyphenyl)thiazol-2-amine (**3r**) as a white solid (109 mg, 90%). LCMS (UV, ESI) R_t = 16.78 min, $[M-H]^+$ m/z = 297.4, 95% purity. ^{1}H NMR (600 MHz, d_6-DMSO): δ = 8.10-8.04 (2H, m), 7.42-7.37 (2H, m), 7.36-7.31 (2H, m), 7.27-7.22 (2H, m), 7.16 (1H, s), 7.06 (1H, dd, J = 8.4, 1.1 Hz), 6.99-6.96 (1H, m), 4.50 (2H, d, J = 5.9 Hz), 3.87 (3H, s). ^{13}C NMR (151 MHz, d_6-DMSO): δ = 166.4, 156.6,

145.8, 139.5, 129.4, 128.3, 128.2, 127.6, 127.0, 123.1, 120.4, 111.4, 105.4, 55.4, 47.8. HRMS (ESI-[+H]) *m/z*: Calcd for $C_{17}H_{17}N_2OS$ 297.1062; Found 297.1057.

N-Benzyl-4-(2-methoxyphenyl)thiazol-3-amine (3s)

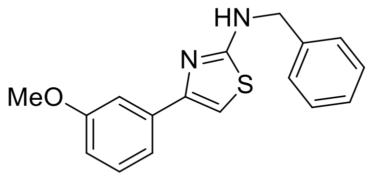

Preformed according to the general protocol with 3-methoxybenzoyl(dimethyloxosulphonio)methanide (92 mg, 0.408 mmol) and *N*-benzyllthiourea (93 mg, 0.612 mmol). The product was purified by automated flash column chromatography (hexane/EtOAc, 100:0 – 90:10, 12 g SiO_2) to give *N*-benzyl-4-(2-methoxyphenyl)thiazol-3-amine (**3s**) as a yellow gum (91 mg, 75%). LCMS (UV, ESI) R_t = 20.58 min, $[M-H]^+$ *m/z* = 297.4, 95% purity. ^{1}H NMR (600 MHz, d_6-DMSO): δ = 8.21 (1H, t, *J* = 5.9 Hz), 7.43-7.36 (4H, m), 7.34 (2H, dd, *J* = 8.5, 6.9 Hz), 7.29-7.23 (2H, m), 7.09 (1H, s), 6.83 (1H, ddd, *J* = 8.2, 2.6, 1.0 Hz), 4.49 (2H, d, *J* = 5.8 Hz), 3.78 (3H, s). ^{13}C NMR (151 MHz, d_6-DMSO): δ = 168.2, 159.4, 149.7, 139.3, 136.3, 129.5, 128.3, 127.6, 127.0, 118.0, 112.9, 111.1, 101.6, 55.0, 47.9. HRMS (ESI-[+H]) *m/z*: Calcd for $C_{17}H_{17}N_2OS$ 297.1062; Found 297.1057.

N-Benzyl-4-(2-methoxyphenyl)thiazol-4-amine (3t)

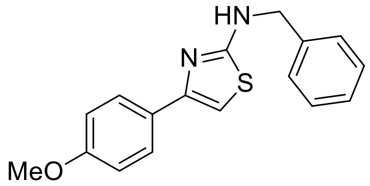

Preformed according to the general protocol with 4-methoxybenzoyl(dimethyloxosulphonio)methanide (92 mg, 0.408 mmol) and *N*-benzyllthiourea (93 mg, 0.612 mmol). The product was purified by automated flash column chromatography (hexane/EtOAc, 100:0 – 90:10, 12 g SiO_2) to give *N*-benzyl-4-(2-methoxyphenyl)thiazol-4-amine (**3t**) as a yellow solid (72 mg, 60%). LCMS (UV, ESI) R_t = 19.11 min, $[M-H]^+$ *m/z* = 297.3, 99% purity. ^{1}H NMR (600 MHz, d_6-DMSO): δ = 8.15 (1H, t, *J* = 5.9 Hz), 7.77-7.73 (2H, m), 7.42-7.38 (2H, m), 7.36-7.31 (2H, m), 7.27-7.23 (1H, m), 6.95-6.91 (2H, m), 6.88 (1H, s), 4.49 (2H, d, *J* = 5.9 Hz), 3.76 (3H, s). ^{13}C NMR (151 MHz, d_6-DMSO): δ = 168.3, 158.6, 149.8, 139.4, 128.3, 127.8, 127.6, 127.0, 126.9, 113.8, 99.0, 55.1, 47.8. HRMS (ESI-[+H]) *m/z*: Calcd for $C_{17}H_{17}N_2OS$ 297.1062; Found 297.1057.

N-Benzyl-4-(thiophen-2-yl)thiazol-2-amine (3u)

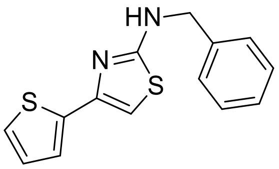

Preformed according to the general protocol with 2-thiophenecarbonyl (dimethyloxosulphonio)methanide (83 mg, 0.408 mmol) and *N*-benzyllthiourea (93 mg, 0.612 mmol). The product was purified by automated flash column chromatography (hexane/EtOAc, 100:0 – 90:10, 12 g SiO_2) to give *N*-benzyl-4-(thiophen-2-yl)thiazol-2-amine (**3u**) as a light brown solid (73 mg, 66%). LCMS (UV, ESI) R_t = 21.25 min, $[M-H]^+$ *m/z* = 273.2, 97% purity. ^{1}H NMR (600 MHz, d_6-DMSO): δ = 8.27 (1H, t, *J* = 5.9 Hz), 7.42-7.37 (4H, m), 7.34 (2H, dd, *J* = 8.5, 6.8 Hz), 7.27-7.23 (1H, m), 7.04 (1H, dd, *J* = 5.0, 3.6 Hz), 6.89 (1H, s), 4.45 (2H, d, *J* = 5.9 Hz). ^{13}C NMR (151 MHz, d_6-DMSO): δ = 168.4, 144.7, 139.1, 139.0, 128.4, 127.8, 127.7, 127.1, 125.0, 123.0, 99.6, 47.9. HRMS (ESI-[+H]) *m/z*: Calcd for $C_{14}H_{13}N_2S_2$ 273.0520; Found 273.0514.

N-Benzyl-4-(*p*-tolyl)thiazol-2-amine (3v)

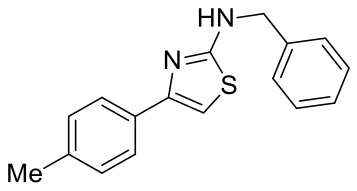

Preformed according to the general protocol with 4-methylbenzoyl(dimethyloxosulphonio)methanide (86 mg, 0.408 mmol) and *N*-benzyllthiourea (93 mg, 0.612 mmol). The product was purified by automated flash column chromatography (hexane/EtOAc, 100:0 – 90:10, 12 g SiO_2) to give *N*-benzyl-4-(*p*-tolyl)thiazol-2-amine (**3v**) a white solid (99 mg, 87%). LCMS (UV, ESI) R_t = 21.61 min, $[M-H]^+$ *m/z* = 281.4, 95% purity. ^{1}H NMR (600 MHz, d_6-DMSO): δ = 8.15 (1H, t, *J* = 5.9 Hz), 7.72-7.68 (2H, m), 7.42-7.38 (2H, m), 7.36-7.32 (2H, m), 7.27-7.23 (1H, m), 7.17 (2H, d, *J* = 8.0 Hz), 6.98 (1H, s), 4.50 (2H, d, *J* = 5.8 Hz), 2.29 (3H, s). ^{13}C NMR (151 MHz, d_6-DMSO): δ = 168.2, 149.9, 139.3, 136.5, 132.2, 129.1, 128.3, 127.6, 127.0, 125.6, 100.3, 47.8, 20.8. HRMS (ESI-[+H]) *m/z*: Calcd for $C_{17}H_{17}N_2S$ 281.1112; Found 281.1120.

N-Benzyl-4-(4-chlorophenyl)thiazol-2-amine (3w)

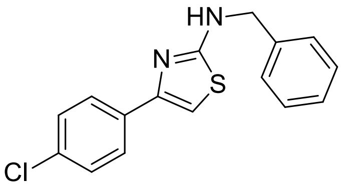

Preformed according to the general protocol with 4-chlorobenzoyl(dimethyloxosulphonio)methanide (94 mg, 0.408 mmol) and *N*-benzyllthiourea (93 mg, 0.612 mmol). The product was purified by automated flash column chromatography (hexane/EtOAc, 100:0 – 90:10, 12 g SiO_2) to give *N*-benzyl-4-(4-chlorophenyl)thiazol-2-amine (**3w**) a white solid (66 mg, 54%). LCMS (UV, ESI) R_t = 23.69 min, $[M-H]^+$ *m/z* = 300.9, 97% purity. ^{1}H NMR (600 MHz, d_6-DMSO): δ = 8.22 (1H, t, J = 5.9 Hz), 7.86-7.81 (2H, m), 7.45-7.40 (2H, m), 7.40-7.37 (2H, m), 7.36-7.32 (2H, m), 7.27-7.23 (1H, m), 7.14 (1H, s), 4.51 (2H, d, J = 5.9 Hz). ^{13}C NMR (151 MHz, d_6-DMSO): δ = 168.4, 148.6, 139.2, 133.7, 131.6, 128.5, 128.4, 127.6, 127.3, 127.0, 102.1, 47.8. HRMS (ESI-[+H]) *m/z*: Calcd for $C_{16}H_{14}ClN_2S$ 301.0566; Found 301.0579.

N-Benzyl-4-(4-bromophenyl)thiazol-2-amine (3x)

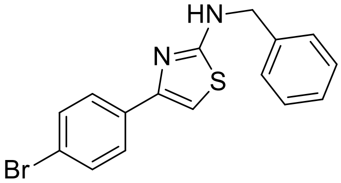

Preformed according to the general protocol with 4-bromobenzoyl(dimethyloxosulphonio)methanide (112 mg, 0.408 mmol) and *N*-benzyllthiourea (93 mg, 0.612 mmol). The product was purified by automated flash column chromatography (hexane/EtOAc, 100:0 – 90:10, 12 g SiO_2) to give *N*-benzyl-4-(4-bromophenyl)thiazol-2-amine (**3x**) as a white solid (74 mg, 53%). LCMS (UV, ESI) R_t = 24.36 min, $[M-H]^+$ *m/z* = 253.0, 99% purity. ^{1}H NMR (600 MHz, d_6-DMSO): δ = 8.23 (1H, t, J = 5.9 Hz), 7.78-7.75 (2H, m), 7.58-7.53 (2H, m), 7.41-7.37 (2H, m), 7.34 (2H, dd, J = 8.4, 6.9 Hz), 7.27-7.23 (1H, m), 7.15 (1H, s), 4.51 (2H, d, J = 5.8 Hz). ^{13}C NMR (151 MHz, d_6-DMSO): δ = 168.4, 148.7, 139.2, 134.1, 131.4, 128.4, 127.6, 127.6, 127.0, 120.2, 102.2, 47.8. HRMS (ESI-[+H]) *m/z*: Calcd for $C_{16}H_{14}BrN_2S$ 345.0061; Found 345.0061.

N-Benzyl-4-(4-fluorophenyl)thiazol-2-amine (3y)

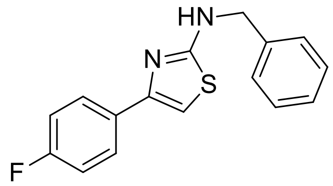

Preformed according to the general protocol with 4-fluorobenzoyl(dimethyloxosulphonio)methanide (87 mg, 0.408 mmol) and *N*-benzyllthiourea (93 mg, 0.612 mmol). The product was purified by automated flash column chromatography (hexane/EtOAc, 100:0 – 90:10, 12 g SiO_2) to give *N*-benzyl-4-(4-fluorophenyl)thiazol-2-amine (**3y**) as a white solid (55 mg, 47%). LCMS (UV, ESI) R_t = 21.57 min, $[M-H]^+$ *m/z* = 285.3, 97% purity. ^{1}H NMR (600 MHz, d_6-DMSO): δ = 8.20 (1H, t, J = 5.9 Hz), 7.87-7.83 (2H, m), 7.39 (2H, dd, J = 8.1, 1.4 Hz), 7.34 (2H, dd, J = 8.5, 6.9 Hz), 7.27-7.24 (1H, m), 7.21-7.17 (2H, m), 7.04 (1H, s), 4.50 (2H, d, J = 5.8 Hz). ^{13}C NMR (151 MHz, d_6-DMSO): δ = 168.4, 161.5 (d, $^1J_{C-F}$ = 244.6 Hz), 148.8, 139.3, 131.5 (d, $^4J_{C-F}$ = 2.9 Hz), 128.4, 127.6, 127.6 (d, $^3J_{C-F}$ = 8.6 Hz), 127.0, 115.3 (d, $^2J_{C-F}$ = 21.4 Hz), 101.0, 47.8. ^{19}F NMR (376 MHz, d_6-DMSO): δ = -115.07. HRMS (ESI-[+H]) *m/z*: Calcd for $C_{16}H_{14}N_2FS$ 285.0862; Found 285.0879.

N-Benzyl-4-cyclobutylthiazol-2-amine (3z)

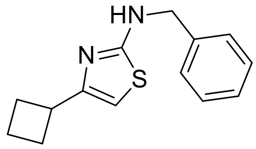

Preformed according to the general protocol with 1-cyclobutyl-2-(dimethyl(oxo)-λ^6-sulfanylidene)ethan-1-one (72 mg, 0.408 mmol) and *N*-benzyllthiourea (93 mg, 0.612 mmol). The product was purified by automated flash column chromatography (hexane/EtOAc, 100:0 – 50:50, 12 g SiO_2) to give *N*-benzyl-4-cyclobutylthiazol-2-amine (**3z**) as a white solid (55 mg, 55%). LCMS (UV, ESI) R_t = 8.00 min, $[M-H]^+$ *m/z* = 245.2, 99% purity. ^{1}H NMR (600 MHz, d_6-DMSO): δ = 7.97 (1H, d, J = 7.1 Hz), 7.37-7.29 (4H, m), 7.26-7.22 (1H, m), 6.19 (1H, s), 4.39 (2H, d, J = 5.9 Hz), 3.33-3.28 (1H, m), 2.19-2.05 (4H, m), 1.93-1.84 (1H, m), 1.82-1.75 (1H, m). ^{13}C NMR (151 MHz, d_6-DMSO): δ = 168.6, 139.2, 128.3, 127.6, 127.0, 109.6, 98.9, 47.9, 36.4, 28.0, 18.0. HRMS (ESI-[+H]) *m/z*: Calcd for $C_{14}H_{17}N_2S$ 245.1112; Found 245.1108.

N-Benzyl-4-methylthiazol-2-amine (3a')

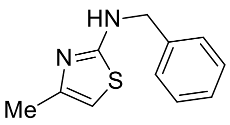

Preformed according to the general protocol with 1-(dimethyl-(oxo)-λ^6-sulfanylidene)propan-2-on (55 mg, 0.408 mmol) and *N*-benzyllthiourea (93 mg, 0.612 mmol). The product was purified by automated flash column chromatography (hexane/EtOAc, 100:0 – 50:50, 12 g SiO_2) to give *N*-benzyl-4-methylthiazol-2-amine (**3a'**) as a pale-yellow solid (51 mg, 61%). LCMS (UV, ESI) R_t = 6.60 min, [M-H]$^+$ *m/z* = 205.1, 97% purity. ^{1}H NMR (600 MHz, d_6-DMSO): δ = 7.94 (1H, t, *J* = 6.0 Hz), 7.34-7.29 (4H, m), 7.26-7.22 (1H, m), 6.14 (1H, d, *J* = 1.4 Hz), 4.40 (2H, d, *J* = 5.9 Hz), 2.07 (3H, d, *J* = 1.2 Hz). ^{13}C NMR (151 MHz, d_6-DMSO): δ = 168.3, 147.8, 139.4, 128.3, 127.4, 126.9, 100.2, 47.7, 17.4. HRMS (ESI-[+H]) *m/z*: Calcd for $C_{11}H_{13}N_2S$ 205.0799; Found 205.0811.

N-Benzyl-4-(*tert*-butyl)thiazol-2-amine (3b')

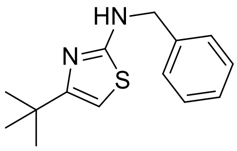

Preformed according to the general protocol with 1-(dimethyl(oxo)-λ^6-sulfanylidene)-3,3-dimethylbutan-2-one (72 mg, 0.408 mmol) and *N*-benzyllthiourea (93 mg, 0.612 mmol). The product was purified by automated flash column chromatography (hexane/EtOAc, 100:0 – 90:10, 12 g SiO_2) to give *N*-benzyl-4-(*tert*-butyl)thiazol-2-amine (**3b'**) as a yellow gum (55 mg, 55%). LCMS (UV, ESI) R_t = 9.10 min, [M-H]$^+$ *m/z* = 247.4, 92% purity. ^{1}H NMR (600 MHz, d_6-DMSO): δ = 7.39-7.27 (6H, m), 6.04 (1H, s), 4.41 (2H, s), 1.27 (9H, s). ^{13}C NMR (151 MHz, d_6-DMSO): δ = 137.8, 128.8, 128.6, 127.9, 127.8, 127.6, 98.3, 50.2, 34.7, 29.8. HRMS (ESI-[+H]) *m/z*: Calcd for $C_{14}H_{19}N_2S$ 247.1269; Found 247.1269.

4-(2-Methoxyphenyl)thiazol-2-amine (3c')

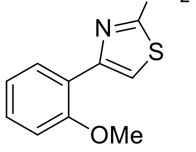

Preformed according to the general protocol with 2-methoxybenzoyl(dimethyloxosulphonio)methanide (92 mg, 0.408 mmol) and thiourea (46 mg, 0.612 mmol). The product was purified by automated flash

column chromatography (hexane/EtOAc, 100:0 – 60:40, 12 g SiO_2) to give 4-(2-methoxyphenyl)thiazol-2-amine (**3c'**) as a yellow gum (60 mg, 71%). LCMS (UV, ESI) R_t = 6.87 min, $[M\text{-}H]^+$ *m/z* = 207.2, 99% purity. ^{1}H NMR (600 MHz, d_6-DMSO): δ = 8.01 (1H, dd, *J* = 7.8, 1.8 Hz), 7.23 (1H, ddd, *J* = 8.2, 7.2, 1.8 Hz), 7.11 (1H, s), 7.06 (1H, dd, *J* = 8.2, 1.1 Hz), 6.99-6.93 (3H, m), 3.88 (3H, s). ^{13}C NMR (151 MHz, d_6-DMSO): δ = 166.3, 156.5, 145.7, 129.3, 128.1, 123.1, 120.3, 111.4, 105.7, 55.4. HRMS (ESI-[+H]) *m/z*: Calcd for $C_{10}H_{11}N_2OS$ 207.0592; Found 207.0596.

4-(4-Methoxyphenyl)thiazol-2-amine (3d')

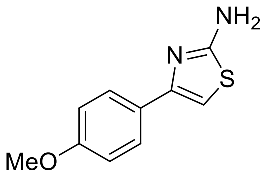

Preformed according to the general protocol with 4-methoxybenzoyl(dimethyloxosulphonio)methanide (92 mg, 0.408 mmol) and thiourea (46 mg, 0.612 mmol). The product was purified by automated flash column chromatography (hexane/EtOAc, 100:0 – 60:40, 12 g SiO_2) to give 4-(4-methoxyphenyl)thiazol-2-amine (**3d'**) as a pale yellow solid (48 mg, 57%). LCMS (UV, ESI) R_t = 6.73 min, $[M\text{-}H]^+$ *m/z* = 207.2, 98% purity. ^{1}H NMR (600 MHz, d_6-DMSO): δ = 7.74-7.69 (2H, m), 7.02 (2H, s), 6.94-6.89 (2H, m), 6.82 (1H, s), 3.76 (3H, s). ^{13}C NMR (151 MHz, d_6-DMSO): δ = 168.1, 158.5, 149.7, 127.9, 126.9, 113.8, 99.3, 55.1. HRMS (ESI-[+H]) *m/z*: Calcd for $C_{10}H_{11}N_2OS$ 207.0592; Found 207.0611.

4-(Thiophen-2-yl)thiazol-2-amine (3e')

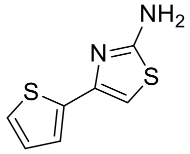

Preformed according to the general protocol with 2-thiophenecarbonyl(dimethyloxosulphonio) methanide (83 mg, 0.408 mmol) and thiourea (46 mg, 0.612 mmol). The product was purified by automated flash column chromatography (hexane/EtOAc, 100:0 – 60:40, 12 g SiO_2) to give 4-(thiophen-2-yl)thiazol-2-amine (**3e'**) as a pale orange solid (23 mg, 52%). LCMS (UV, ESI) R_t = 6.73 min, $[M\text{-}H]^+$ *m/z* = 183.0, 95% purity. ^{1}H NMR (600 MHz, d_6-DMSO): δ = 7.39 (1H, dd, *J* = 5.0, 1.2 Hz), 7.37 (1H, dd, *J* = 3.6, 1.1 Hz), 7.15 (2H, s), 7.03 (1H, dd, *J* = 5.0, 3.6 Hz), 6.84 (1H, s). ^{13}C NMR

(151 MHz, d_6-DMSO): δ = 168.3, 144.5, 139.2, 127.8, 124.7, 122.7, 99.8. HRMS (ESI-[+H]) *m/z*: Calcd for $C_7H_7N_2S_2$ 183.0051; Found 183.0054.

4-(4-Bromophenyl)thiazol-2-amine (3f')

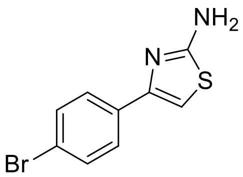

Preformed according to the general protocol with 4-bromobenzoyl(dimethyloxosulphonio)methanide (112 mg, 0.408 mmol) and thiourea (46 mg, 0.612 mmol). The product was purified by automated flash column chromatography (hexane/EtOAc, 100:0 – 60:40, 12 g SiO_2) to give 4-(4-bromophenyl)thiazol-2-amine (**3f'**) as a pale yellow solid (48 mg, 46%). LCMS (UV, ESI) R_t = 11.73 min, $[M\text{-}H]^+$ *m/z* = 256.7, 99% purity. ^{1}H NMR (600 MHz, d_6-DMSO): δ = 7.76-7.72 (2H, m), 7.57-7.52 (2H, m), 7.11 (2H, s), 7.09 (1H, s). ^{13}C NMR (151 MHz, d_6-DMSO): δ = 168.4, 148.6, 134.1, 131.4, 127.6, 120.1, 102.4. HRMS (ESI-[+H]) *m/z*: Calcd for $C_9H_8BrN_2S$ 254.9592; Found 254.9600.

4-(4-Chlorophenyl)thiazol-2-amine (3g')

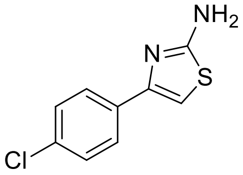

Preformed according to the general protocol with 4-chlorobenzoyl(dimethyloxosulphonio)methanide (94 mg, 0.408 mmol) and thiourea (46 mg, 0.612 mmol). The product was purified by automated flash column chromatography (hexane/EtOAc, 100:0 – 60:40, 12 g SiO_2) to give 4-(4-chlorophenyl)thiazol-2-amine (**3g'**) as a yellow solid (38 mg, 44%). LCMS (UV, ESI) R_t = 10.83 min, $[M\text{-}H]^+$ *m/z* = 211.0, 91% purity. ^{1}H NMR (600 MHz, d_6-DMSO): δ = 7.83-7.78 (2H, m), 7.44-7.39 (2H, m), 7.11 (2H, s), 7.08 (1H, s). ^{13}C NMR (151 MHz, d_6-DMSO): δ = 168.4, 148.6, 133.8, 131.5, 128.5, 127.2, 102.3. HRMS (ESI-[+H]) *m/z*: Calcd for $C_9H_8ClN_2S$ 211.0097; Found 211.0099.

4-(Pyridin-3-yl)thiazol-2-amine (3h')

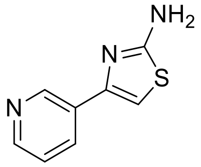

Preformed according to the general protocol with 2-(dimethyl(oxo)-λ^6-sulfaneylidene)-1-(pyridin-3-yl)ethan-1-oneone (80 mg, 0.408 mmol) and thiourea (46 mg, 0.612 mmol). The product was purified by automated flash column chromatography (hexane/EtOAc, 100:0 – 0:100, 12 g SiO_2) to give 4-(pyridin-3-yl)thiazol-2-amine (**3h'**) as a white solid (51 mg, 71%). LCMS (UV, ESI) R_t = 5.48 min, $[M\text{-}H]^+$ *m/z* = 178.0, 96% purity. ^1H NMR (600 MHz, d_6-DMSO): δ = 9.00 (1H, d, J = 2.2 Hz), 8.45 (1H, dd, J = 4.9, 1.6 Hz), 8.11 (1H, dt, J = 8.0, 2.0 Hz), 7.39 (1H, dd, J = 7.9, 4.7 Hz), 7.21-7.16 (3H, m). ^{13}C NMR (151 MHz, d_6-DMSO): δ = 183.8, 168.7, 148.1, 146.9, 132.6, 130.5, 123.7, 103.1. HRMS (ESI-[+H]) *m/z*: Calcd for $C_8H_8N_3S$ 178.0439; Found 178.0446.

***N*-Benzyl-4-(pyridin-3-yl)thiazol-2-amine (3i')**

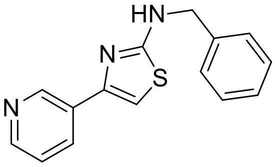

Preformed according to the general protocol with 2-(dimethyl(oxo)-λ^6-sulfaneylidene)-1-(pyridin-3-yl)ethan-1-oneone (80 mg, 0.408 mmol) and *N*-benzyllthiourea (93 mg, 0.612 mmol). The product was purified by automated flash column chromatography (hexane/EtOAc, 100:0 – 0:100, 12 g SiO_2) to give *N*-benzyl-4-(pyridin-3-yl)thiazol-2-amine (**3i'**) as an off-white solid (56 mg, 51%). LCMS (UV, ESI) R_t = 8.81 min, $[M\text{-}H]^+$ *m/z* = 268.3, 99% purity. ^1H NMR (600 MHz, $CDCl_3$): δ = 9.03 (1H, d, J = 2.4 Hz), 8.50 (1H, dd, J = 4.8, 1.6 Hz), 8.09 (1H, dt, J = 8.0, 2.0 Hz), 7.42-7.28 (6H, m), 6.79 (1H, s), 4.53 (2H, d, J = 4.9 Hz). ^{13}C NMR (151 MHz, $CDCl_3$): δ = 169.7, 148.5, 148.2, 147.5, 137.5, 133.6, 130.7, 128.9, 128.0, 127.9, 123.7, 102.6, 50.0. HRMS (ESI-[+H]) *m/z*: Calcd for $C_{15}H_{14}N_3S$ 268.0908; Found 268.0905.

Insertion/Cyclisation of Selenoureas

4-(3-Methoxyphenyl)-1,3-selenazol-2-amine (7a)

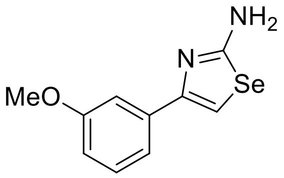

Preformed according to the general protocol with 3-methoxybenzoyl(dimethyloxosulphonio)methanide (92 mg, 0.408 mmol) and selenourea (75 mg, 0.612 mmol). The product was purified by automated flash column chromatography (hexane/EtOAc, 100:0 – 60:40, 12 g SiO_2) to give 4-(3-methoxyphenyl)-1,3-selenazol-2-amine (**7a**) as a light brown solid (50 mg, 48%). LCMS (UV, ESI) R_t = 12.27 min, $[M-H]^+$ *m/z* = 255.0, 99% purity. ^1H NMR (600 MHz, d_6-DMSO): δ = 7.48 (1H, s), 7.39-7.33 (2H, m), 7.32 (2H, s), 7.26-7.22 (1H, m), 6.81 (1H, ddd, J = 8.1, 2.6, 1.0 Hz), 3.76 (3H, s). ^{13}C NMR (151 MHz, d_6-DMSO): δ = 169.2, 159.4, 150.5, 137.2, 129.5, 118.3, 112.7, 111.2, 106.4, 55.0. HRMS (ESI-[+H]) *m/z*: Calcd for $C_{10}H_{11}N_2O^{80}Se$ 255.0037; Found 255.0049.

4-(4-Methoxyphenyl)-1,3-selenazol-2-amine (7b)

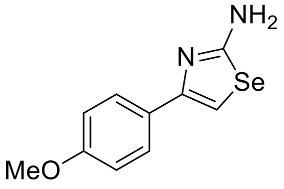

Preformed according to the general protocol with 4-methoxybenzoyl(dimethyloxosulphonio)methanide (92 mg, 0.408 mmol) and selenourea (75 mg, 0.612 mmol). The product was purified by automated flash column chromatography (hexane/EtOAc, 100:0 – 60:40, 12 g SiO_2) to give 4-(4-methoxyphenyl)-1,3-selenazol-2-amine (**7b**) as a yellow solid (41 mg, 40%). LCMS (UV, ESI) R_t = 11.51 min, $[M-H]^+$ *m/z* = 255.0, 94% purity. ^1H NMR (600 MHz, d_6-DMSO): δ = 7.74-7.69 (2H, m), 7.29-7.26 (3H, m), 6.92-6.86 (2H, m), 3.76 (3H, s). ^{13}C NMR (151 MHz, d_6-DMSO): δ = 169.1, 158.3, 150.4, 128.7, 127.1, 113.8, 103.6, 55.1. HRMS (ESI-[+H]) *m/z*: Calcd for $C_{10}H_{11}N_2O^{80}Se$ 255.0037; Found 255.0049.

4-(Thiophen-2-yl)-1,3-selenazol-2-amine (7c)

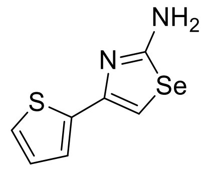

Preformed according to the general protocol with 2-thiophenecarbonyl(dimethyloxosulphonio)methanide (83 mg, 0.408 mmol) and selenourea (75 mg, 0.612 mmol). The product was purified by automated flash column chromatography (hexane/EtOAc, 100:0 – 60:40, 12 g SiO_2) to give 4-(yhiophen-2-yl)-1,3-selenazol-2-amine (**7c**) as a light-yellow solid (38 mg, 42%). LCMS (UV, ESI) R_t = 22.78 min, $[M-H]^+$ *m/z* = 253.0, 97% purity. ^1H NMR (600 MHz, d_6-DMSO): δ = 7.41 (2H, s), 7.37 (1H, dd, J = 5.1, 1.2 Hz), 7.34 (1H, dd, J = 3.7, 1.2 Hz), 7.29 (1H, s), 7.01 (1H, dd, J = 5.0, 3.6 Hz). ^{13}C NMR (151 MHz, d_6-DMSO): δ = 169.5, 145.2, 140.2, 127.7, 124.6, 122.5, 103.9. HRMS (ESI-[+H]) *m/z*: Calcd for $C_7H_7N_2S^{80}Se$ 230.9495; Found 230.9427.

4-(*p*-Tolyl)-1,3-selenazol-2-amine (7d)

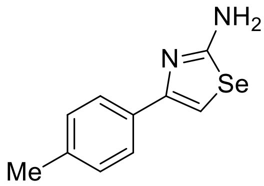

Preformed according to the general protocol with 4-methylbenzoyl (dimethyloxosulphonio)methanide (86 mg, 0.408 mmol) and selenourea (75 mg, 0.612 mmol). The product was purified by automated flash column chromatography (hexane/EtOAc, 100:0 – 60:40, 12 g SiO_2) to give 4-(*p*-tolyl)-1,3-selenazol-2-amine (**7d**) as a pale yellow solid (44 mg, 45%). LCMS (UV, ESI) R_t = 12.60 min, $[M-H]^+$ *m/z* = 239.0, 97% purity. ^1H NMR (600 MHz, d_6-DMSO): δ = 7.68 (2H, d, J = 7.9 Hz), 7.37 (1H, s), 7.29 (2H, s), 7.14 (2H, d, J = 7.9 Hz), 2.28 (3H, s). ^{13}C NMR (151 MHz, d_6-DMSO): δ = 169.2, 150.7, 136.1, 133.1, 129.0, 125.8, 104.9, 20.8. HRMS (ESI-[+H]) *m/z*: Calcd for $C_{10}H_{11}N_2{}^{80}Se$ 239.0087; Found 239.0079.

4-(4-Chlorophenyl)-1,3-selenazol-2-amine (7e)

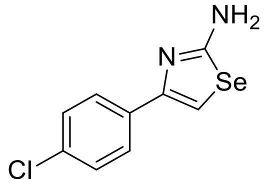

Preformed according to the general protocol with 4-chlorobenzoyl (dimethyloxosulphonio)methanide (94 mg, 0.408 mmol) and selenourea (75 mg, 0.612 mmol). The product was purified by automated flash column chromatography (hexane/EtOAc, 100:0 – 60:40, 12 g SiO_2) to give 4-(4-chlorophenyl)-1,3-selenazol-2-amine (**7e**) as a yellow solid (36 mg, 34%). LCMS (UV, ESI) R_t = 9.64 min, $[M-H]^+$ *m/z* = 258.8, 96% purity. ^{1}H NMR (600 MHz, d_6-DMSO): δ = 7.83-7.78 (2H, m), 7.53 (1H, s), 7.42-7.37 (2H, m), 7.36 (2H, s). ^{13}C NMR (151 MHz, d_6-DMSO): δ = 169.5, 149.4, 134.6, 131.2, 128.4, 127.6, 106.8. HRMS (ESI-[+H]) *m/z*: Calcd for $C_9H_8N_2Cl^{80}Se$ 258.9541; Found 258.9535.

4-(4-Bromophenyl)-1,3-selenazol-2-amine (7f)

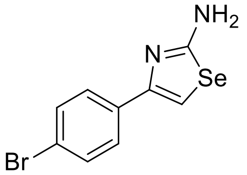

Preformed according to the general protocol with 4-bromobenzoyl (dimethyloxosulphonio)methanide (112 mg, 0.408 mmol) and selenourea (75 mg, 0.612 mmol). The product was purified by automated flash column chromatography (hexane/EtOAc, 100:0 – 70:30, 24 g SiO_2) to give 4-(4-chlorophenyl)-1,3-selenazol-2-amine (**7f**) as a pale-yellow solid (51 mg, 41%). LCMS (UV, ESI) R_t = 9.66 min, $[M-H]^+$ *m/z* = 302.7, 99% purity. ^{1}H NMR (600 MHz, d_6-DMSO): δ = 7.77-7.71 (2H, m), 7.56-7.49 (3H, m), 7.36 (2H, s). ^{13}C NMR (151 MHz, d_6-DMSO): δ = 169.5, 149.5, 134.9, 131.4, 127.9, 119.8, 106.9. HRMS (ESI-[+H]) *m/z*: Calcd for $C_9H_8N_2Br^{80}Se$ 302.9036; Found 302.9051.

4-(4-Nitrophenyl)-1,3-selenazol-2-amine (7g)

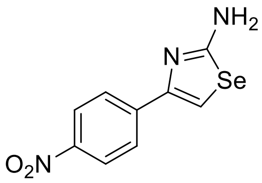

Preformed according to the general protocol with 4-nitrobenzoyl (dimethyloxosulphonio)methanide (98 mg, 0.408 mmol) and selenourea (75 mg, 0.612 mmol). The product was purified by automated flash column chromatography (hexane/EtOAc, 100:0 – 70:30, 24 g SiO_2) to give 4-(4-nitrophenyl)-1,3-selenazol-2-amine (**7g**) as an orange solid (33 mg, 30%). LCMS (UV, ESI) R_t = 10.26 min, $[M-H]^+$ *m/z* = 269.9, 99% purity. ^{1}H NMR (600 MHz, d_6-DMSO): δ = 8.24-8.18 (2H, m), 8.07-8.02 (2H, m), 7.89 (1H, s), 7.48 (2H, s). ^{13}C NMR (151 MHz, d_6-DMSO): δ = 169.8, 148.8, 145.7, 141.7, 126.6, 124.0, 111.6. HRMS (ESI-[+H]) *m/z*: Calcd for $C_9H_8N_3O_2{}^{80}Se$ 269.9782; Found 269.9795.

Synthesis of Prospective peptidyl-tRNA hydrolase (Pth) Inhibitors

Additional Solvent Screen for the Iridium-mediated cyclisation

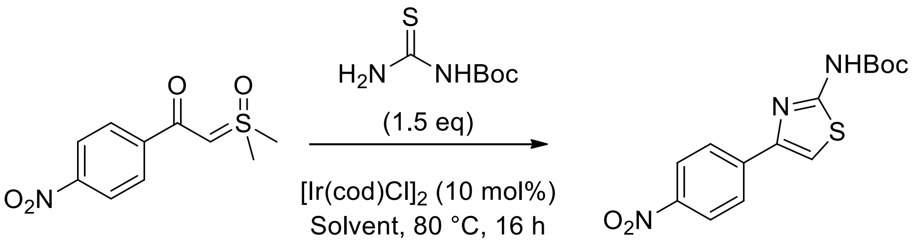

Scheme S3. Solvent Screen for the NO_2 Ylide Iridium Cyclisation.

General protocol:

A mixture of *N*-Boc-thiourea (54 mg, 0.306 mmol), 4-nitrobenzoyl(dimethyloxosulphonio)methanide (49 mg, 0.204 mmol), and solvent (1 mL) was heated to 80 °C. To the mixture was added $[Ir(cod)Cl]_2$ (13.7 mg, 0.0204 mmol) and the reaction stirred at 80 °C for 24 h. The reaction was cooled to ambient temperature, concentrated under reduced pressure, and to the mixture was added a known amount of durene as an external standard. The % yield/conversion was calculated by the integration of the durene and product signals in the proton NMR.

Solvent	NMR Yield (%)
**Toluene**	46
**MeOH**	45
**DMA**	55
**DCM**[a]	5
**DCE**	66
**MeCN**	57

[a]Conducted in a sealed tube at 80 °C.

Table S3. Results of the Solvent Screen for the Iridium Cyclisation.

***tert*-Butyl (4-(4-nitrophenyl)thiazol-2-yl)carbamate (3j')**

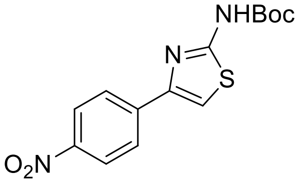

A mixture of 4-nitrobenzoyl(dimethyloxosulphonio)methanide (492 mg, 2.04 mmol), *N*-Boc-thiourea (538 mg, 3.06 mmol), and DCE (10 mL) was heated to 80 °C and to the mixture was added $[Ir(cod)Cl]_2$ (136 mg, 0.204 mmol). The resulting mixture was stirred for 24 h at 80 °C. The reaction was cooled to ambient temperature and concentrated under reduced pressure. The resulting residue was purified by was purified by automated reverse phase chromatography (H_2O (1% formic acid)/Acetonitrile (1% formic acid), 95:5 – 5:95, 36 g C28) to give *tert*-butyl (4-(4-nitrophenyl)thiazol-2-yl)carbamate (**3j'**) as an orange solid (470 mg, 72%). LCMS (UV, ESI) R_t = 22.47 min, $[M-H]^+$ *m/z* = did not ionise, 97% purity. ^1H NMR (600 MHz, d_6-DMSO): δ = 11.77 (1H, s), 8.30 (2H, d, *J* = 9.4 Hz), 8.13 (2H, d, *J* = 8.8 Hz), 7.96 (1H, s), 1.50 (9H, s). ^{13}C NMR (151 MHz, d_6-DMSO): δ = 161.8, 160.3, 147.0, 146.4, 140.3, 126.5, 124.2, 112.1, 81.5, 27.9. HRMS (ESI-[+H]) *m/z*: Calcd for $C_{14}H_{16}N_3O_4S$ 322.0862; Found 322.0872.

tert-Butyl (4-(4-aminophenyl)thiazol-2-yl)carbamate (3k')

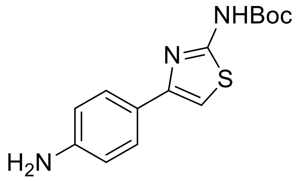

Palladium on carbon (10%) (91 mg, 0.087 mmol), MeOH (8 mL), and *tert*-butyl (4-(4-nitrophenyl)thiazol-2-yl)carbamate (278 mg, 0.865 mmol) were added to one chamber of the 400 cm^3 COware apparatus under a flow of argon. The first reaction chamber was sealed and to the second was added aqueous 7M HCl (14 mL), followed by zinc (3.14 g), while retaining a flow of argon. The second tube was sealed, and the reaction stirred for 16 h at ambient temperature. To the mixture was added Celite®, and the solution stirred for 15 minutes. The resulting mixture was filtered through Celite® and concentrated under reduced pressure. The resulting residue was purified by automated flash column chromatography (hexane/EtOAc, 100:0 – 0:100, 24 g SiO_2) to give *tert*-butyl (4-(4-aminophenyl)thiazol-2-yl)carbamate (**3k'**) as a white solid (220 mg, 87%). LCMS (UV, ESI) R_t = 18.08 min, $[M-H]^+$ *m/z* = 292.0, 99% purity. ^1H NMR (600 MHz, d_6-DMSO): δ = 11.46 (1H, s), 7.52 (2H, d, *J* = 8.2 Hz), 7.13 (1H, s), 6.56 (2H, d, *J* = 8.2 Hz), 5.24 (2H, s), 1.48 (9H, s). ^{13}C NMR (151 MHz, d_6-

DMSO): δ = 159.5, 158.8, 150.6, 149.0, 127.1, 122.9, 114.1, 103.1, 80.2, 28.4. HRMS (ESI-[+H]) *m/z*: Calcd for $C_{14}H_{18}N_3O_2S$ 292.1197; Found 292.0920.

4-(4-Aminophenyl)thiazol-2-amine dihydrochloride (3l')

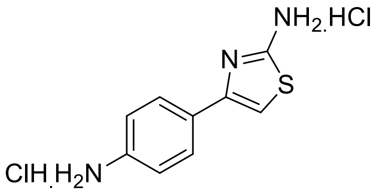

To a mixture of 1,4-dioxane (2 mL) and *tert*-butyl (4-(4-aminophenyl)thiazol-2-yl)carbamate (25 mg, 0.086 mmol) was added HCl (4M in 1,4-dioxane) (1 mL) and the resulting mixture stirred for 16 h at ambient temperature. The resulting mixture was concentrated under reduced pressure to give 4-(4-aminophenyl)thiazol-2-amine dihydrochloride (**3l'**) as a yellow solid (23 mg, 100%). LCMS (UV, ESI) R_t = 5.74 min, $[M-H]^+$ *m/z* = 192.0, 99% purity. ^{1}H NMR (600 MHz, d_6-DMSO): δ = 8.76 (2H, s, br), 7.75 (2H, d, J = 8.1 Hz), 7.21 (2H, d, J = 8.1 Hz), 7.13 (1H, s). ^{13}C NMR (151 MHz, d_6-DMSO): δ = 170.1, 127.0, 120.7, 101.4 (one carbon not observed). HRMS (ESI-[+H]) *m/z*: Calcd for $C_9H_{10}N_3S$ 192.0595; Found 192.0612.

tert-Butyl (4-(4-acetamidophenyl)thiazol-2-yl)carbamate (3m')

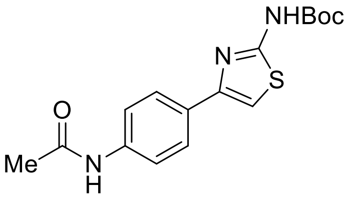

To a mixture of *tert*-butyl (4-(4-aminophenyl)thiazol-2-yl)carbamate (43 mg, 0.148 mmol), triethylamine (41 µL, 0.296 mmol), and anhydrous DCM (1.5 mL) was added acetyl chloride (13 µL, 0.178 mmol) and the resulting mixture stirred for 16 h at ambient temperature under an argon atmosphere. To the mixture was added saturated aqueous $NaHCO_3$ (10 mL) and DCM (10 mL). The resulting biphasic mixture was separated, and the aqueous phase extracted with DCM (3 x 10 mL). The combined organic extracts were washed with brine (10 mL), dried over anhydrous MgSO4, filtered, and concentrated under reduced pressure to give *tert*-butyl (4-(4-acetamidophenyl)thiazol-2-yl)carbamate (**3m'**) as a white gum of sufficient purity by NMR to be used in the subsequent reaction without further purification.

N-(4-(2-Aminothiazol-4-yl)phenyl)acetamide (3s')

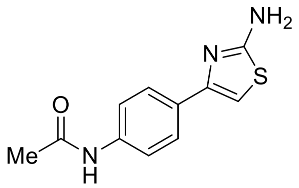

To *tert*-butyl (4-(4-acetamidophenyl)thiazol-2-yl)carbamate (49 mg, 0.148 mmol) was added HCl (4M in 1,4-dioxane) (2 mL) and the resulting mixture stirred for 72 h at ambient temperature. The resulting mixture was concentrated under reduced pressure and to the residue was added DCM (5 mL) and saturated aqueous Na_2CO_3 (5 mL). The resulting biphasic mixture was separated, and the aqueous phase extracted with DCM (3 x 5 mL). The combined organic extracts were dried over anhydrous $MgSO_4$, filtered, and concentrated under reduced pressure. The resulting residue was purified by automated flash column chromatography (hexane/EtOAc, 100:0 – 90:10, 12 g SiO_2) to give *N*-(4-(2-aminothiazol-4-yl)phenyl)acetamide (**3s'**) as a pale-yellow solid (30 mg, 87%). LCMS (UV, ESI) R_t = 7.02 min, $[M-H]^+$ *m/z* = 234.0, 99% purity. ^{1}H NMR (600 MHz, d_6-DMSO): δ = 9.97 (1H, s), 7.70 (2H, d, J = 8.80 Hz), 7.56 (2H, d, J = 8.8 Hz), 7.03 (2H, s), 6.87 (1H, s), 3.35 (3H, s). ^{13}C NMR (151 MHz, d_6-DMSO): δ = 168.2, 168.1, 149.7, 138.4, 130.0, 125.9, 118.8, 100.1, 24.1. HRMS (ESI-[+H]) *m/z*: Calcd for $C_{11}H_{12}N_3OS$ 234.0701; Found 234.0695.

tert-Butyl (4-(4-propionamidophenyl)thiazol-2-yl)carbamate (3n')

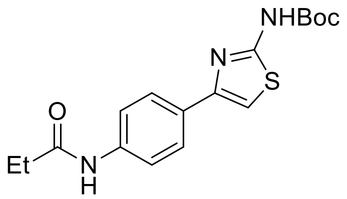

To a mixture of *tert*-butyl (4-(4-aminophenyl)thiazol-2-yl)carbamate (39 mg, 0.134 mmol), triethylamine (37 µL, 0.268 mmol), and anhydrous DCM (1.5 mL) was added propionyl chloride (14 µL, 0.161 mmol) and the resulting mixture stirred for 16 h at ambient temperature under an argon atmosphere. The mixture was concentrated under reduced pressure. The resulting residue was purified by automated flash column chromatography (hexane/EtOAc, 100:0 – 0:100, 12 g SiO_2) to give *tert*-butyl (4-(4-propionamidophenyl)thiazol-2-yl)carbamate (**3n'**) as an off-white solid (43 mg, 92%). LCMS (UV, ESI) R_t = 18.82 min, $[M-H]^+$ *m/z* = 348.0, 96% purity. ^{1}H NMR (600 MHz, d_6-DMSO): δ = 11.57 (1H, s), 9.95 (1H, s), 7.80-7.75 (2H, m), 7.66-7.60 (2H, m), 7.43 (1H, s), 2.32 (2H, q, J = 7.6 Hz), 1.49 (9H, s), 1.08 (3H, t, J = 7.6 Hz). ^{13}C NMR (151 MHz, d_6-DMSO): δ = 172.1, 159.6, 149.0,

138.9, 129.2, 126.1, 119.0, 106.1, 81.2, 48.7, 29.6, 28.0, 9.7. HRMS (ESI-[+H]) *m/z*: Calcd for $C_{17}H_{22}N_3O_3S$ 348.1382; Found 348.1380.

***tert*-Butyl (4-(4-(cyclopropanecarboxamido)phenyl)thiazol-2-yl)carbamate (3o')**

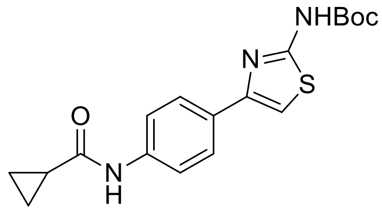

To a mixture of *tert*-butyl (4-(4-aminophenyl)thiazol-2-yl)carbamate (39 mg, 0.134 mmol), triethylamine (37 µL, 0.268 mmol), and anhydrous DCM (1.5 mL) was added cyclopropanecarbonyl chloride (15 µL, 0.161 mmol) and the resulting mixture stirred for 16 h at ambient temperature under an argon atmosphere. The mixture was concentrated under reduced pressure. The resulting residue was purified by automated flash column chromatography (hexane/EtOAc, 100:0 – 0:100, 12 g SiO_2) to give *tert*-butyl (4-(4-(cyclopropanecarboxamido)phenyl)thiazol-2-yl)carbamate (**3o'**) as a white solid (40 mg, 83%). LCMS (UV, ESI) R_t = 19.48 min, $[M-H]^+$ *m/z* = 360.0, 95% purity. ^{1}H NMR (600 MHz, d_6-DMSO): δ = 11.54 (1H, s), 10.29 (1H, s), 7.80-7.75 (2H, m), 7.64-7.59 (2H, m), 7.42 (1H, s), 1.81-1.74 (1H, m), 1.48 (9H, s), 0.83-0.75 (4H, m). ^{13}C NMR (151 MHz, d_6-DMSO): δ = 171.9, 159.7, 149.1, 139.0, 129.3, 126.2, 119.1, 106.2, 81.3, 48.8, 28.0, 14.7, 7.4. HRMS (ESI-[+H]) *m/z*: Calcd for $C_{18}H_{22}N_3O_3S$ 360.1382; Found 360.1396.

***tert*-Butyl (4-(4-(cyclobutanecarboxamido)phenyl)thiazol-2-yl)carbamate (3p')**

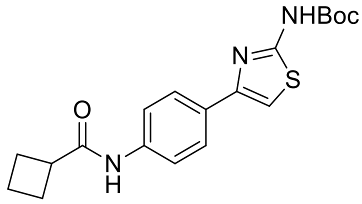

To a mixture of *tert*-butyl (4-(4-aminophenyl)thiazol-2-yl)carbamate (39 mg, 0.134 mmol), triethylamine (37 µL, 0.268 mmol), and anhydrous DCM (1.5 mL) was added cyclobutanecarbonyl chloride (18 µL, 0.161 mmol) and the resulting mixture stirred for 16 h at ambient temperature under an argon atmosphere. The mixture was concentrated under reduced pressure. The resulting residue was purified by automated flash column chromatography (hexane/EtOAc, 100:0 – 0:100, 12 g SiO_2) to give *tert*-butyl (4-(4-(cyclobutanecarboxamido)phenyl)thiazol-2-yl)carbamate (**3p'**) as a white solid (37 mg,

74%). LCMS (UV, ESI) R_t = 20.81 min, $[M-H]^+$ m/z = 374.0, 93% purity. ^{1}H NMR (600 MHz, d_6-DMSO): δ = 11.57 (1H, s), 9.81 (1H, s), 7.79-7.76 (2H, m), 7.66-7.63 (2H, m), 7.43 (1H, s), 3.26-3.19 (1H, m), 2.27-2.17 (2H, m), 2.14-2.06 (2H, m), 1.99-1.91 (1H, m), 1.84-1.77 (1H, m), 1.48 (s, 9H). ^{13}C NMR (151 MHz, d_6-DMSO): δ = 172.9, 159.6, 149.0, 138.9, 129.2, 126.1, 119.1, 106.1, 56.7, 27.9, 27.2, 24.6, 17.8 (one carbon not observed). HRMS (ESI-[+H]) m/z: Calcd for $C_{19}H_{24}N_3O_3S$ 374.1538; Found 374.1533.

tert-Butyl (4-(4-isobutyramidophenyl)thiazol-2-yl)carbamate (3q')

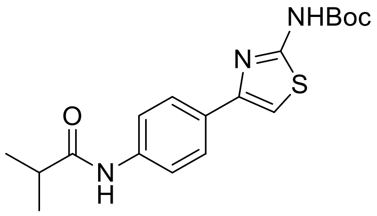

To a mixture of *tert*-butyl (4-(4-aminophenyl)thiazol-2-yl)carbamate (39 mg, 0.134 mmol), triethylamine (37 µL, 0.268 mmol), and anhydrous DCM (1.5 mL) was added isobutyryl chloride (17 µL, 0.161 mmol) and the resulting mixture stirred for 16 h at ambient temperature under an argon atmosphere. The mixture was concentrated under reduced pressure. The resulting residue was purified by automated flash column chromatography (hexane/EtOAc, 100:0 – 0:100, 12 g SiO_2) to give *tert*-butyl (4-(4-isobutyramidophenyl)thiazol-2-yl)carbamate (**3q'**) as a white solid (33 mg, 68%). LCMS (UV, ESI) R_t = 20.18 min, $[M-H]^+$ m/z = 362.0, 94% purity. ^{1}H NMR (600 MHz, d_6-DMSO): δ = 11.57 (1H, s), 9.91 (1H, s), 7.80-7.75 (2H, m), 7.67-7.62 (2H, m), 7.44 (1H, s), 2.59 (1H, p, J = 6.8 Hz), 1.49 (9H, s), 1.10 (6H, d, J = 6.8 Hz). ^{13}C NMR (151 MHz, d_6-DMSO): δ = 175.2, 159.4, 152.9, 149.0, 139.0, 129.2, 126.0, 119.1, 106.1, 80.6, 35.0, 27.9, 19.6. HRMS (ESI-[+H]) m/z: Calcd for $C_{18}H_{24}N_3O_3S$ 362.1538; Found 362.1518.

tert-Butyl (4-(4-benzamidophenyl)thiazol-2-yl)carbamate (3r')

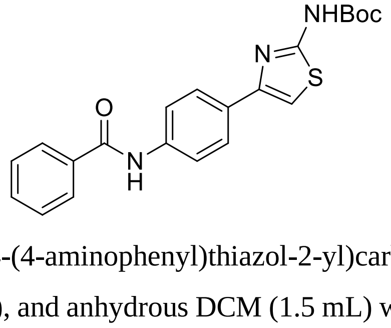

To a mixture of *tert*-butyl (4-(4-aminophenyl)thiazol-2-yl)carbamate (39 mg, 0.134 mmol), triethylamine (37 µL, 0.268 mmol), and anhydrous DCM (1.5 mL) was added benzoyl chloride (19 µL,

0.161 mmol) and the resulting mixture stirred for 16 h at ambient temperature under an argon atmosphere. The mixture was concentrated under reduced pressure. The resulting residue was purified by automated flash column chromatography (hexane/EtOAc, 100:0 – 50:50, 12 g SiO_2) to give *tert*-butyl (4-(4-benzamidophenyl)thiazol-2-yl)carbamate (**3r'**) as a white solid (37 mg, 70%). LCMS (UV, ESI) R_t = 21.48 min, $[M-H]^+$ *m/z* = 396.0, 99% purity. ^{1}H NMR (600 MHz, d_6-DMSO): δ = 11.60 (1H, s), 10.34 (1H, s), 7.99-7.93 (2H, m), 7.88-7.81 (4H, m), 7.62-7.58 (1H, m), 7.58-7.51 (2H, m), 7.49 (1H, s), 1.50 (9H, s). ^{13}C NMR (151 MHz, d_6-DMSO): δ = 165.6, 159.6, 153.0, 149.0, 138.7, 135.0, 131.6, 129.9, 128.5, 127.7, 126.0, 120.3, 106.4, 81.2, 28.0. HRMS (ESI-[+H]) *m/z*: Calcd for $C_{21}H_{22}N_3O_3S$ 396.1382; Found 396.1390.

N-(4-(2-Aminothiazol-4-yl)phenyl)propionamide (3t')

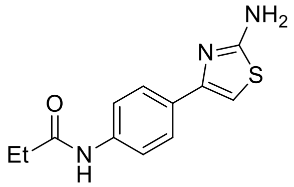

To a mixture of *tert*-butyl (4-(4-propionamidophenyl)thiazol-2-yl)carbamate (20 mg, 0.058 mmol) and DCM (2 mL) was added dropwise TFA (2 mL) and the resulting mixture stirred for 16 h at ambient temperature. The resulting mixture was concentrated under reduced pressure to give *N*-(4-(2-aminothiazol-4-yl)phenyl)propionamide (**3t'**) as an off-white gum (14 mg, 98%). LCMS (UV, ESI) R_t = 6.64 min, $[M-H]^+$ *m/z* = 248.0, 99% purity. ^{1}H NMR (600 MHz, d_6-DMSO): δ = 9.99 (1H, s), 7.94 (2H, s, br), 7.68 (2H, d, J = 8.8 Hz), 7.63 (2H, d, J = 8.7 Hz), 6.98 (1H, s), 2.33 (2H, q, J = 7.6 Hz), 1.08 (3H, t, J = 7.6 Hz). ^{13}C NMR (151 MHz, d_6-DMSO): δ = 172.1, 169.2, 158.3, 139.4, 126.2, 118.9, 100.6, 29.6, 9.7 (one carbon not observed). HRMS (ESI-[+H]) *m/z*: Calcd for $C_{12}H_{15}N_3OS$ 248.0858; Found 248.0846.

N-(4-(2-Aminothiazol-4-yl)phenyl)cyclopropanecarboxamide (3u')

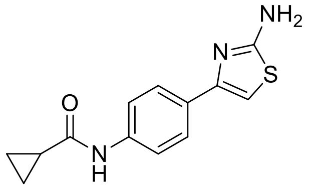

To a mixture of *tert*-butyl (4-(4-(cyclopropanecarboxamido)phenyl)thiazol-2-yl)carbamate (27 mg, 0.075 mmol) and DCM (2 mL) was added dropwise TFA (2 mL) and the resulting mixture stirred for 16 h at ambient temperature. The resulting mixture was concentrated under reduced pressure to give a

white solid (29 mg). The resulting residue was purified by automated reverse phase chromatography (H_2O (1% formic acid)/Acetonitrile (1% formic acid), 95:5 – 5:95, 12 g C28) to give *N*-(4-(2-aminothiazol-4-yl)phenyl)cyclopropanecarboxamide (**3u’**) as a white solid (14 mg, 72%). LCMS (UV, ESI) R_t = 6.94 min, $[M-H]^+$ *m/z* = 260.2, 98% purity. ^{1}H NMR (600 MHz, d_6-DMSO): δ = 10.23 (1H, s), 7.70 (2H, d, *J* = 8.4 Hz), 7.58 (2H, *d*, J = 8.4 Hz), 7.02 (2H, s), 6.87 (1H, s), 1.80-1.72 (1H, m), 0.82-0.75 (4H, m). ^{13}C NMR (151 MHz, d_6-DMSO): δ = 171.6, 168.1, 149.7, 138.5, 129.8, 126.0, 118.8, 100.12, 14.6, 7.2. HRMS (ESI-[+H]) *m/z*: Calcd for $C_{13}H_{15}N_3OS$ 260.0858; Found 260.0857.

N-(4-(2-Aminothiazol-4-yl)phenyl)cyclobutanecarboxamide (3v’)

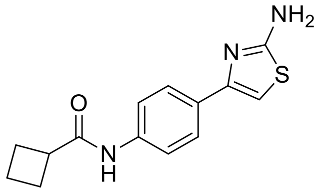

To a mixture of *tert*-butyl (4-(4-(cyclobutanecarboxamido)phenyl)thiazol-2-yl)carbamate (18 mg, 0.048 mmol) and DCM (2 mL) was added dropwise TFA (2 mL) and the resulting mixture stirred for 16 h at ambient temperature. The resulting mixture was concentrated under reduced pressure to give an off-white solid (18 mg). The resulting residue was purified by automated reverse phase chromatography (H_2O (1% formic acid)/Acetonitrile (1% formic acid), 95:5 – 5:95, 12 g C28) to give *N*-(4-(2-aminothiazol-4-yl)phenyl)cyclobutanecarboxamide (**3v’**) a white solid (12 mg, 91%). LCMS (UV, ESI) R_t = 7.19 min, $[M-H]^+$ *m/z* = 274.2, 99% purity. ^{1}H NMR (600 MHz, d_6-DMSO): δ = 9.77 (1H, s), 7.72-7.67 (2H, m), 7.64-7.54 (2H, m), 7.02 (2H, s), 6.88 (1H, s), 3.22 (1H, p, *J* = 8.4 Hz), 2.22 (2H, dq, *J* = 12.5, 9.6 Hz), 2.10 (2H, ddt, *J* = 12.0, 8.8, 4.3 Hz), 1.94 (1H, dq, *J* = 10.8, 8.8 Hz), 1.83-1.77 (1H, m). ^{13}C NMR (151 MHz, d_6-DMSO): δ = 172.8, 168.1, 149.7, 138.5, 129.9, 125.9, 119.0, 100.1, 24.6, 17.8 (one carbon not observed). HRMS (ESI-[+H]) *m/z*: Calcd for $C_{14}H_{17}N_3OS$ 274.1014; Found 274.1011.

N-(4-(2-Aminothiazol-4-yl)phenyl)isobutyramide (3w’)

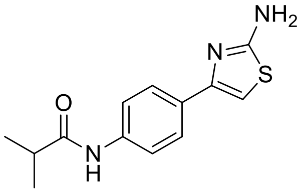

To a mixture of *tert*-butyl (4-(4-isobutyramidophenyl)thiazol-2-yl)carbamate (22 mg, 0.061 mmol) and DCM (2 mL) was added dropwise TFA (2 mL) and the resulting mixture stirred for 16 h at ambient

temperature. The resulting mixture was concentrated under reduced pressure to give a brown solid (24 mg). The resulting residue was purified by automated reverse phase chromatography (H_2O (1% formic acid)/Acetonitrile (1% formic acid), 95:5 – 5:95, 12 g C28) to give *N*-(4-(2-aminothiazol-4-yl)phenyl)isobutyramide (**3w'**) as an off-white solid (10 mg, 63%). LCMS (UV, ESI) R_t = 6.64 min, $[M\text{-}H]^+$ *m/z* = 262.2, 99% purity. ^1H NMR (600 MHz, d_6-DMSO): δ = 9.87 (1H, s), 7.73-7.68 (2H, m), 7.65-7.57 (2H, m), 7.03 (2H, s), 6.88 (1H, s), 2.59 (1H, *p*, J = 6.8 Hz), 1.09 (6H, *d*, J = 6.8 Hz). ^{13}C NMR (151 MHz, d_6-DMSO): δ = 175.2, 168.1, 149.7, 138.5, 129.9, 125.9, 119.0, 100.1, 35.0, 19.6. HRMS (ESI-[+H]) *m/z*: Calcd for $C_{13}H_{17}N_3OS$ 262.1014; Found 262.1034.

N-(4-(2-Aminothiazol-4-yl)phenyl)benzamide (3x')

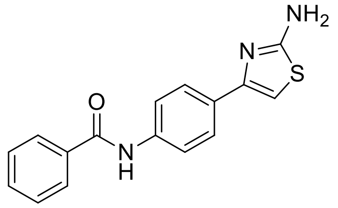

To a mixture of *tert*-butyl (4-(4-benzamidophenyl)thiazol-2-yl)carbamate (35 mg, 0.089 mmol) and DCM (2 mL) was added dropwise TFA (2 mL) and the resulting mixture stirred for 16 h at ambient temperature. The resulting mixture was concentrated under reduced pressure to give an off-white solid (35 mg). The resulting residue was purified by automated reverse phase chromatography (H_2O (1% formic acid)/Acetonitrile (1% formic acid), 95:5 – 5:95, 12 g C28) to give *N*-(4-(2-aminothiazol-4-yl)phenyl)benzamide (**3x'**) as a white solid (16 mg, 61%). LCMS (UV, ESI) R_t = 8.00 min, $[M\text{-}H]^+$ *m/z* = 296.0, 96% purity. ^1H NMR (600 MHz, d_6-DMSO): δ = 10.30 (1H, s), 7.97-7.94 (2H, m), 7.81-7.75 (4H, m), 7.62-7.58 (1H, m), 7.56-7.51 (2H, m), 7.05 (2H, s), 6.94 (1H, s). ^{13}C NMR (151 MHz, d_6-DMSO): δ = 168.2, 165.5, 149.7, 138.3, 135.0, 131.6, 130.5, 128.4, 127.7, 125.8, 120.2, 100.5. HRMS (ESI-[+H]) *m/z*: Calcd for $C_{16}H_{15}N_3OS$ 296.0858; Found 296.0870.

References

(1) Barday, M.; Janot, C.; Halcovitch, N. R.; Muir, J.; Aïssa, C. Cross-Coupling of α-Carbonyl Sulfoxonium Ylides with C−H Bonds. *Angewandte Chemie International Edition* **2017**, *56* (42), 13117–13121. https://doi.org/10.1002/anie.201706804.

(2) Zhu, S.; Shi, K.; Zhu, H.; Jia, Z.-K.; Xia, X.-F.; Wang, D.; Zou, L.-H. Copper-Catalyzed Annulation or Homocoupling of Sulfoxonium Ylides: Synthesis of 2,3-Diaroylquinolines or α,α,β-Tricarbonyl Sulfoxonium Ylides. *Organic Letters* **2020**, *22* (4), 1504–1509. https://doi.org/10.1021/acs.orglett.0c00085.

(3) Day, D. P.; Mora Vargas, J. A.; Burtoloso, A. C. B. Direct Synthesis of α-Fluoro-α-Triazol-1-Yl Ketones from Sulfoxonium Ylides: A One-Pot Approach. *The Journal of Organic Chemistry* **2021**, *86* (17), 12427–12435. https://doi.org/10.1021/acs.joc.1c01441.

(4) Yuan, Y.; Wu, X.-F. Direct Access to 1,1-Dicarbonyl Sulfoxonium Ylides from Aryl Halides or Triflates: Palladium-Catalyzed Carbonylation. *Organic Letters* **2019**, *21* (13), 5310–5314. https://doi.org/10.1021/acs.orglett.9b01926.

(5) Talero, A. G.; Martins, B. S.; Burtoloso, A. C. B. Coupling of Sulfoxonium Ylides with Arynes: A Direct Synthesis of Pro-Chiral Aryl Ketosulfoxonium Ylides and Its Application in the Preparation of α-Aryl Ketones. *Organic Letters* **2018**, *20* (22), 7206–7211. https://doi.org/10.1021/acs.orglett.8b03126.

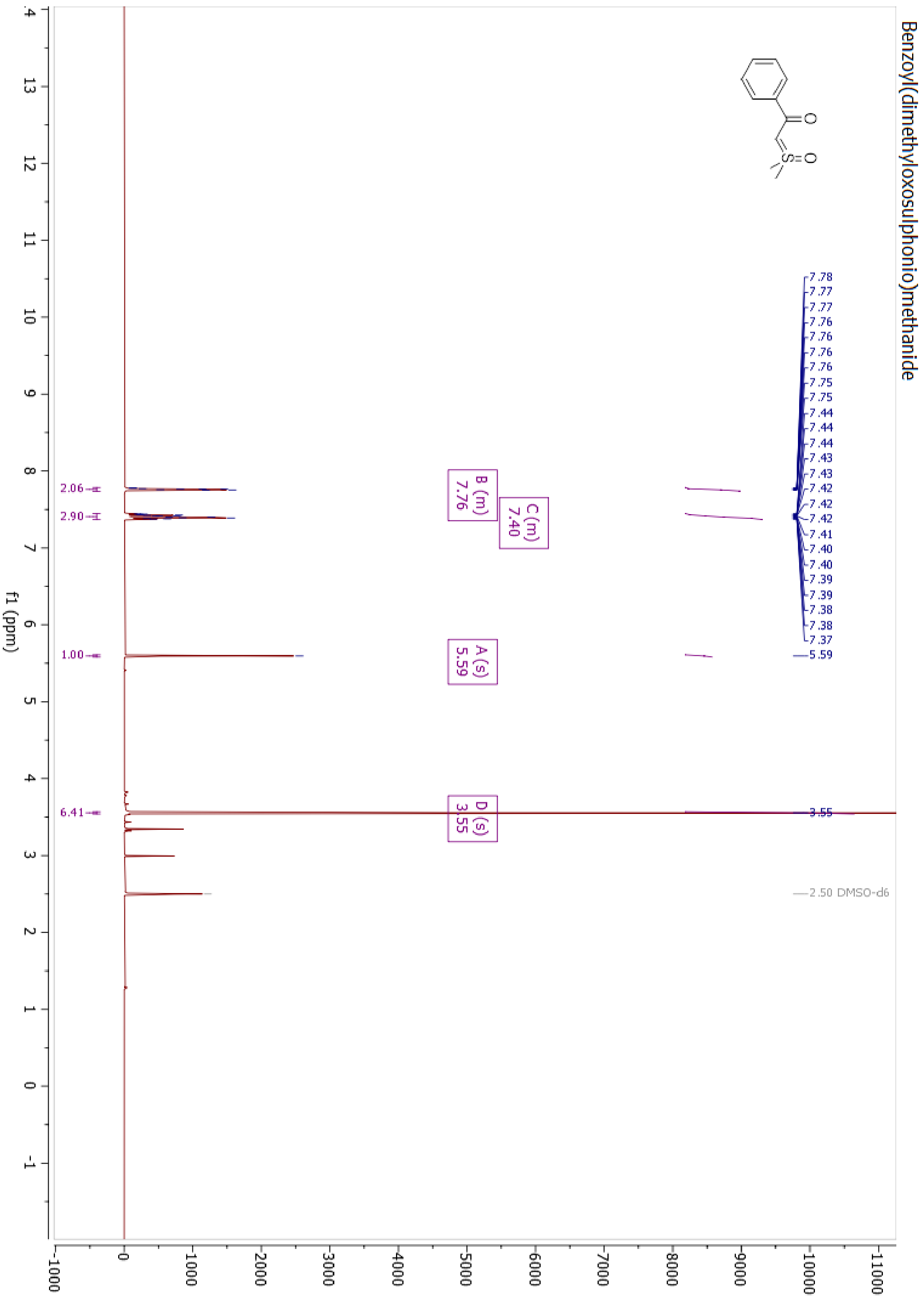

Figure S1. ^{1}H NMR spectrum of **1a** in d_6-DMSO (600 MHz)

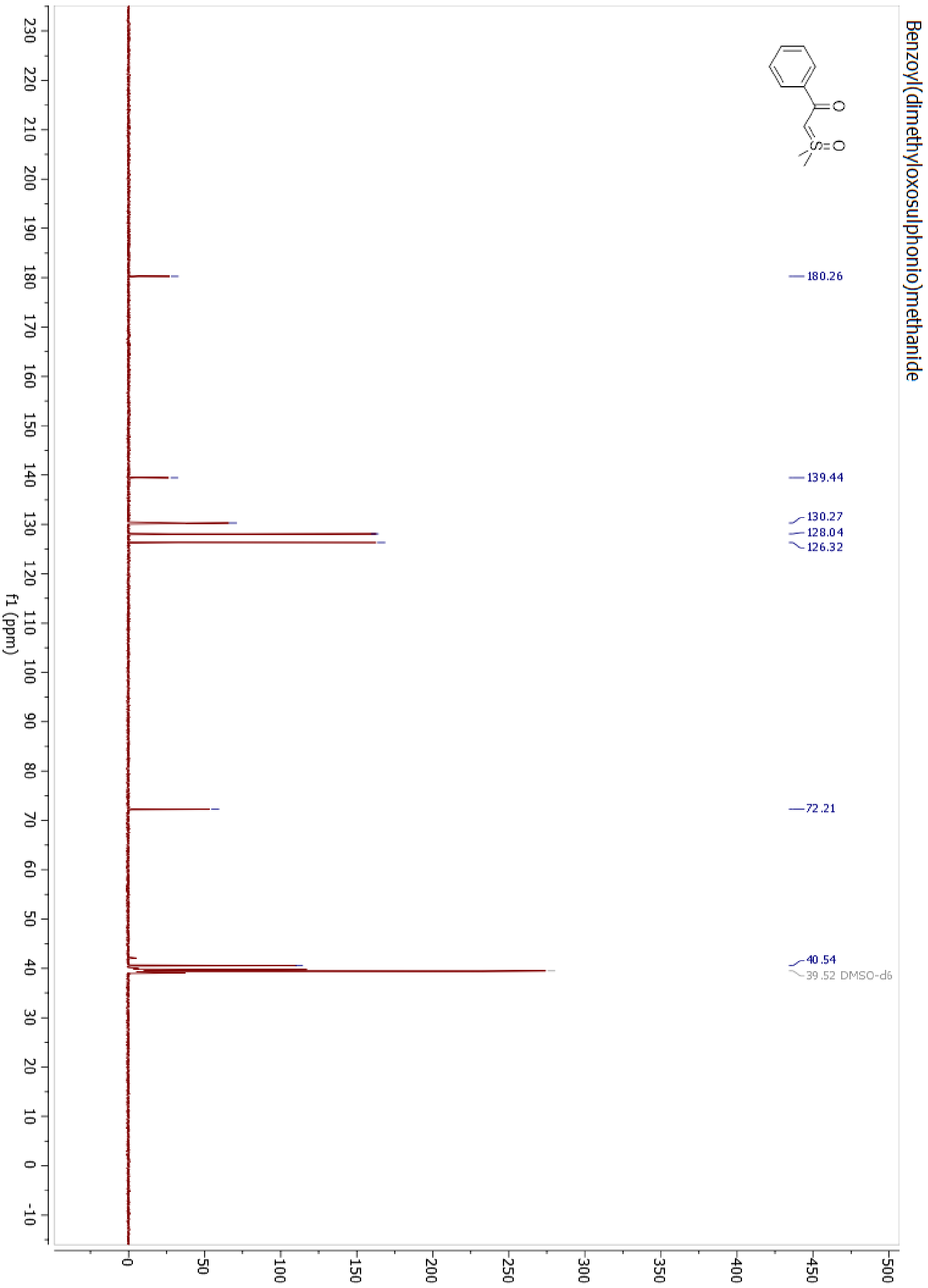

Figure S2. ^{13}C NMR spectrum of **1a** in d_6-DMSO (151 MHz)

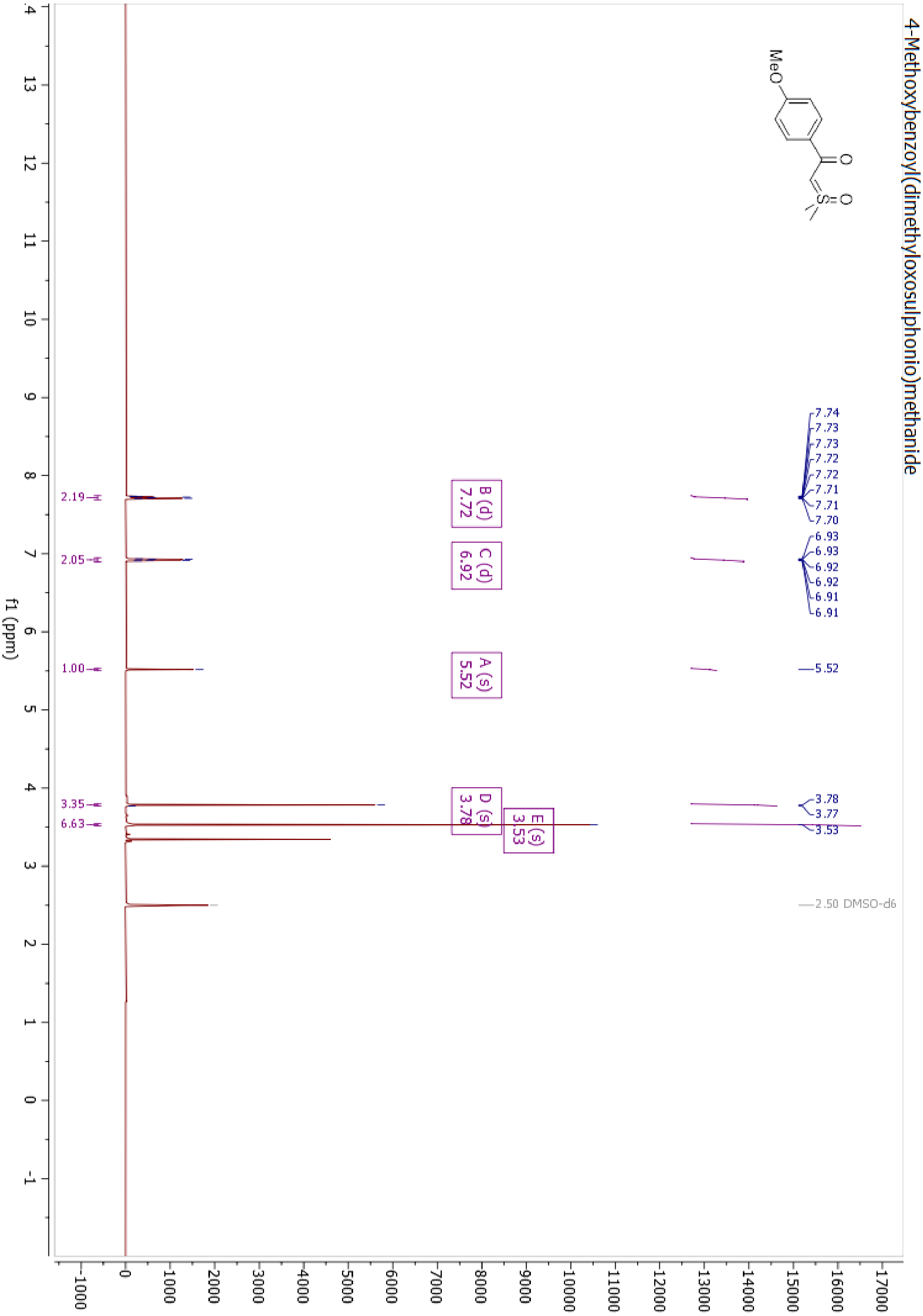

Figure S3. ^{1}H NMR spectrum of **1b** in d_6-DMSO (600 MHz)

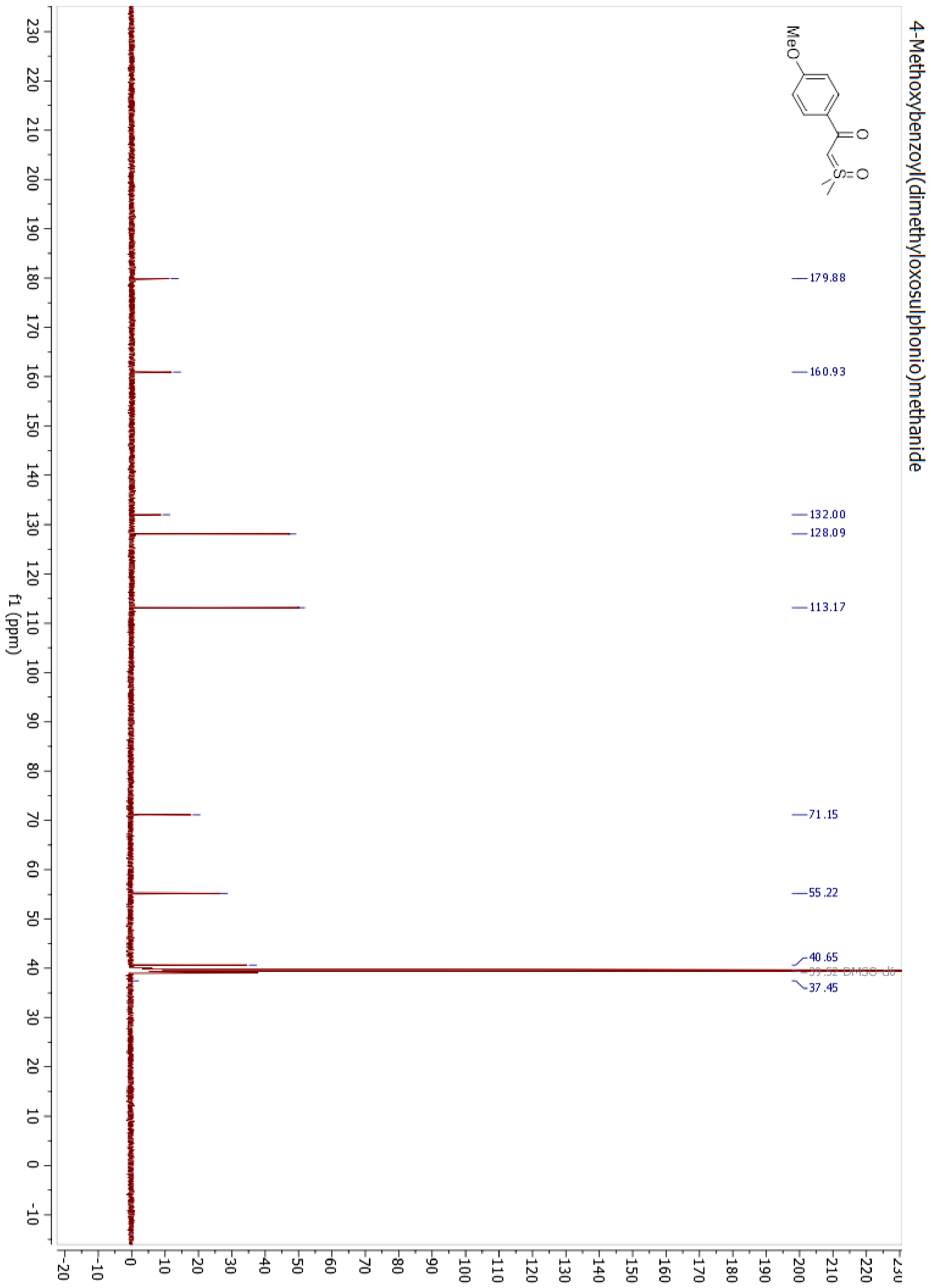

Figure S4. ^{13}C NMR spectrum of **1b** in d_6-DMSO (151 MHz)

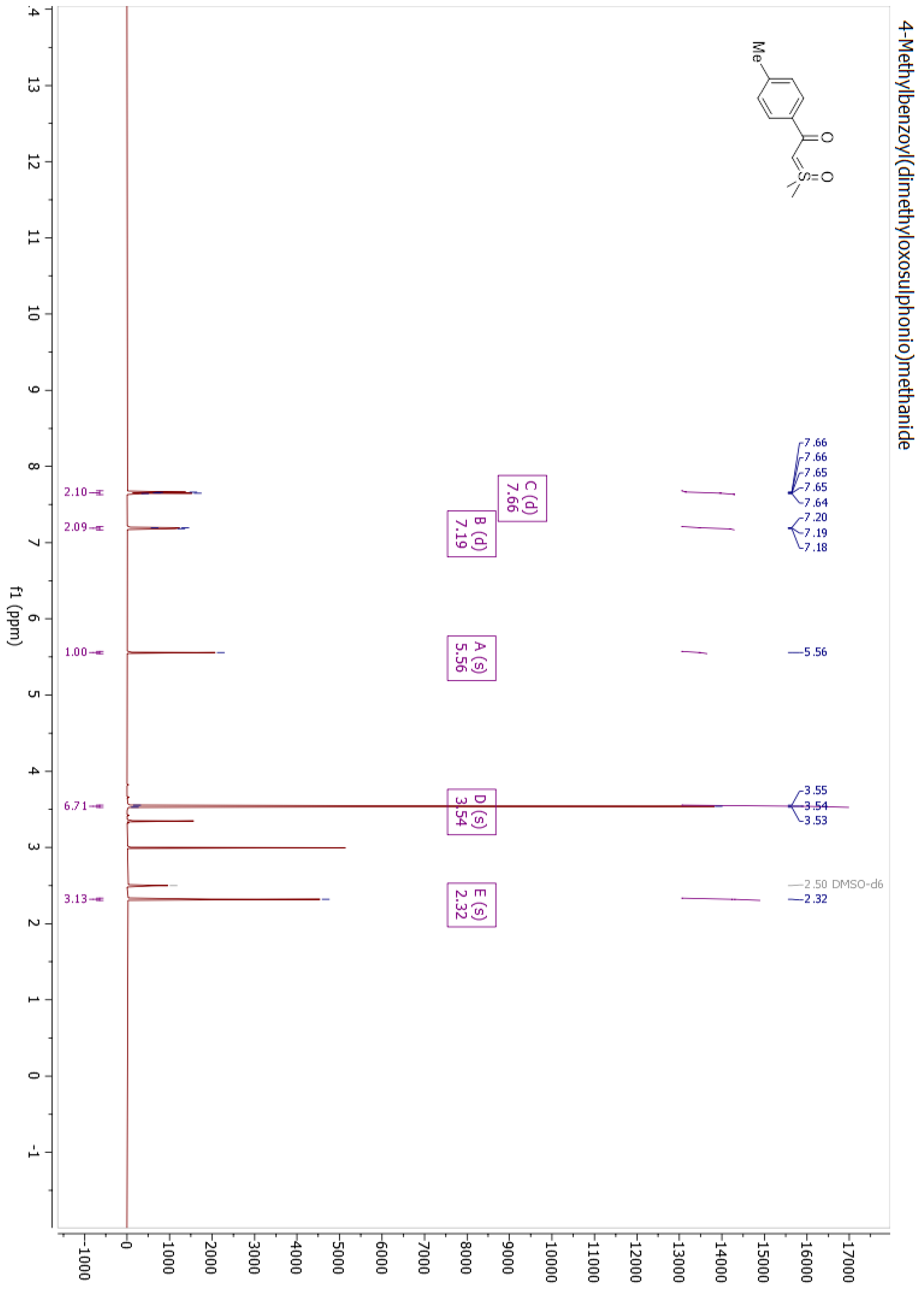

Figure S5. ^{1}H NMR spectrum of **1c** in d_6-DMSO (600 MHz)

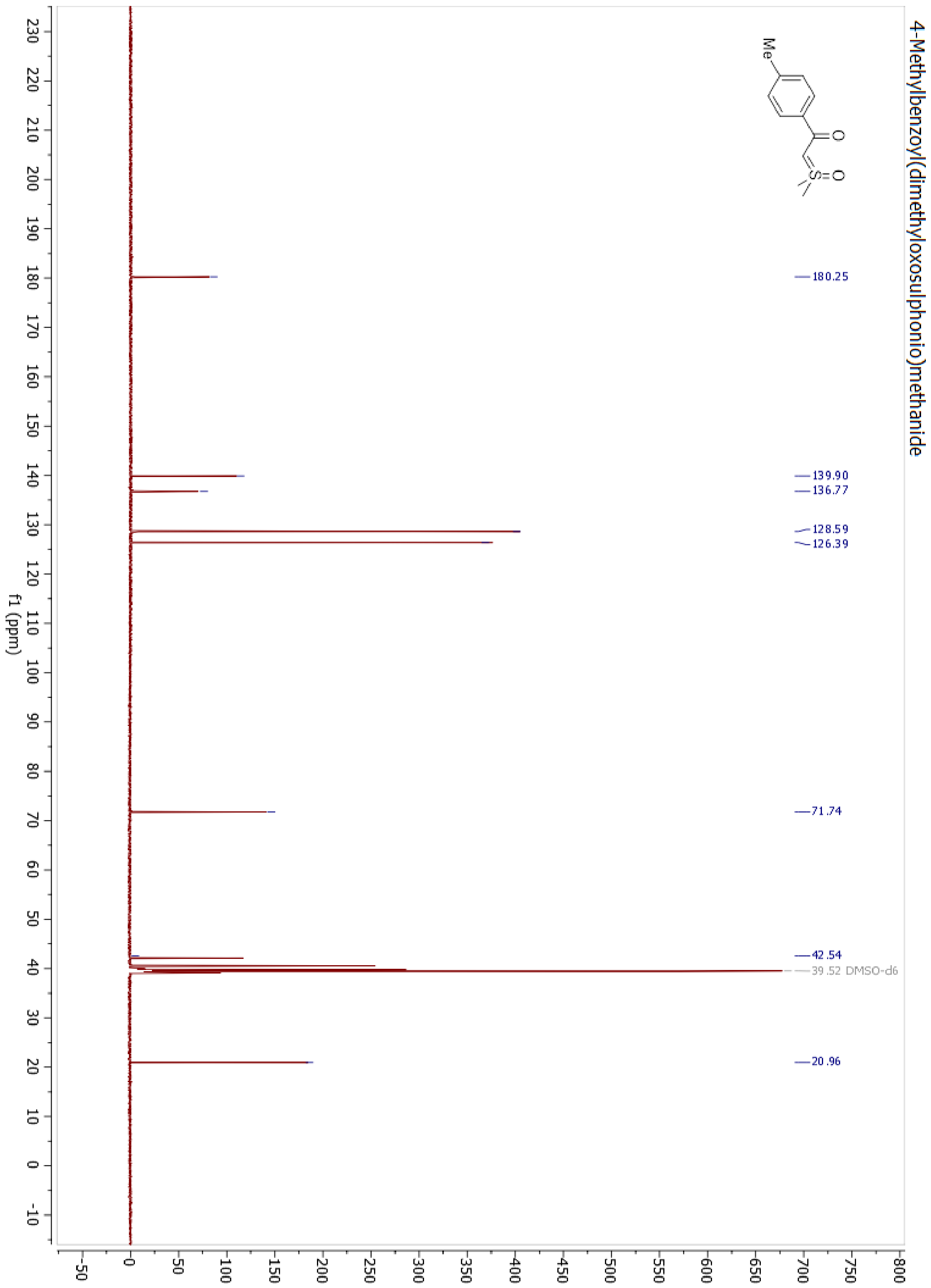

Figure S6. ^{13}C NMR spectrum of **1c** in d_6-DMSO (151 MHz)

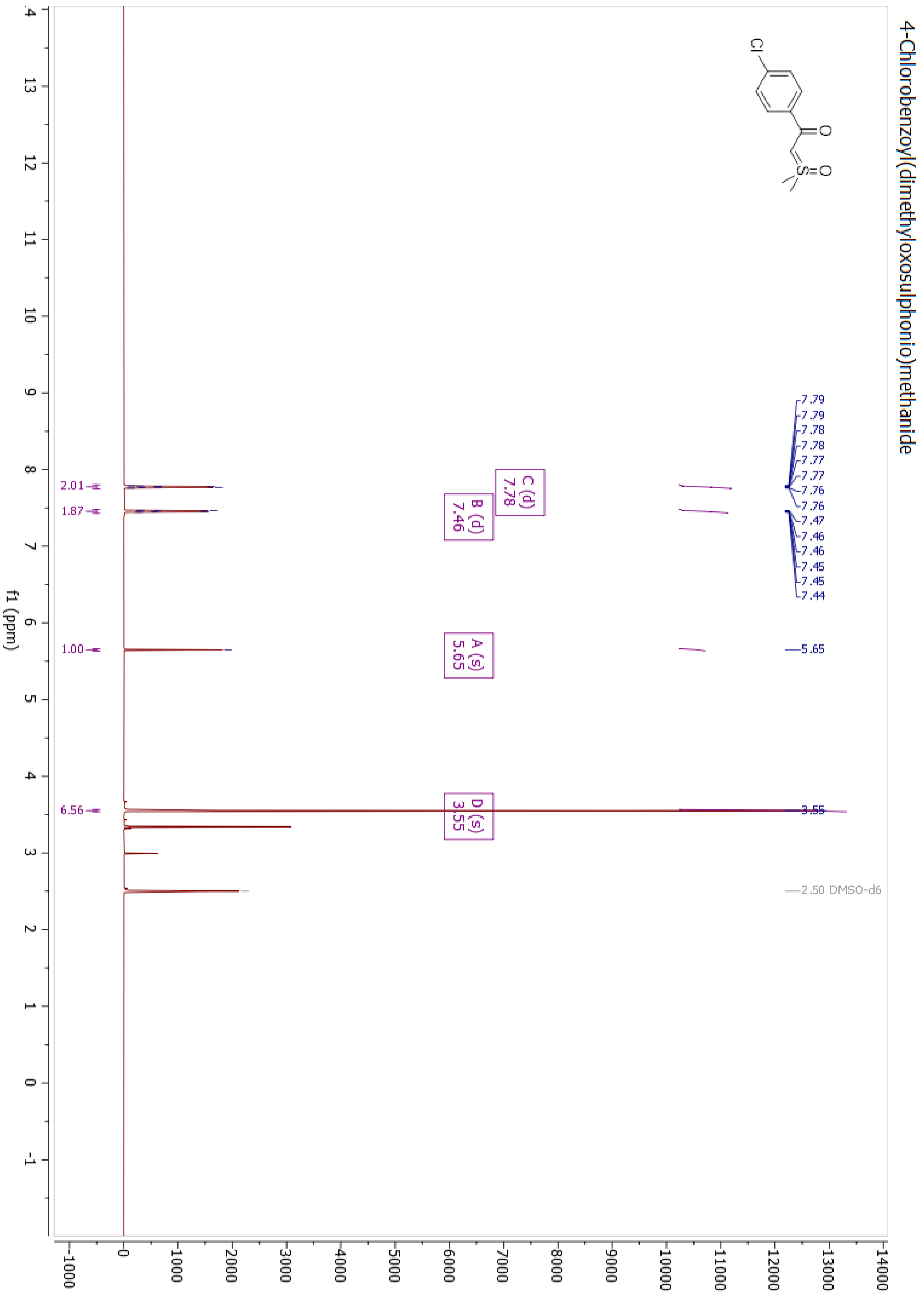

Figure S7. ^{1}H NMR spectrum of **1d** in d_6-DMSO (600 MHz)

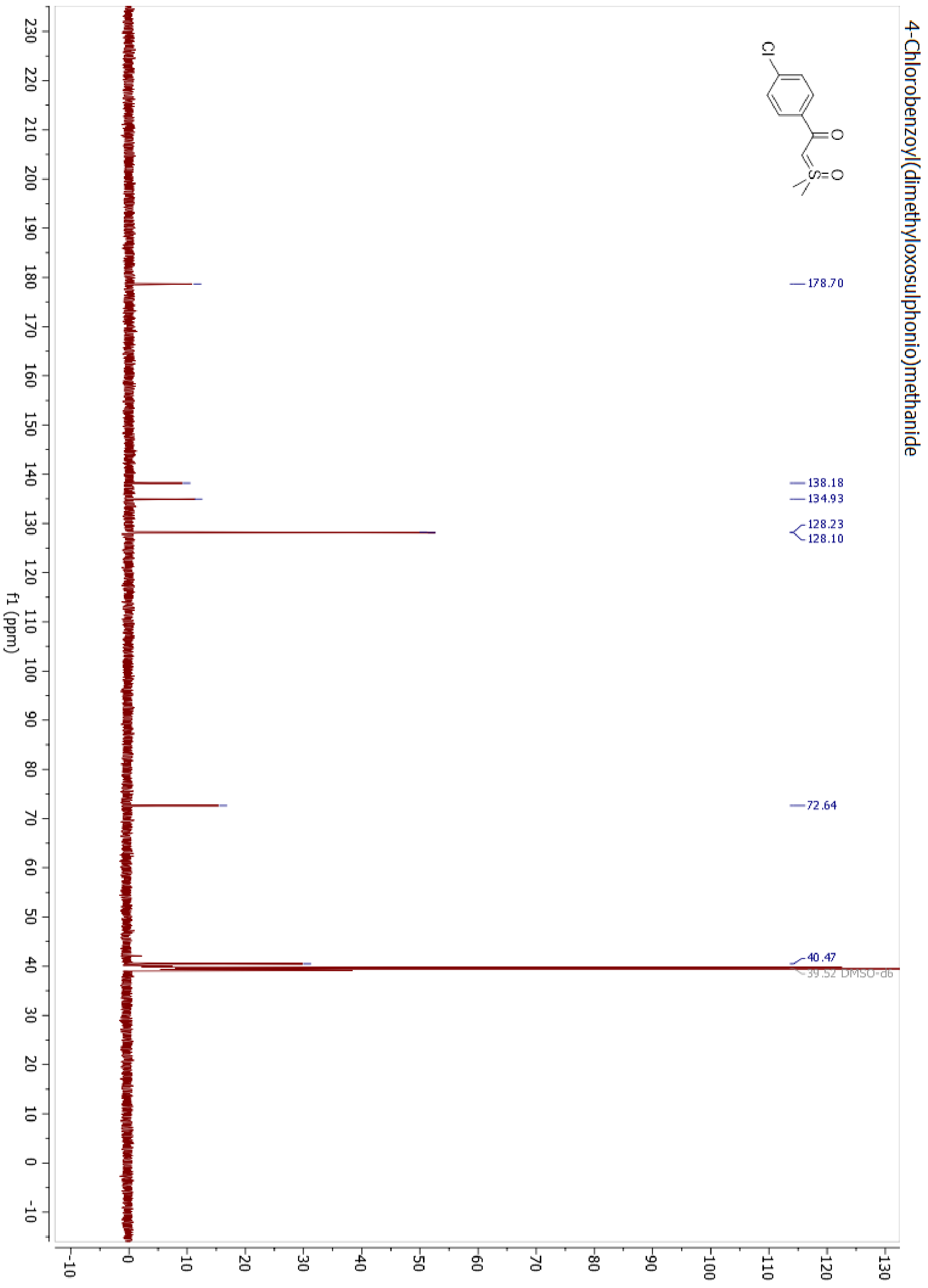

Figure S8. ^{13}C NMR spectrum of **1d** in d_6-DMSO (151 MHz)

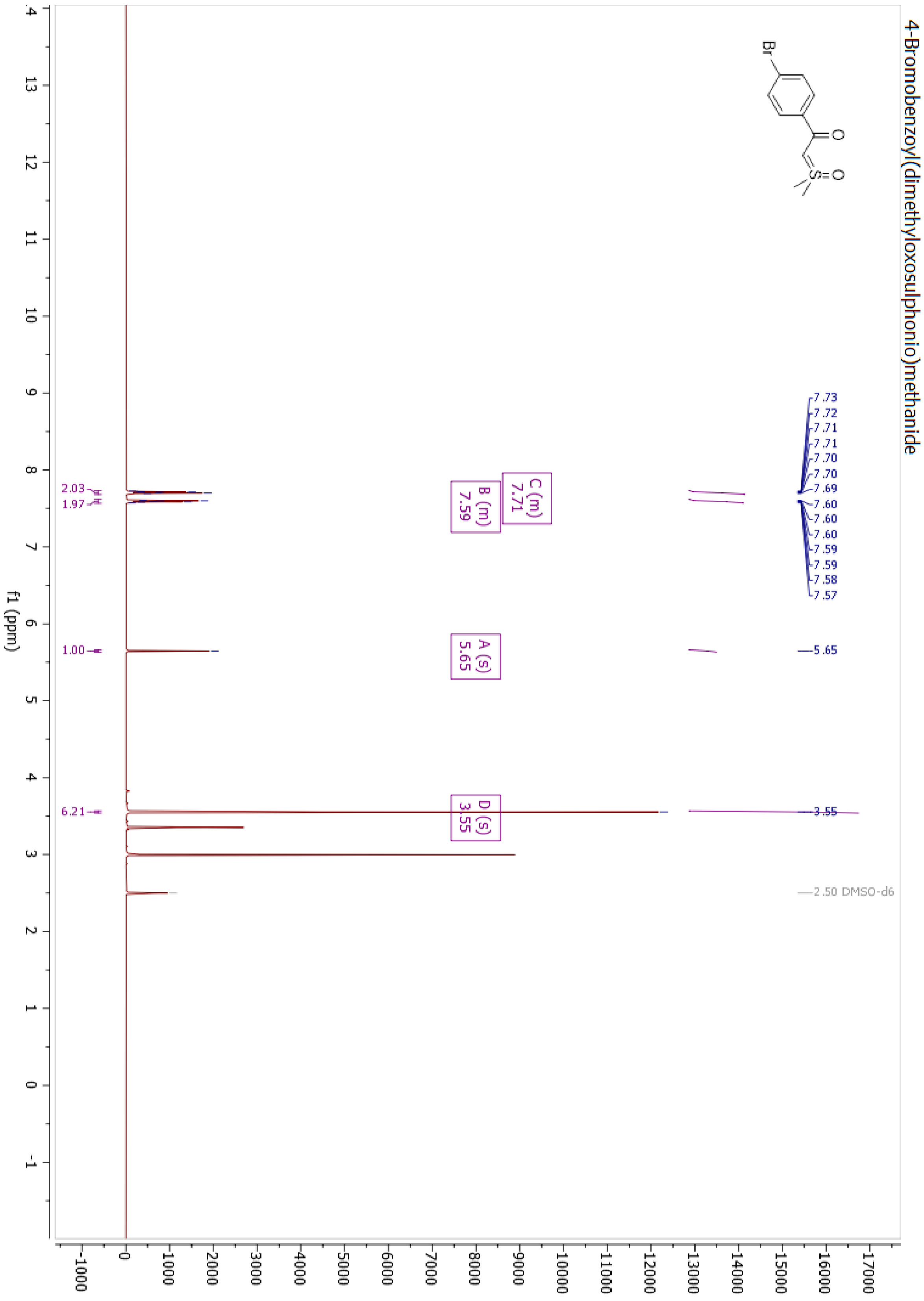

Figure S9. ^{1}H NMR spectrum of **1e** in d_6-DMSO (600 MHz)

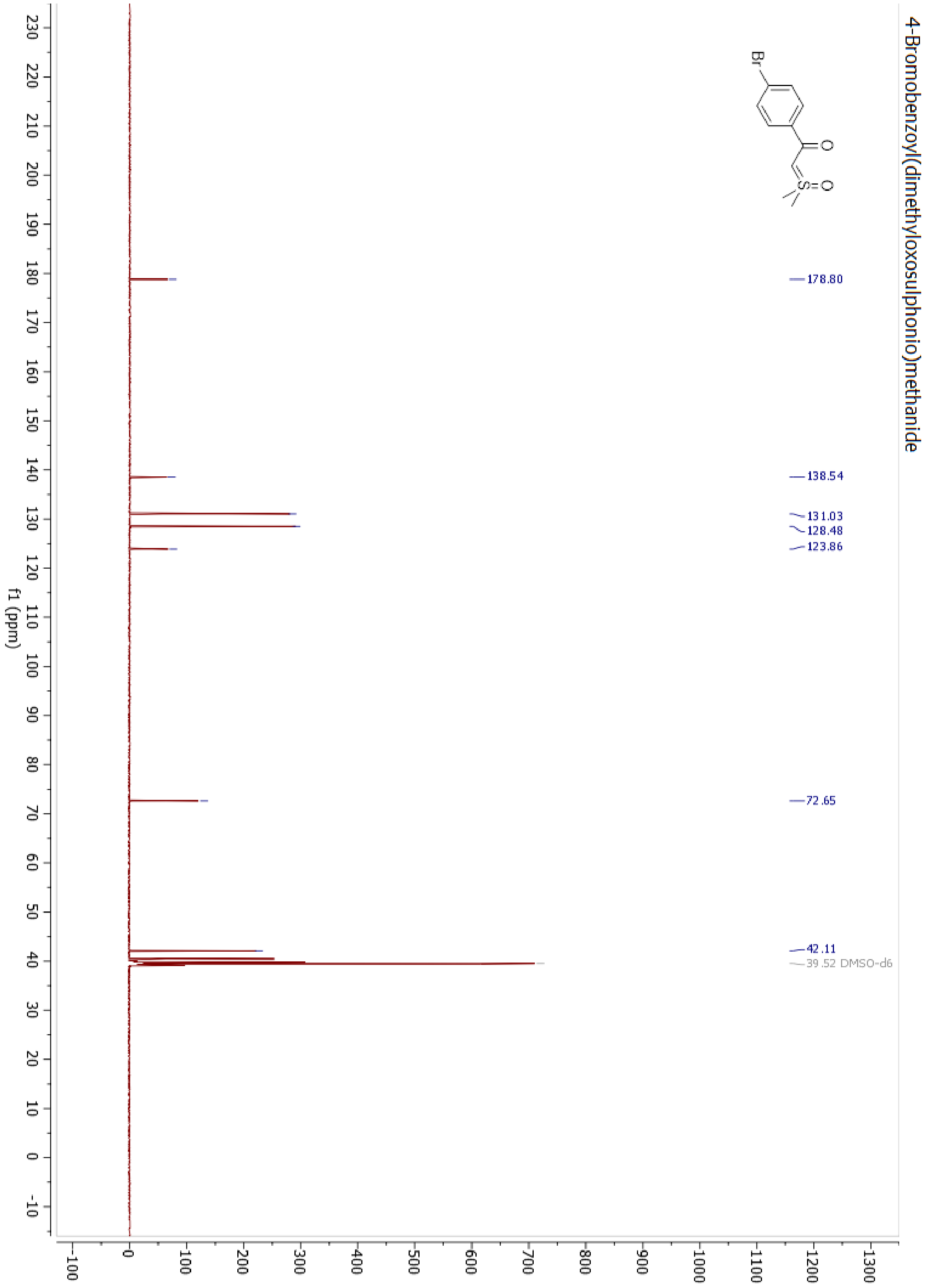

Figure S10. ^{13}C NMR spectrum of **1e** in d_6-DMSO (151 MHz)

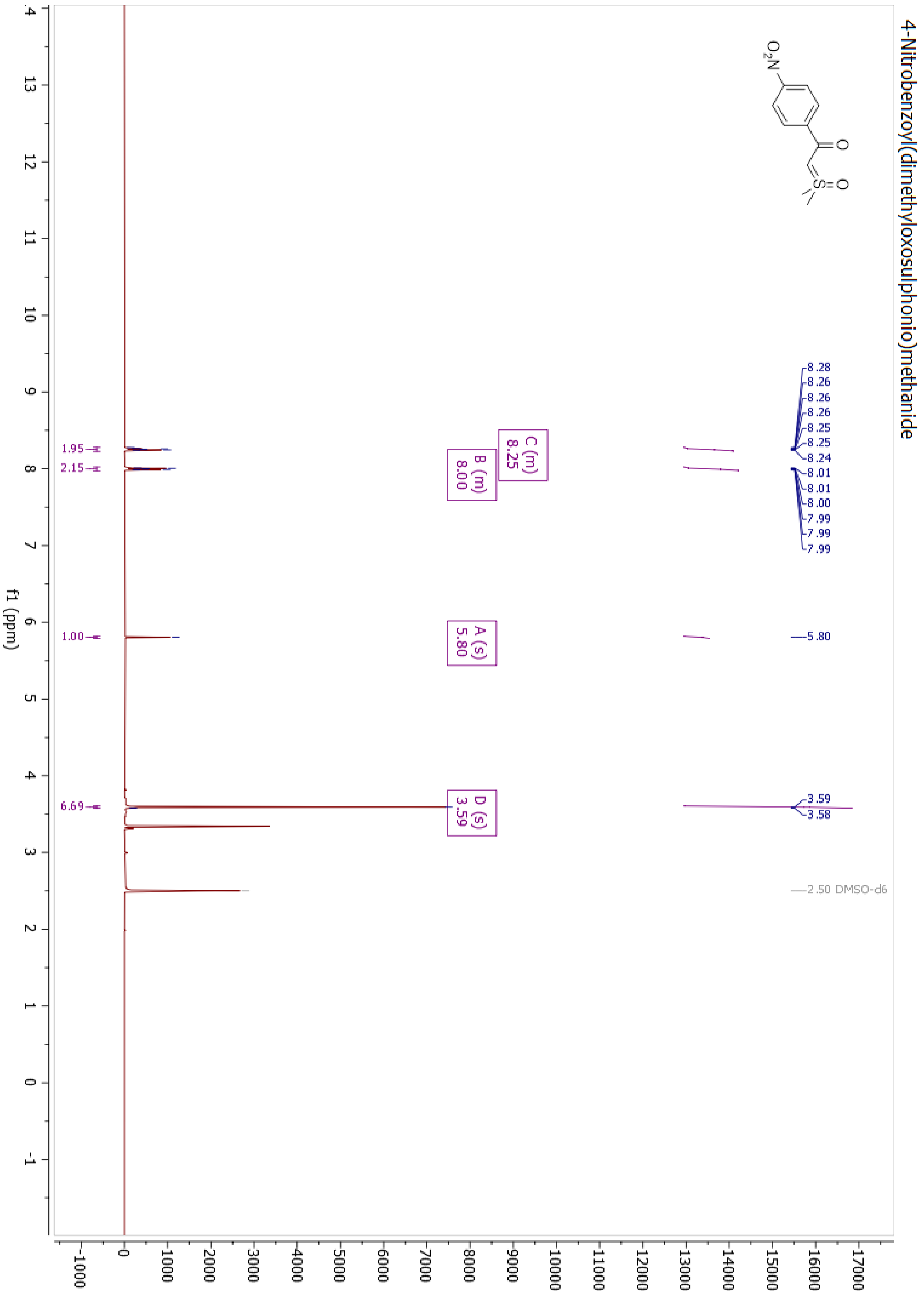

Figure S11. ^{1}H NMR spectrum of **1f** in d_6-DMSO (600 MHz)

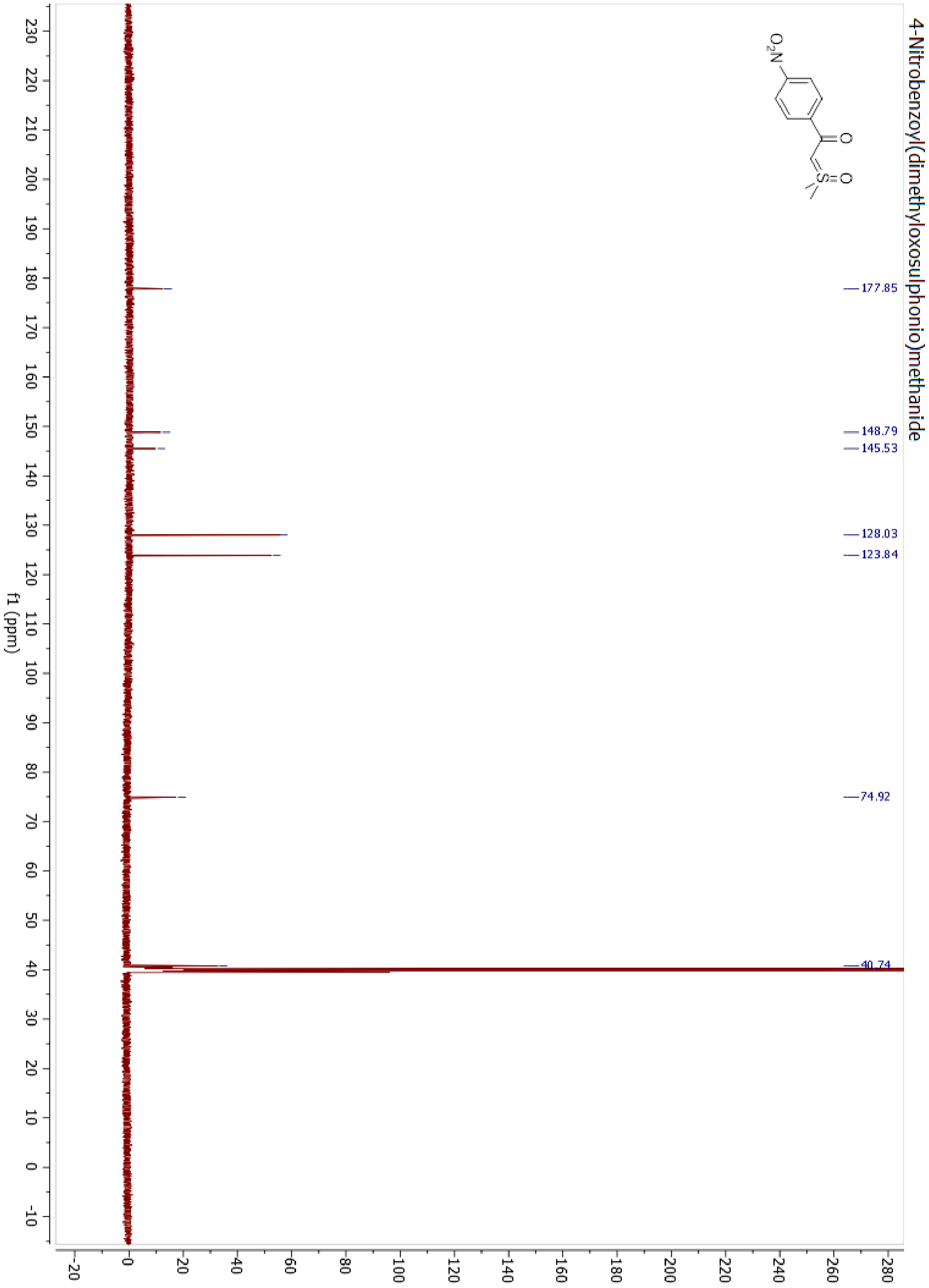

Figure S12. ^{13}C NMR spectrum of **1f** in d_6-DMSO (151 MHz)

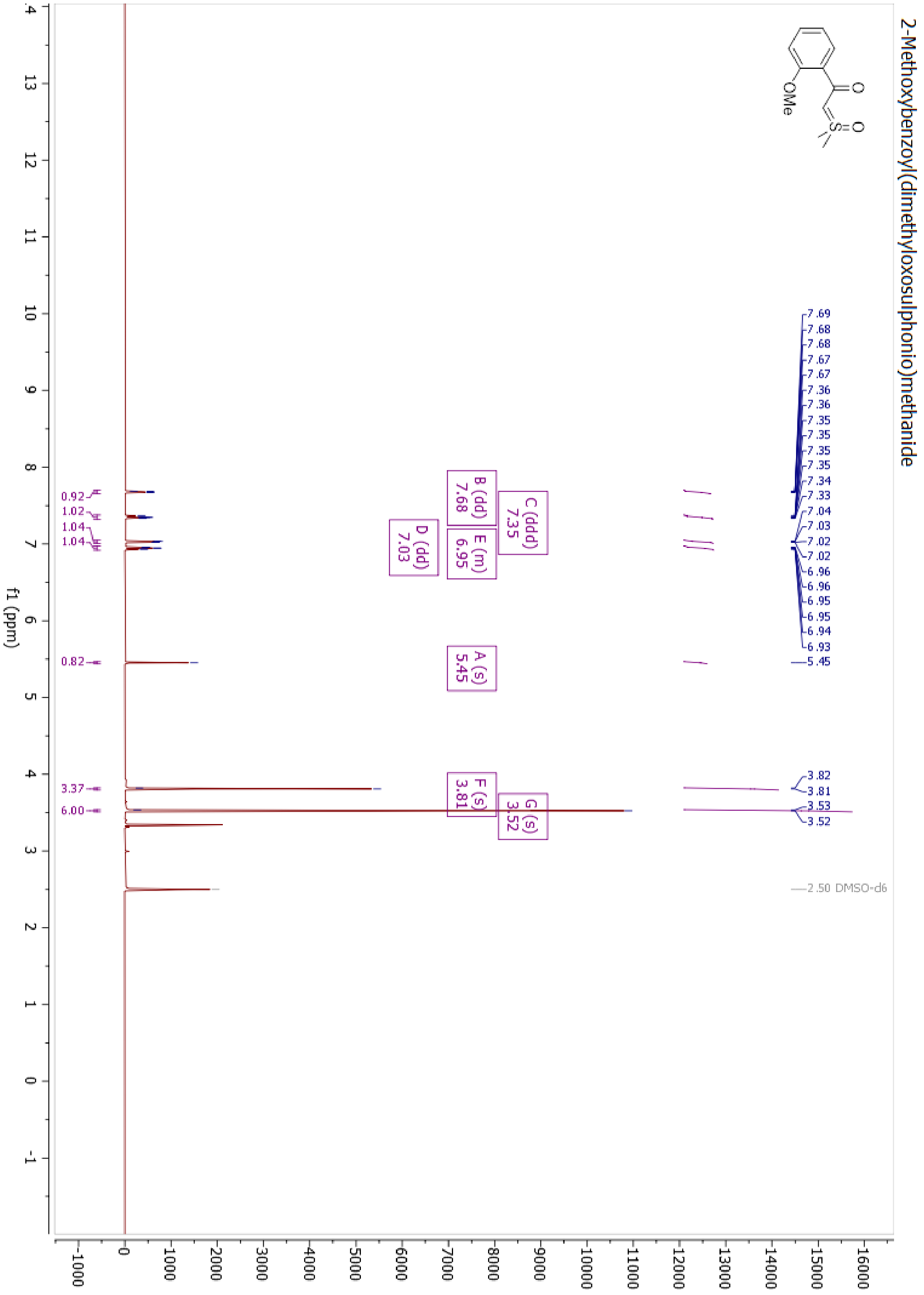

Figure S13. ^{1}H NMR spectrum of **1g** in d_6-DMSO (600 MHz)

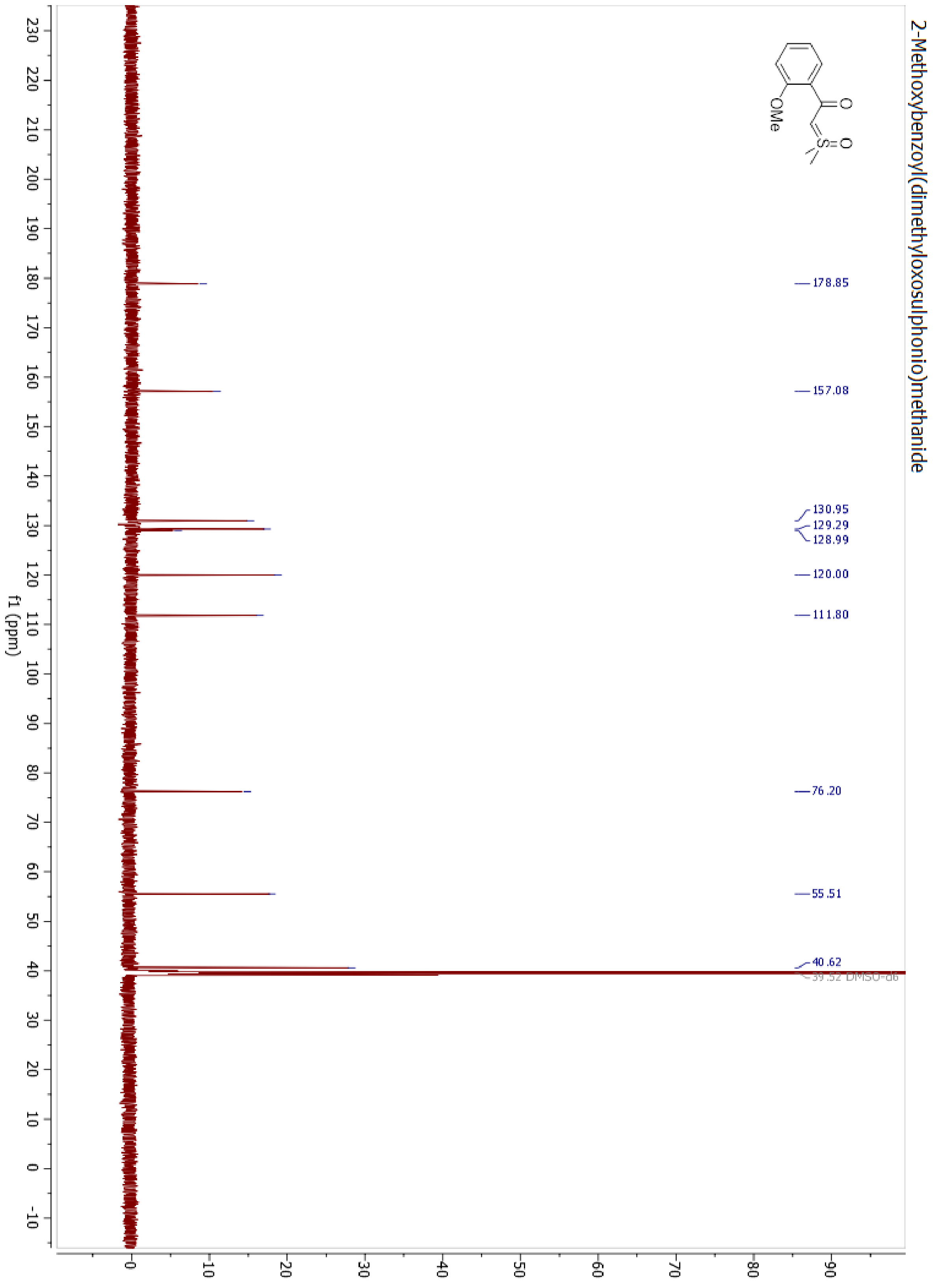

Figure S14. ^{13}C NMR spectrum of **1g** in d_6-DMSO (151 MHz)

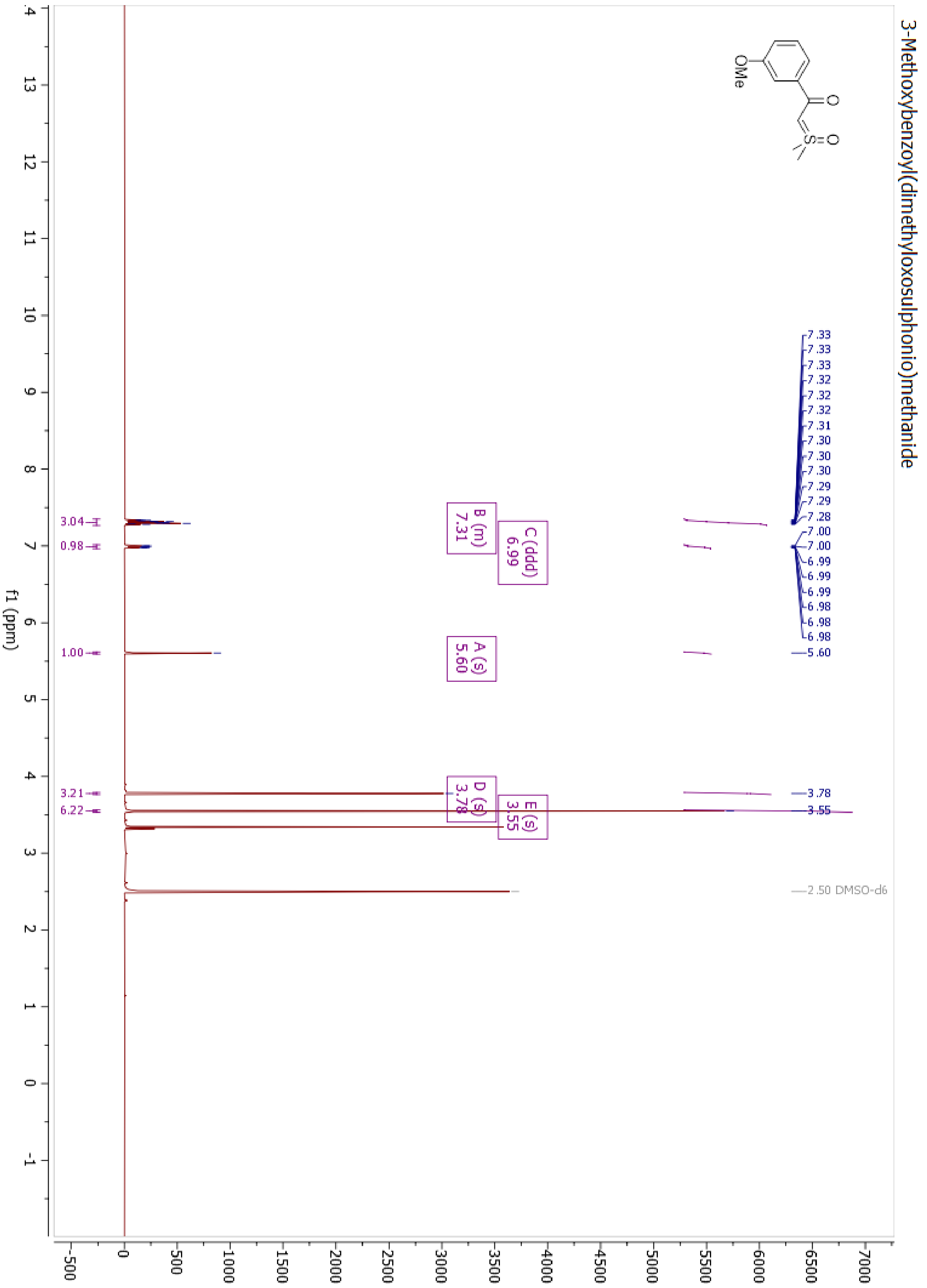

Figure S15. ^{1}H NMR spectrum of **1h** in d_6-DMSO (600 MHz)

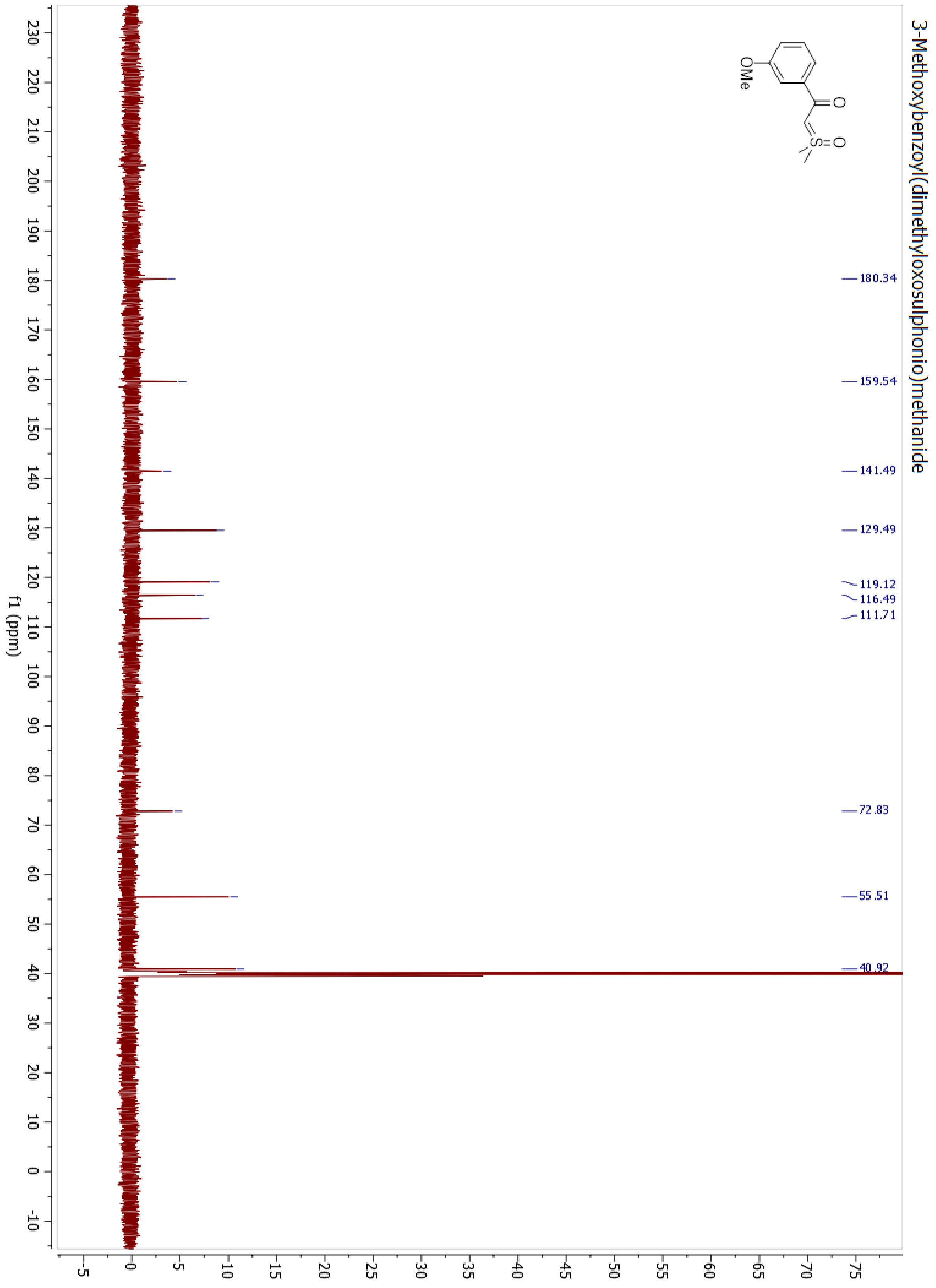

Figure S16. ^{13}C NMR spectrum of **1h** in d_6-DMSO (151 MHz)

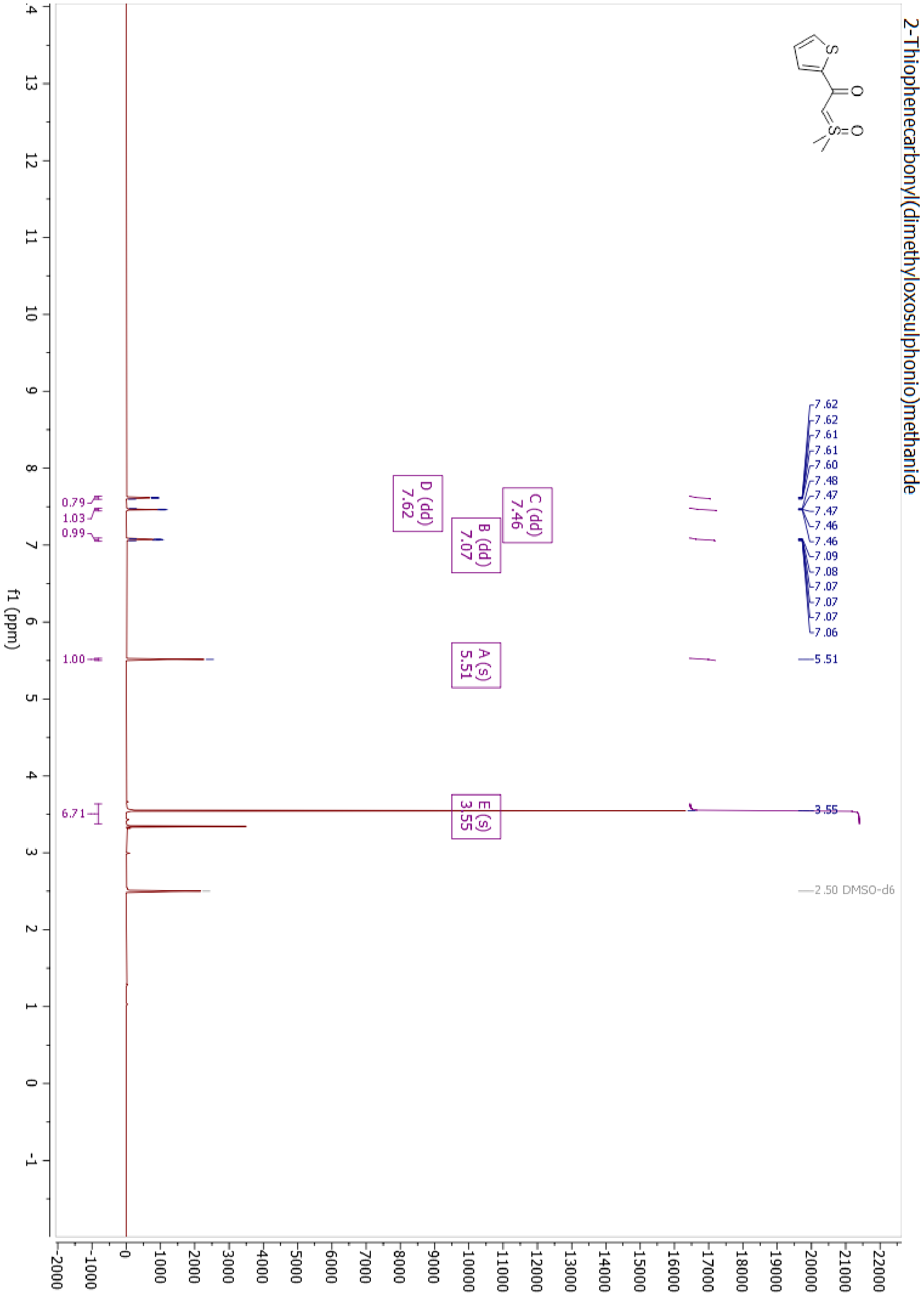

Figure S17. ^{1}H NMR spectrum of **1i** in d_6-DMSO (600 MHz)

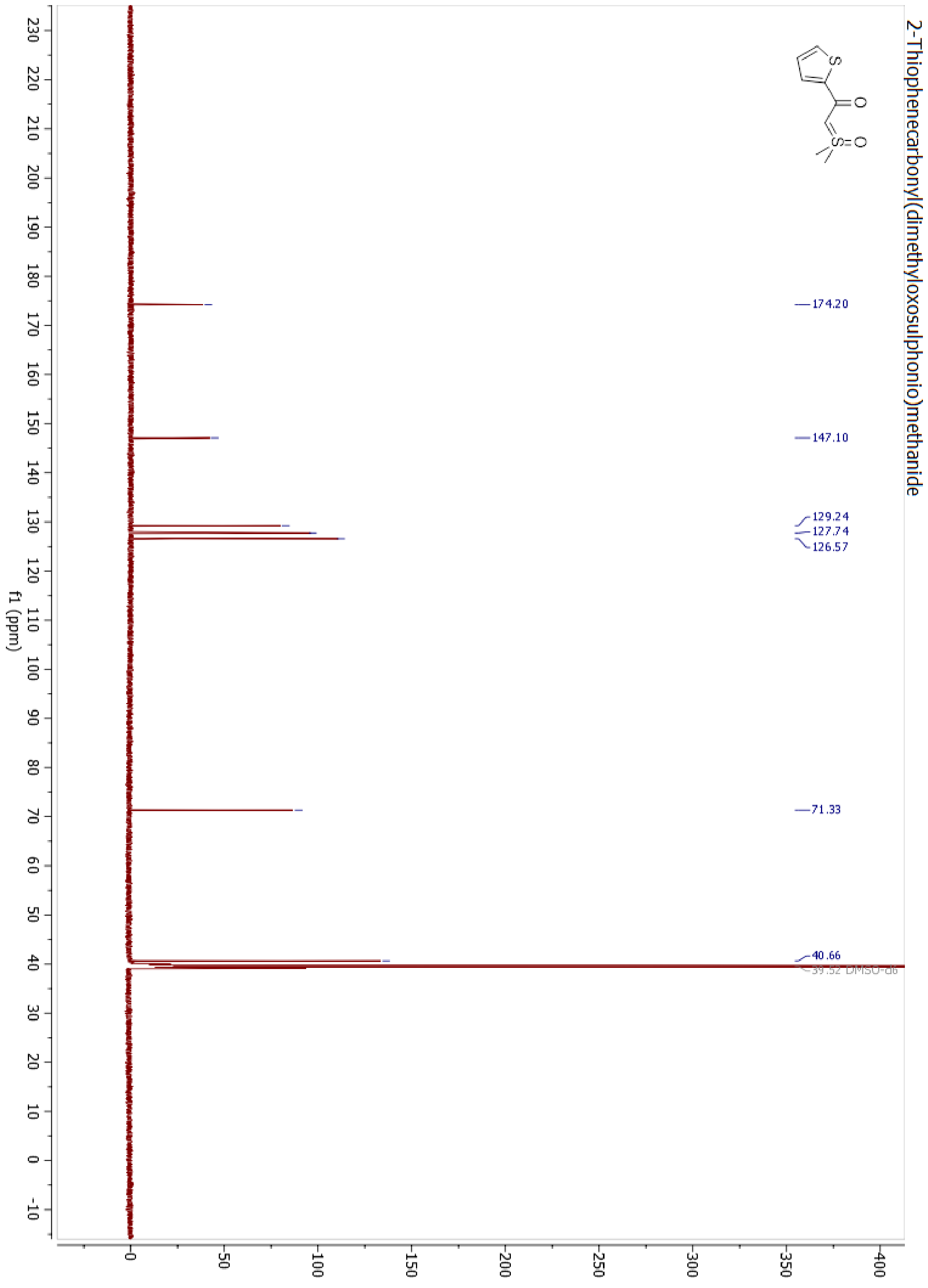

Figure S18. ^{13}C NMR spectrum of **1i** in d_6-DMSO (151 MHz)

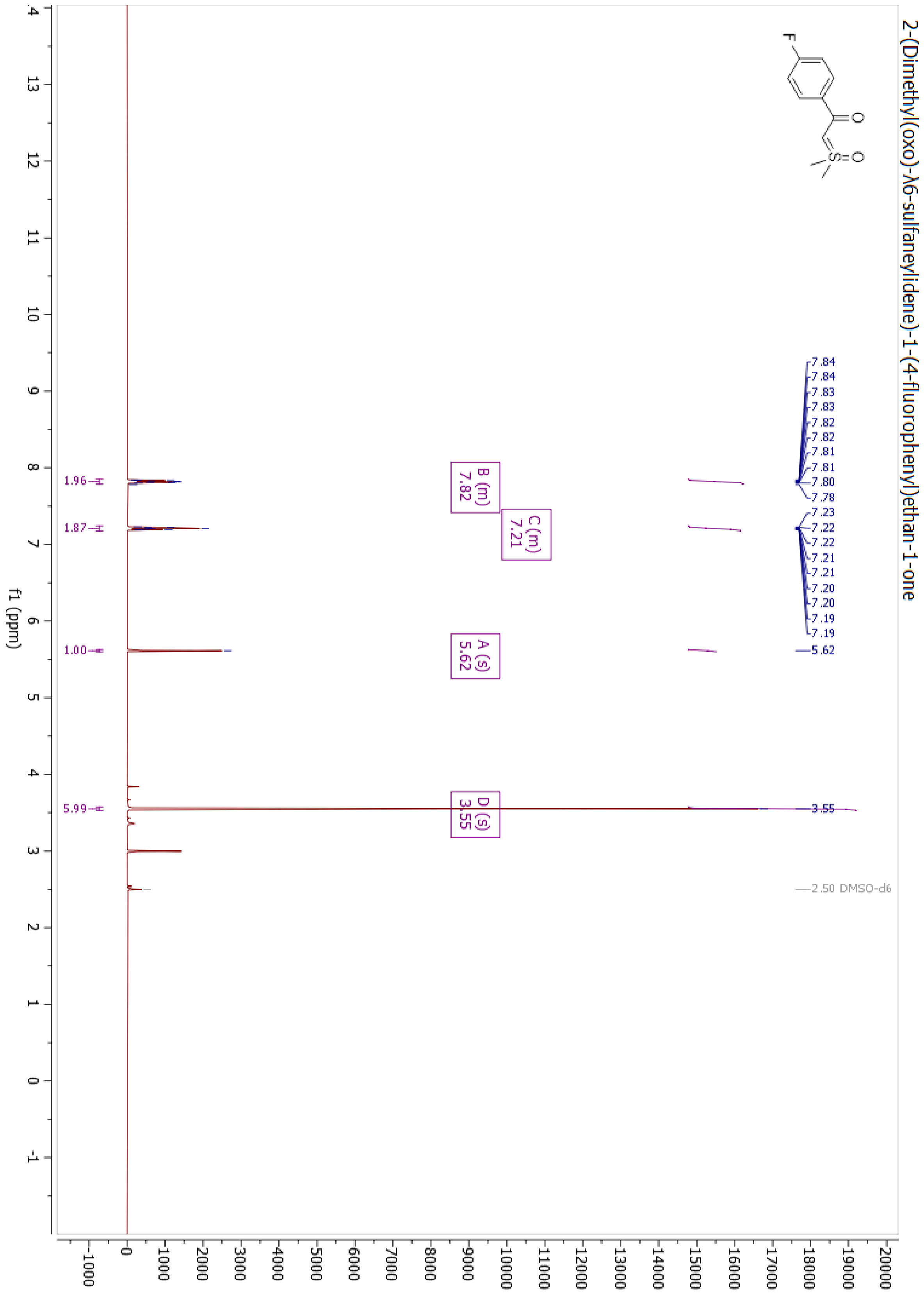

Figure S19. ^{1}H NMR spectrum of **1j** in d_6-DMSO (600 MHz)

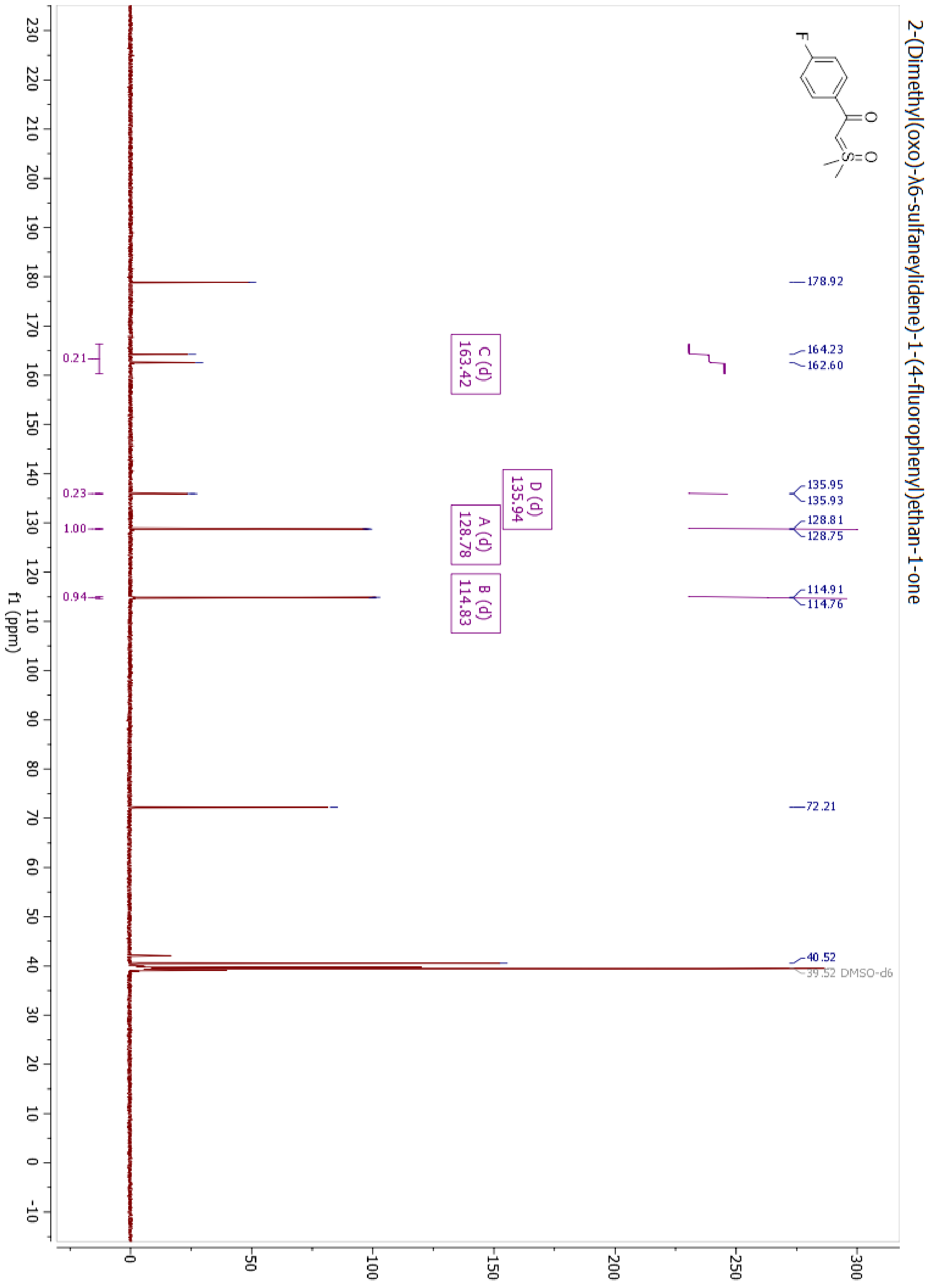

Figure S20. ^{13}C NMR spectrum of **1j** in d_6-DMSO (151 MHz)

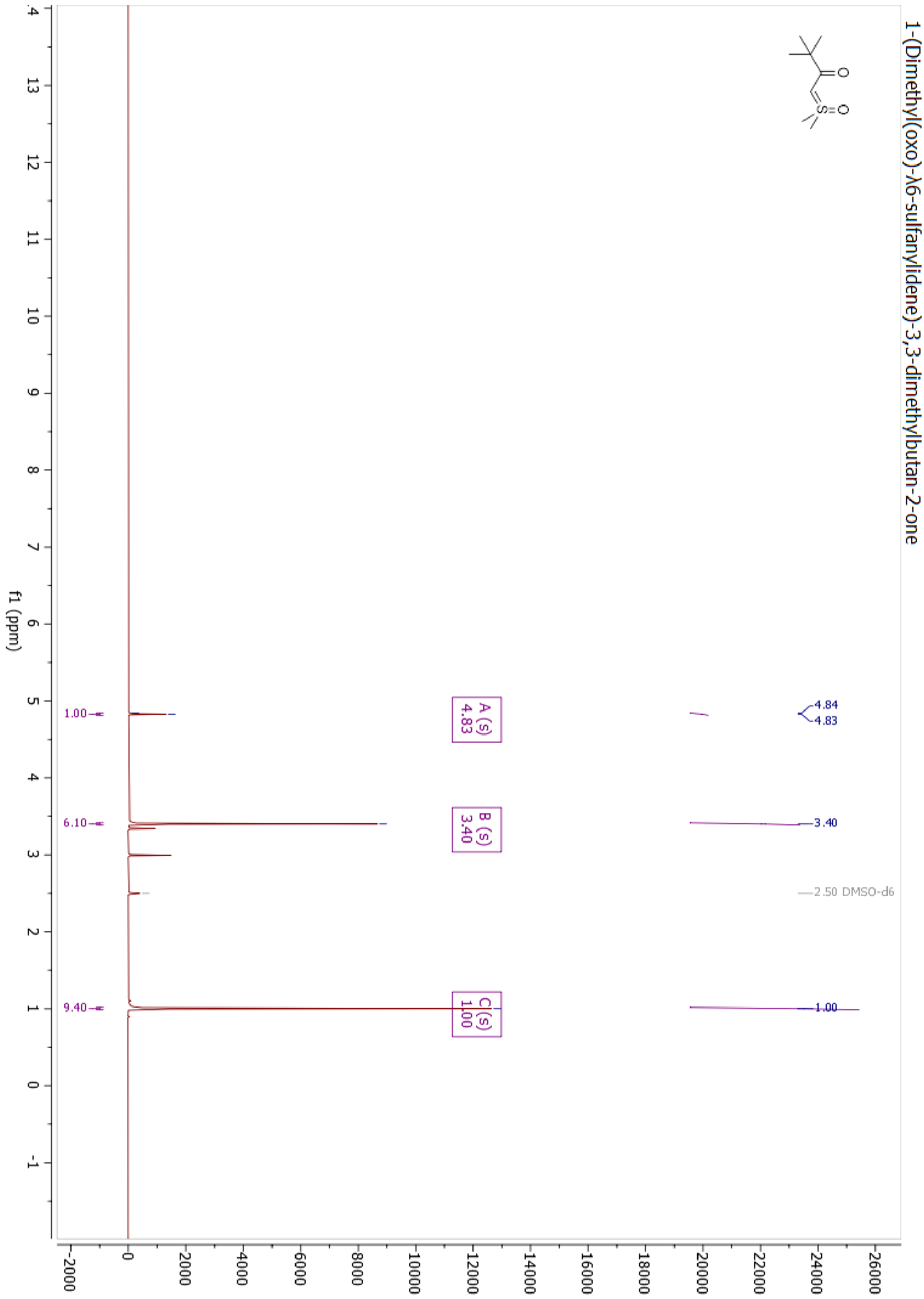

Figure S21. ^{1}H NMR spectrum of **1k** in d_6-DMSO (600 MHz)

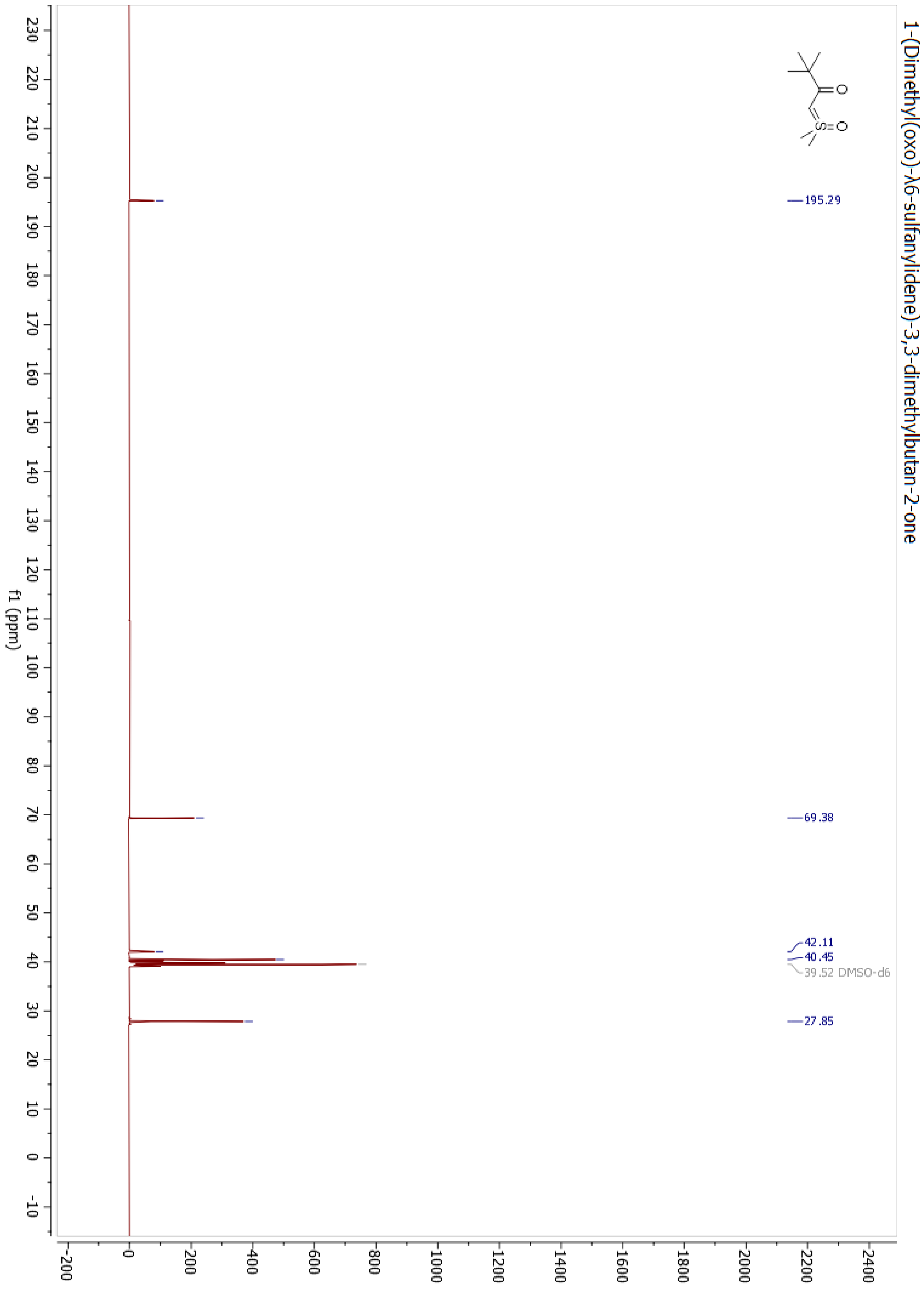

Figure S22. ^{13}C NMR spectrum of **1k** in d_6-DMSO (151 MHz)

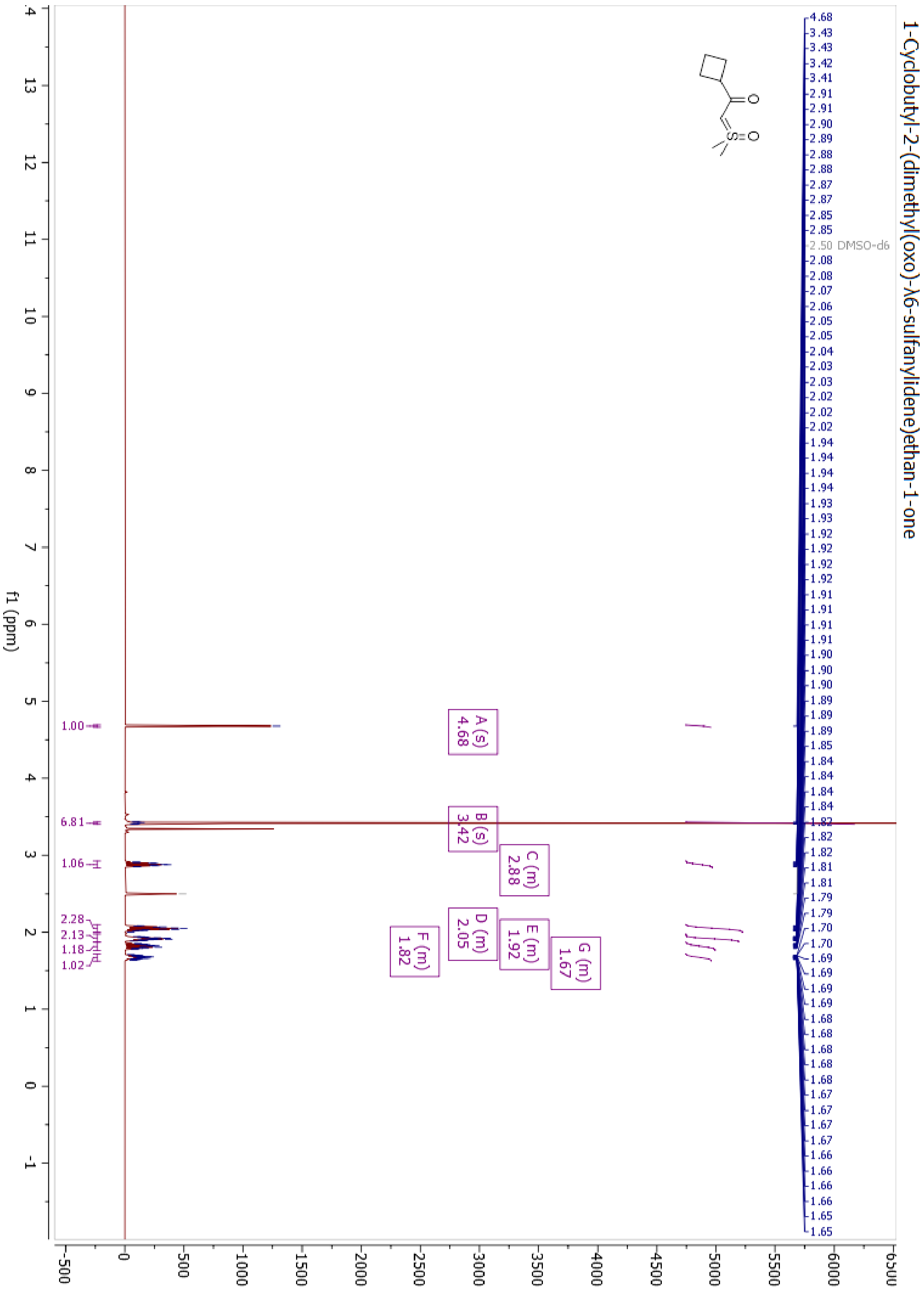

Figure S23. ^{1}H NMR spectrum of **1l** in d_6-DMSO (600 MHz)

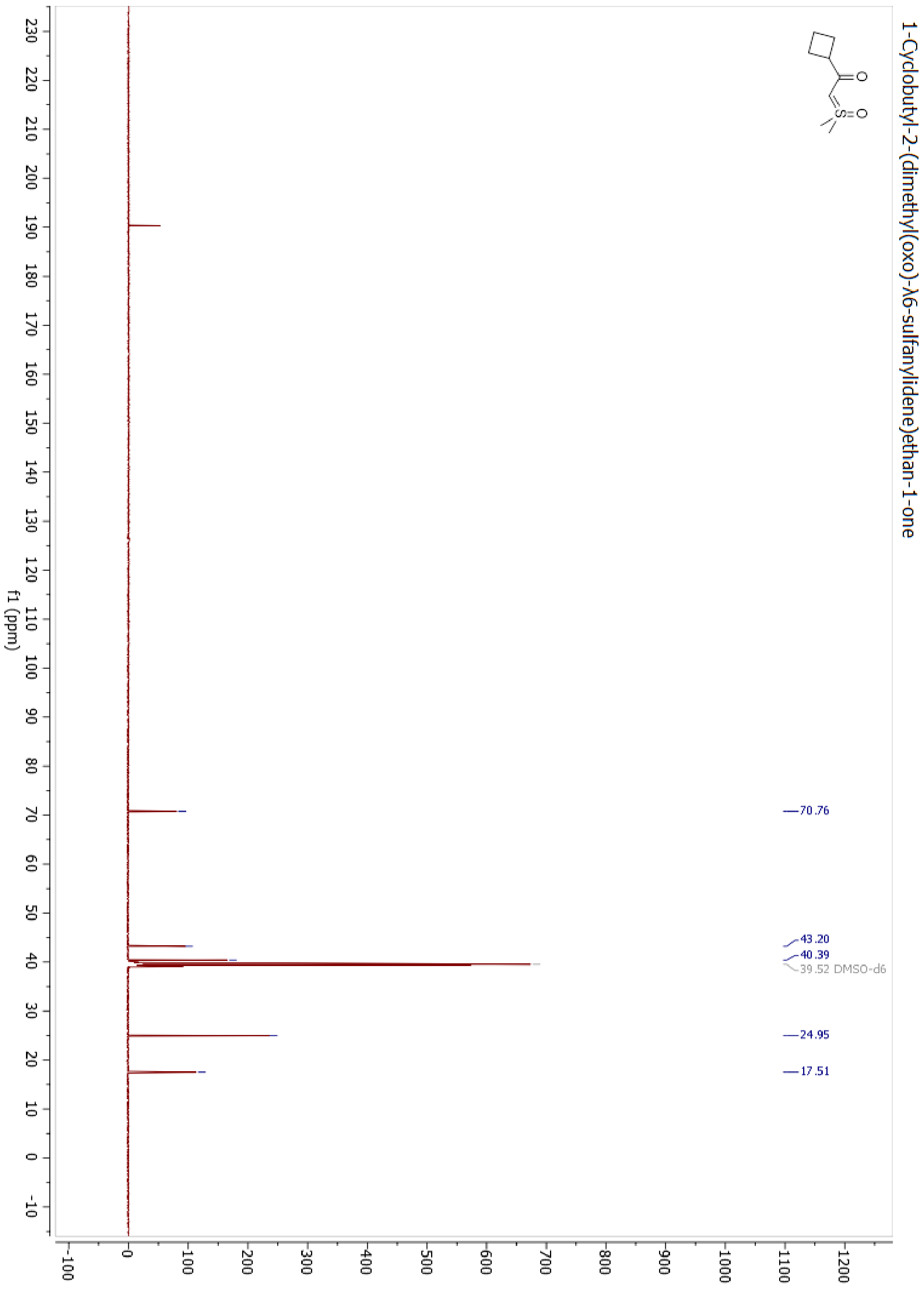

Figure S24. ^{13}C NMR spectrum of **1l** in d_6-DMSO (151 MHz)

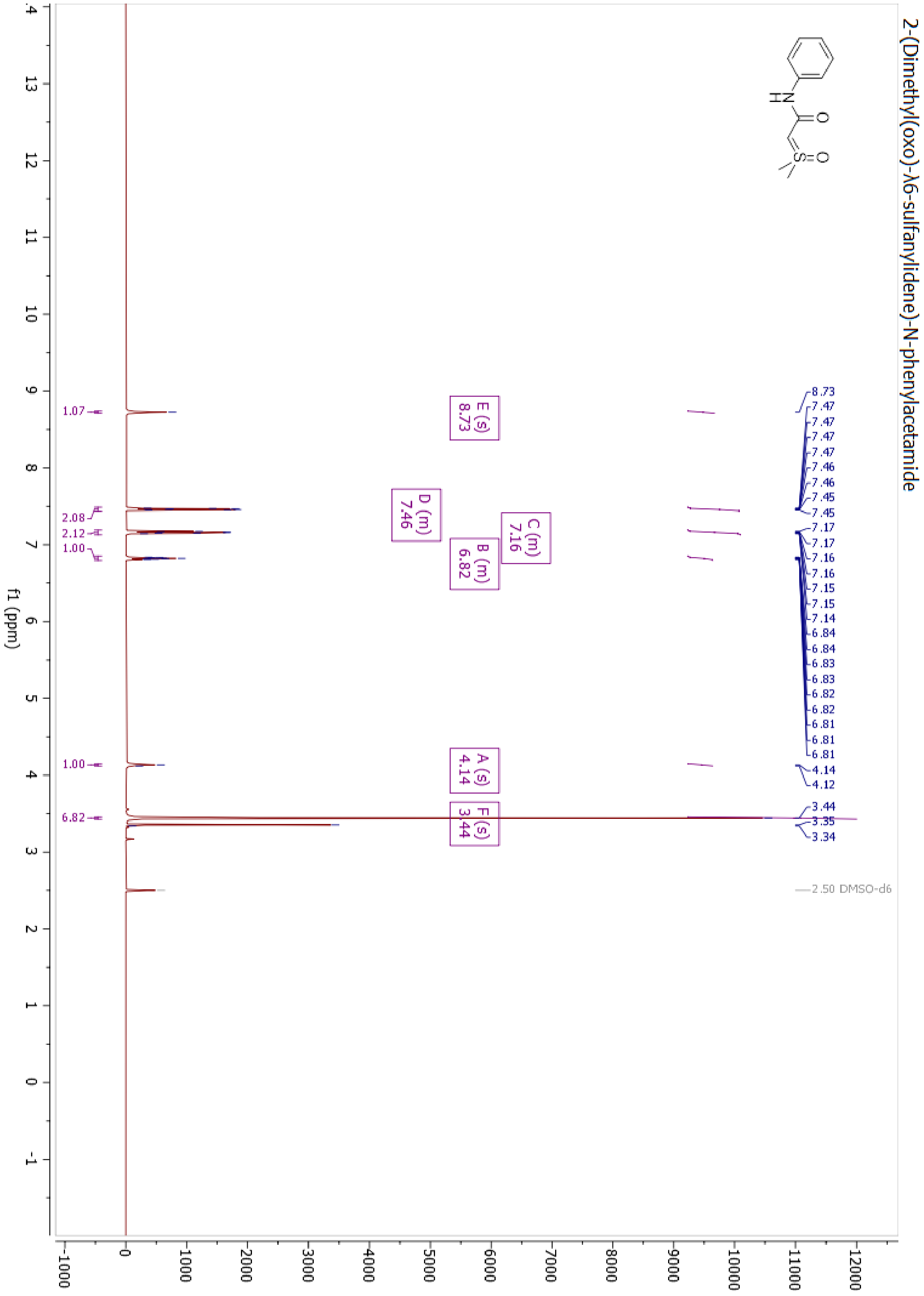

Figure S25. ^{1}H NMR spectrum of **1m** in d_6-DMSO (600 MHz)

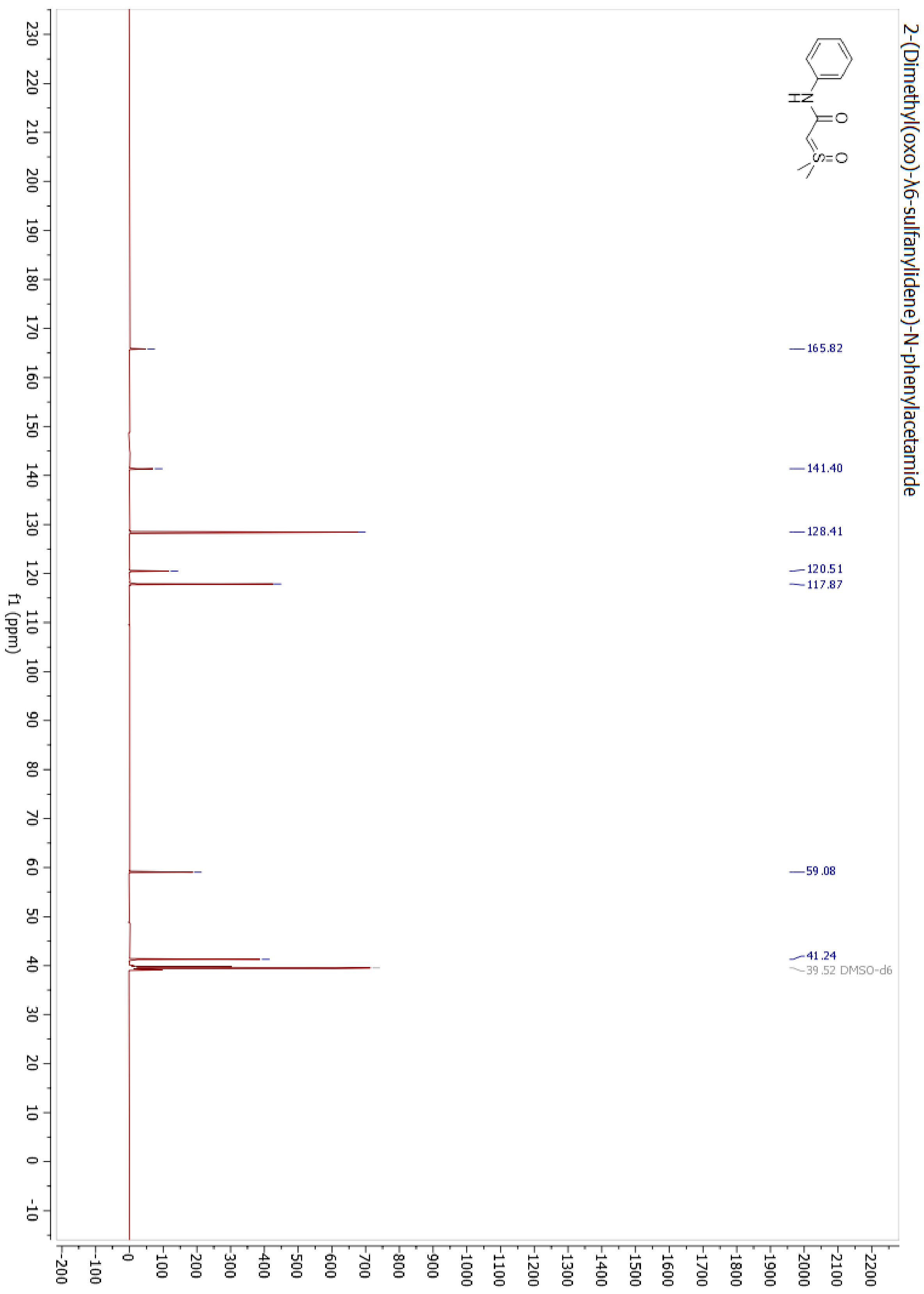

Figure S26. ^{13}C NMR spectrum of **1m** in d_6-DMSO (151 MHz)

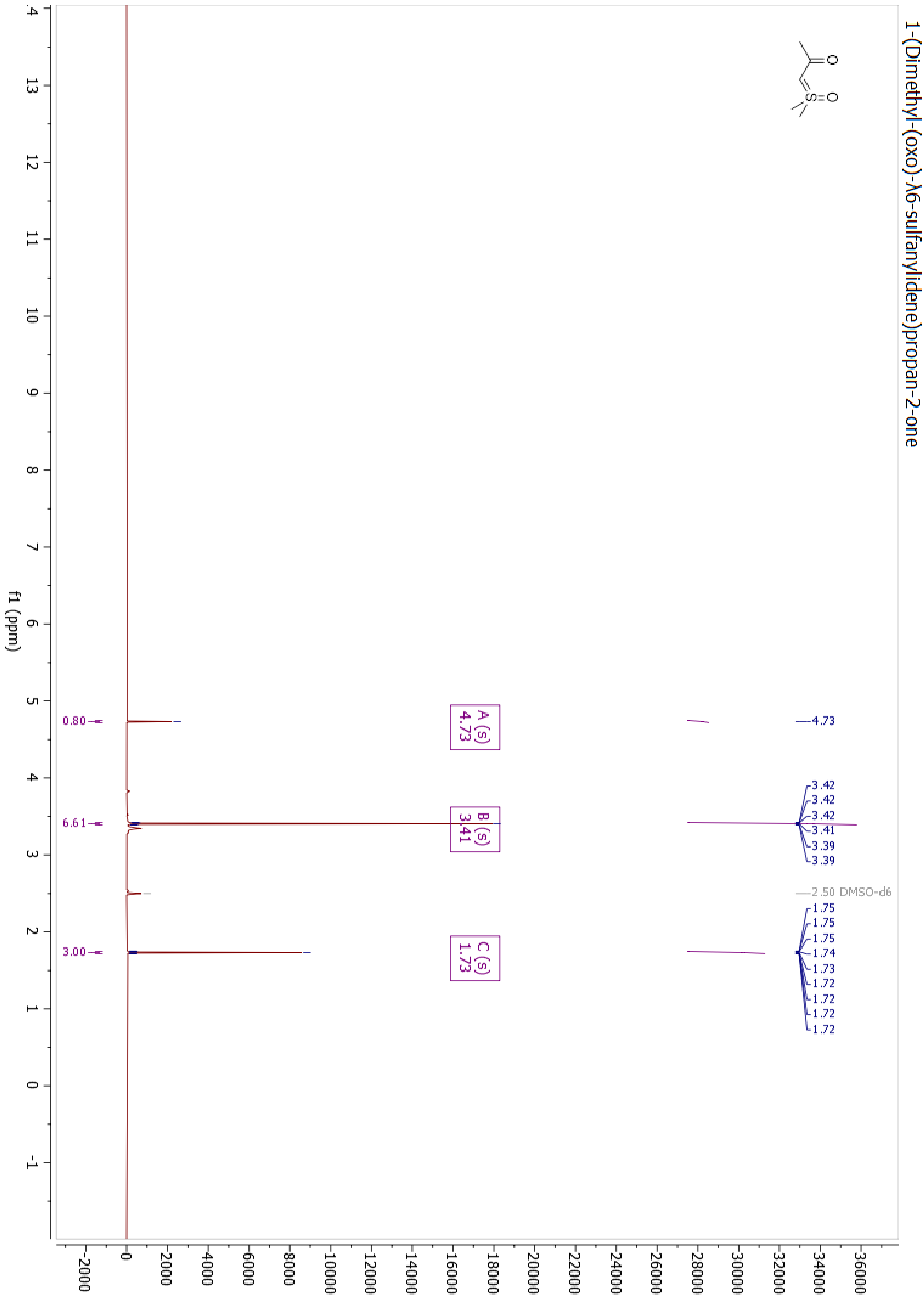

Figure S27. ^{1}H NMR spectrum of **1n** in d_6-DMSO (600 MHz)

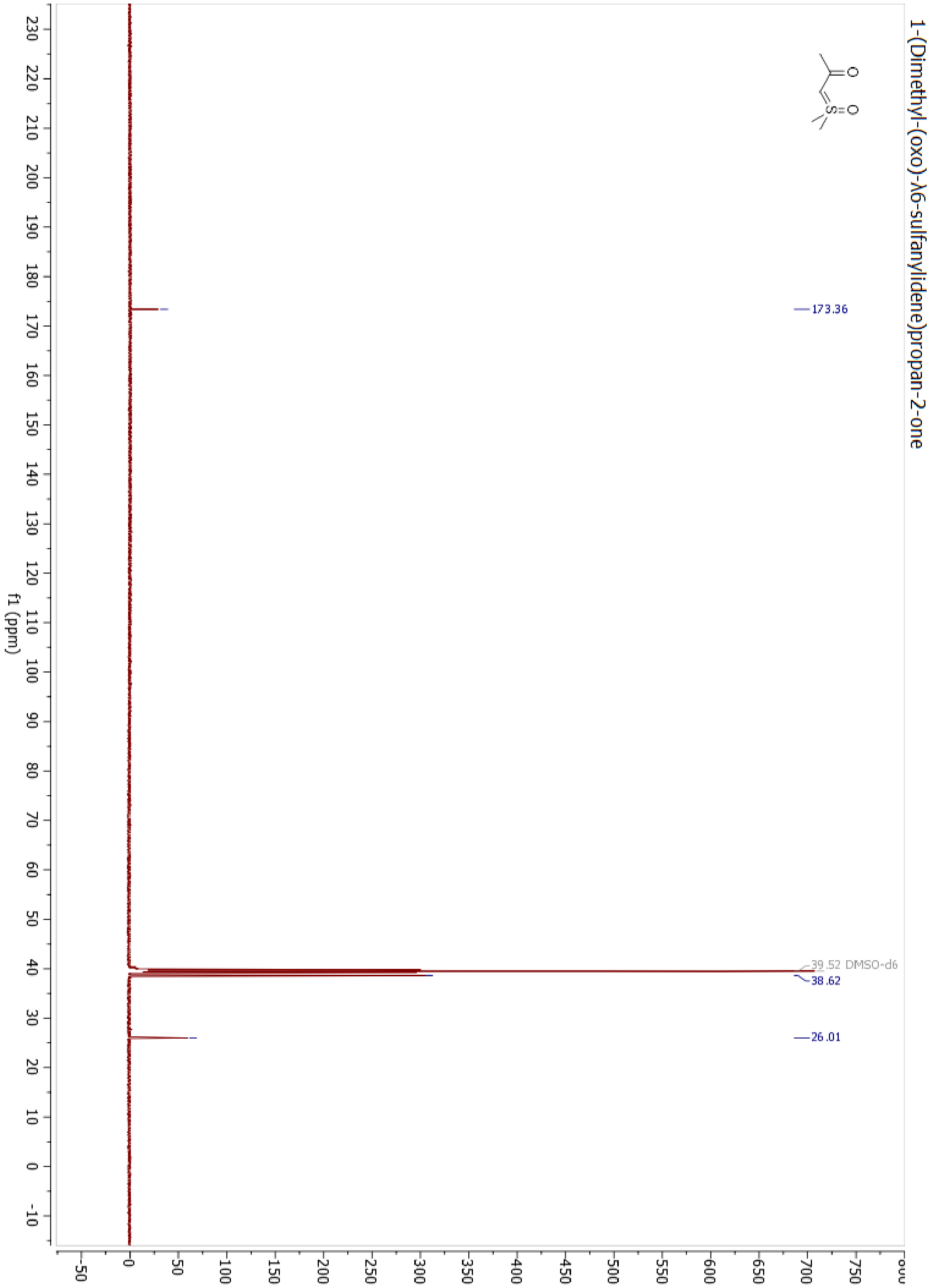

Figure S28. ^{13}C NMR spectrum of **1n** in d_6-DMSO (151 MHz)

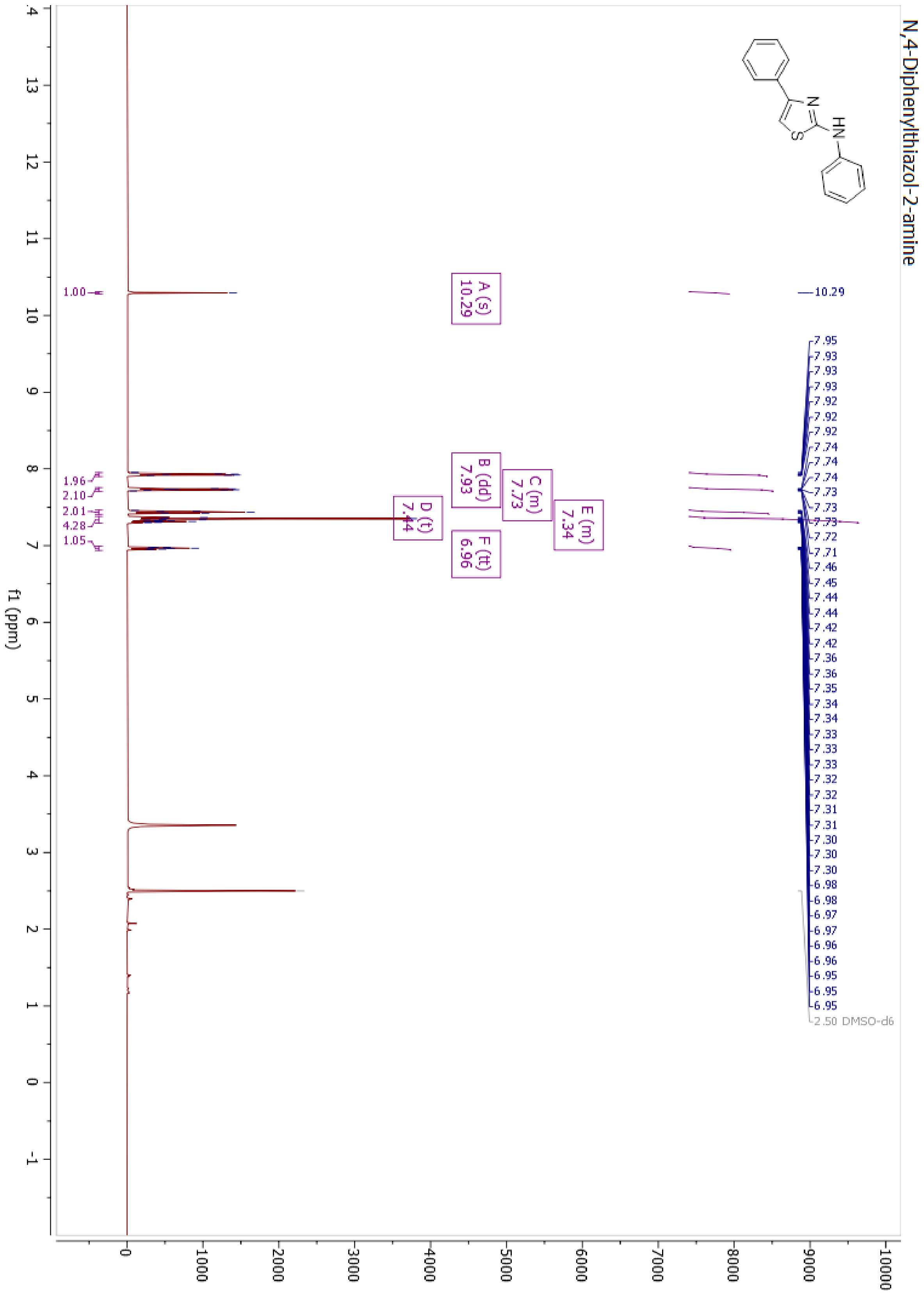

Figure S29. ^{1}H NMR spectrum of **3a** in d_6-DMSO (600 MHz)

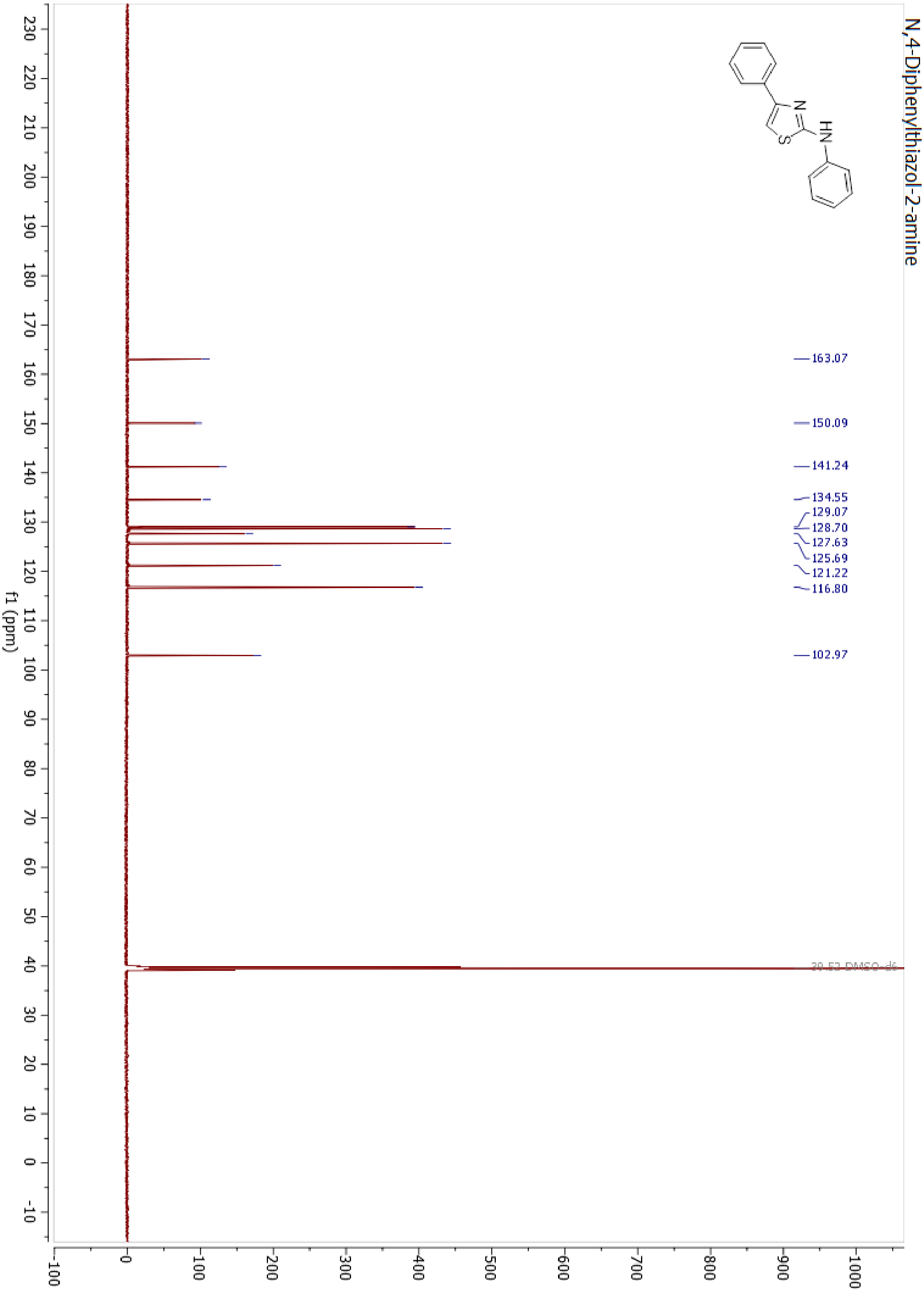

Figure S30. ^{13}C NMR spectrum of **3a** in d_6-DMSO (151 MHz)

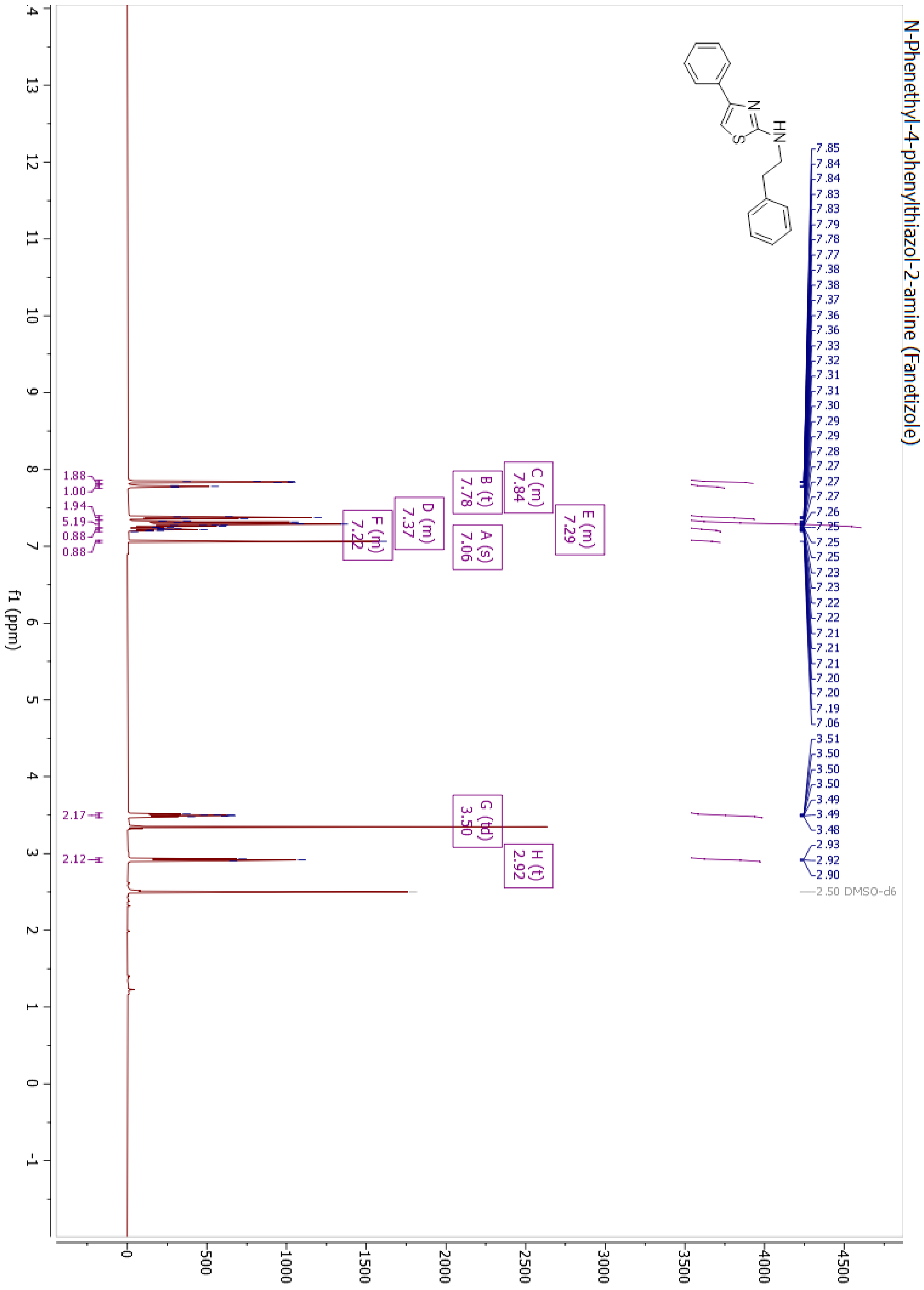

Figure S31. ^{1}H NMR spectrum of **3b** in d_6-DMSO (600 MHz)

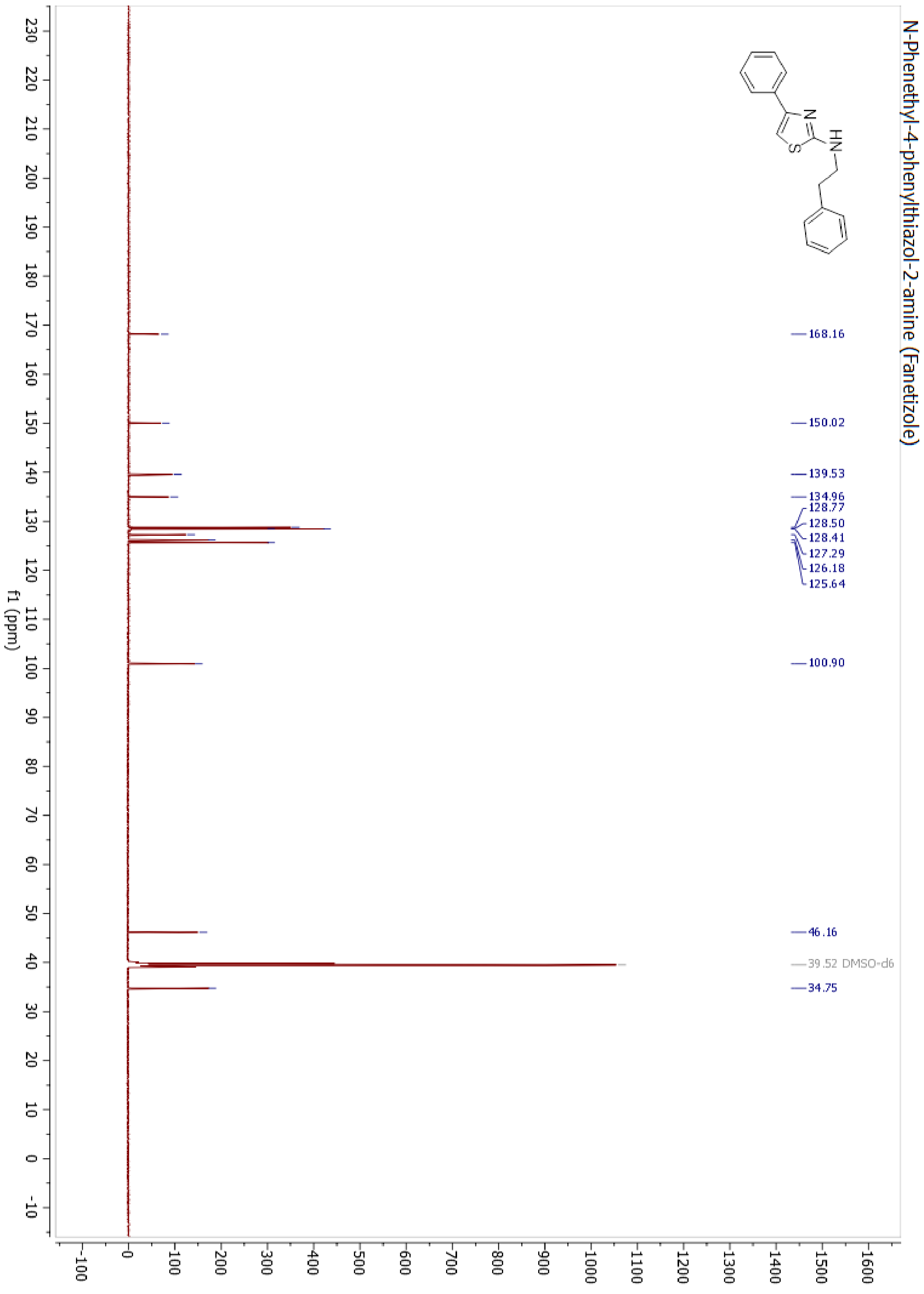

Figure S32. ^{13}C NMR spectrum of **3b** in d_6-DMSO (151 MHz)

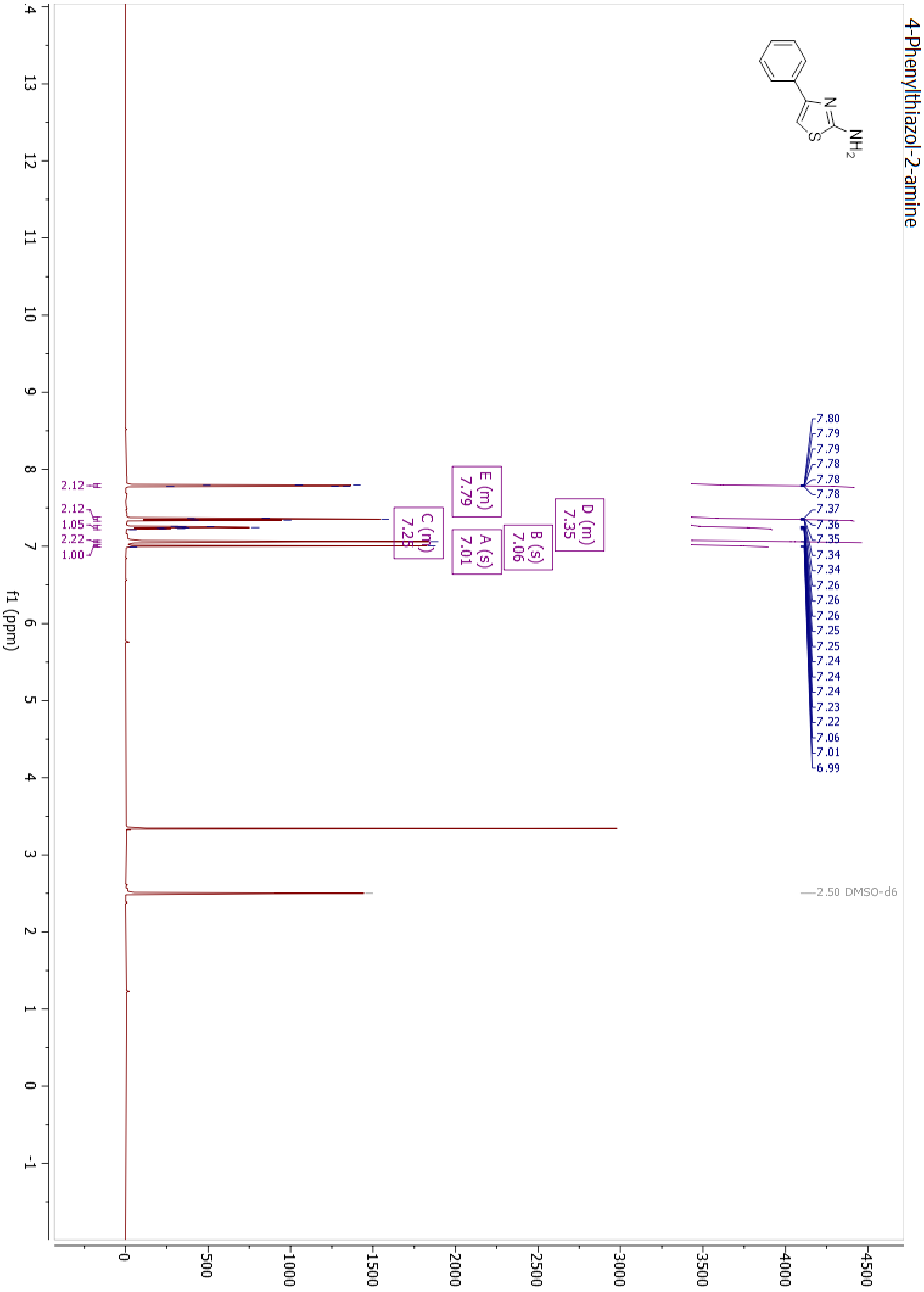

Figure S33. ^{13}C NMR spectrum of **3b** in d_6-DMSO (151 MHz)

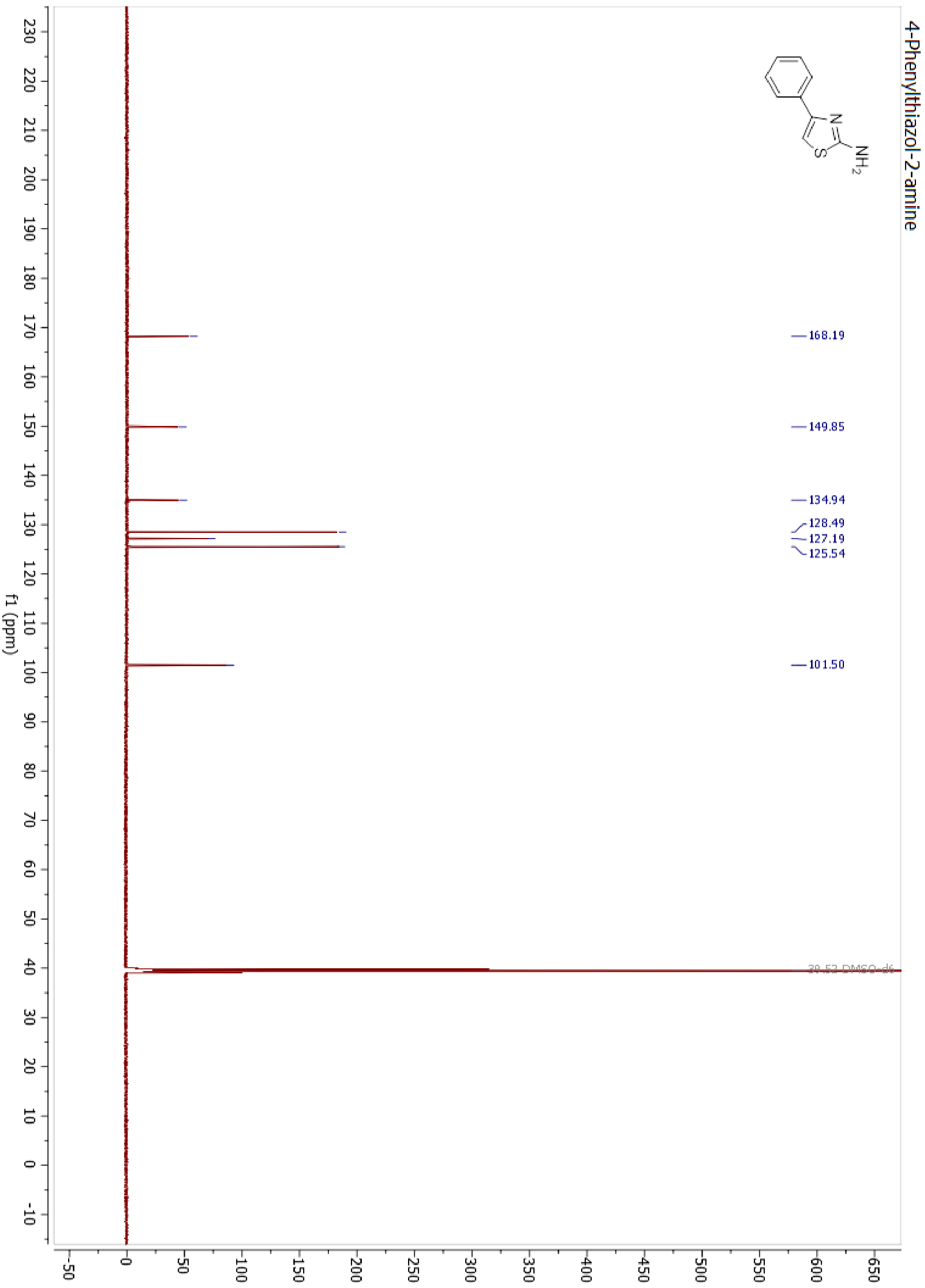

Figure S34. ^{13}C NMR spectrum of **3c** in d_6-DMSO (151 MHz)

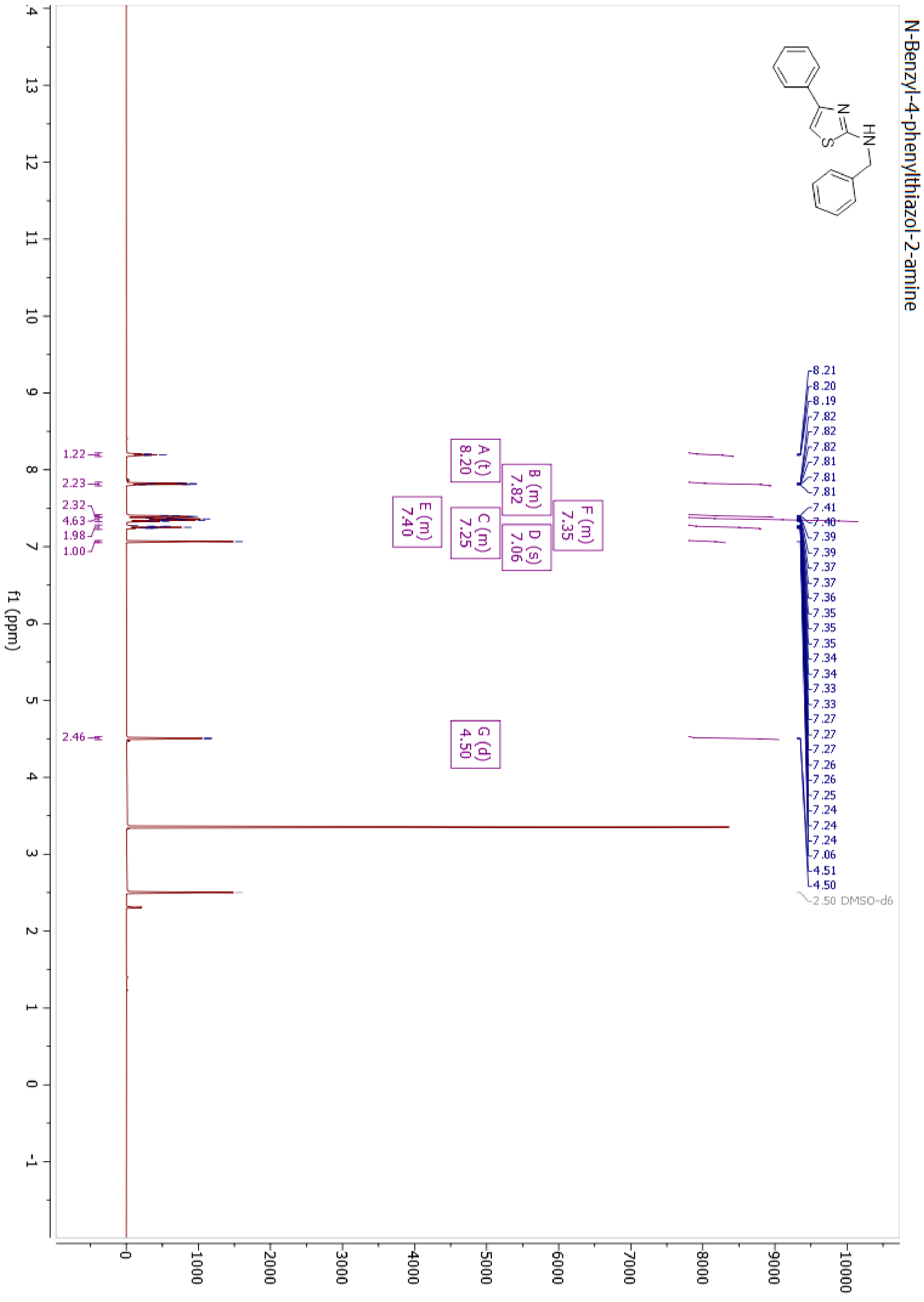

Figure S35. ^{1}H NMR spectrum of **3d** in d_6-DMSO (600 MHz)

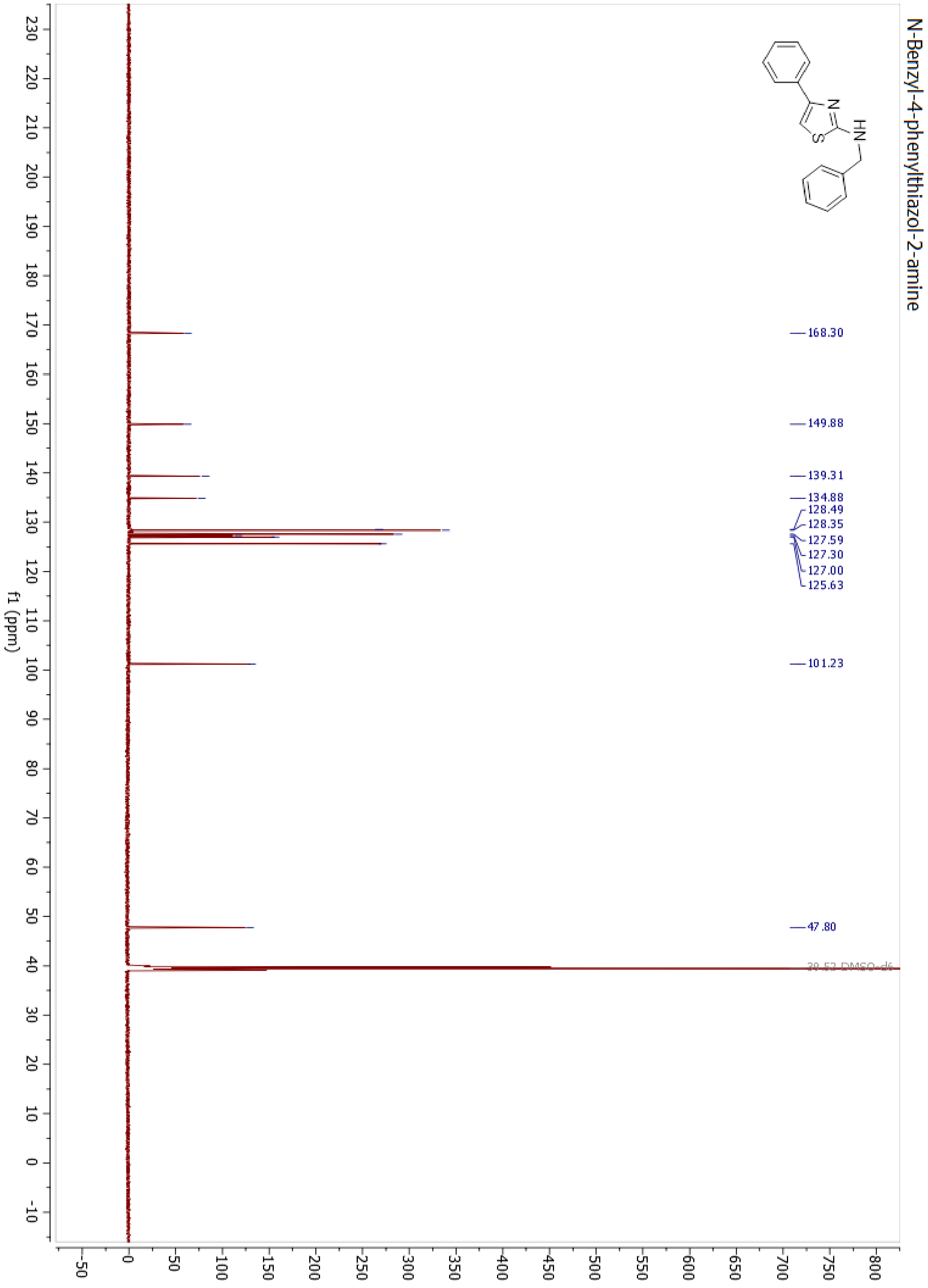

Figure S36. ^{13}C NMR spectrum of **3d** in d_6-DMSO (151 MHz)

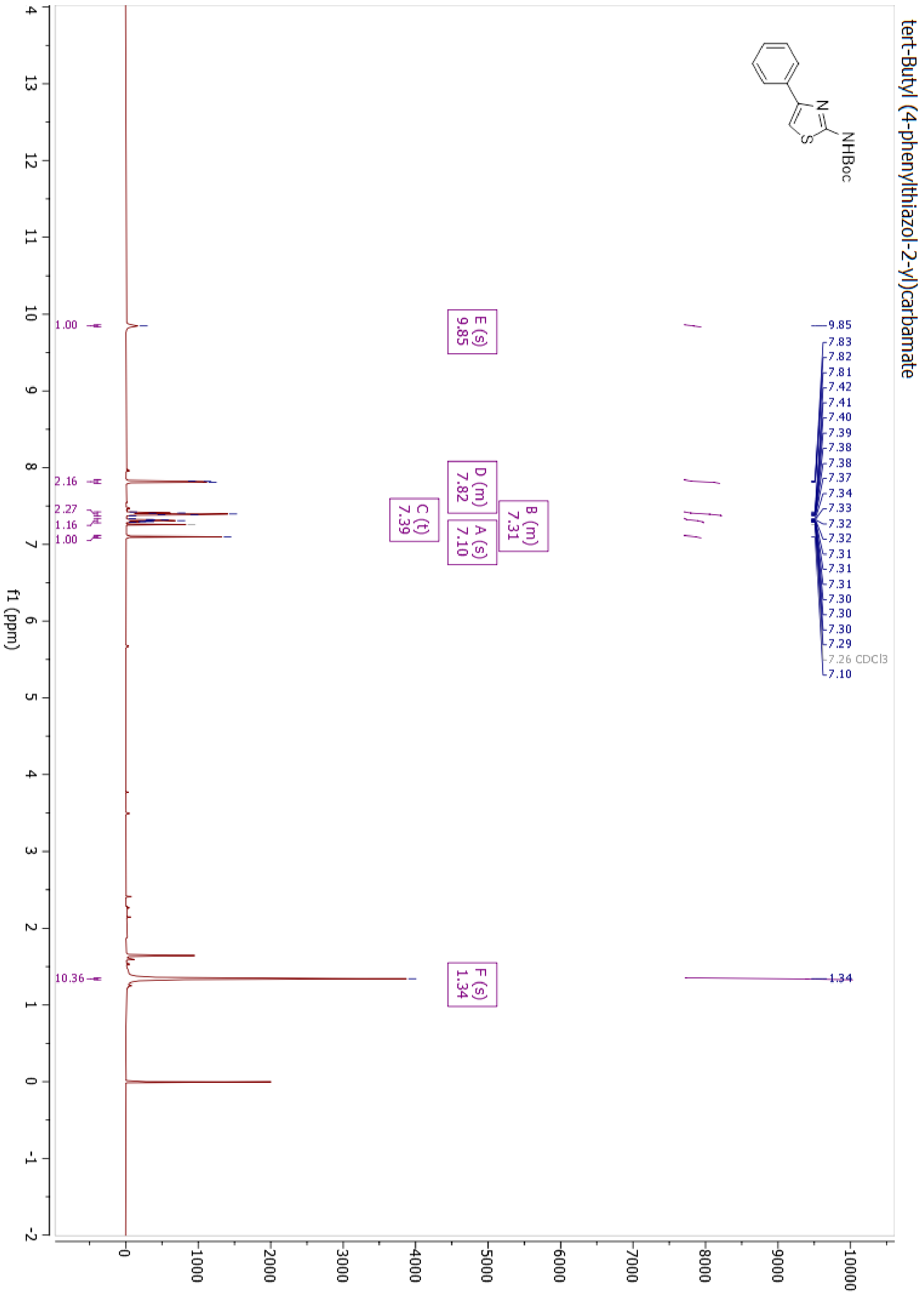

Figure S37. ^{1}H NMR spectrum of **3e** in d_6-DMSO (600 MHz)

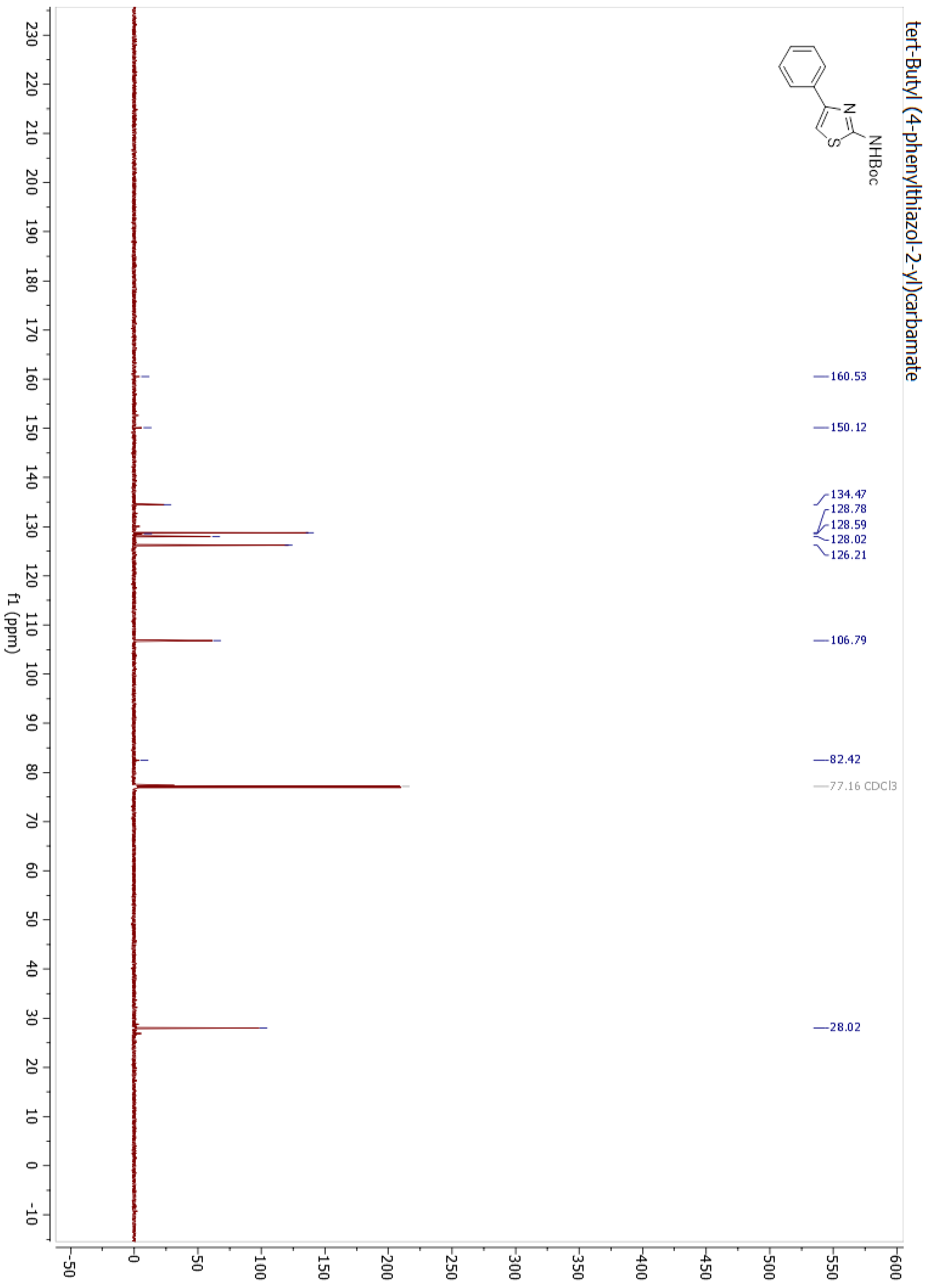

Figure S38. ^{13}C NMR spectrum of **3e** in d_6-DMSO (151 MHz)

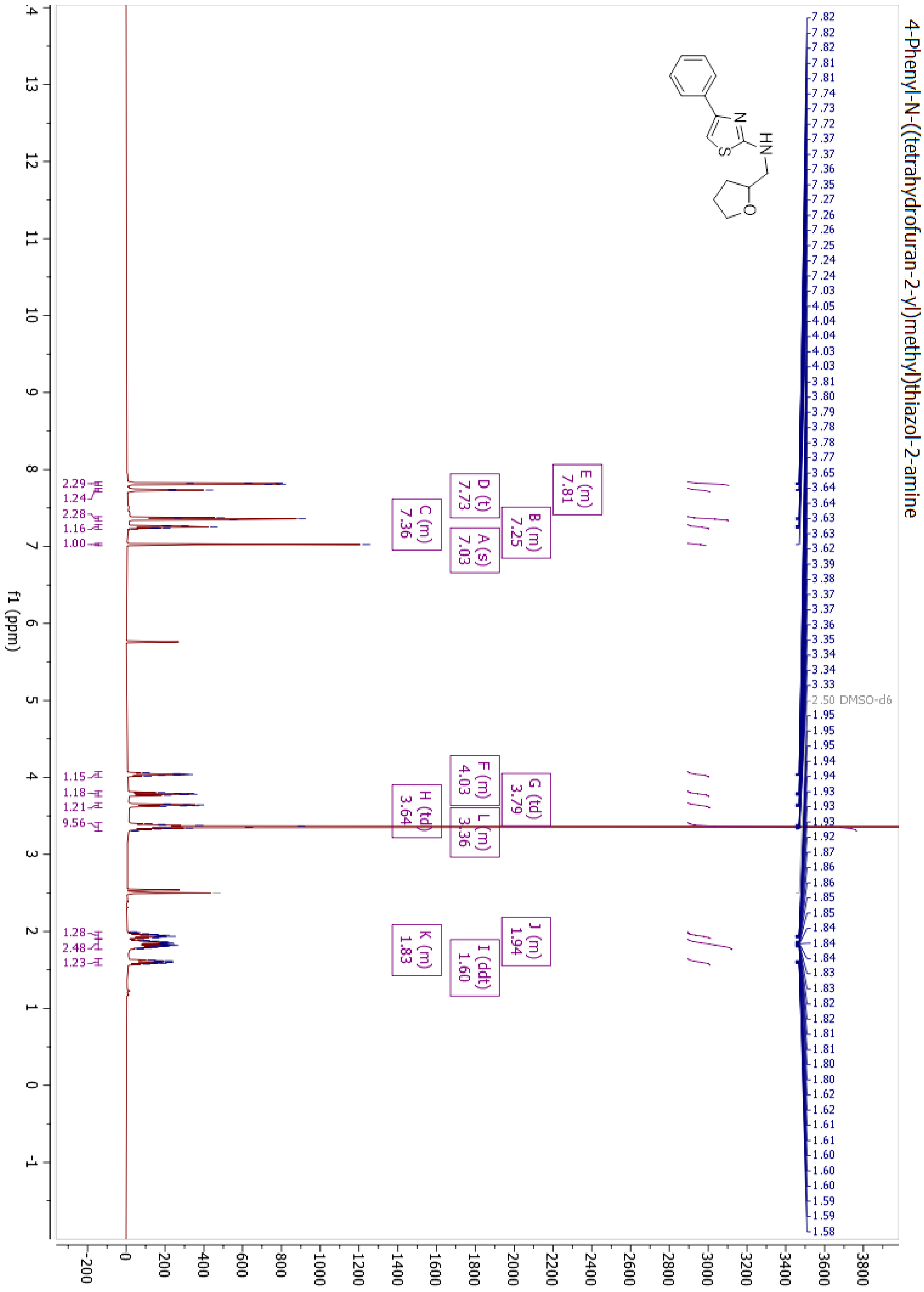

Figure S39. ^{1}H NMR spectrum of **3f** in d_6-DMSO (600 MHz)

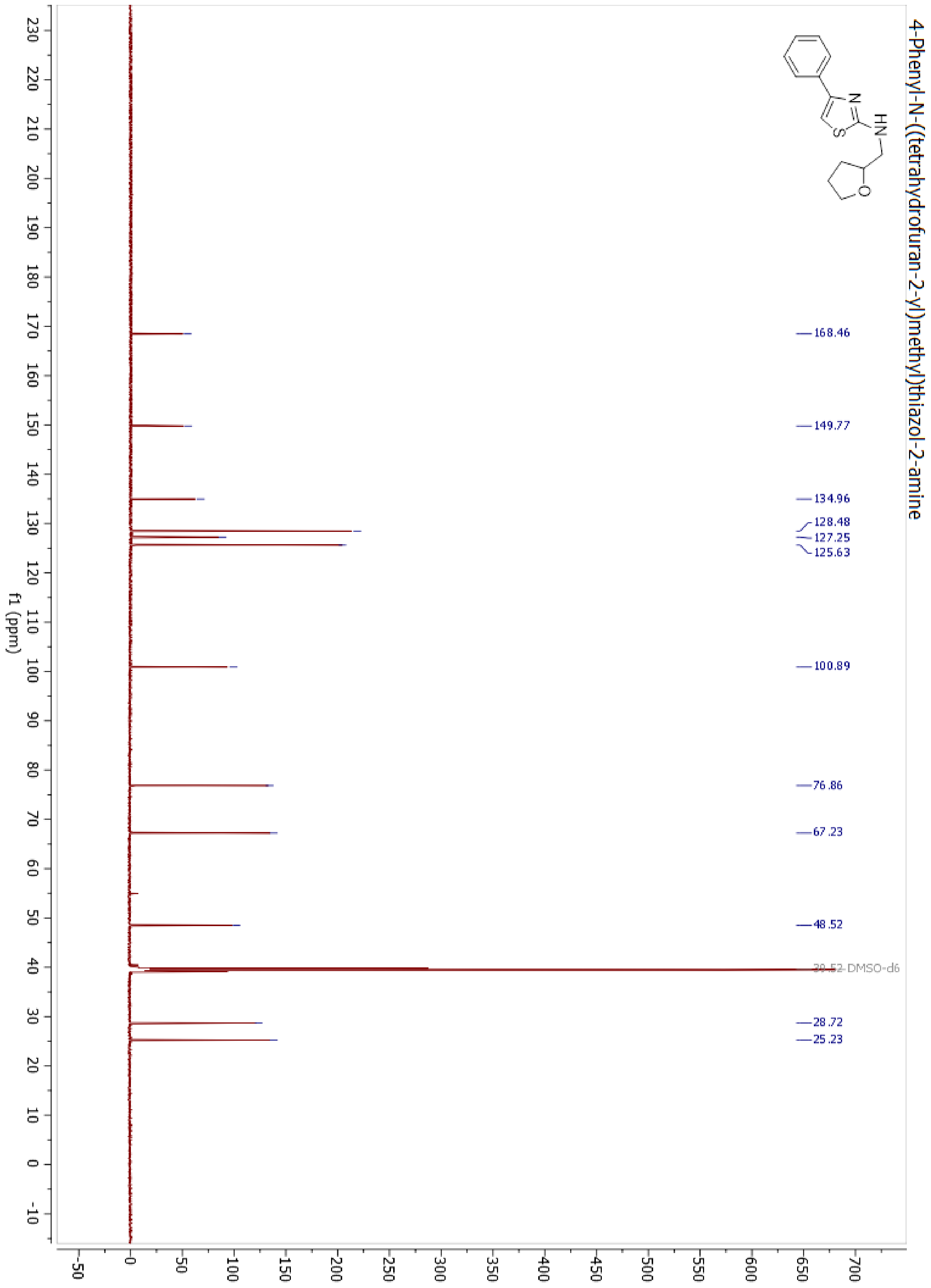

Figure S40. ^{13}C NMR spectrum of **3f** in d_6-DMSO (151 MHz)

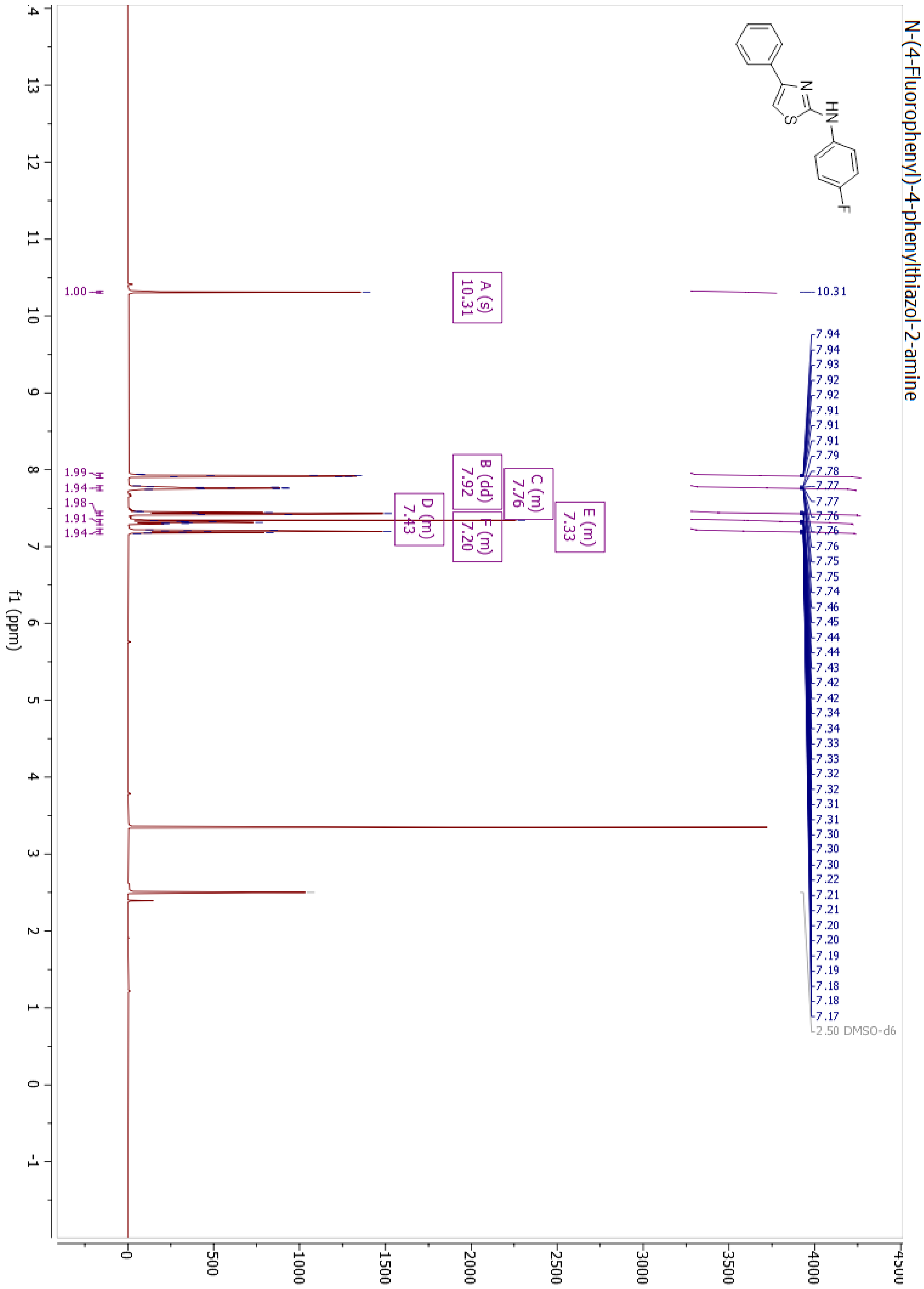

Figure S41. ^{1}H NMR spectrum of **3g** in d_6-DMSO (600 MHz)

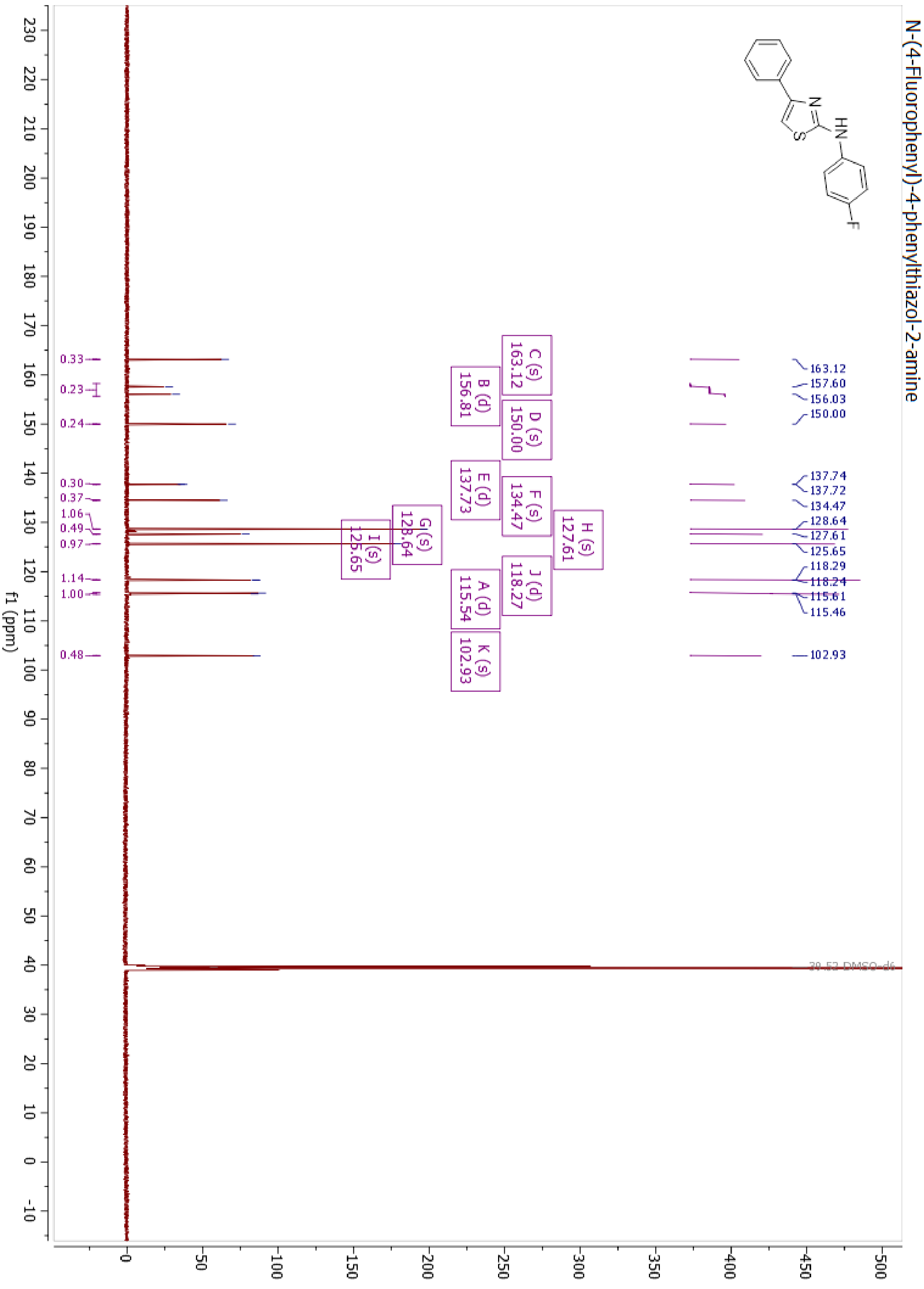

Figure S42. ^{13}C NMR spectrum of **3g** in d_6-DMSO (151 MHz)

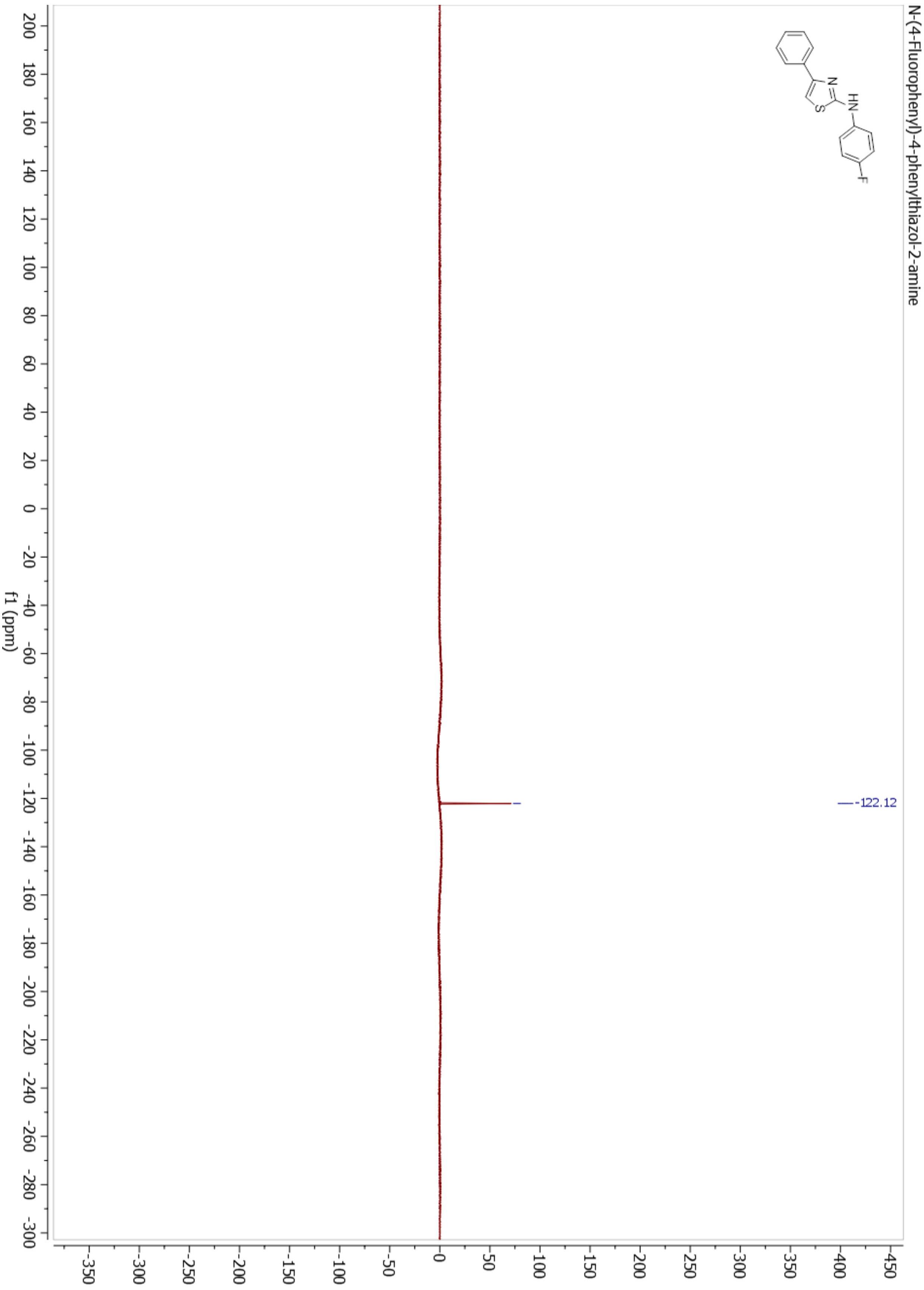

Figure S43. ^{19}F NMR spectrum of **3g** in *d*$_6$-DMSO (376 MHz)

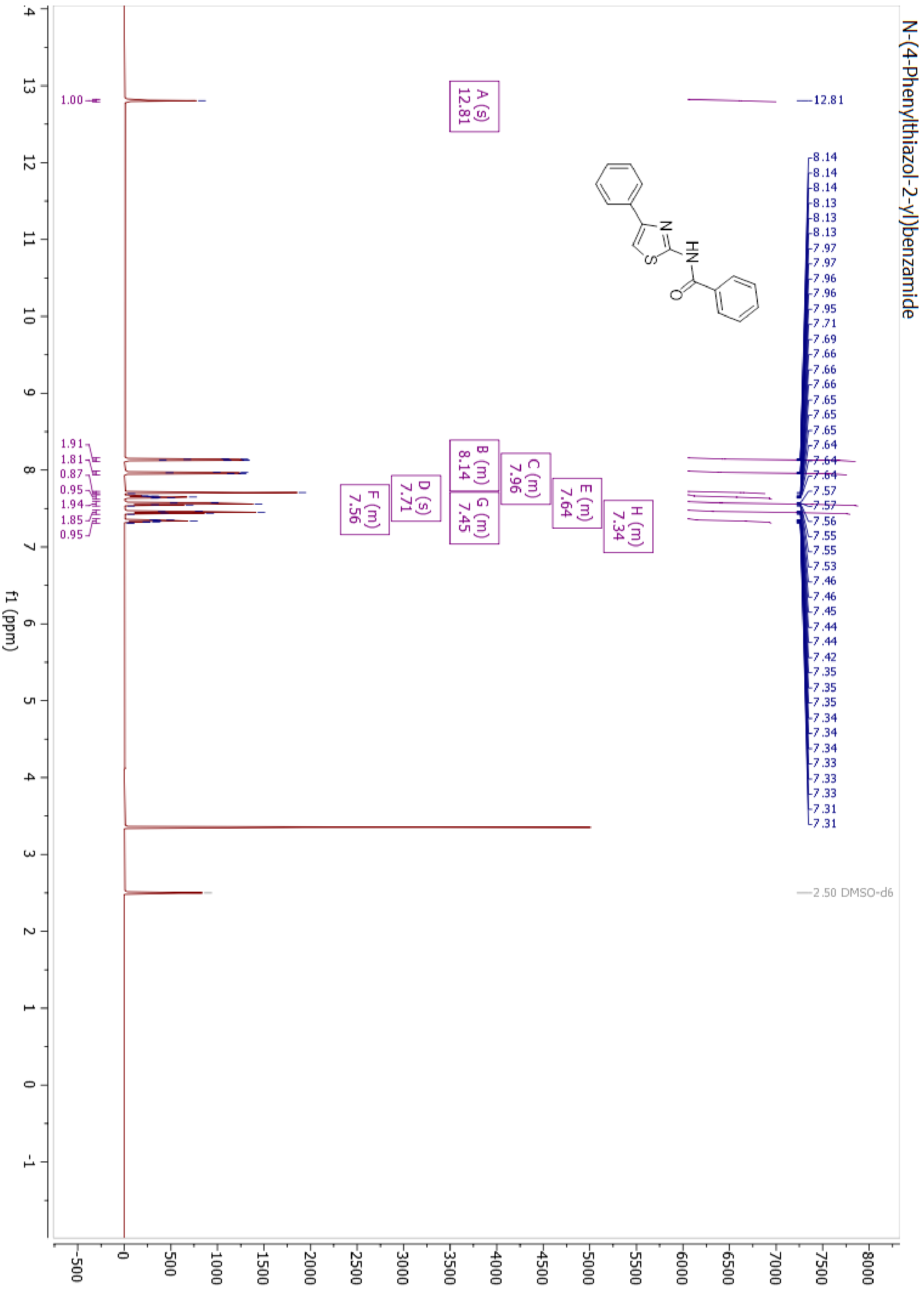

Figure S44. ^{1}H NMR spectrum of **3h** in d_6-DMSO (600 MHz)

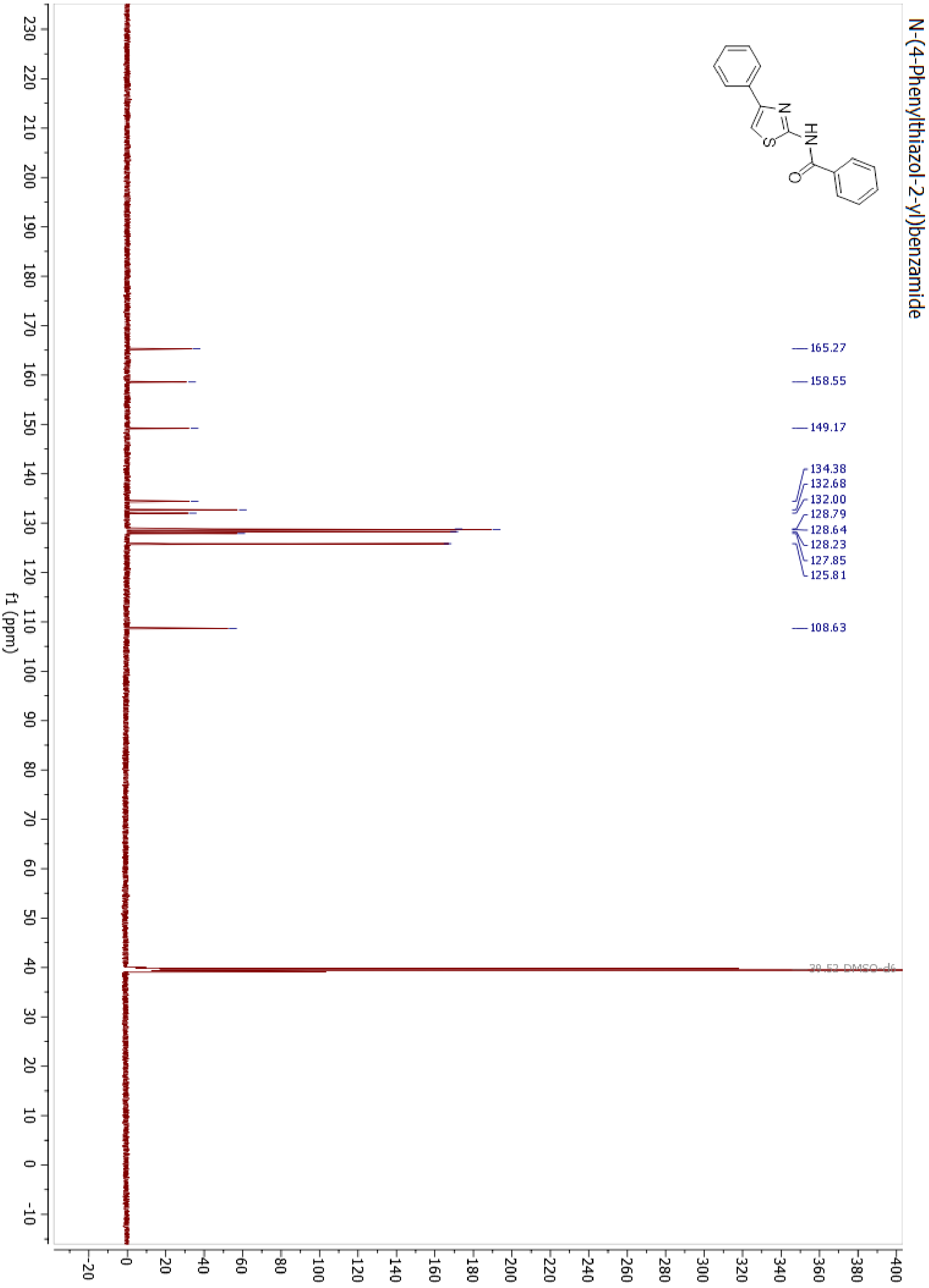

Figure S45. ^{13}C NMR spectrum of **3h** in d_6-DMSO (151 MHz)

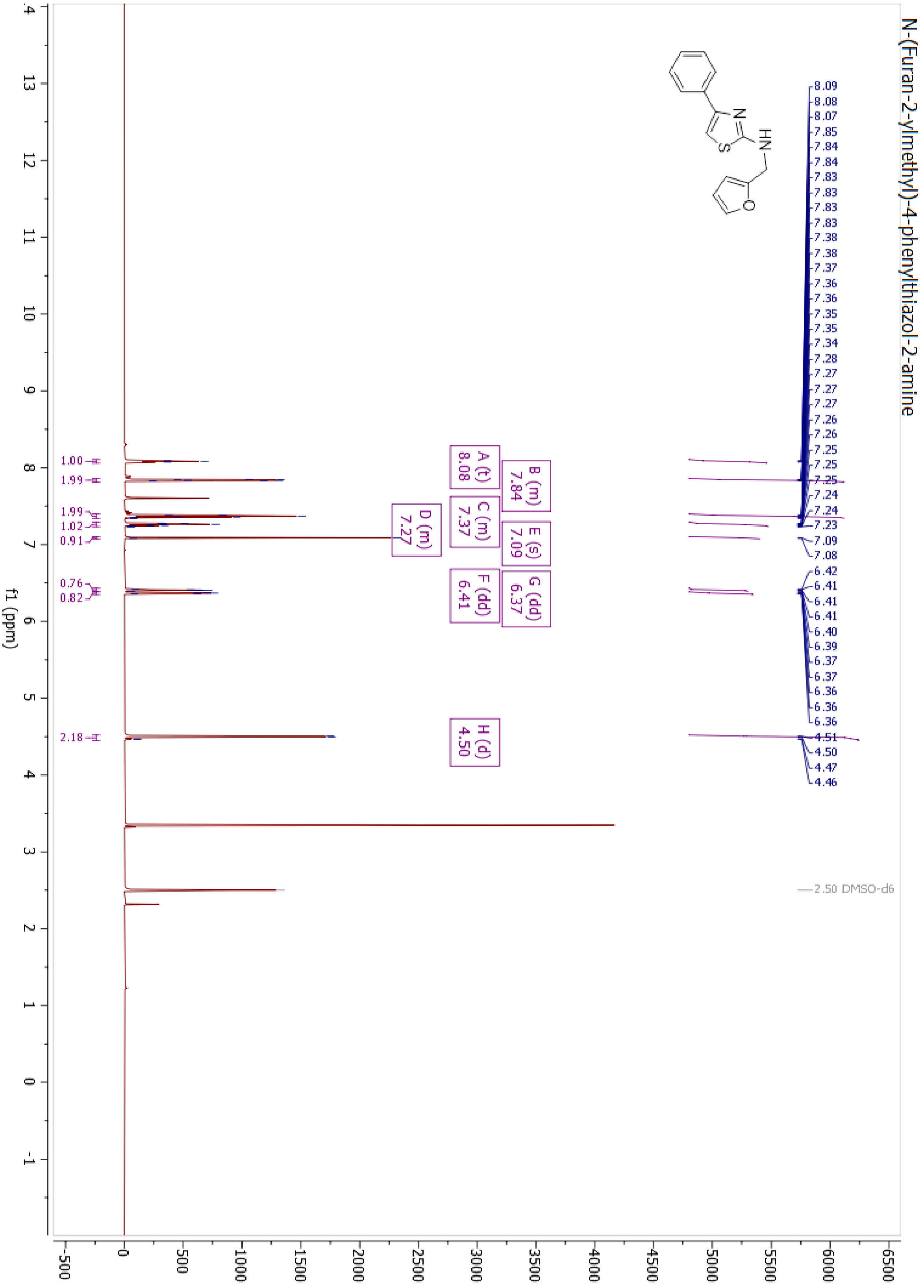

Figure S46. ^{1}H NMR spectrum of **3i** in d_6-DMSO (600 MHz)

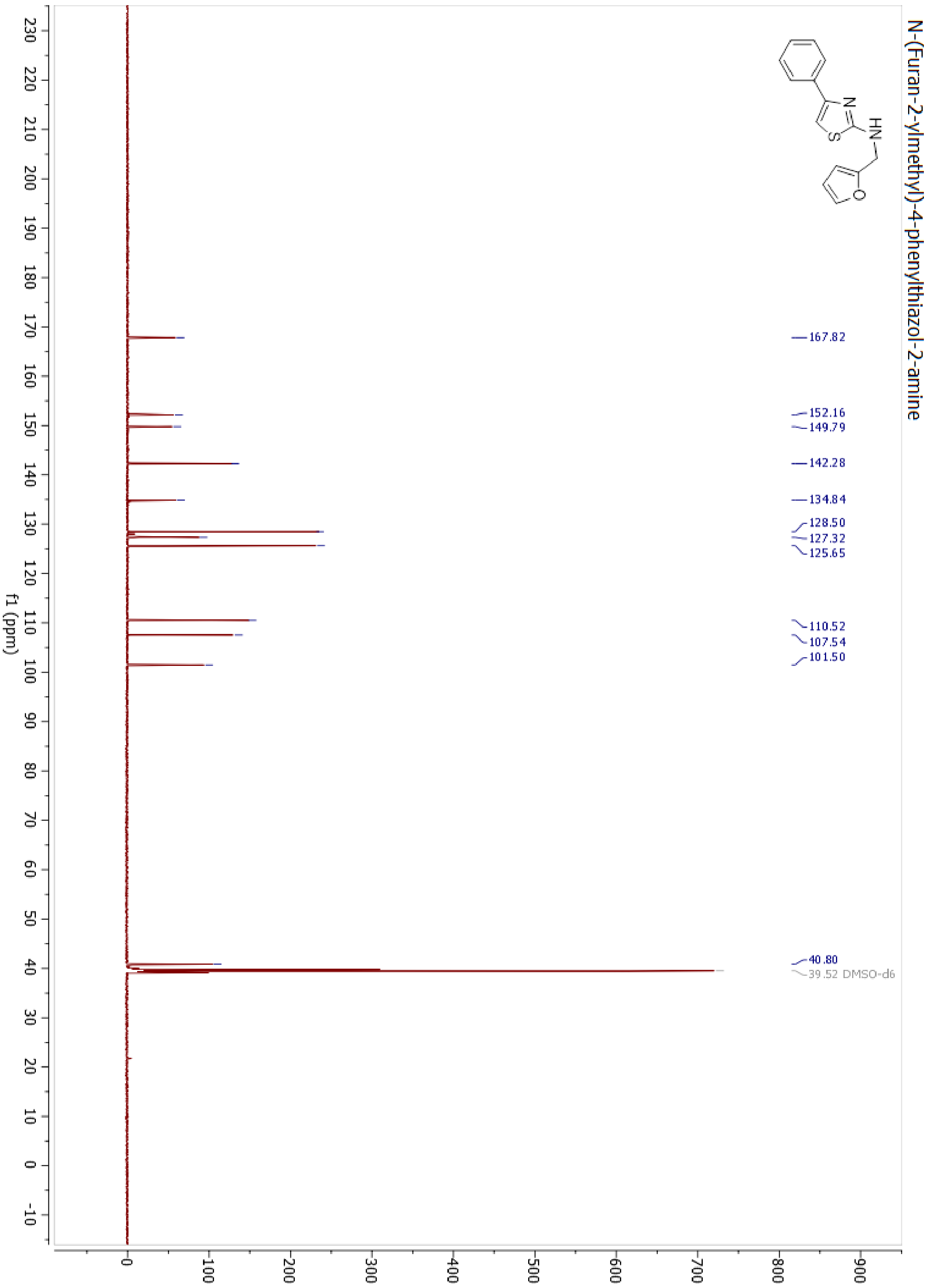

Figure S47. ^{13}C NMR spectrum of **3i** in d_6-DMSO (151 MHz)

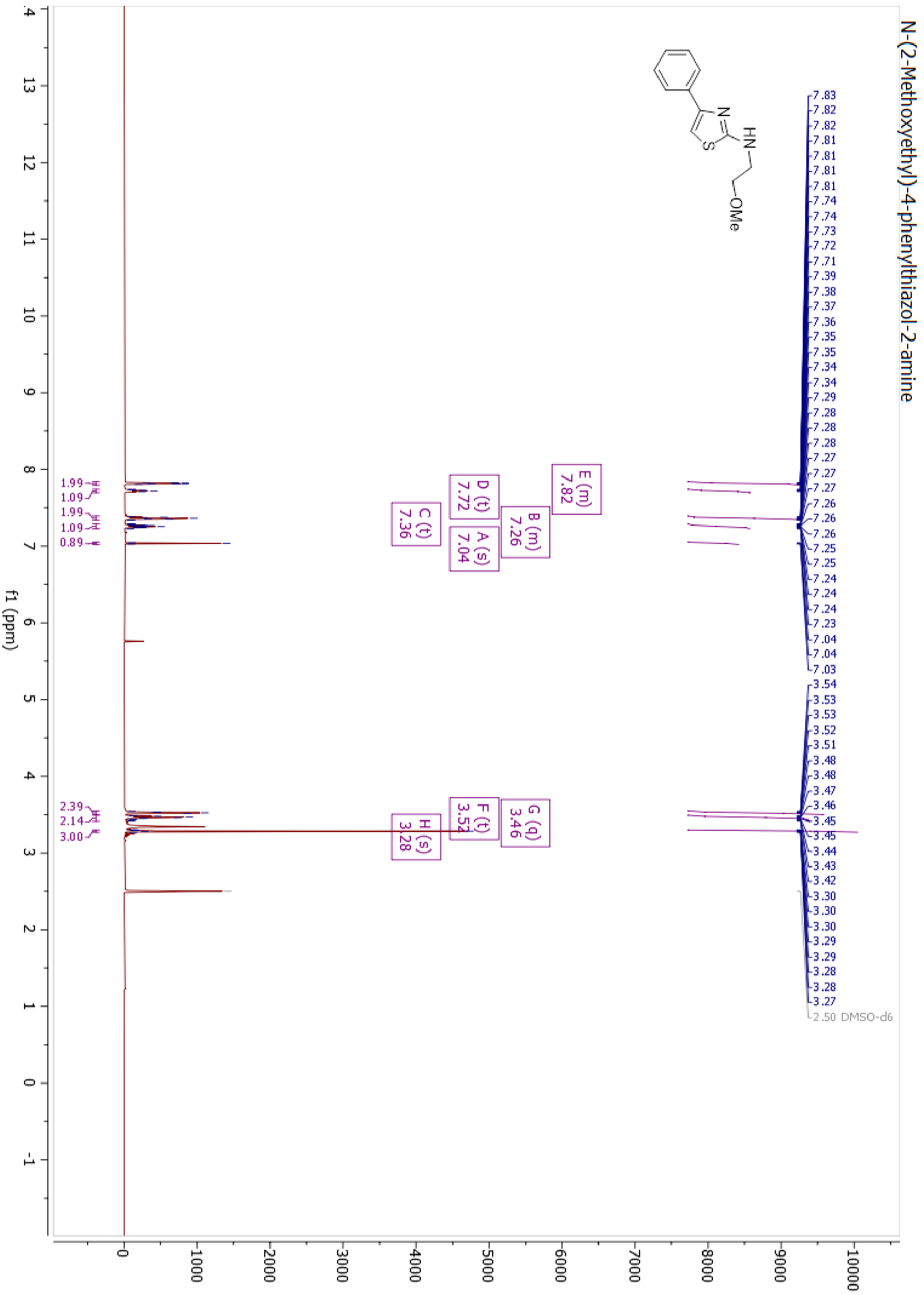

Figure S48. ^{1}H NMR spectrum of **3j** in d_6-DMSO (600 MHz)

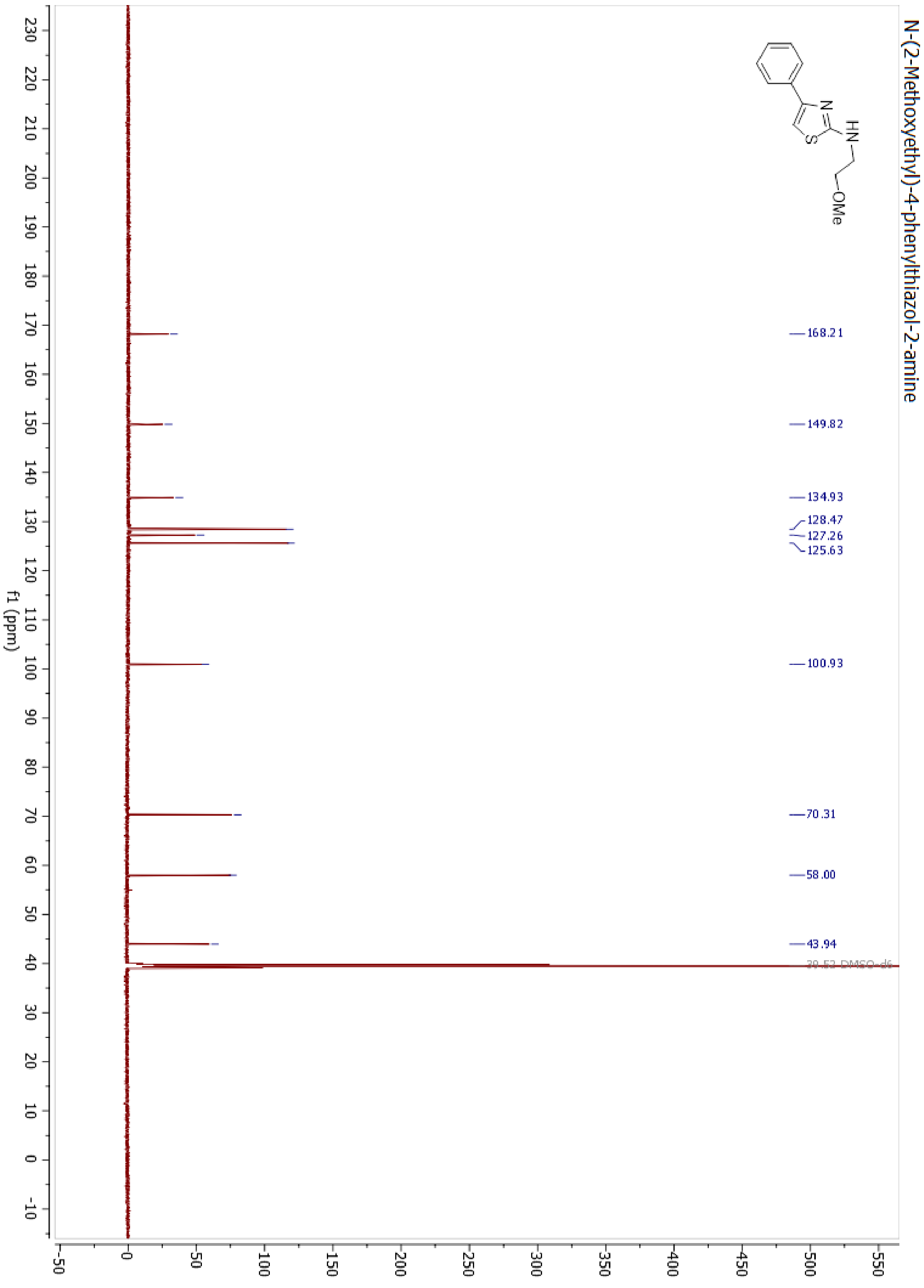

Figure S49. ^{13}C NMR spectrum of **3j** in d_6-DMSO (151 MHz)

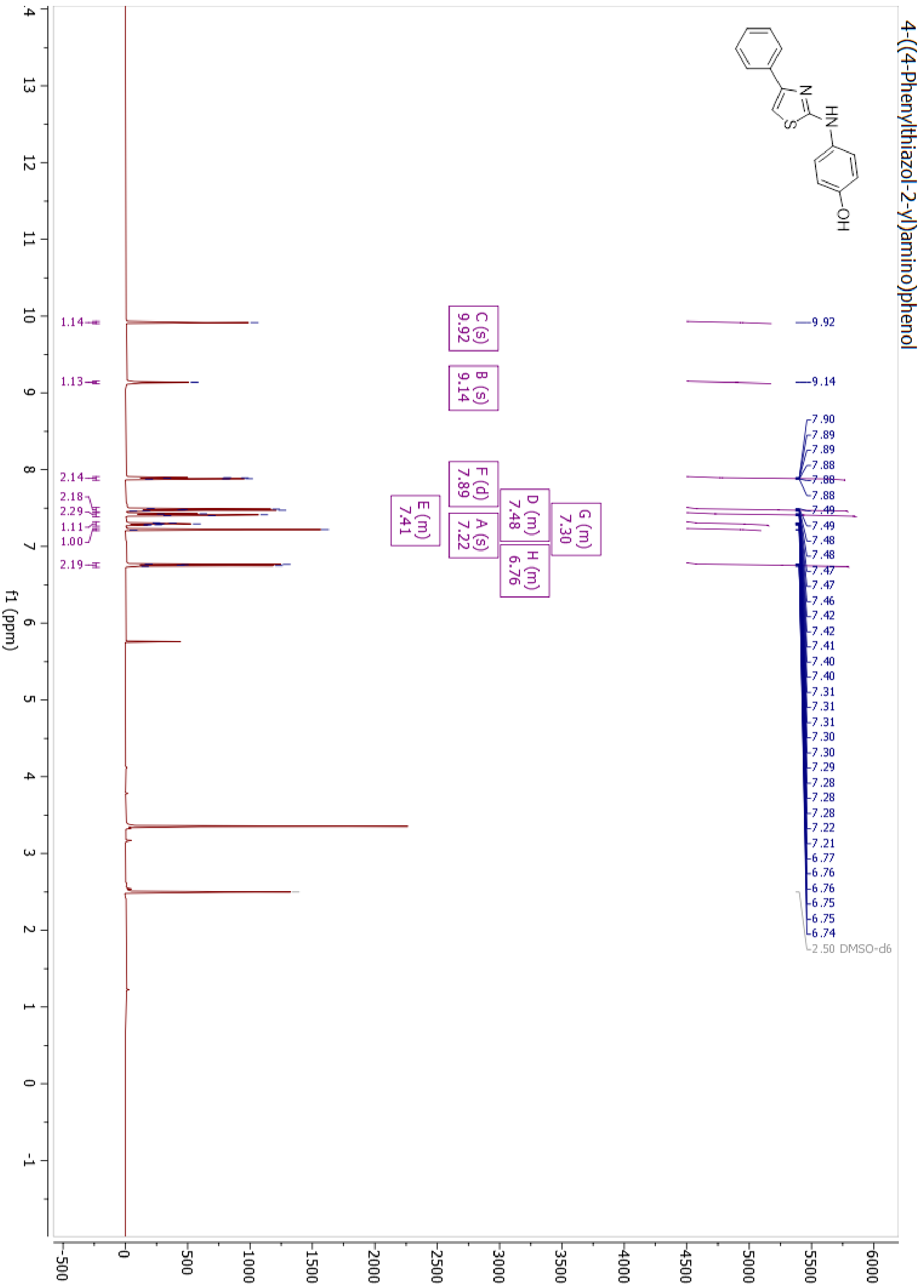

Figure S50. ^{1}H NMR spectrum of **3k** in d_6-DMSO (600 MHz)

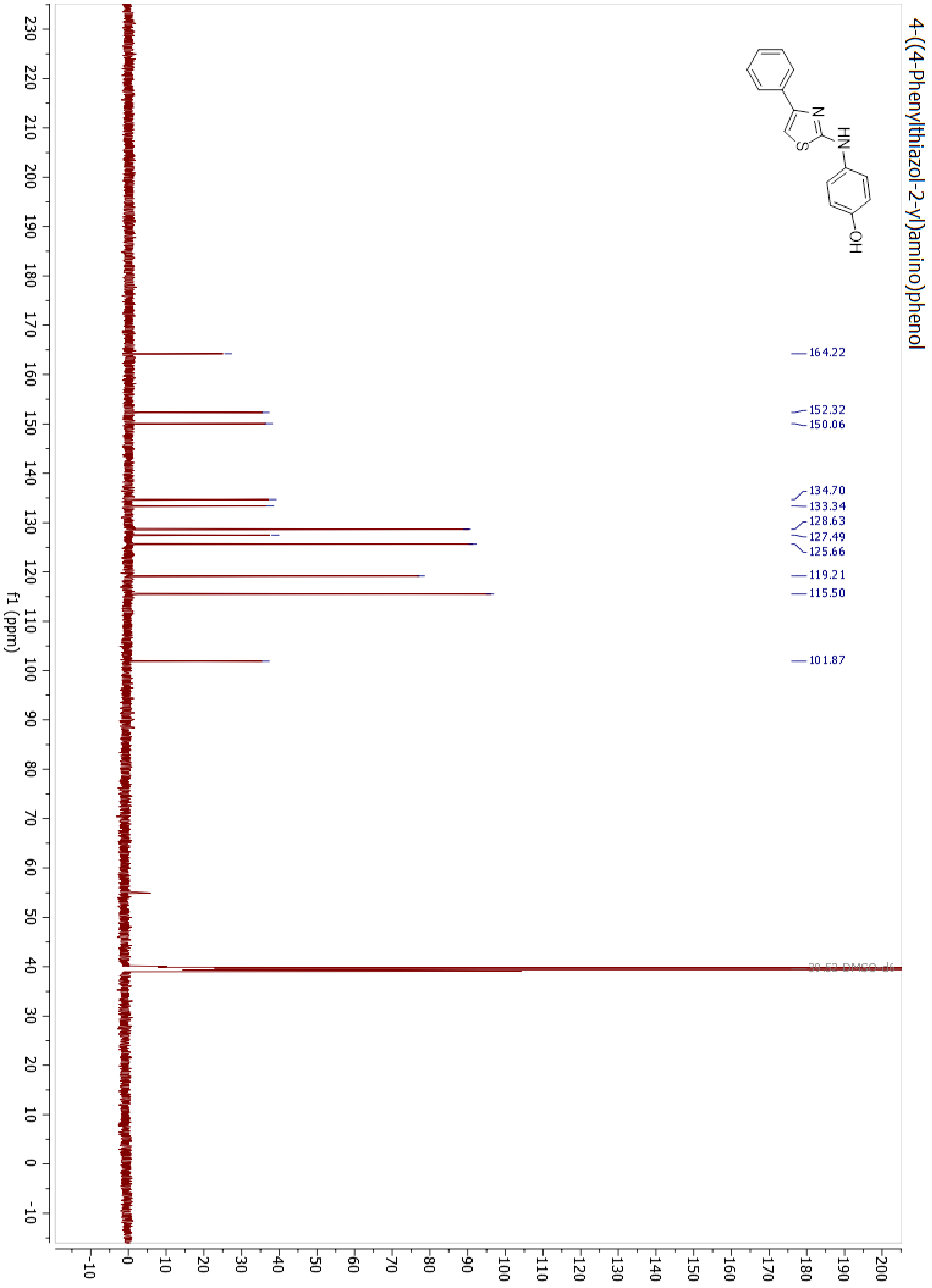

Figure S51. ^{13}C NMR spectrum of **3k** in d_6-DMSO (151 MHz)

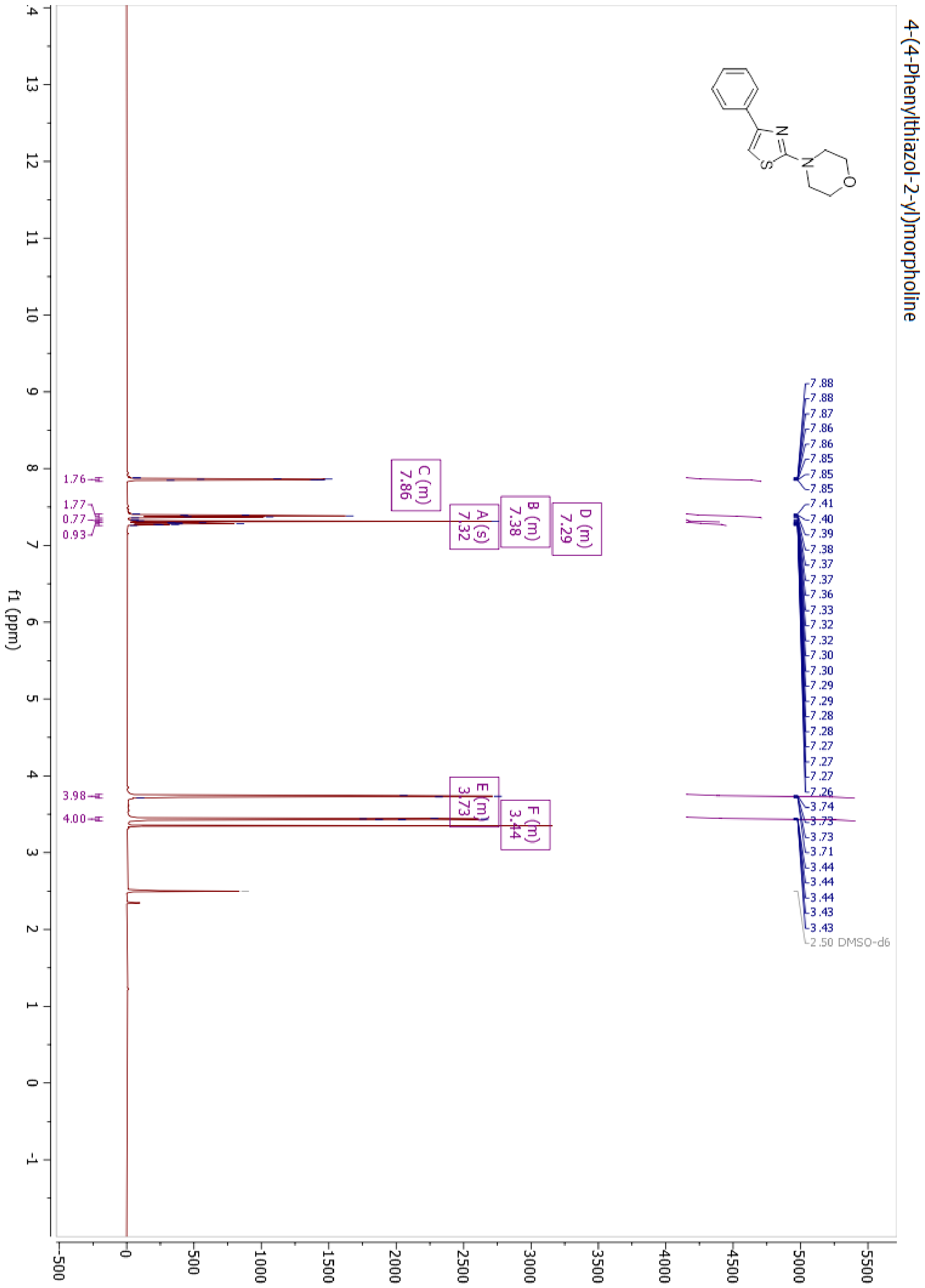

Figure S52. ^{1}H NMR spectrum of **3l** in d_6-DMSO (600 MHz)

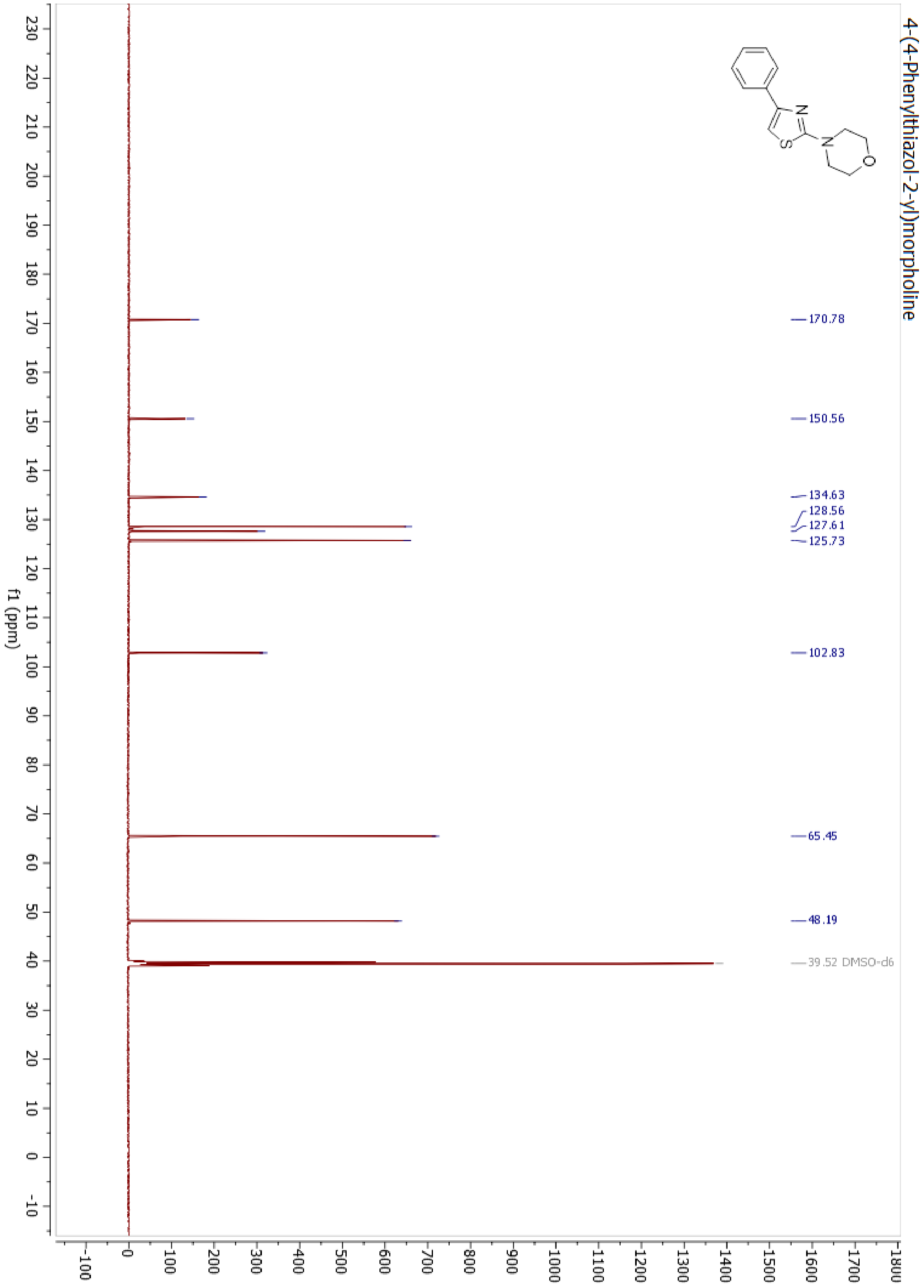

Figure S53. ^{13}C NMR spectrum of **3l** in d_6-DMSO (151 MHz)

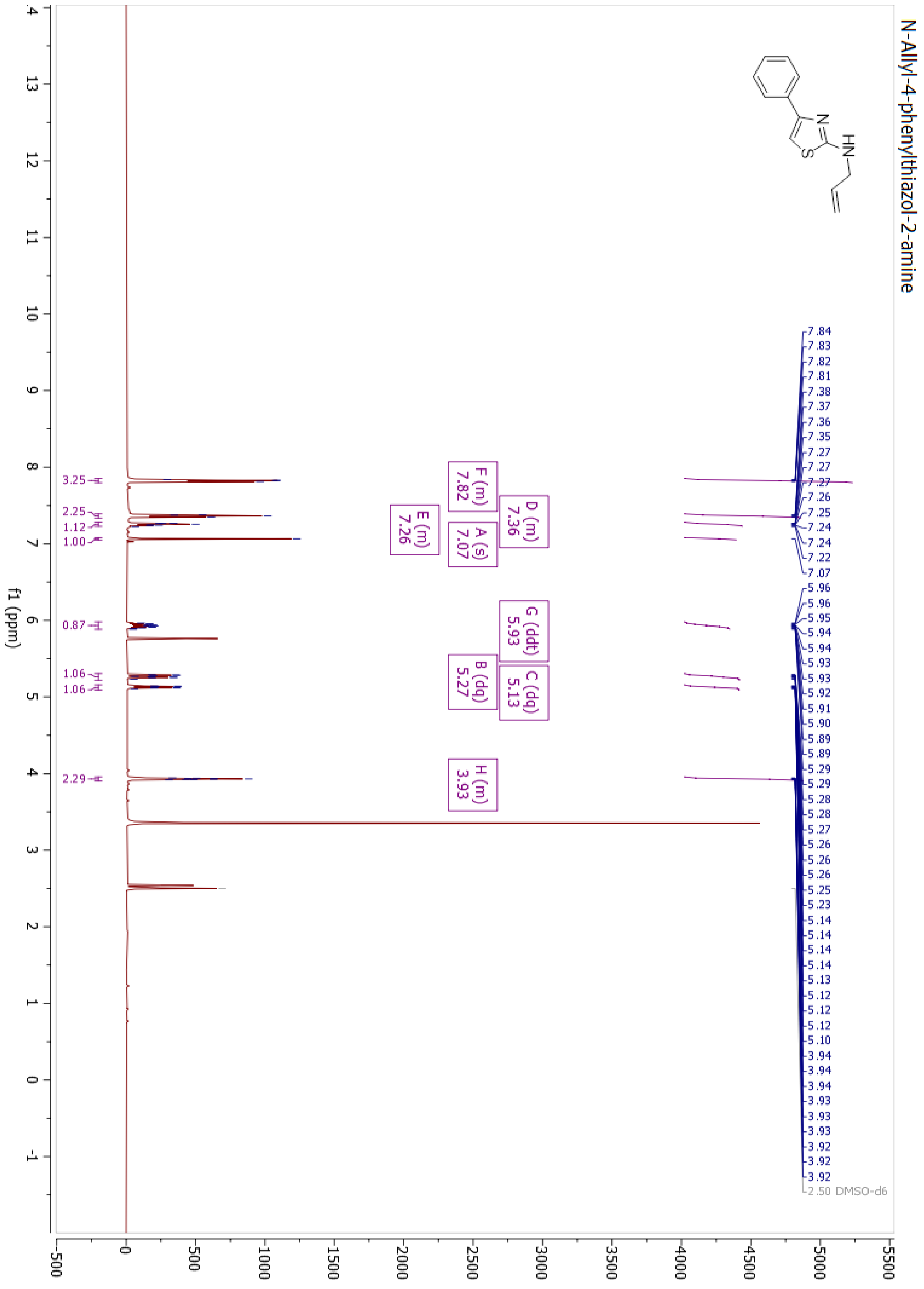

Figure S54. ^{1}H NMR spectrum of **3m** in d_6-DMSO (600 MHz)

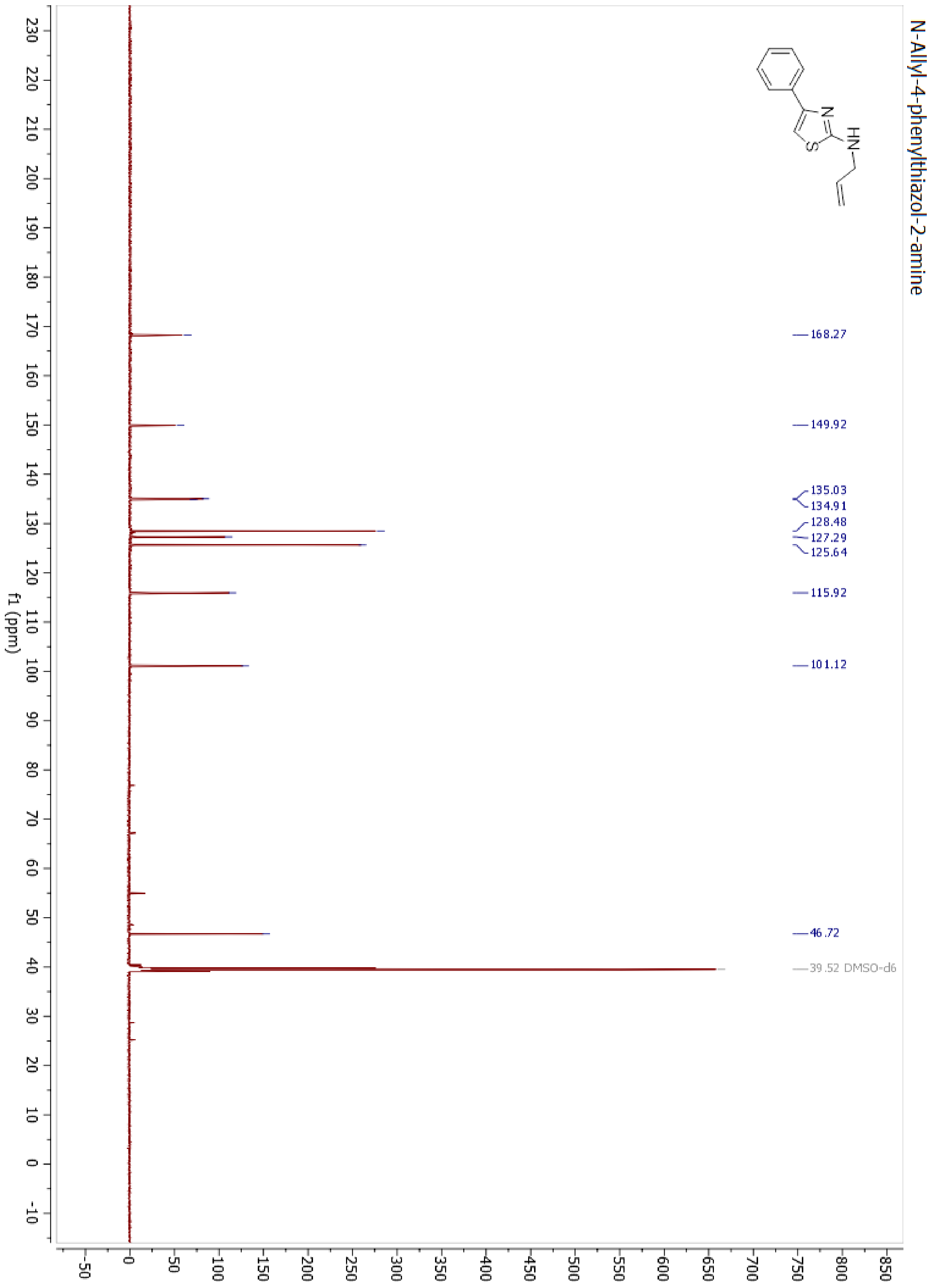

Figure S55. ^{13}C NMR spectrum of **3m** in d_6-DMSO (151 MHz)

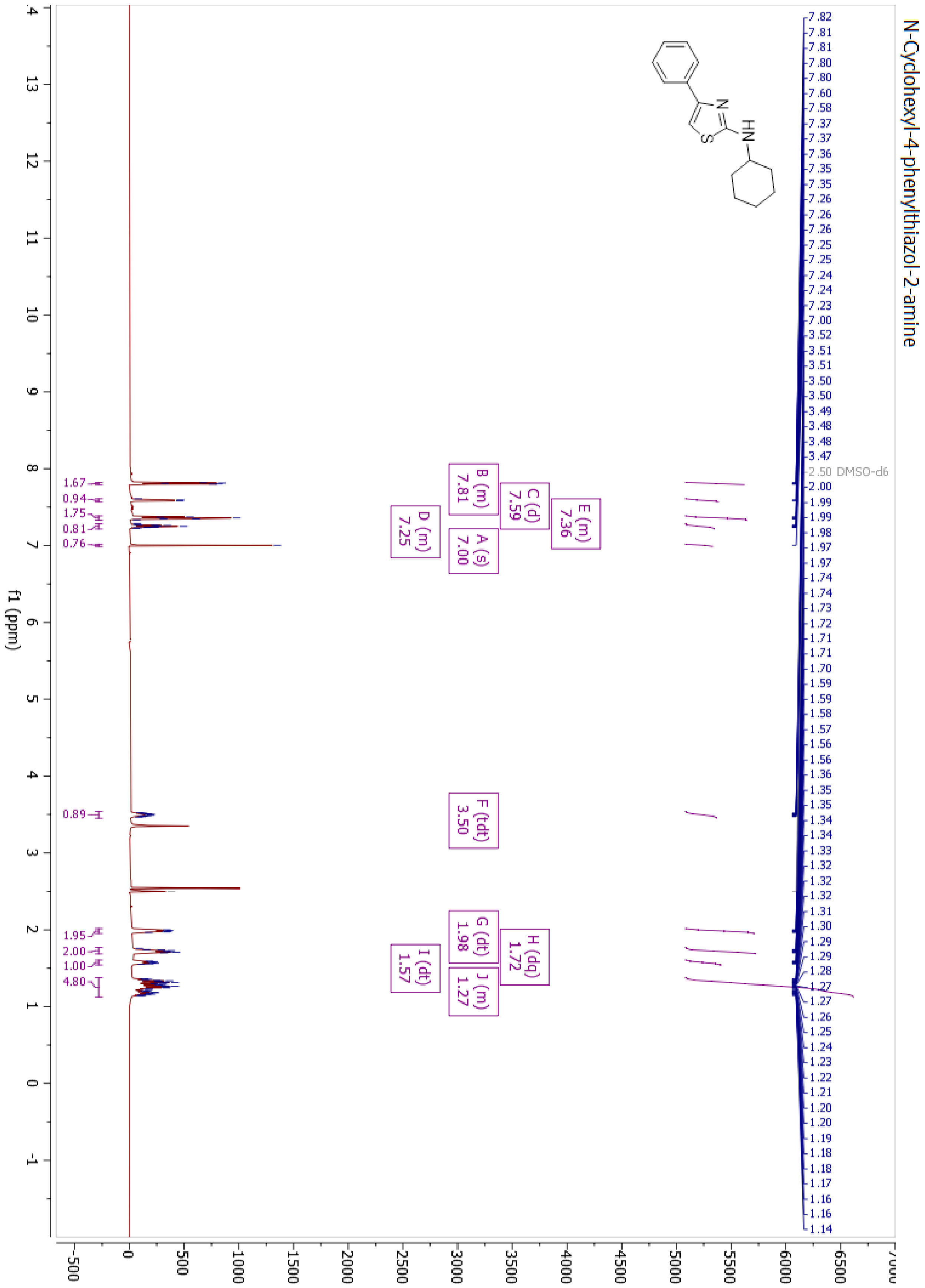

Figure S56. ^{1}H NMR spectrum of **3n** in d_6-DMSO (600 MHz)

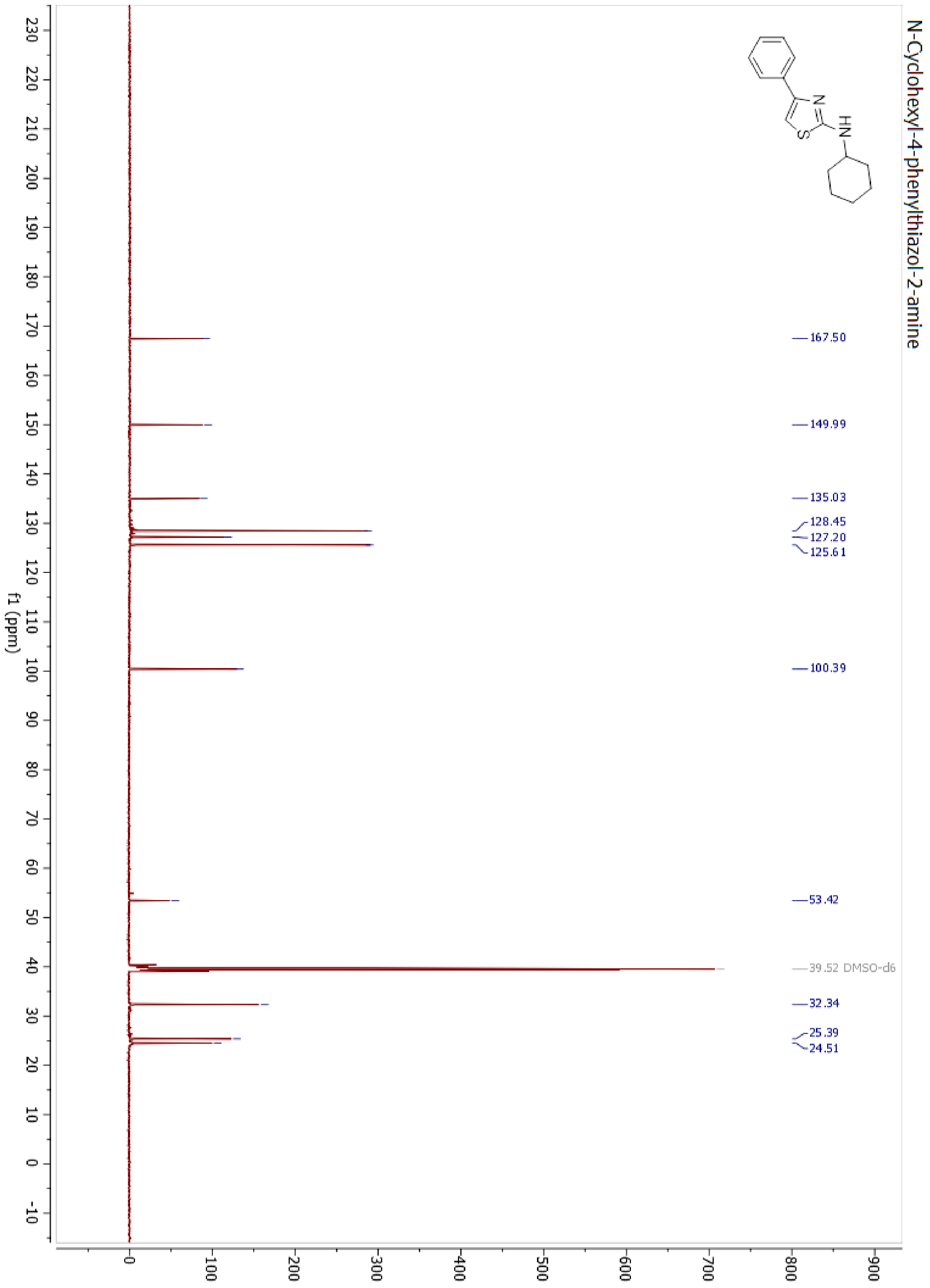

Figure S57. ^{13}C NMR spectrum of **3n** in d_6-DMSO (151 MHz)

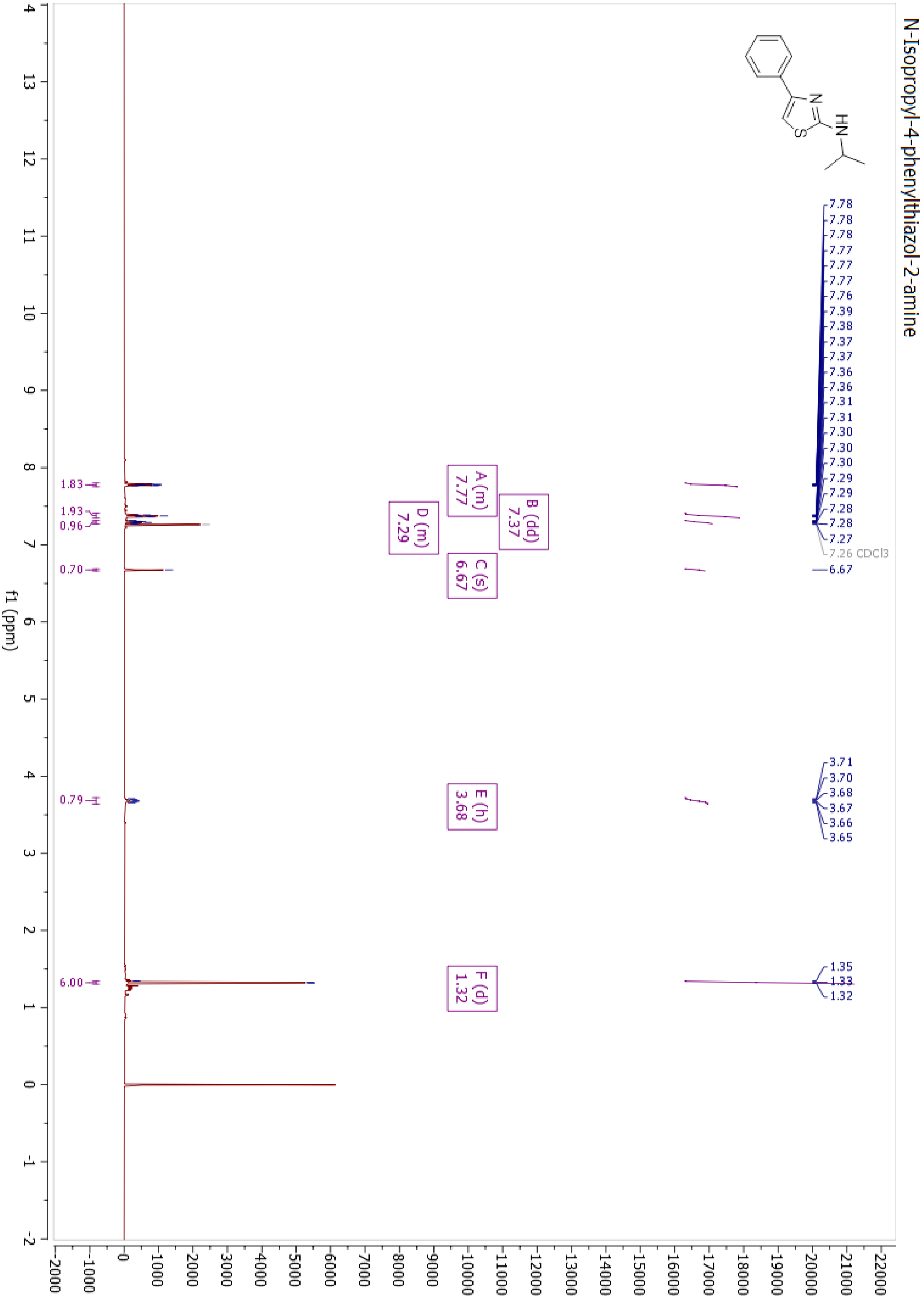

Figure S58. ^{1}H NMR spectrum of **3o** in d_6-DMSO (600 MHz)

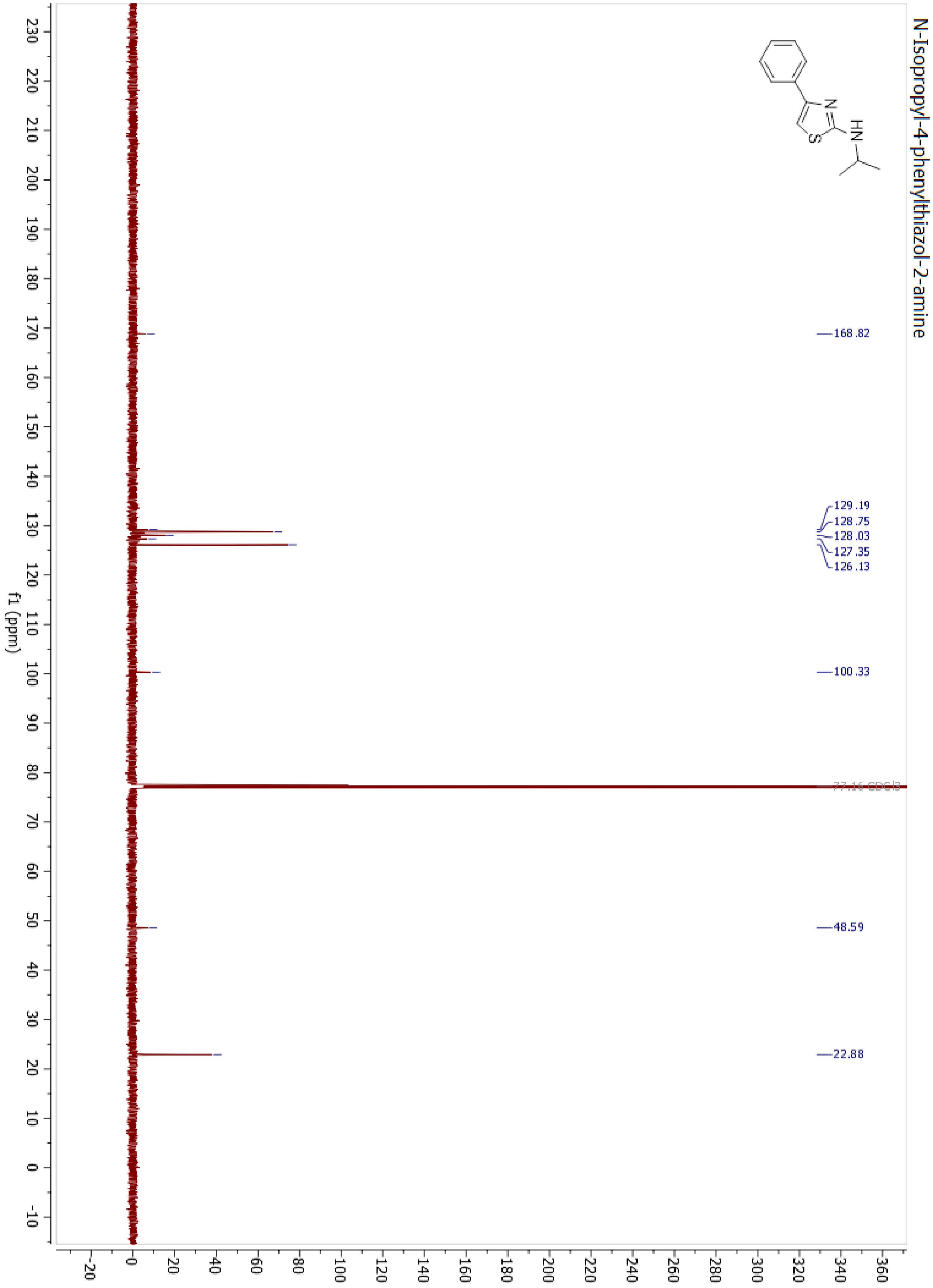

Figure S59. ^{13}C NMR spectrum of **3o** in d_6-DMSO (151 MHz)

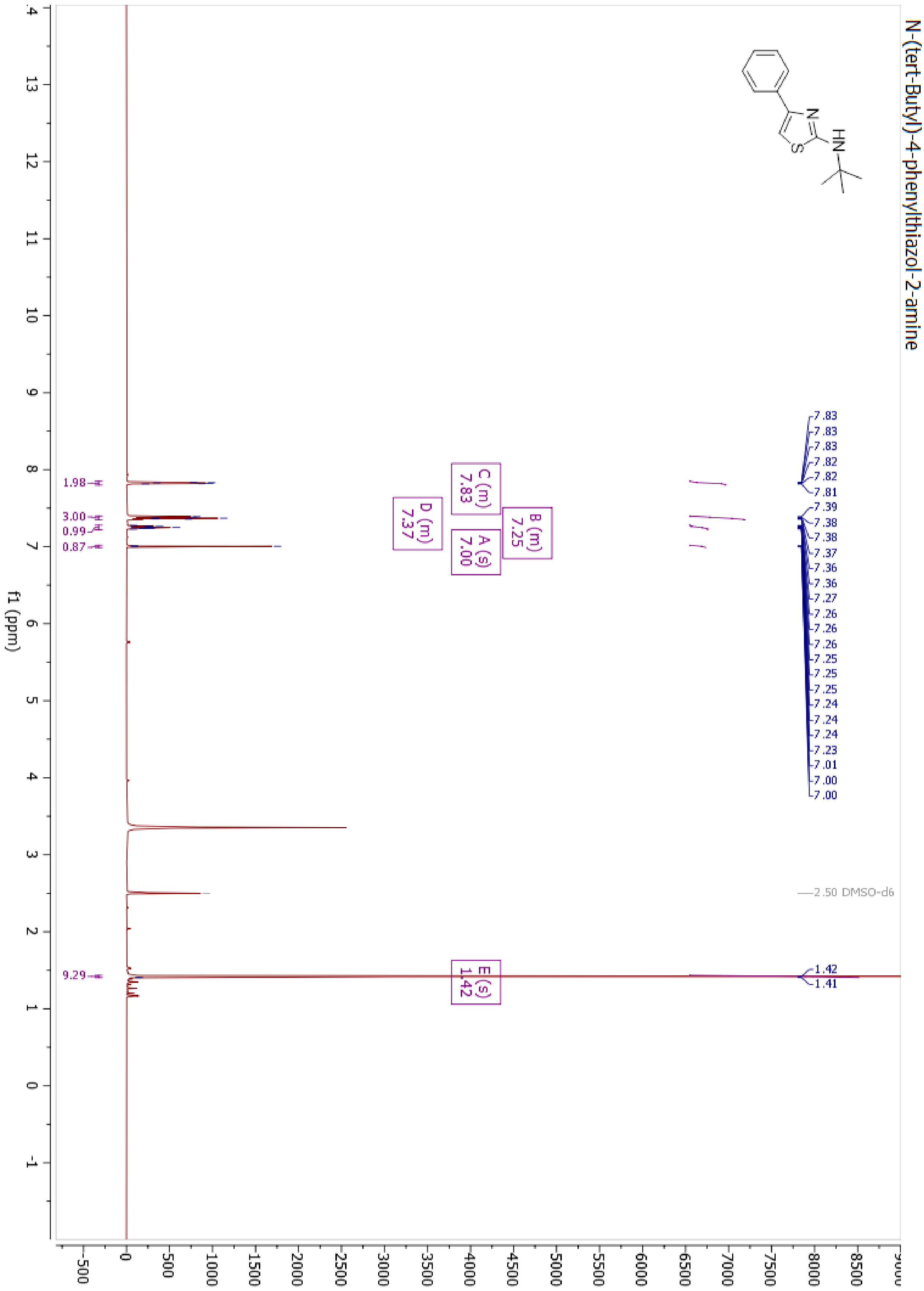

Figure S60. ^{1}H NMR spectrum of **3p** in d_6-DMSO (600 MHz)

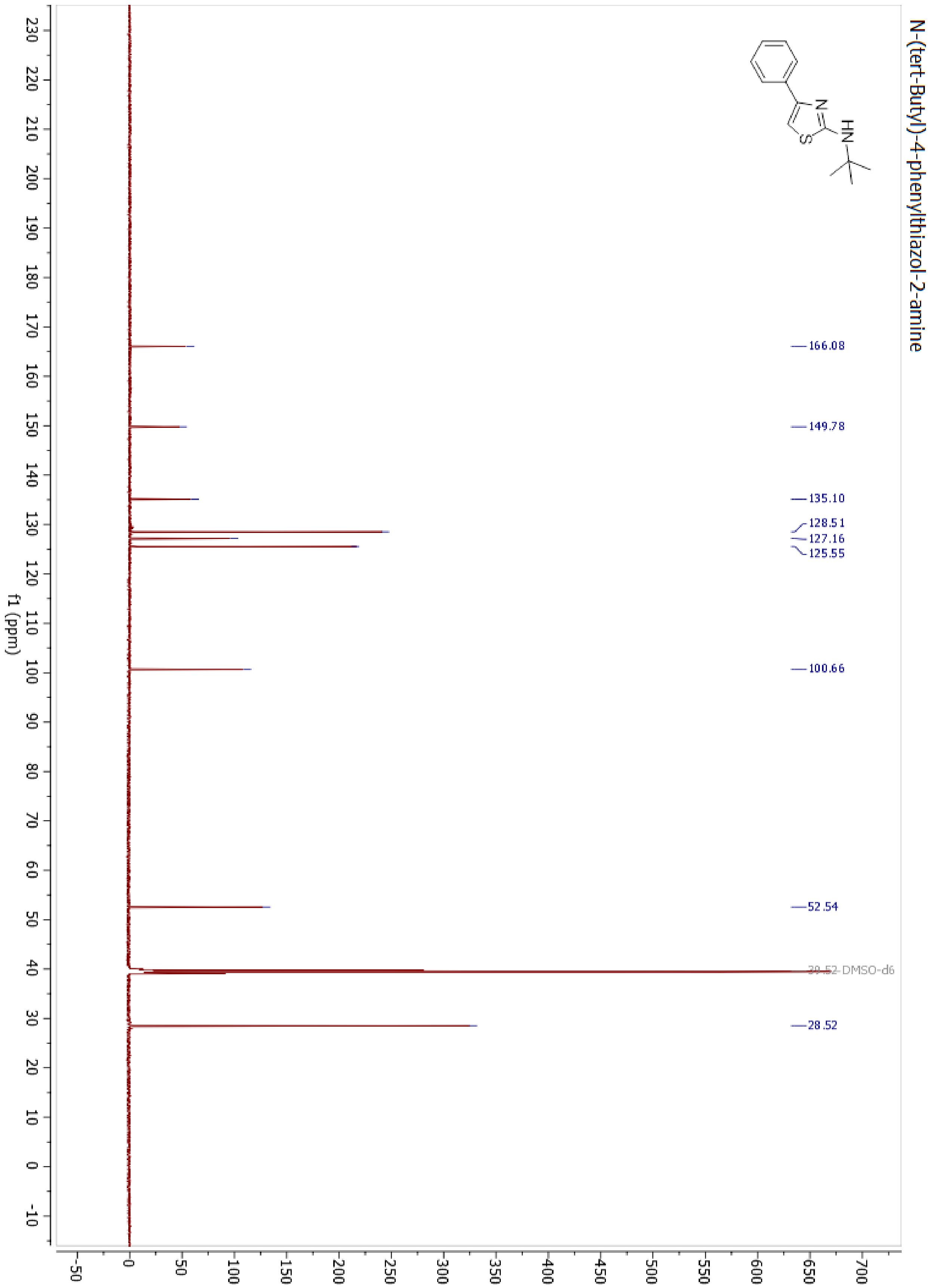

Figure S61. ^{13}C NMR spectrum of **3p** in d_6-DMSO (151 MHz)

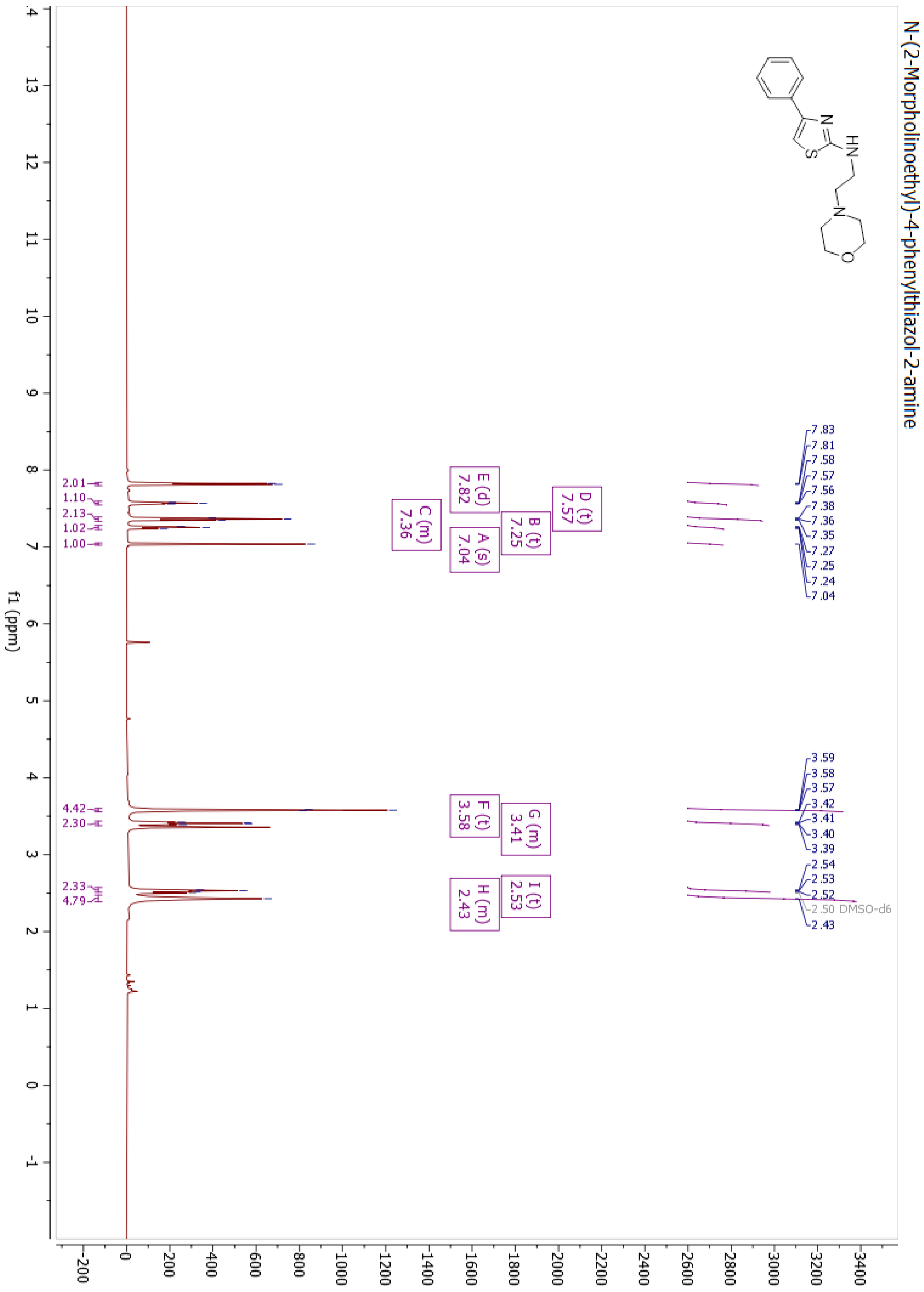

Figure S62. ^{1}H NMR spectrum of **3q** in d_6-DMSO (600 MHz)

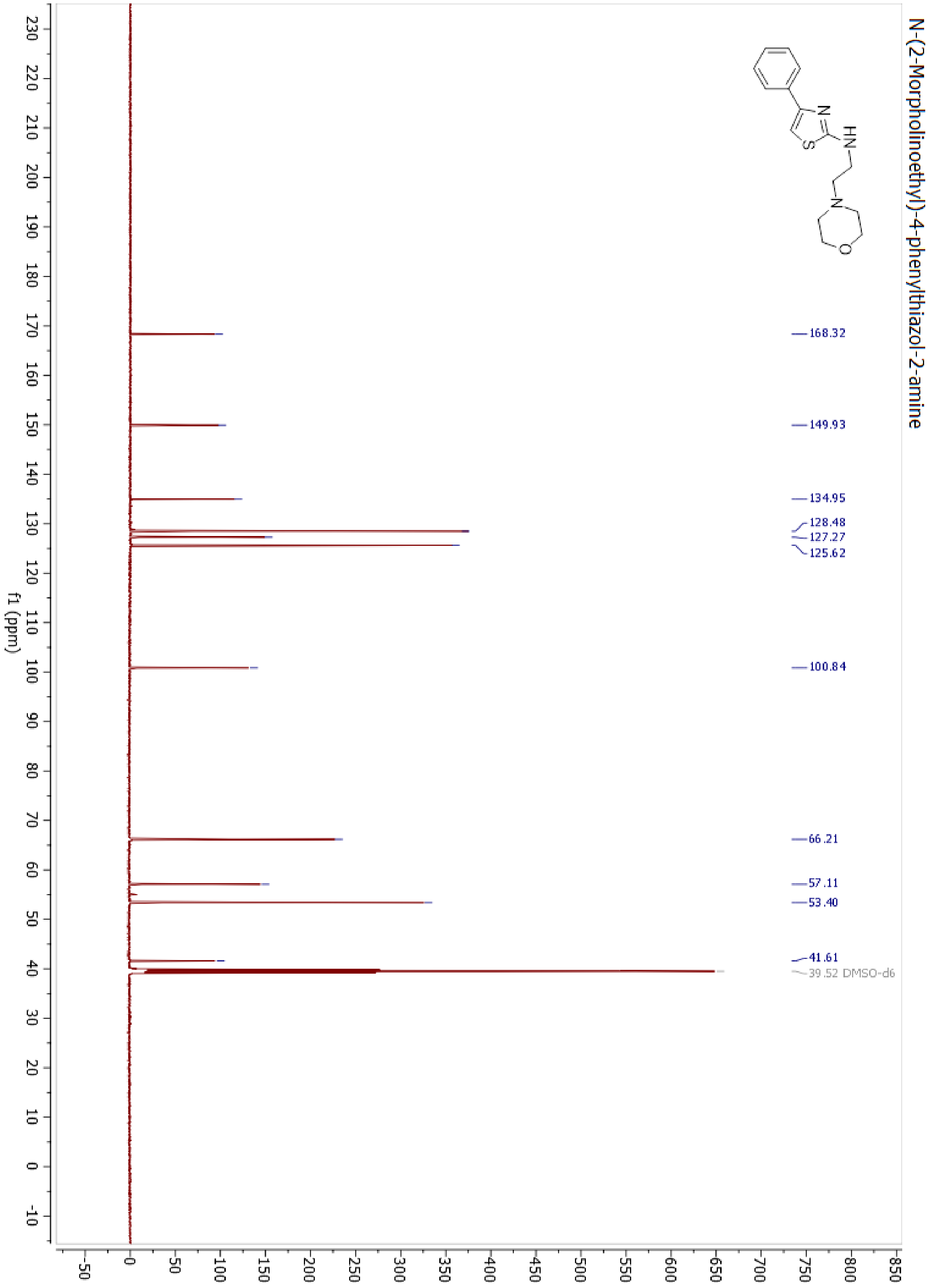

Figure S63. ^{13}C NMR spectrum of **3q** in d_6-DMSO (151 MHz)

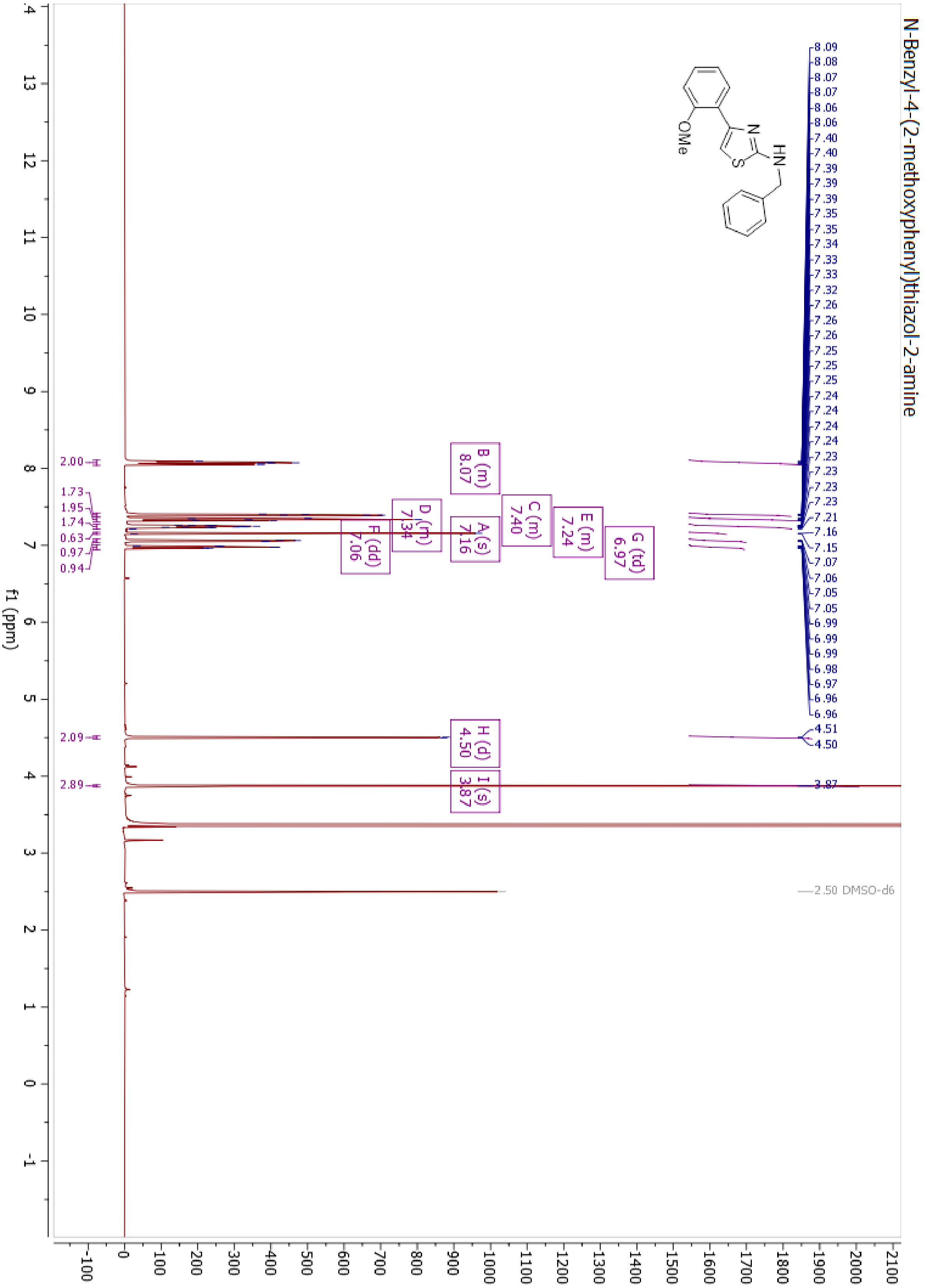

Figure S64. ^{1}H NMR spectrum of **3r** in d_6-DMSO (600 MHz)

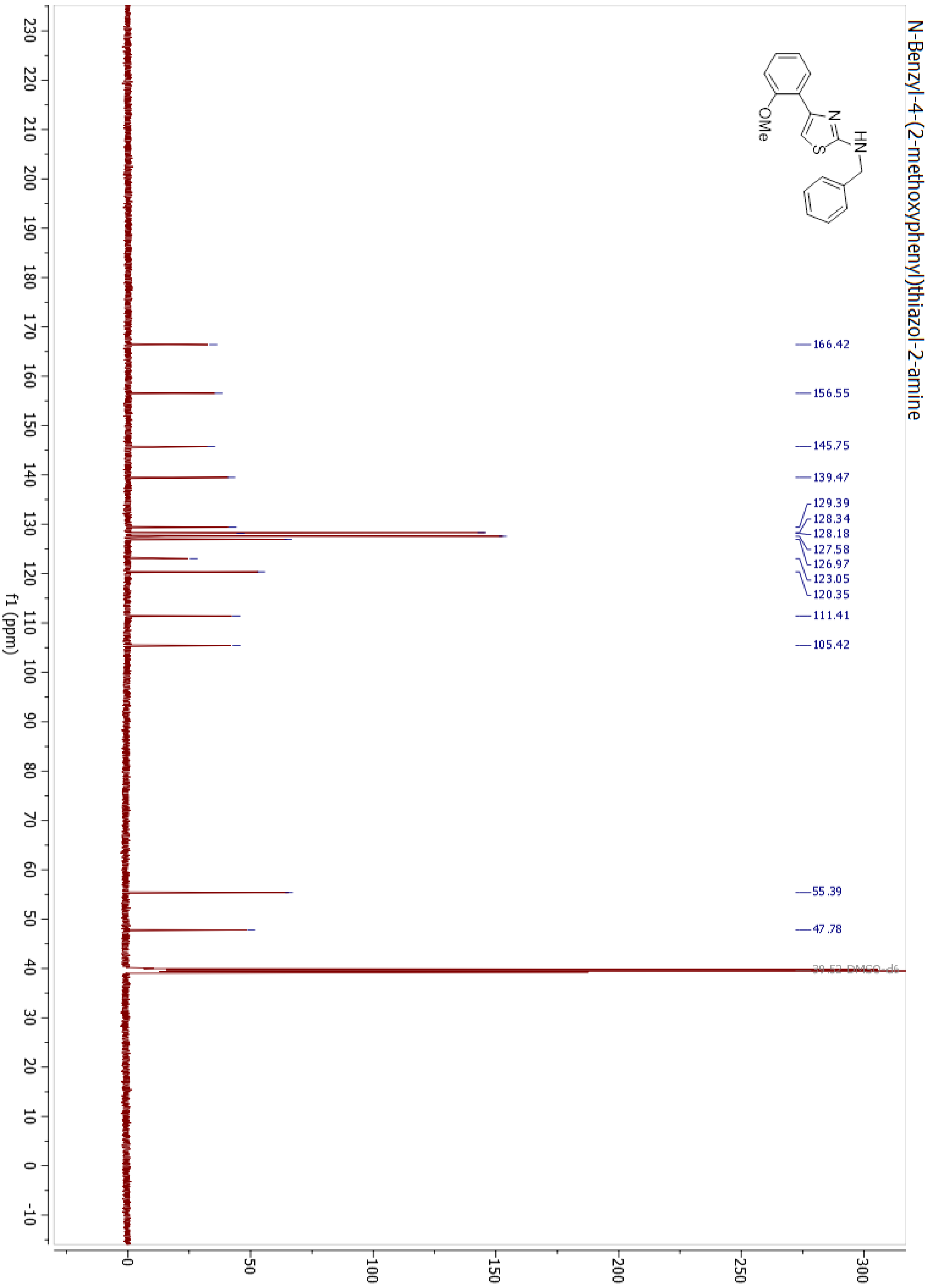

Figure S65. ^{13}C NMR spectrum of **3r** in d_6-DMSO (151 MHz)

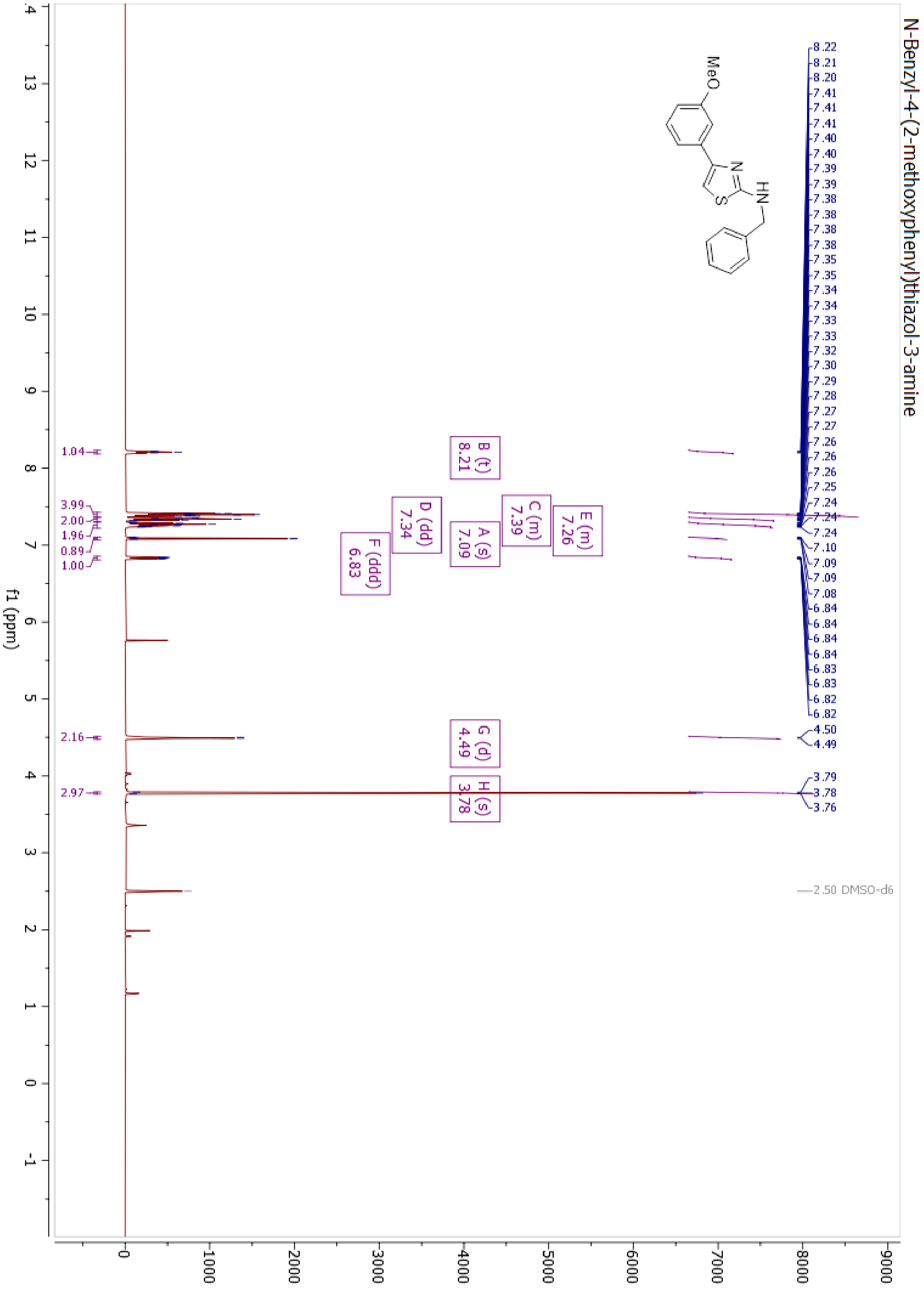

Figure S66. ^{1}H NMR spectrum of **3s** in d_6-DMSO (600 MHz)

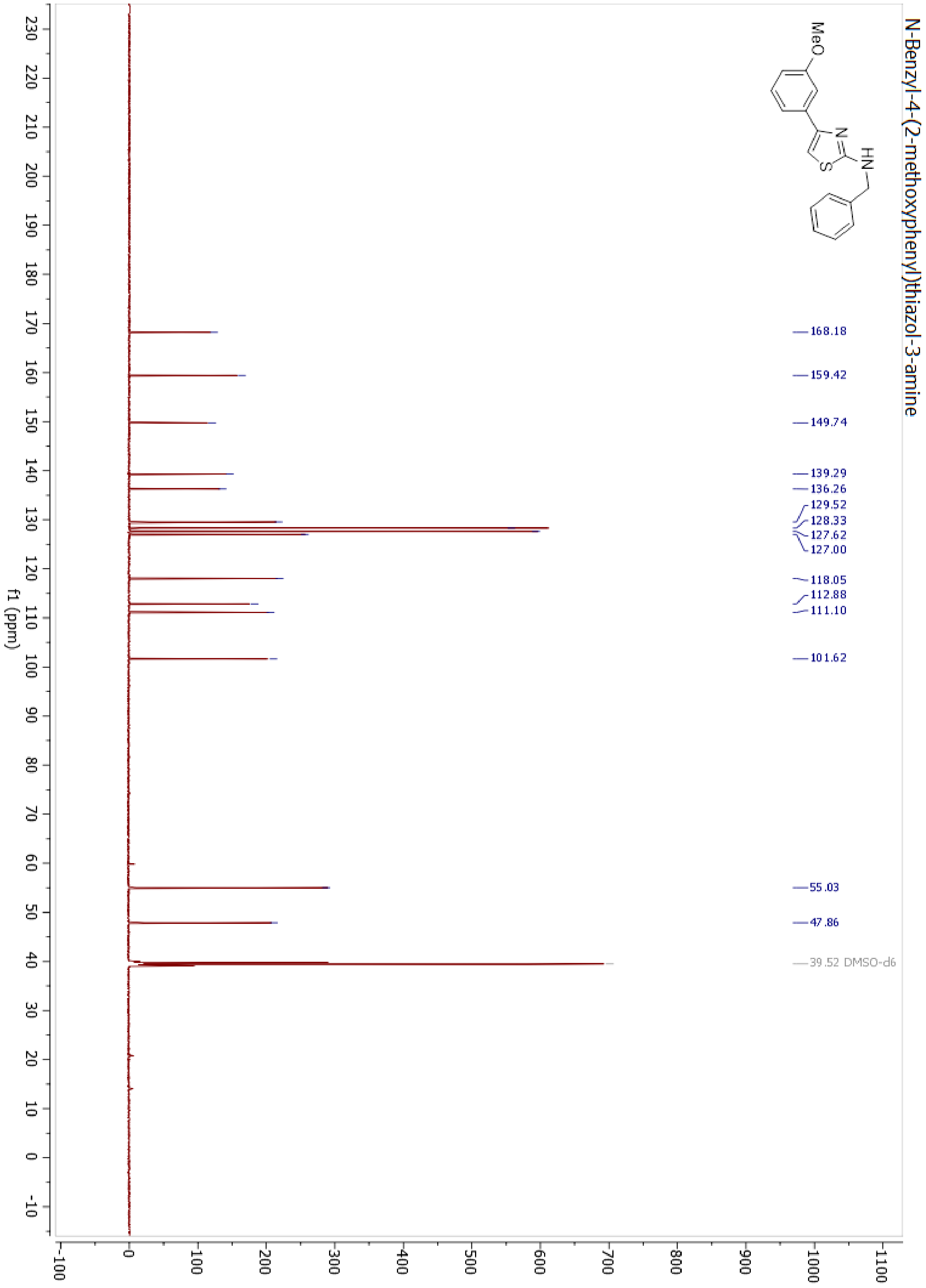

Figure S67. ^{13}C NMR spectrum of **3s** in d_6-DMSO (151 MHz)

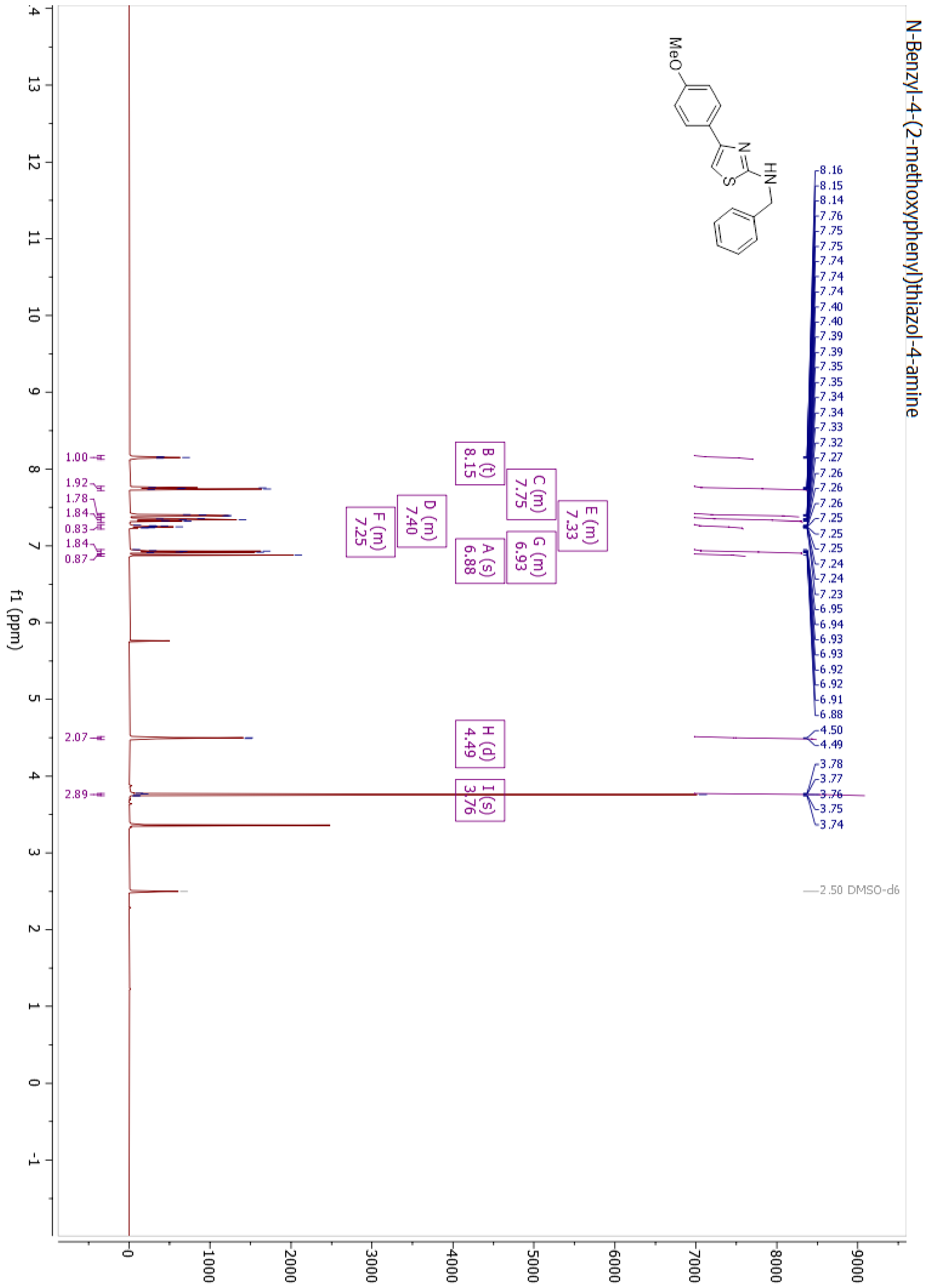

Figure S68. ^{1}H NMR spectrum of **3t** in d_6-DMSO (600 MHz)

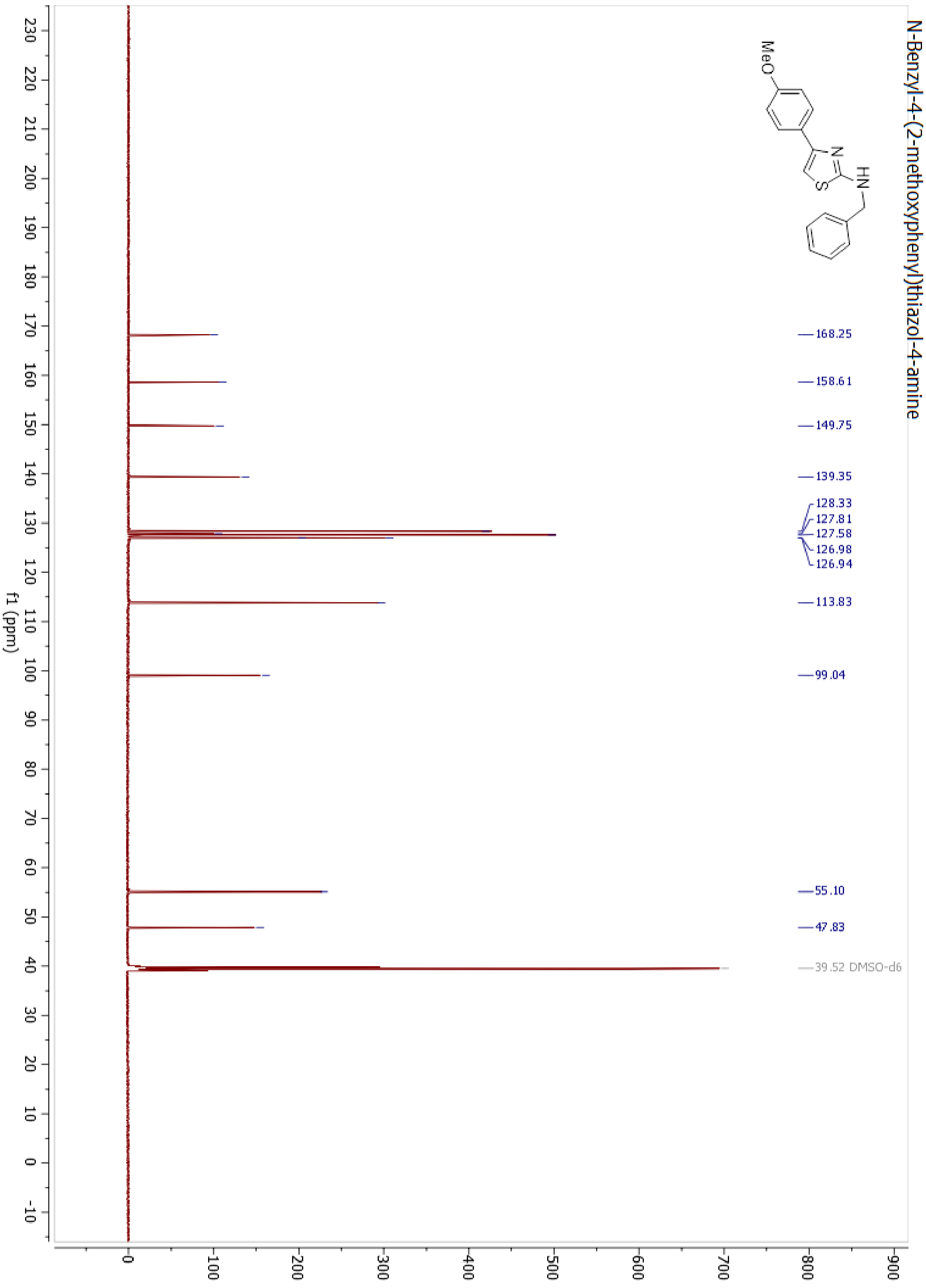

Figure S69. ^{13}C NMR spectrum of **3t** in d_6-DMSO (151 MHz)

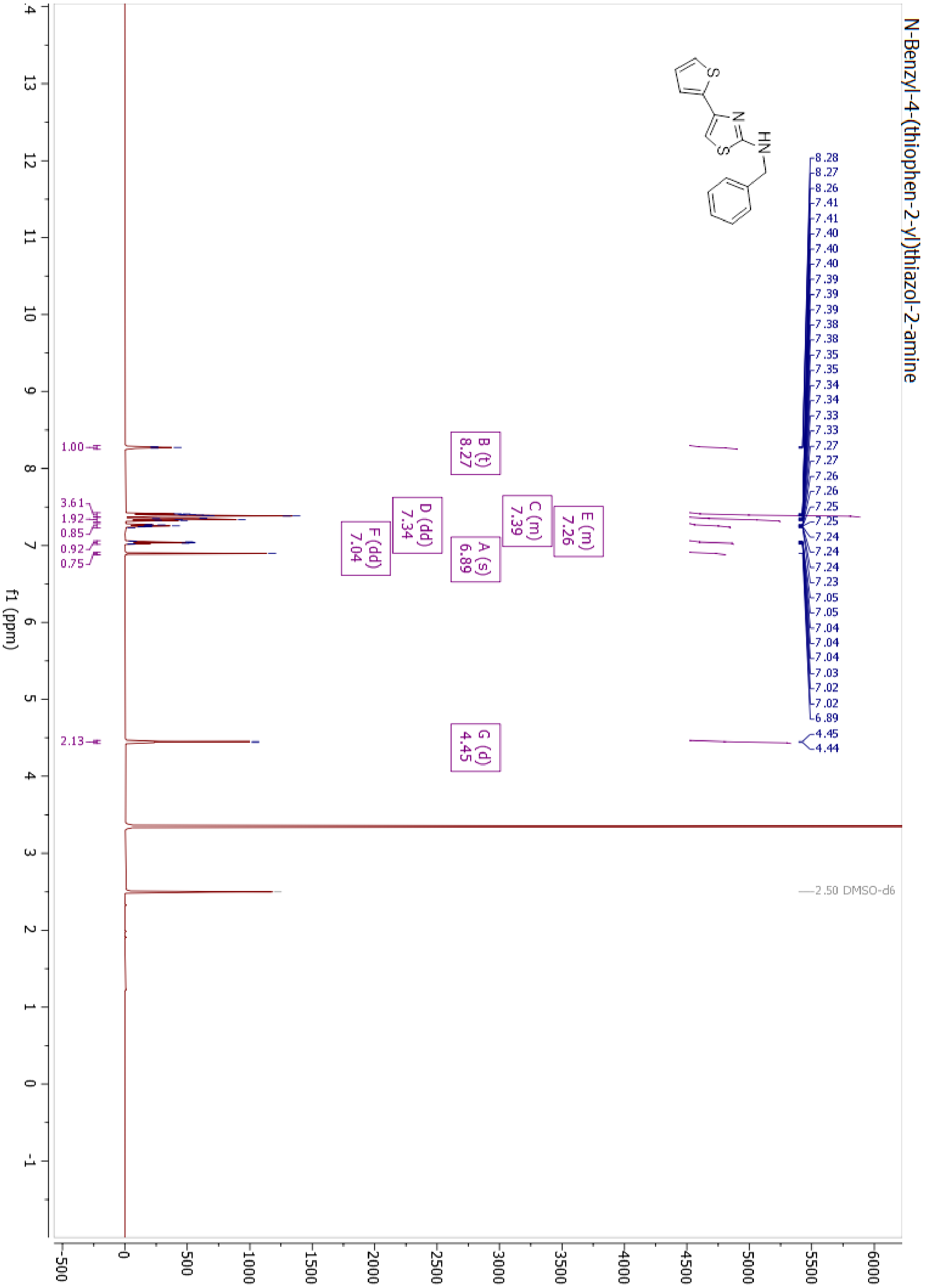

Figure S70. ^{1}H NMR spectrum of **3u** in d_6-DMSO (600 MHz)

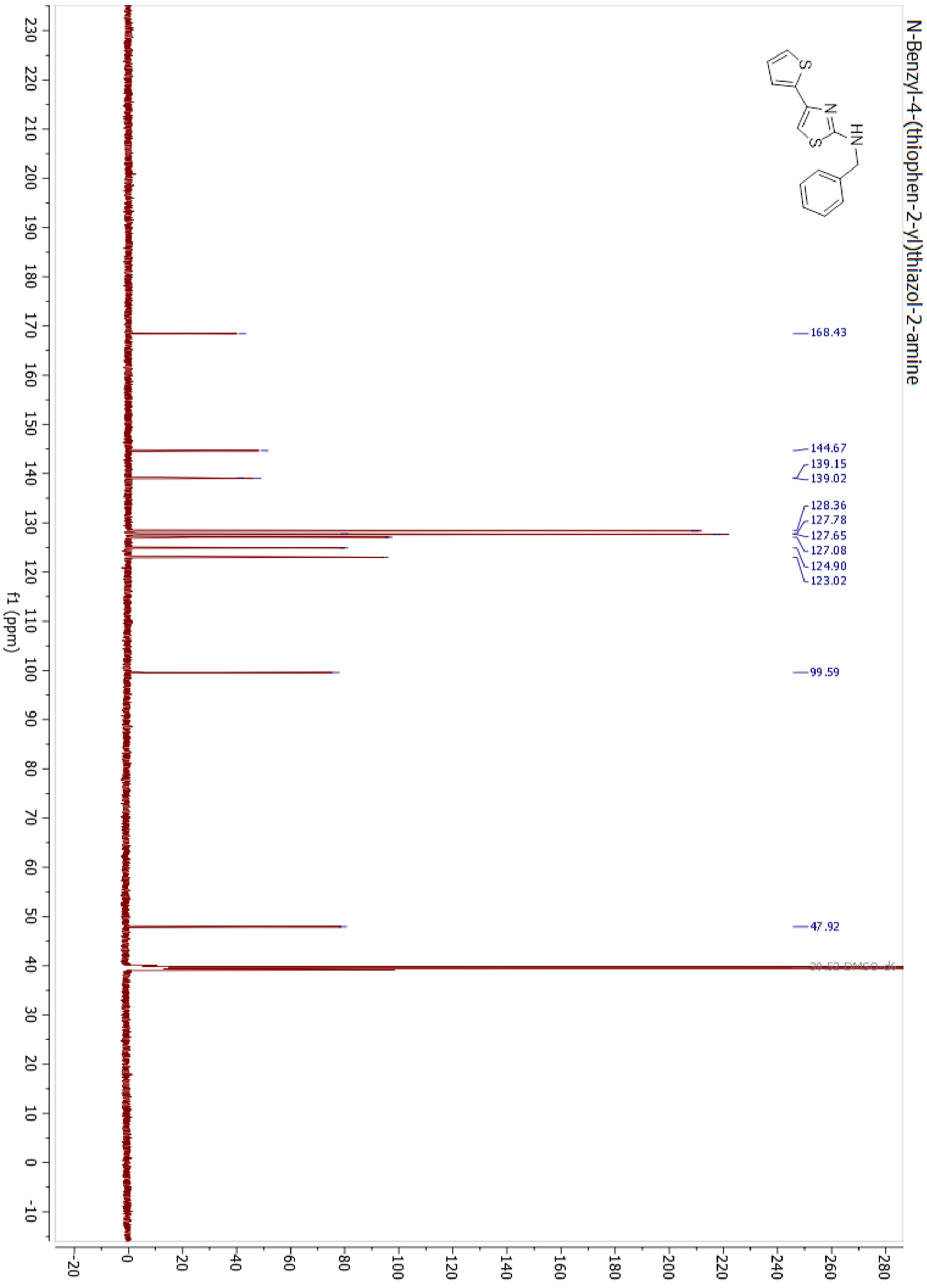

Figure S71. ^{13}C NMR spectrum of **3u** in d_6-DMSO (151 MHz)

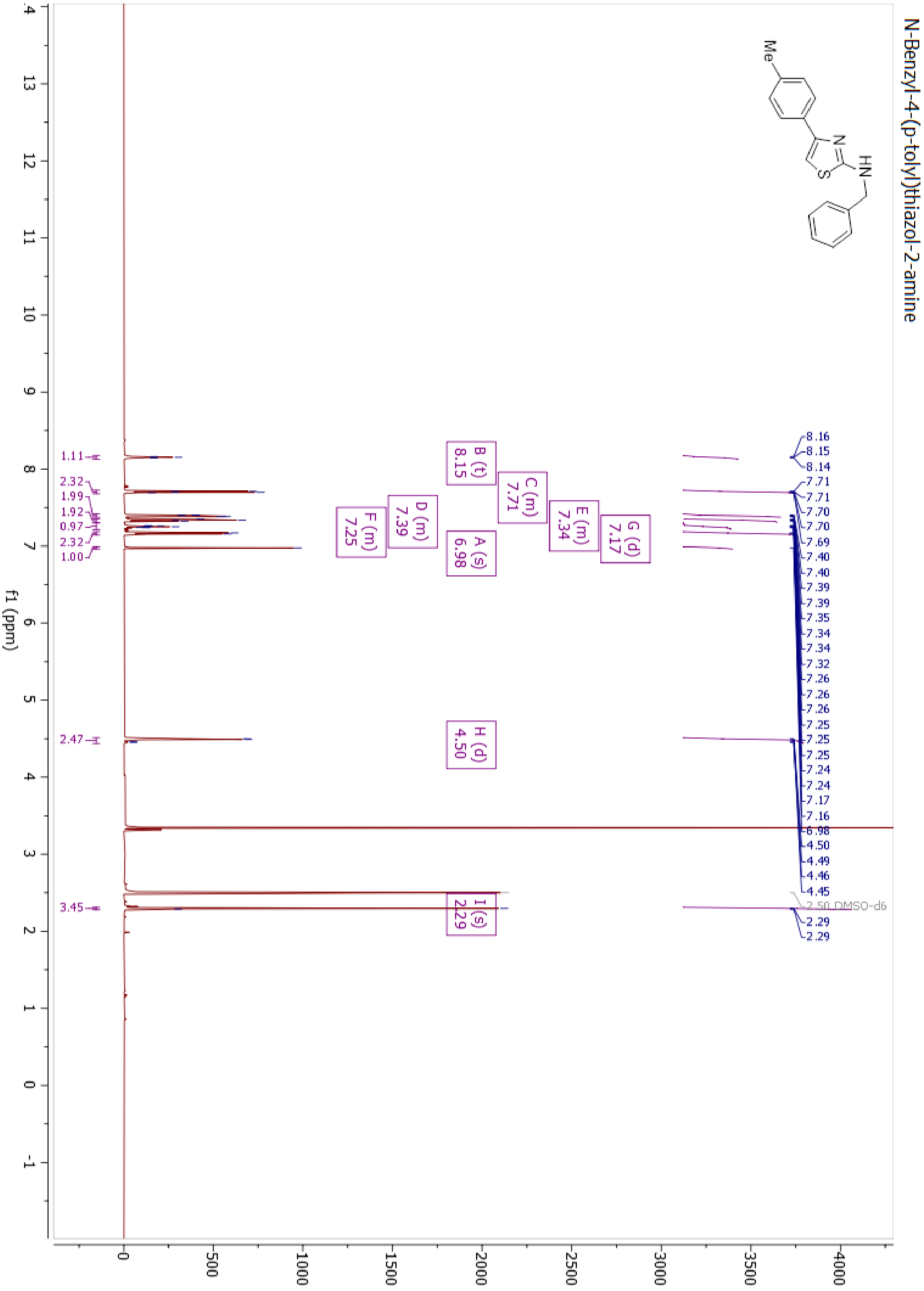

Figure S72. ^{1}H NMR spectrum of **3v** in d_6-DMSO (600 MHz)

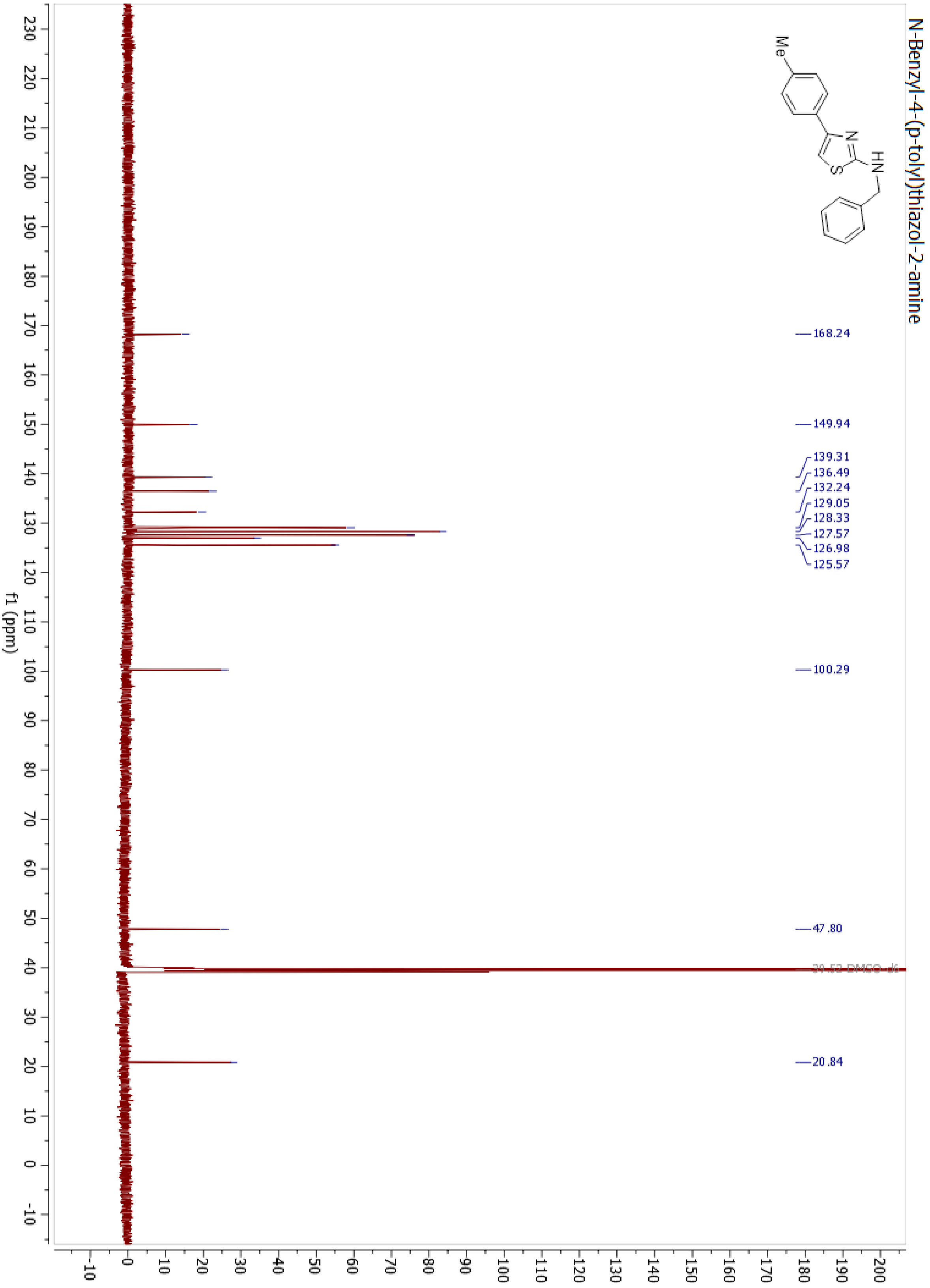

Figure S73. ^{13}C NMR spectrum of **3v** in d_6-DMSO (151 MHz)

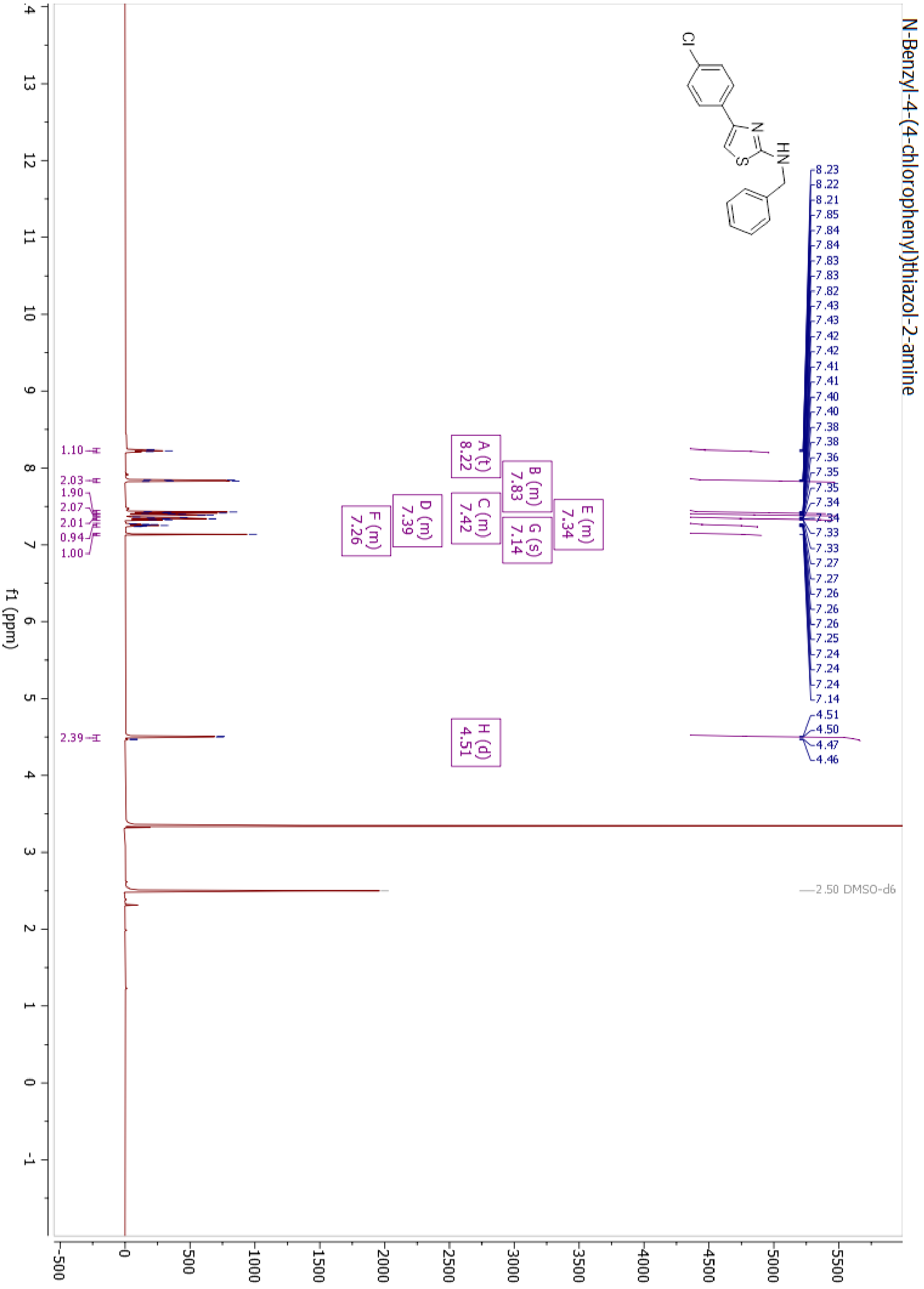

Figure S74. ^{1}H NMR spectrum of **3w** in d_6-DMSO (600 MHz)

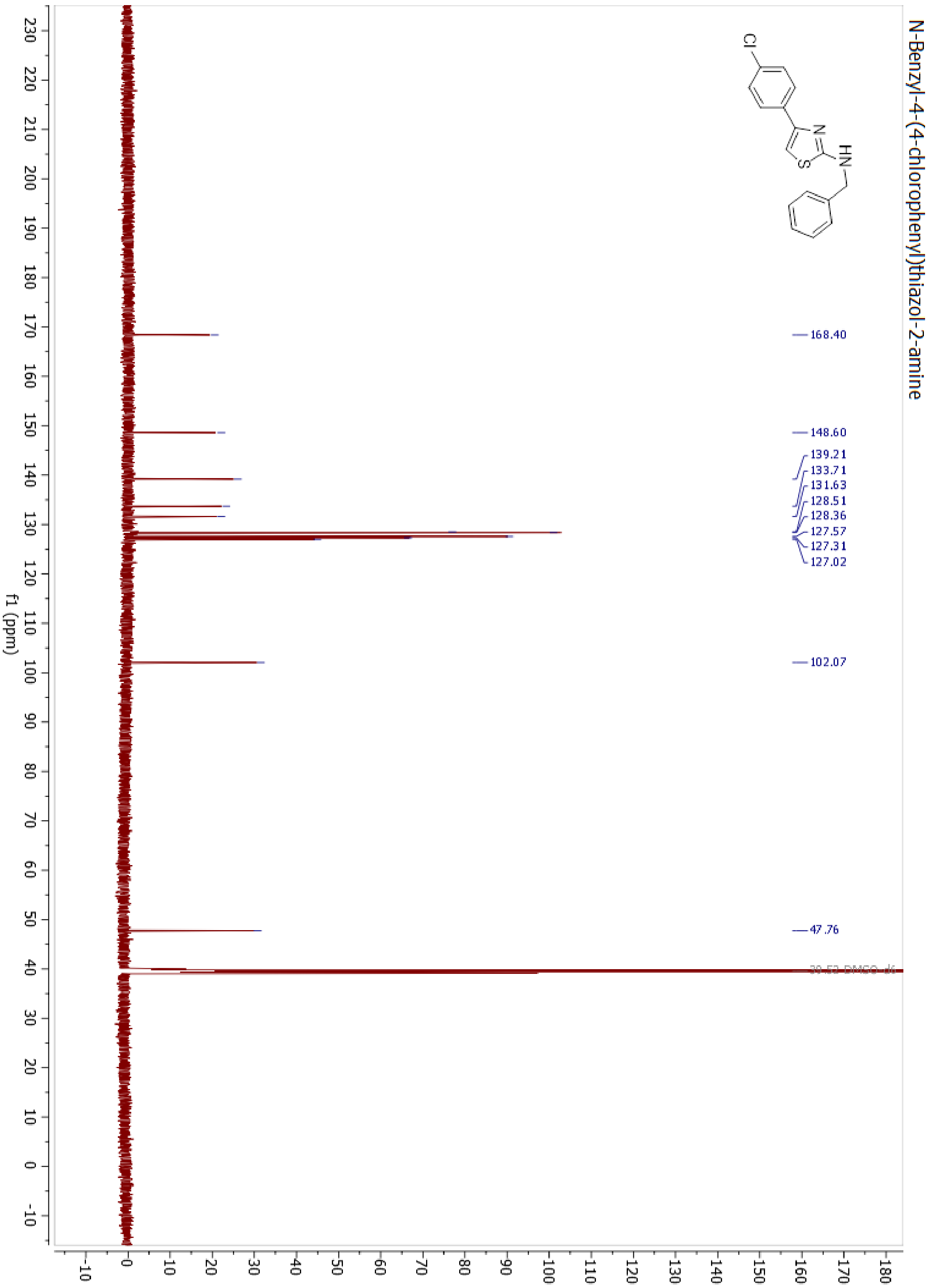

Figure S75. ^{13}C NMR spectrum of **3w** in d_6-DMSO (151 MHz)

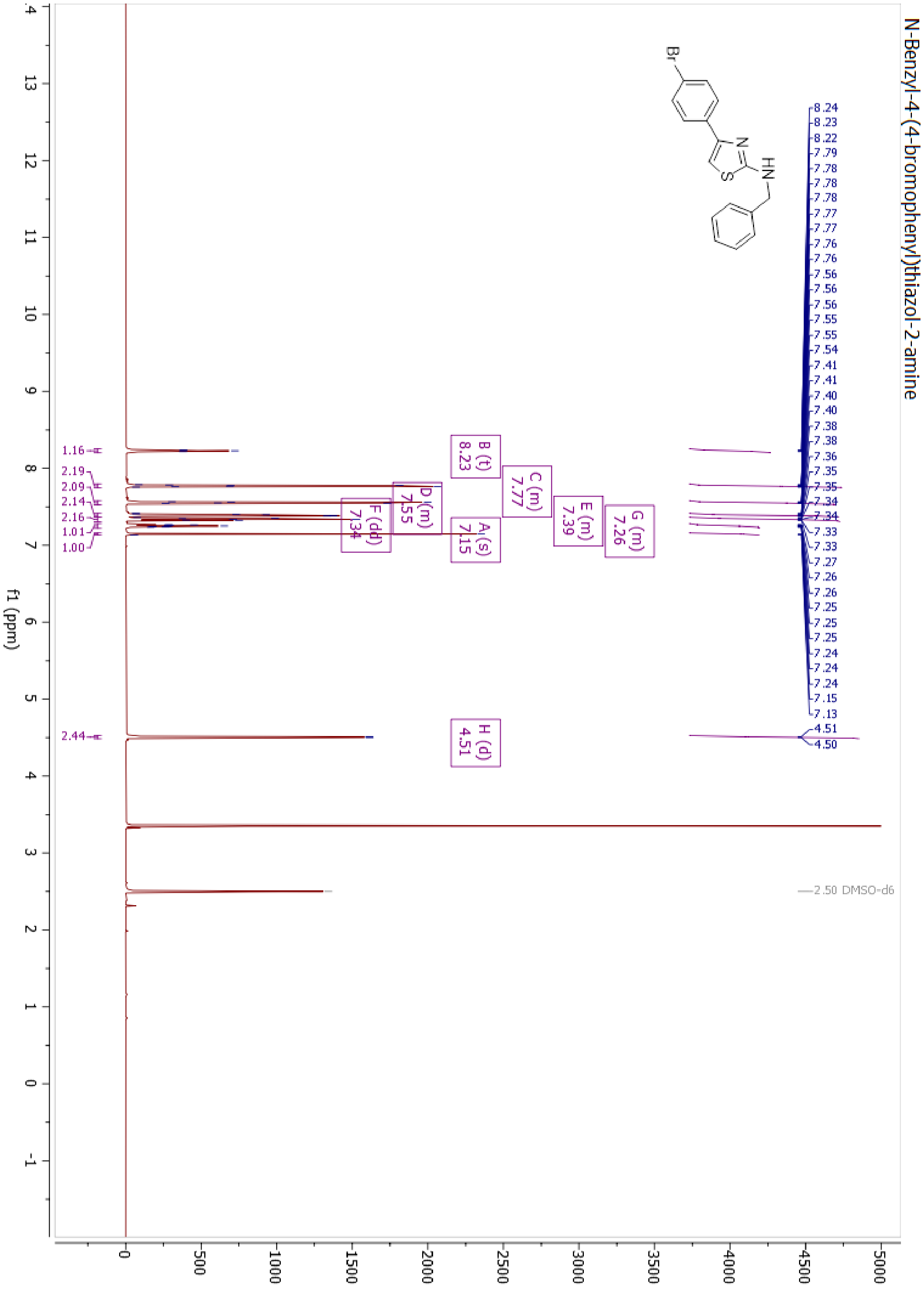

Figure S76. ^{1}H NMR spectrum of **3x** in d_6-DMSO (600 MHz)

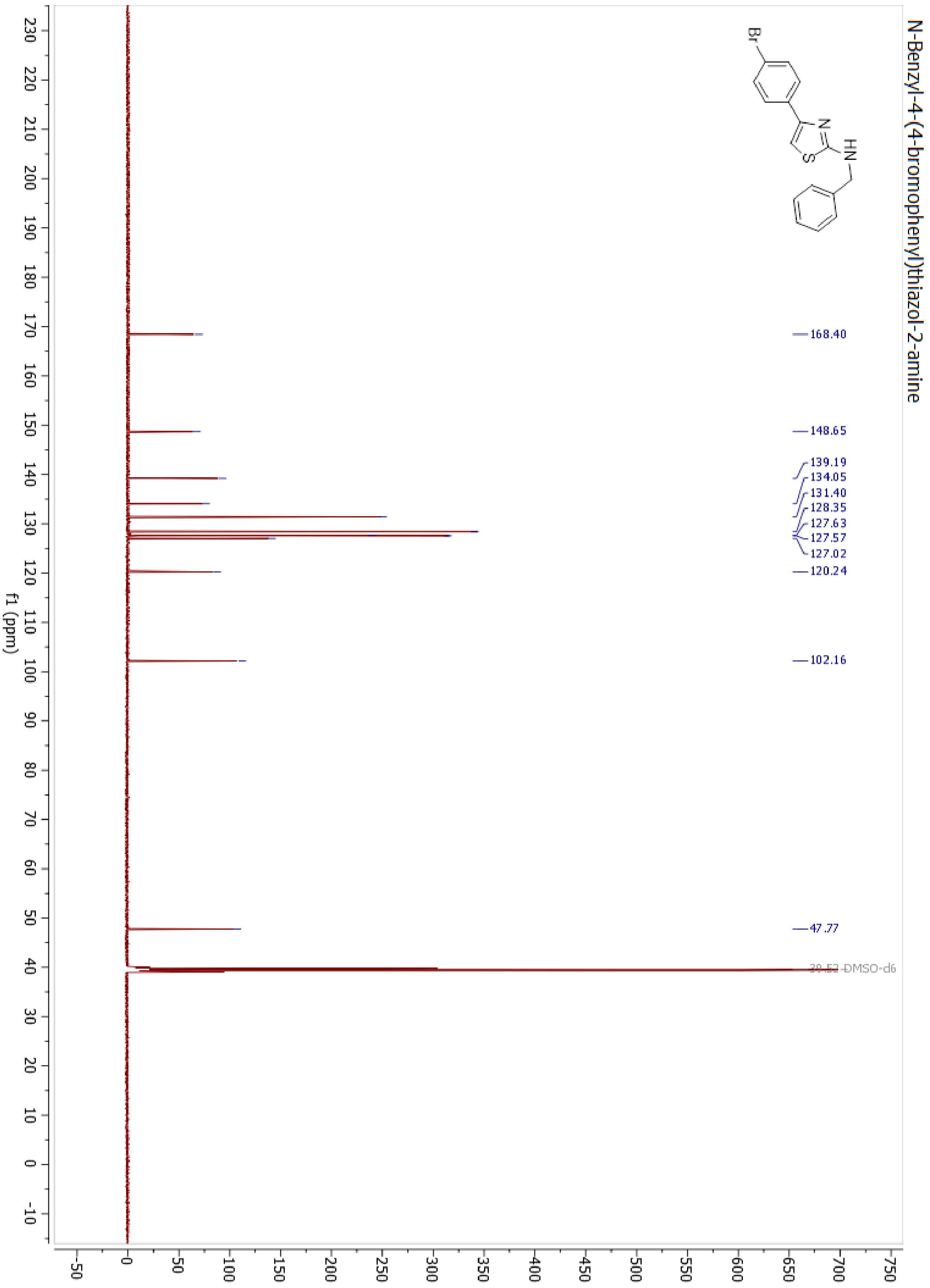

Figure S77. ^{13}C NMR spectrum of **3x** in d_6-DMSO (151 MHz)

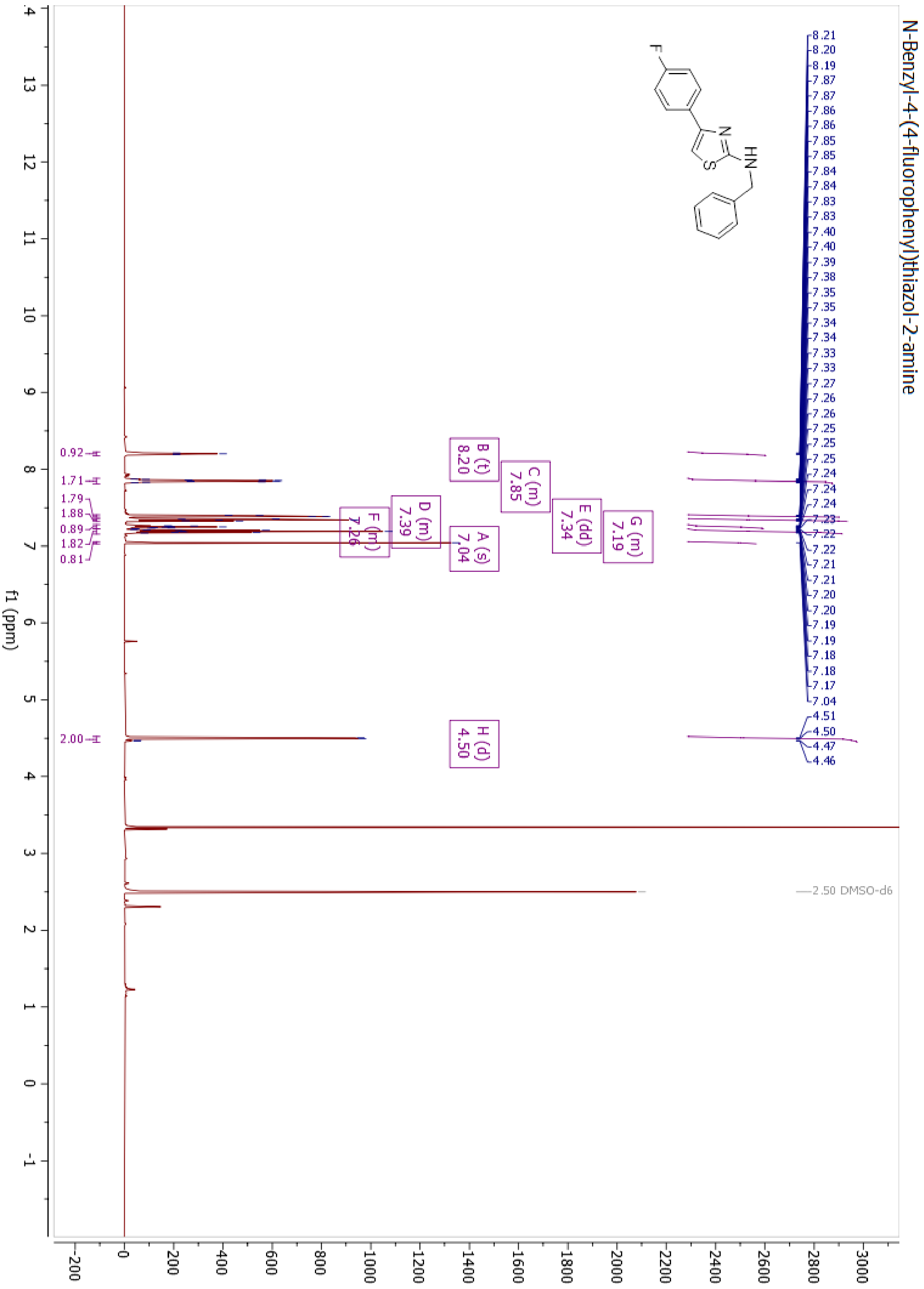

Figure S78. ^{1}H NMR spectrum of **3y** in d_6-DMSO (600 MHz)

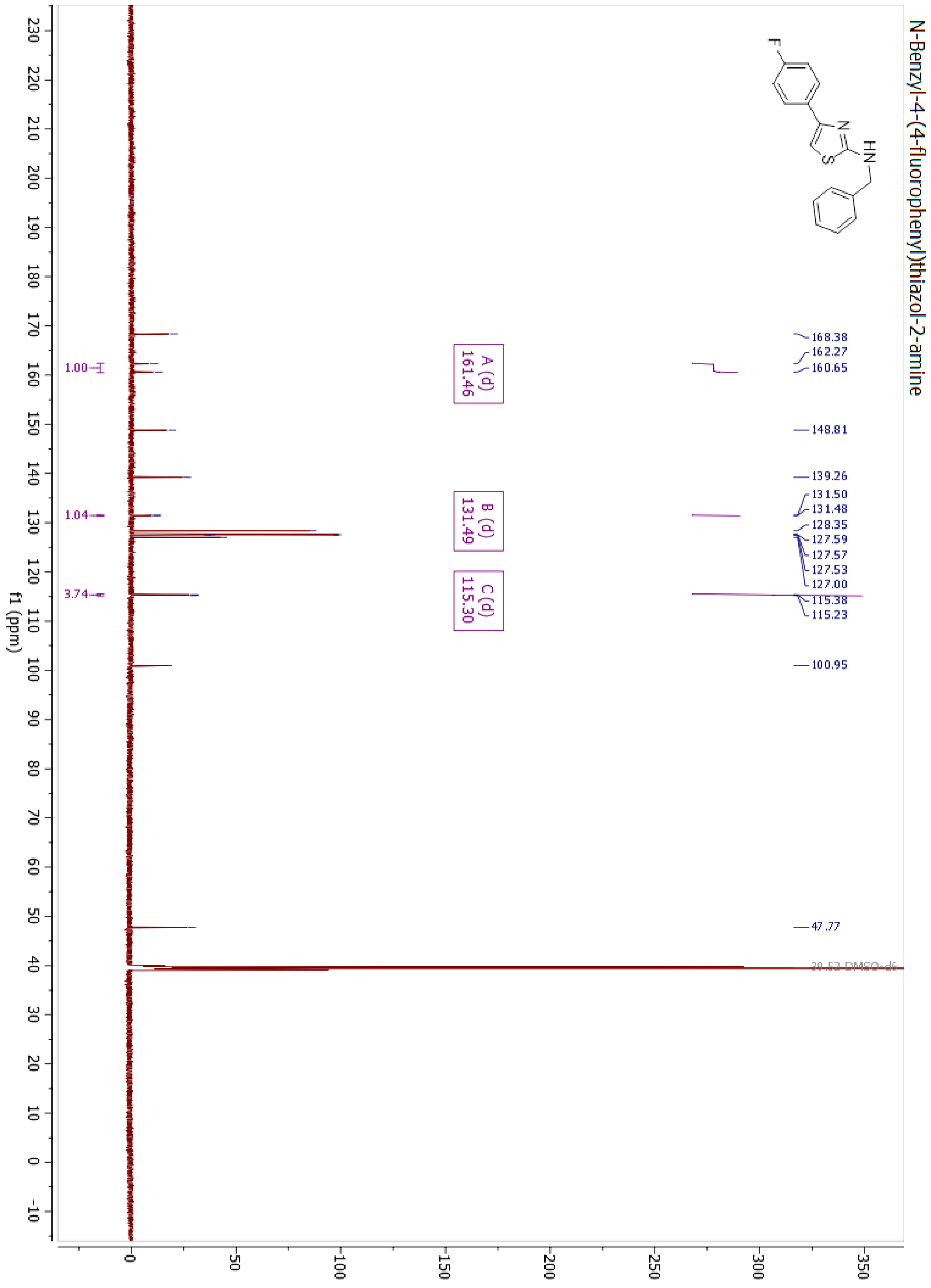

Figure S79. ^{13}C NMR spectrum of **3y** in d_6-DMSO (151 MHz)

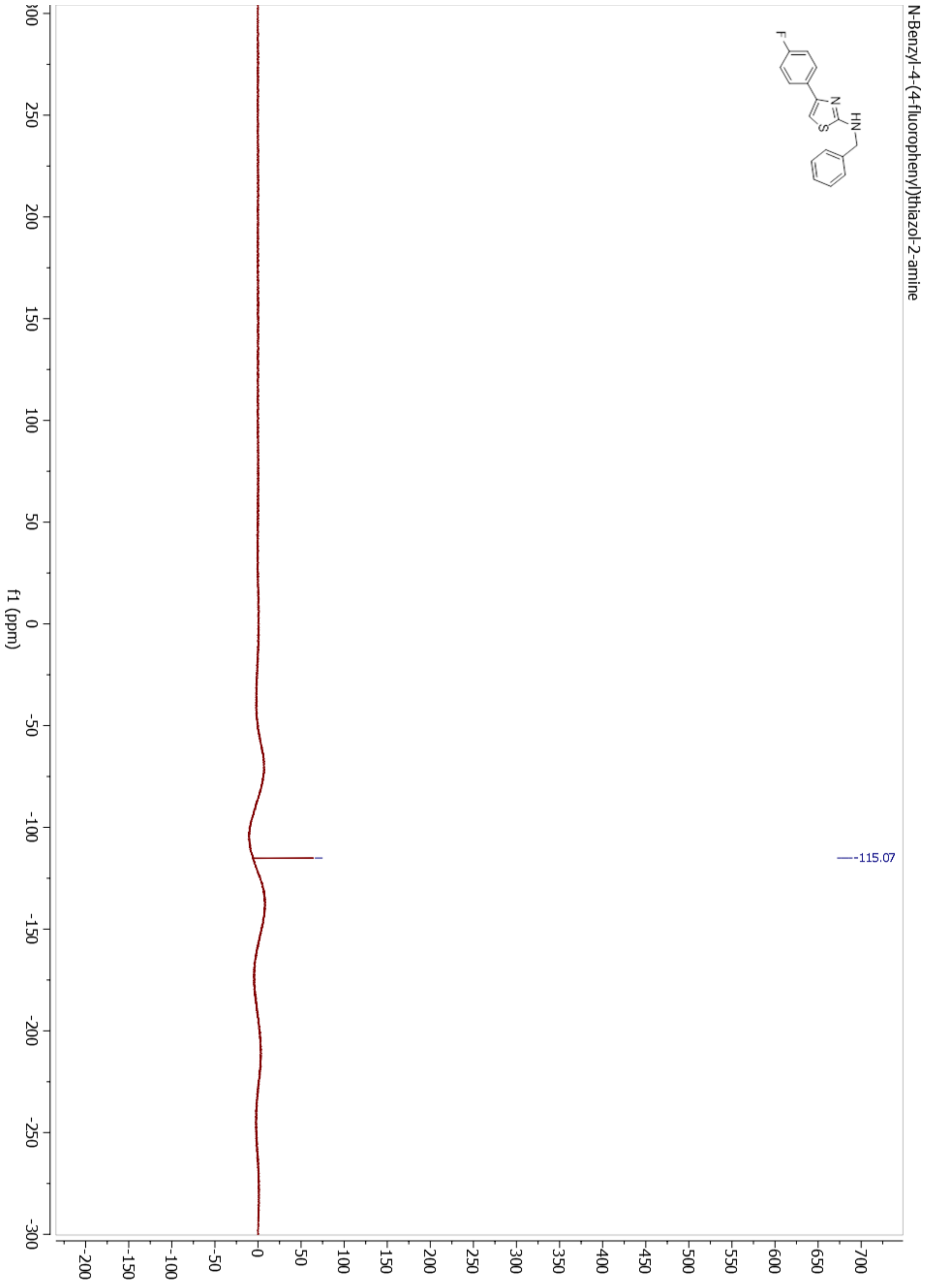

Figure S80. ^{19}F NMR spectrum of **3y** in d_6-DMSO (376 MHz)

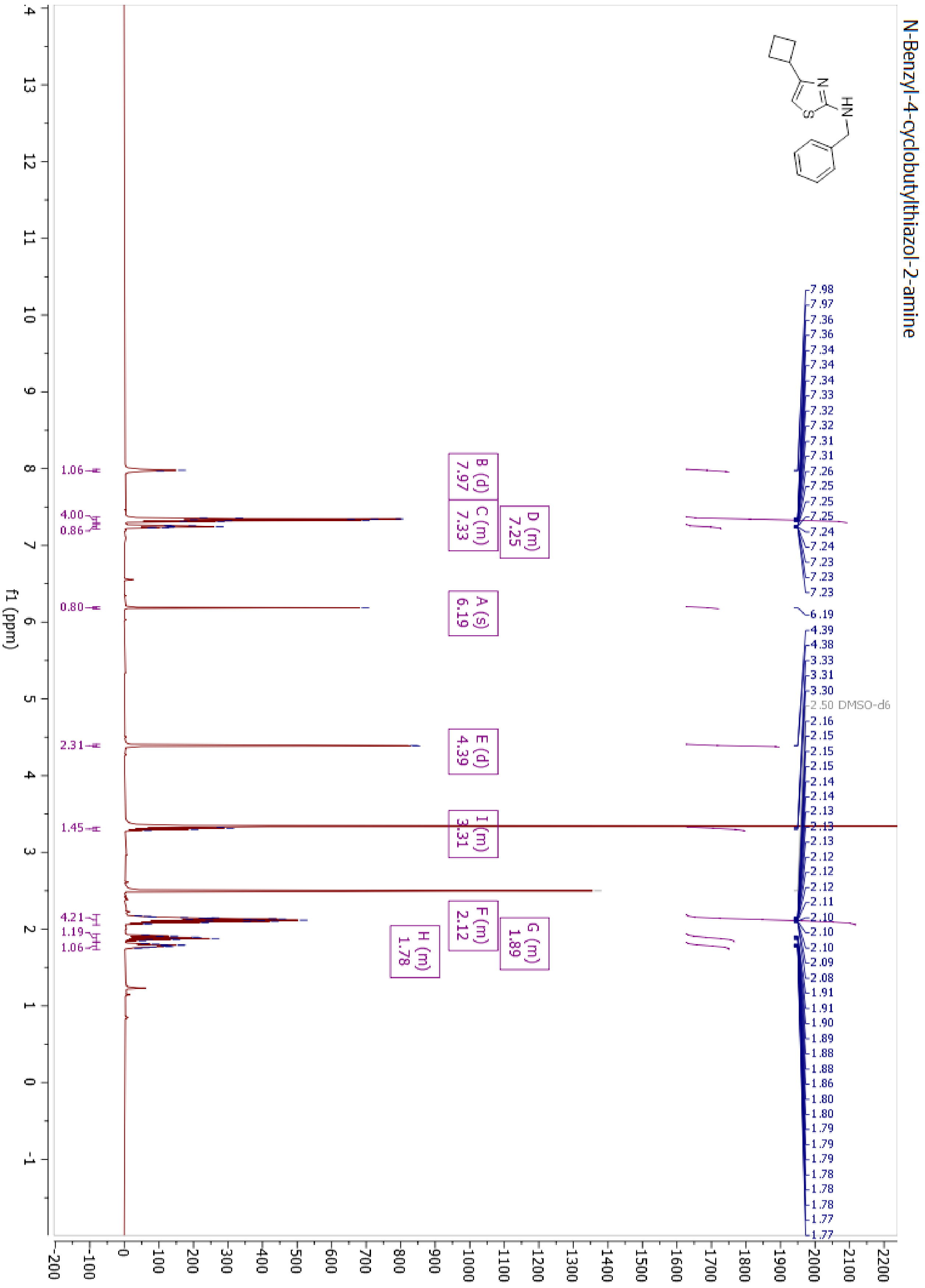

Figure S81. ^{1}H NMR spectrum of **3z** in d_6-DMSO (600 MHz)

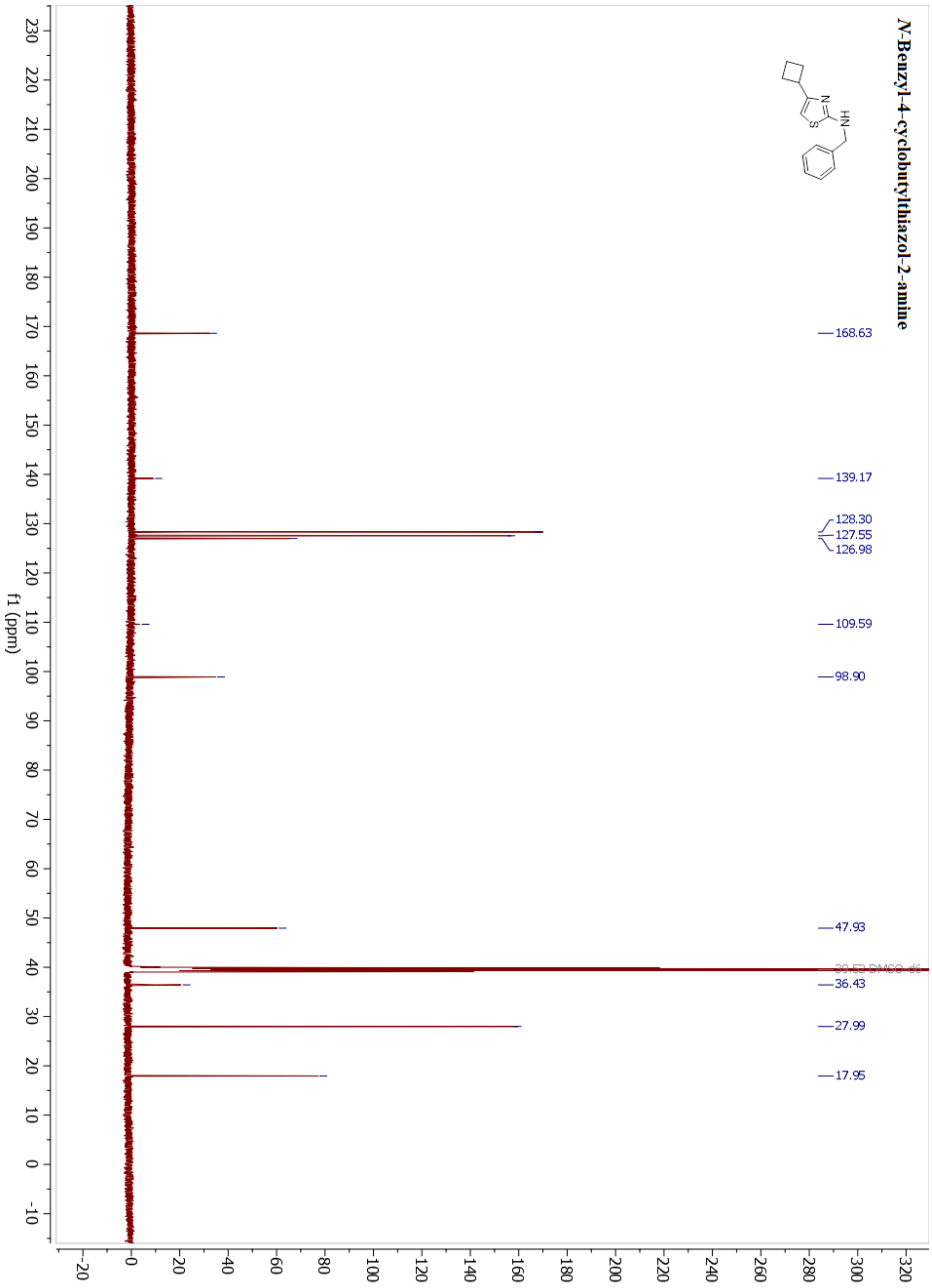

Figure S82. ^{13}C NMR spectrum of **3z** in d_6-DMSO (151 MHz)

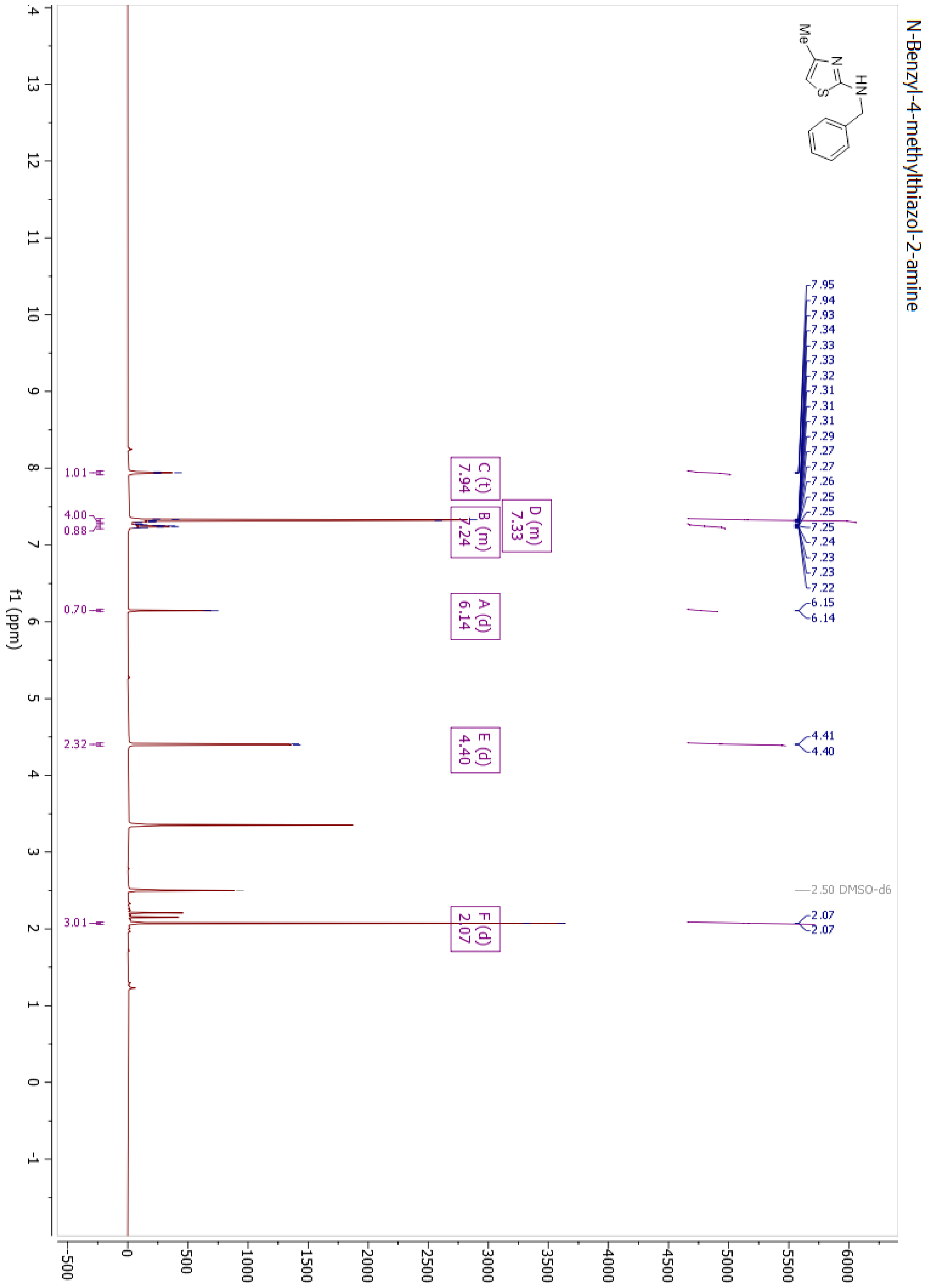

Figure S83. ^{1}H NMR spectrum of **3a’** in d_6-DMSO (600 MHz)

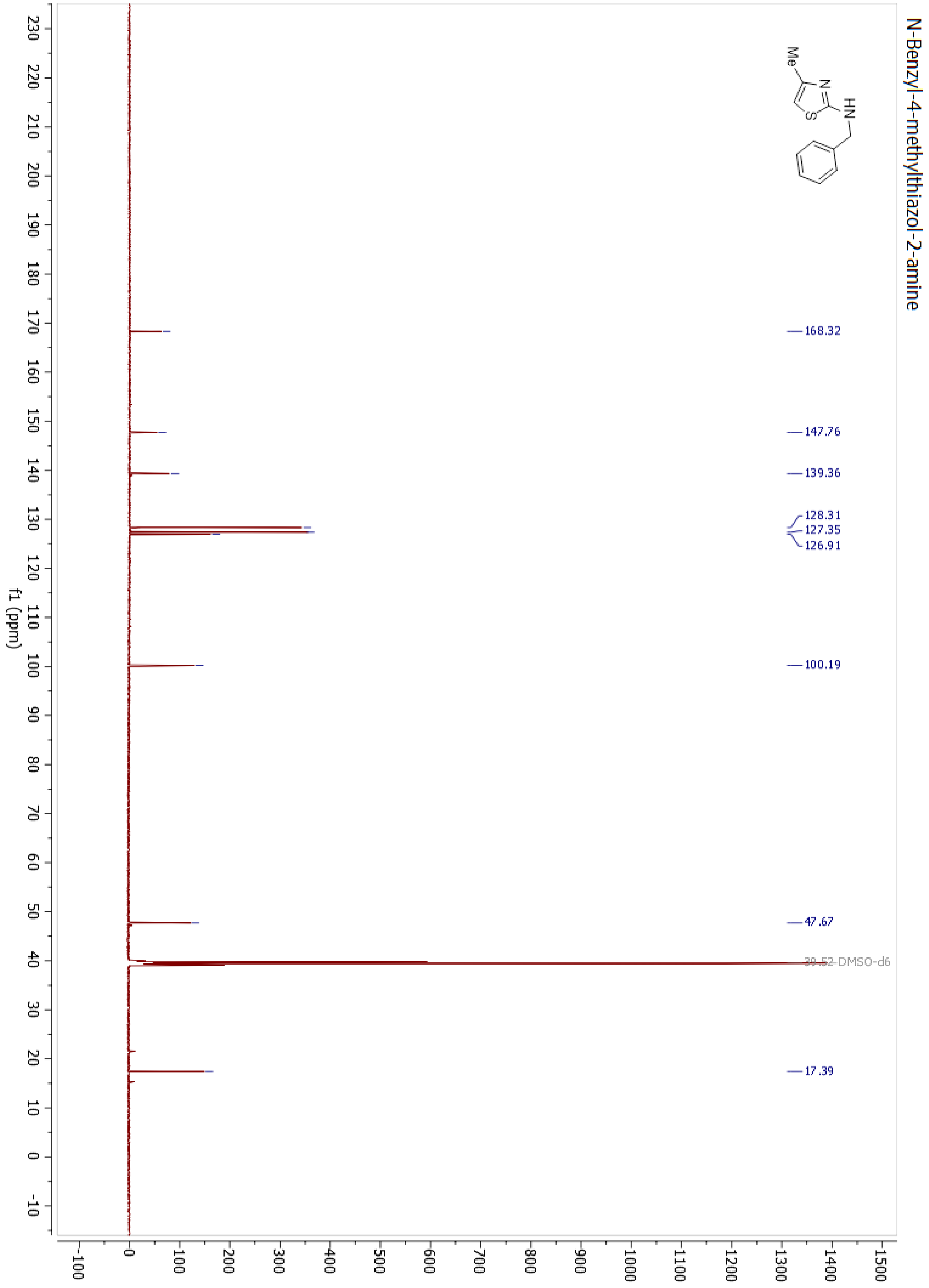

Figure S84. ^{13}C NMR spectrum of **3a’** in d_6-DMSO (151 MHz)

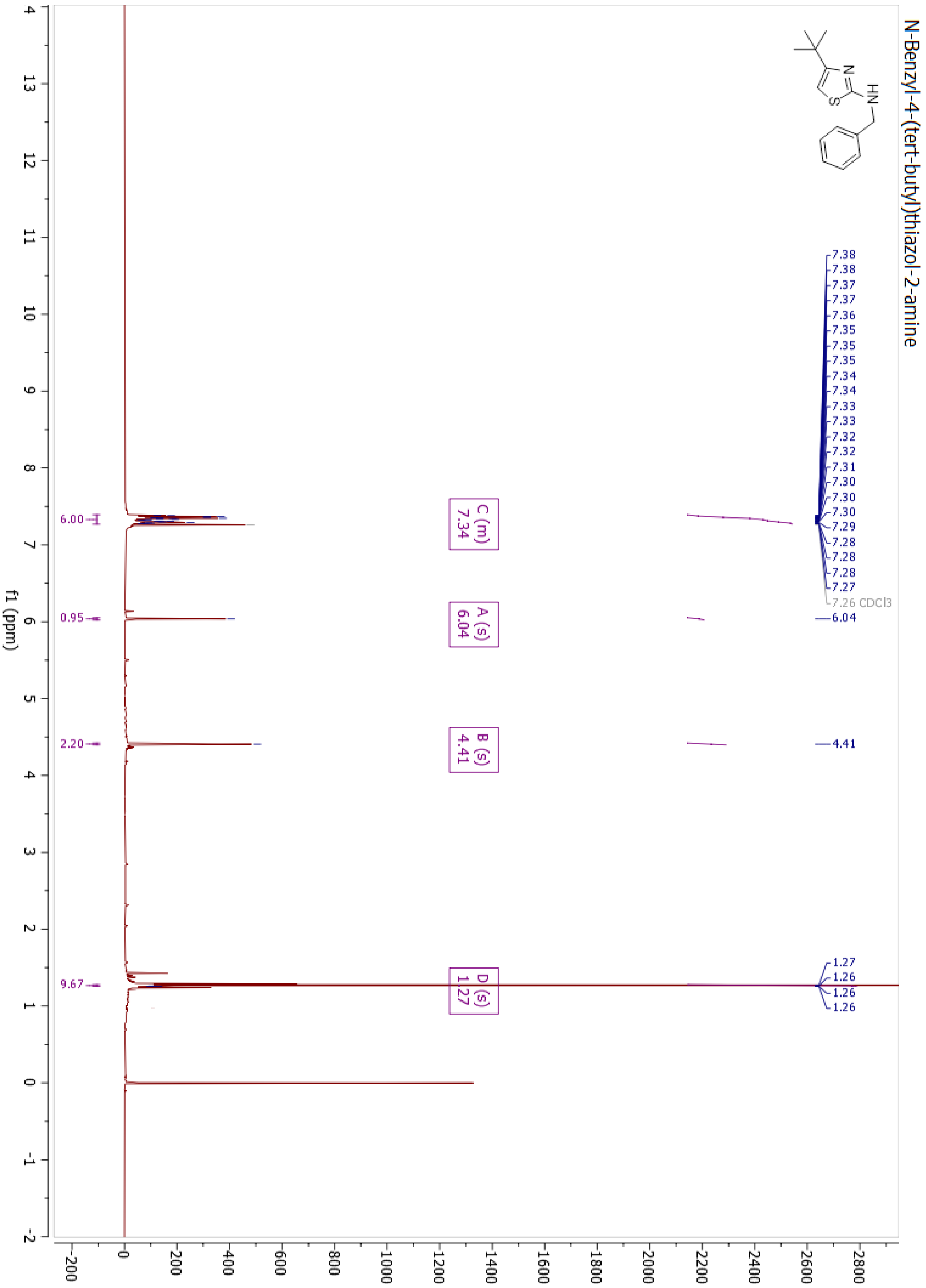

Figure S85. ^{1}H NMR spectrum of **3b’** in d_6-DMSO (600 MHz)

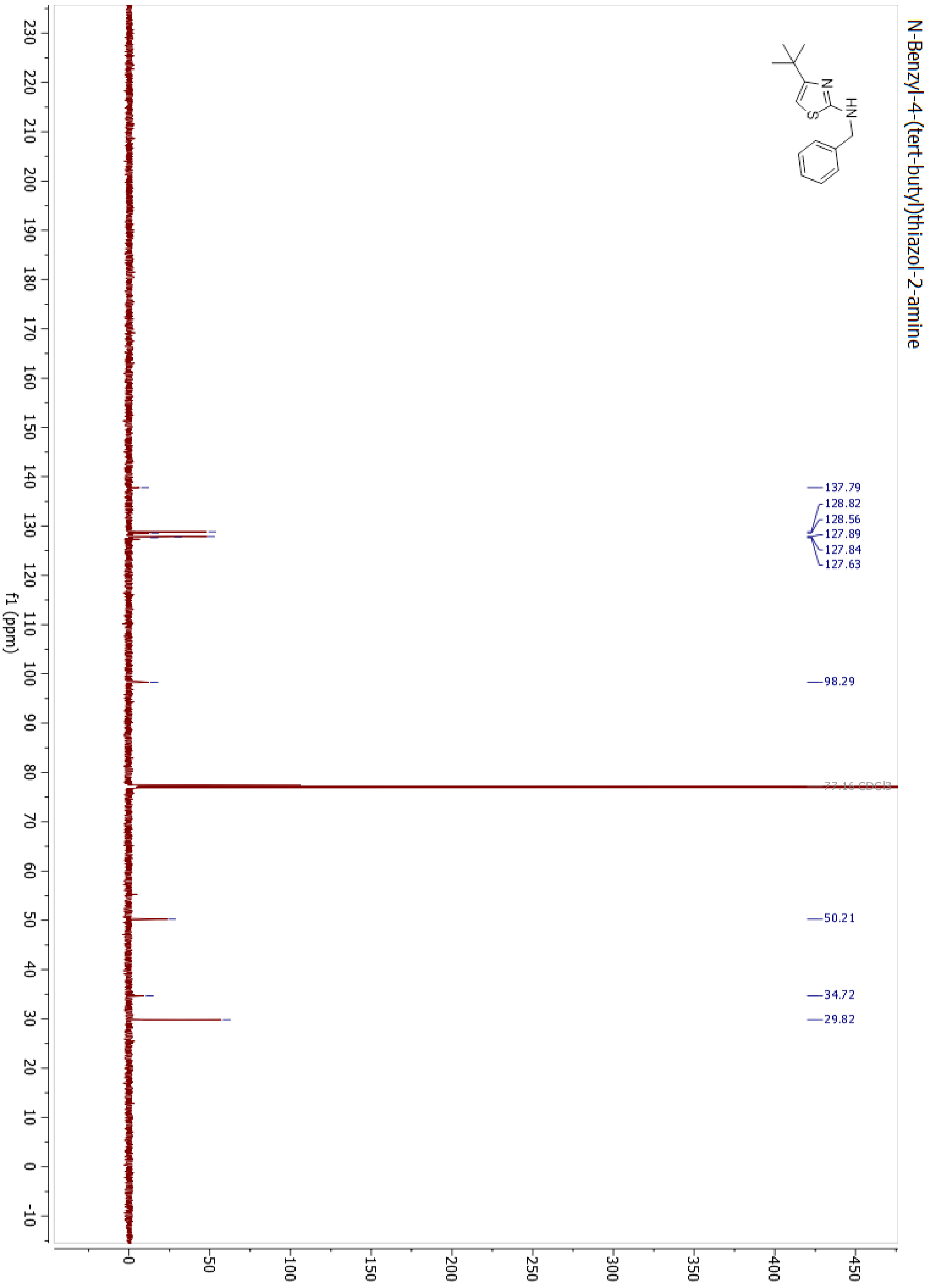

Figure S86. ^{13}C NMR spectrum of **3b’** in d_6-DMSO (151 MHz)

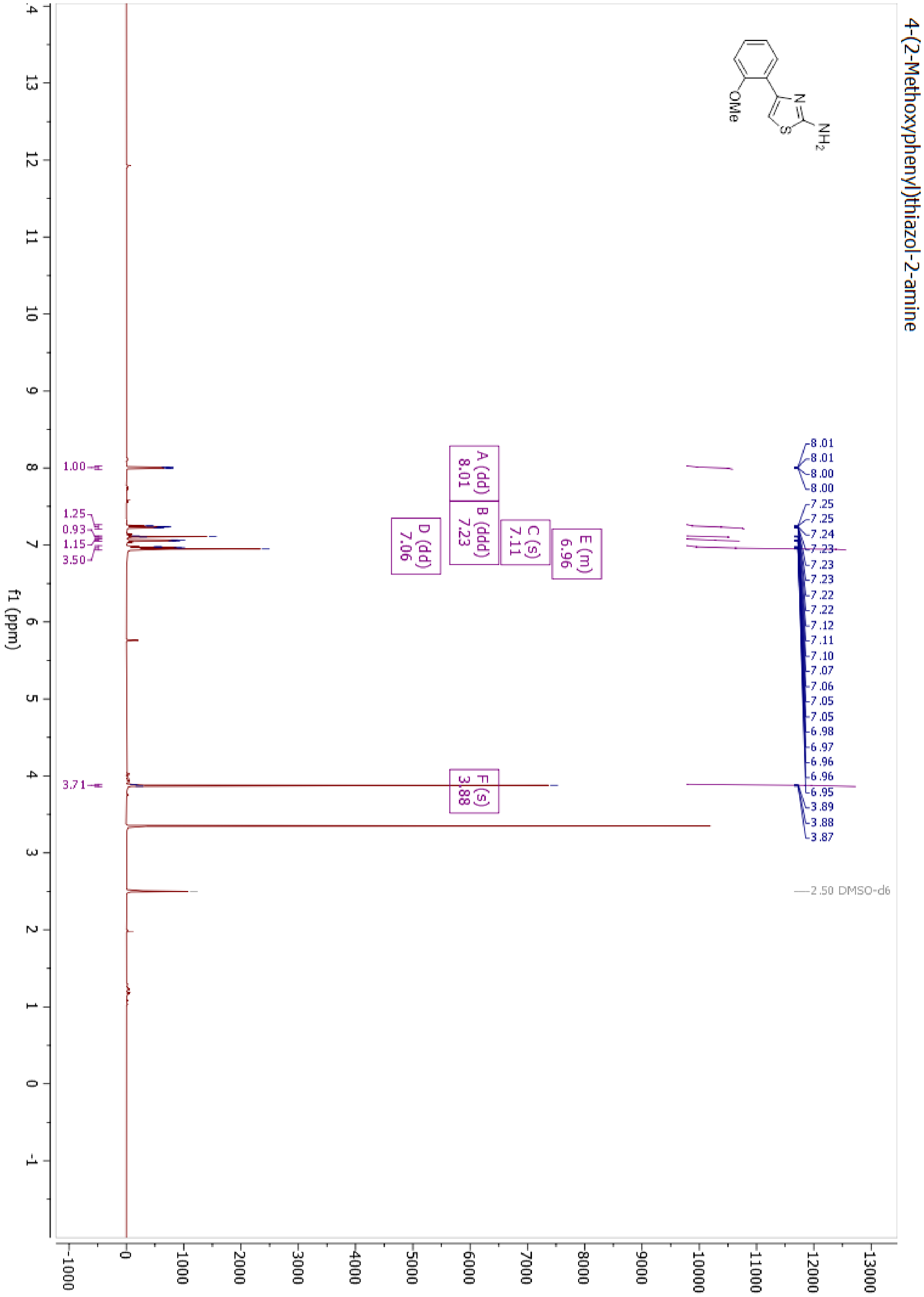

Figure S87. ^{1}H NMR spectrum of **3c'** in d_6-DMSO (600 MHz)

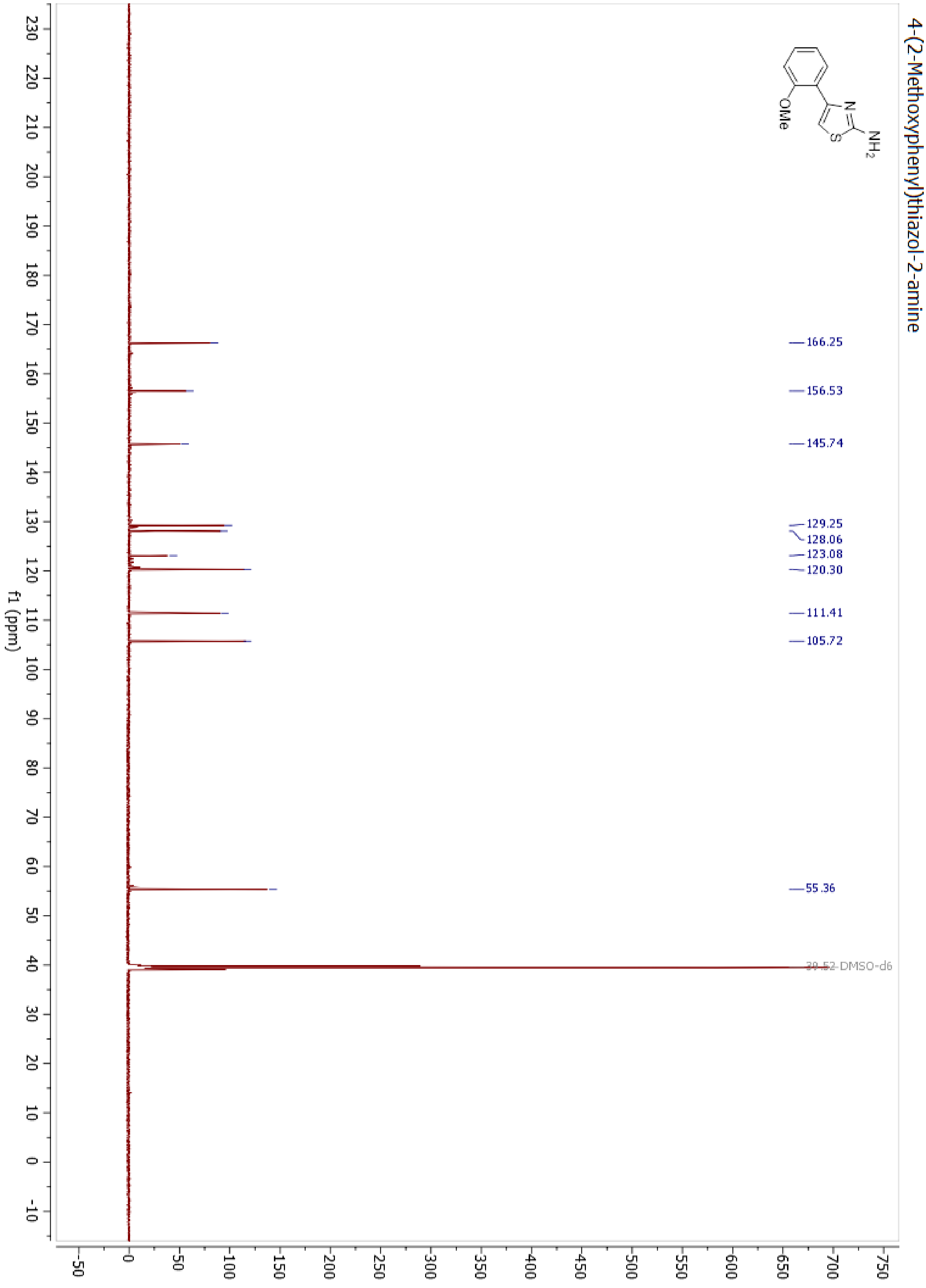

Figure S88. ^{13}C NMR spectrum of **3c’** in d_6-DMSO (151 MHz)

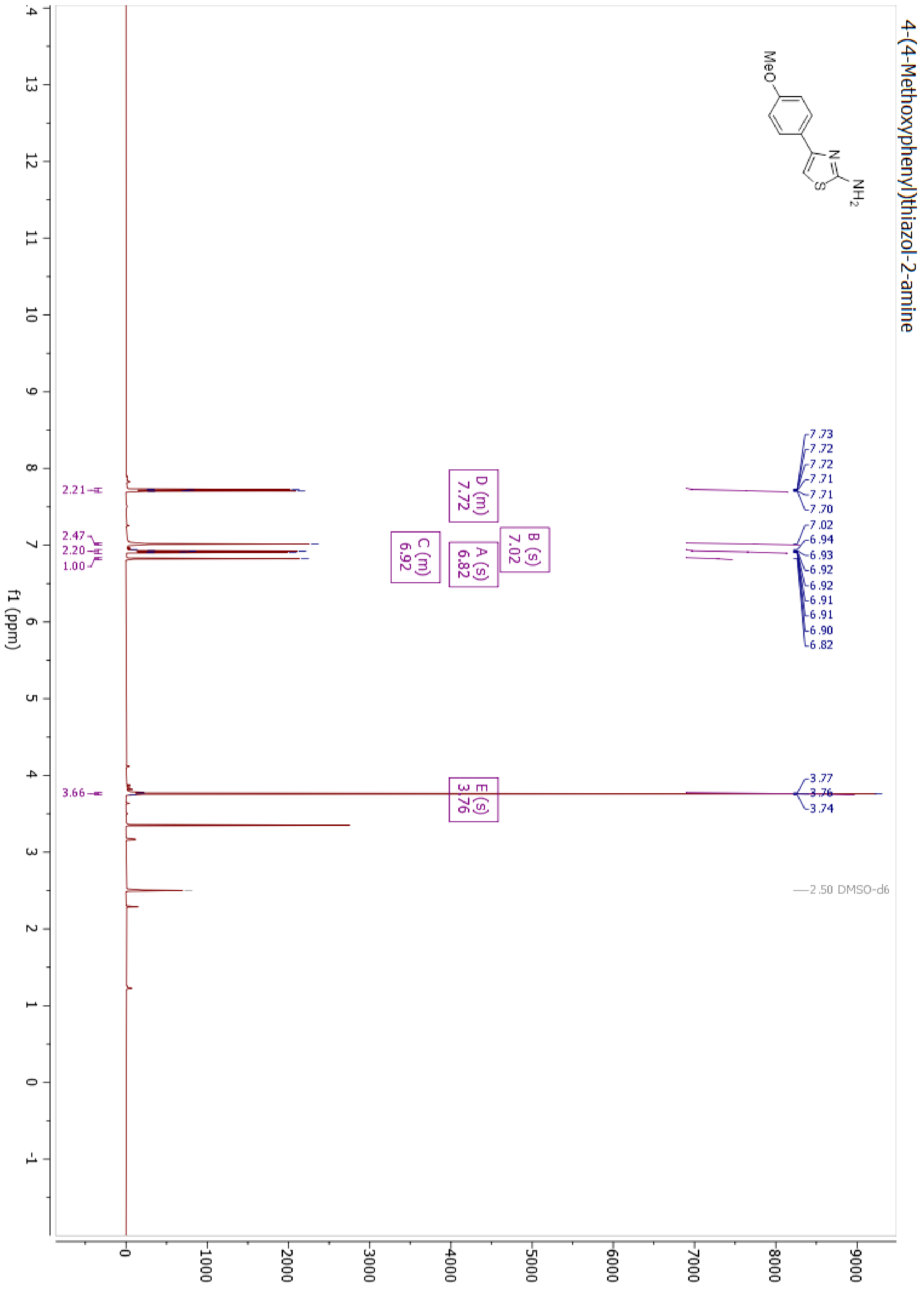

Figure S89. ^{1}H NMR spectrum of **3d’** in d_6-DMSO (600 MHz)

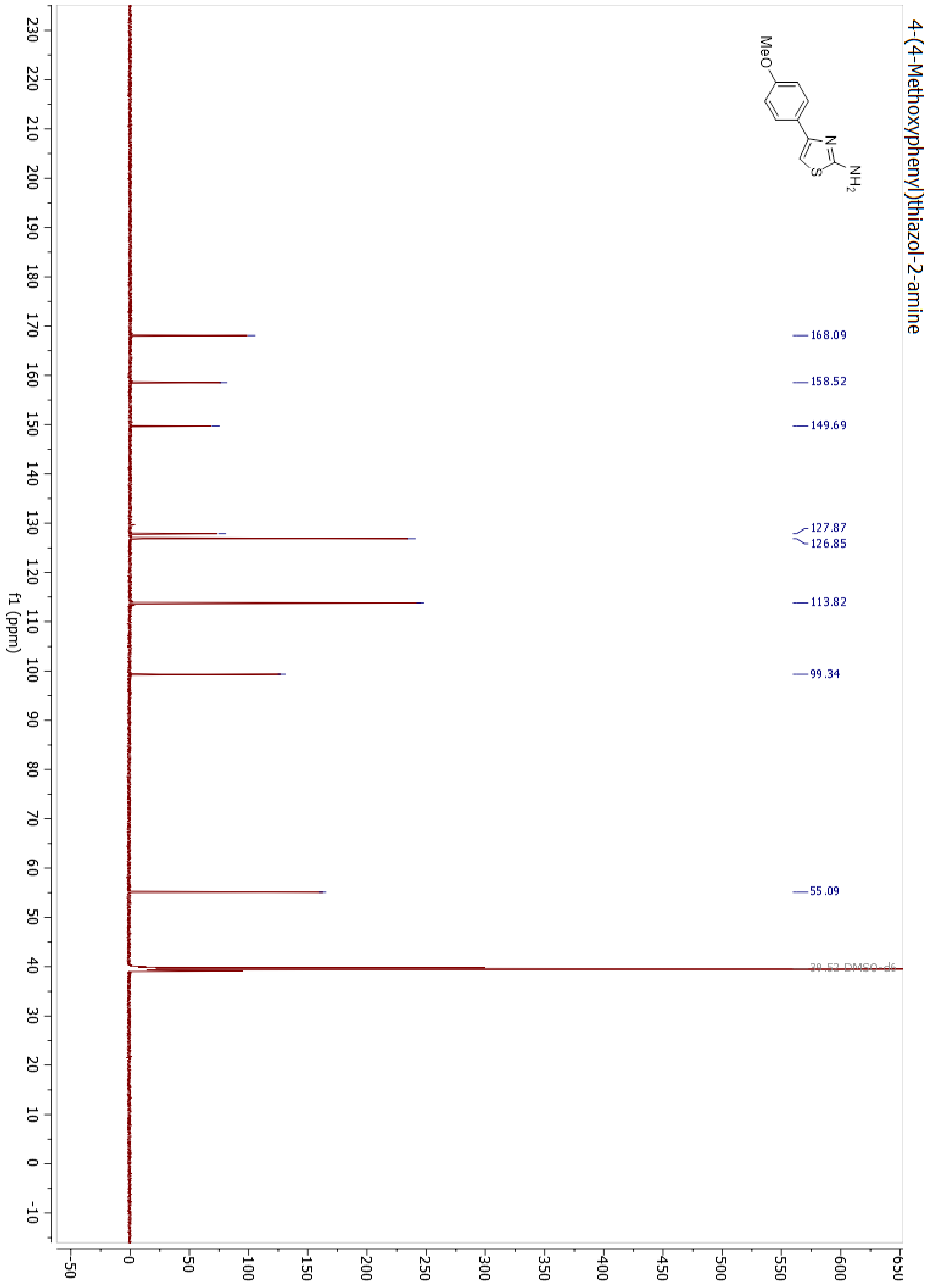

Figure S90. ^{13}C NMR spectrum of **3d’** in d_6-DMSO (151 MHz)

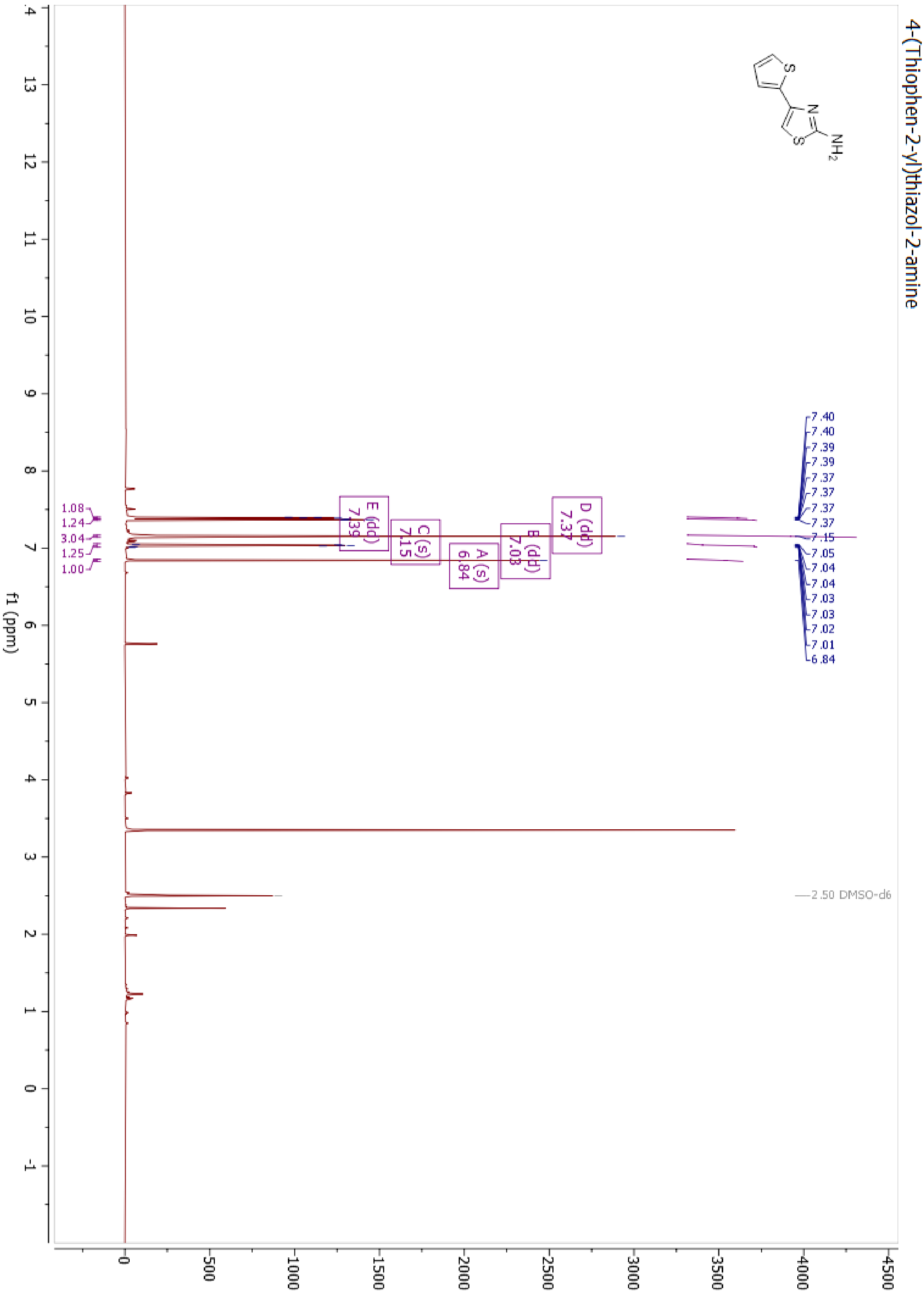

Figure S91. ^{1}H NMR spectrum of **3e’** in d_6-DMSO (600 MHz)

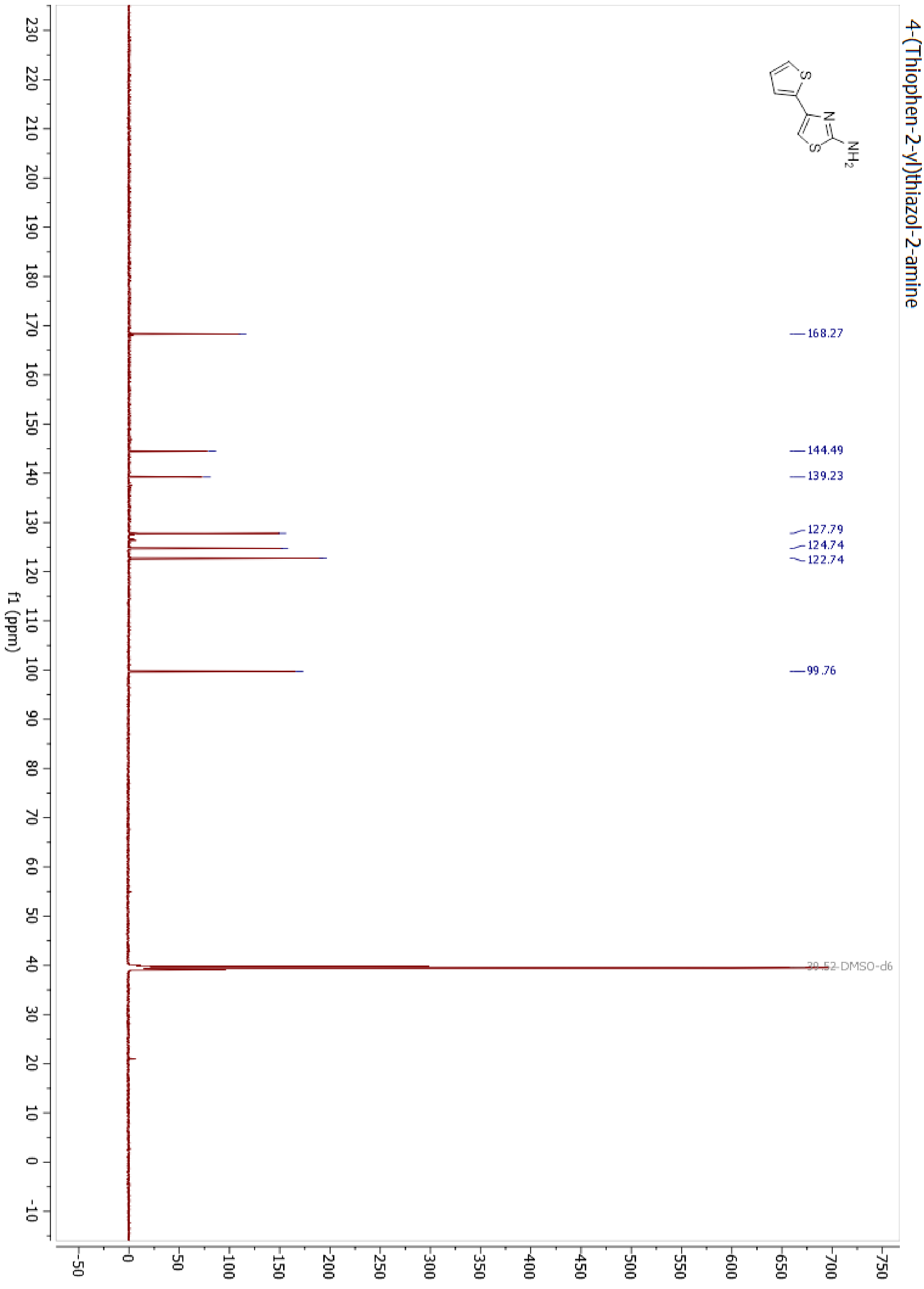

Figure S92. ^{13}C NMR spectrum of **3e’** in d_6-DMSO (151 MHz)

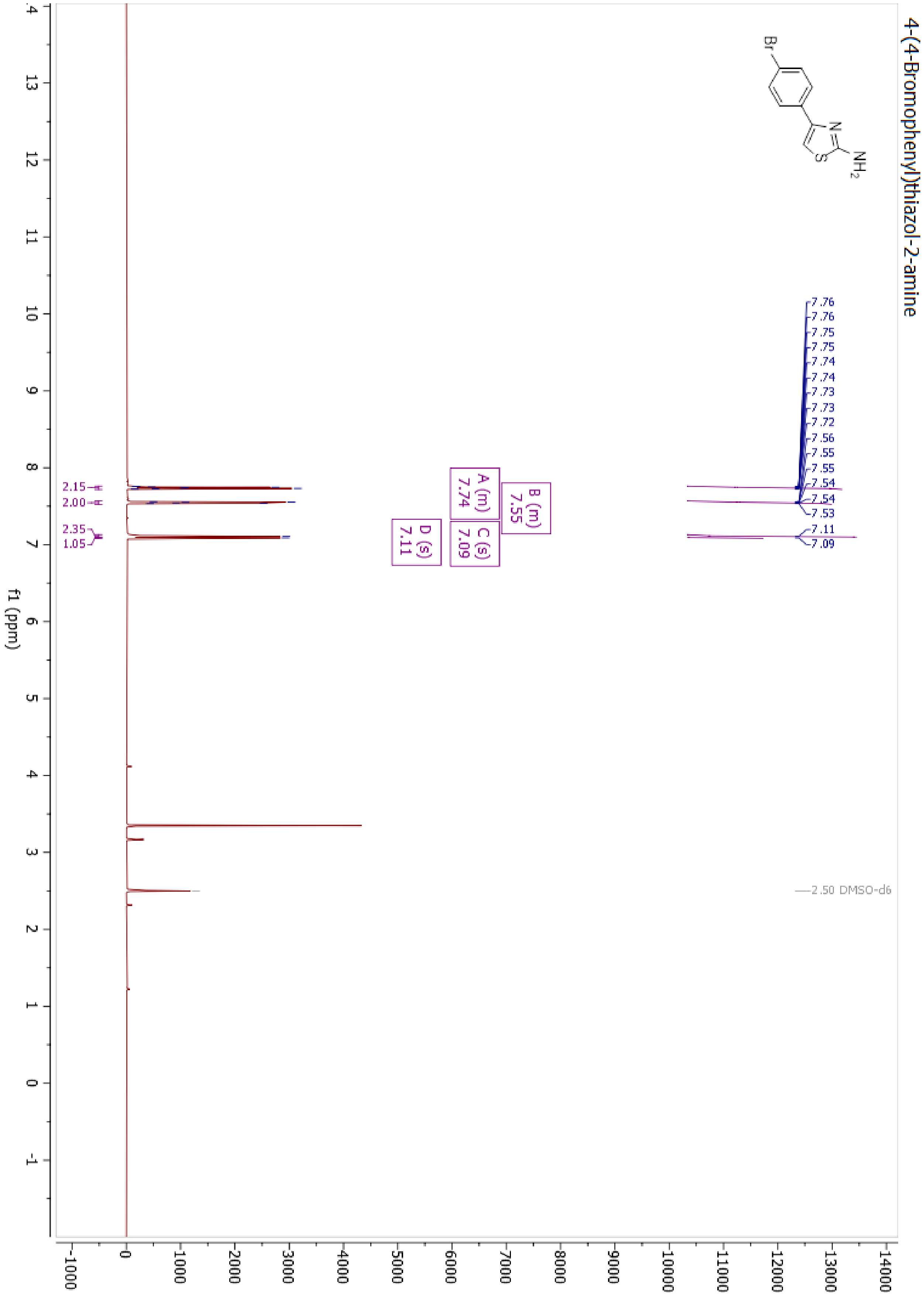

Figure S93. ^{1}H NMR spectrum of **3f'** in d_6-DMSO (600 MHz)

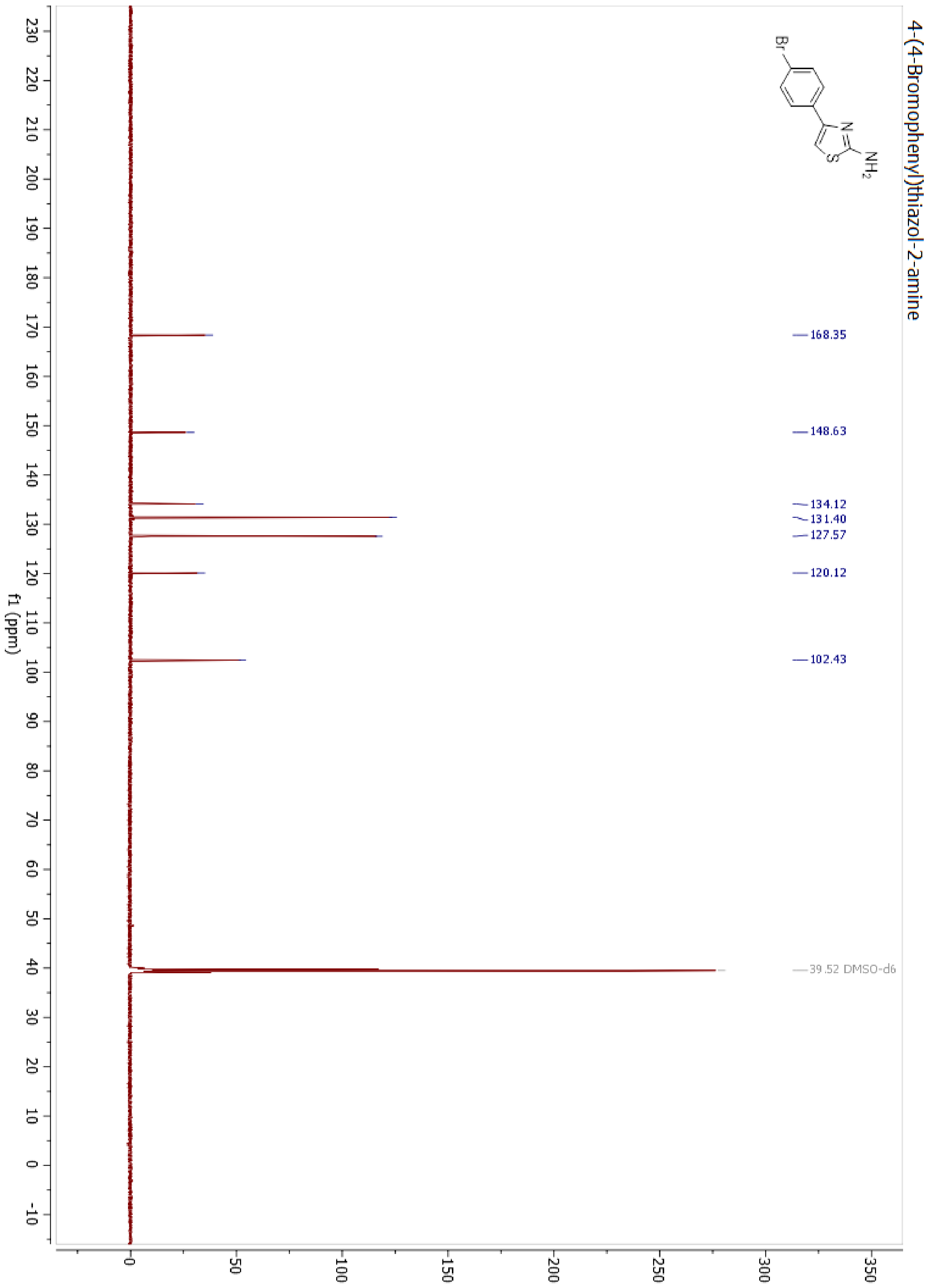

Figure S94. ^{13}C NMR spectrum of **3f’** in d_6-DMSO (151 MHz)

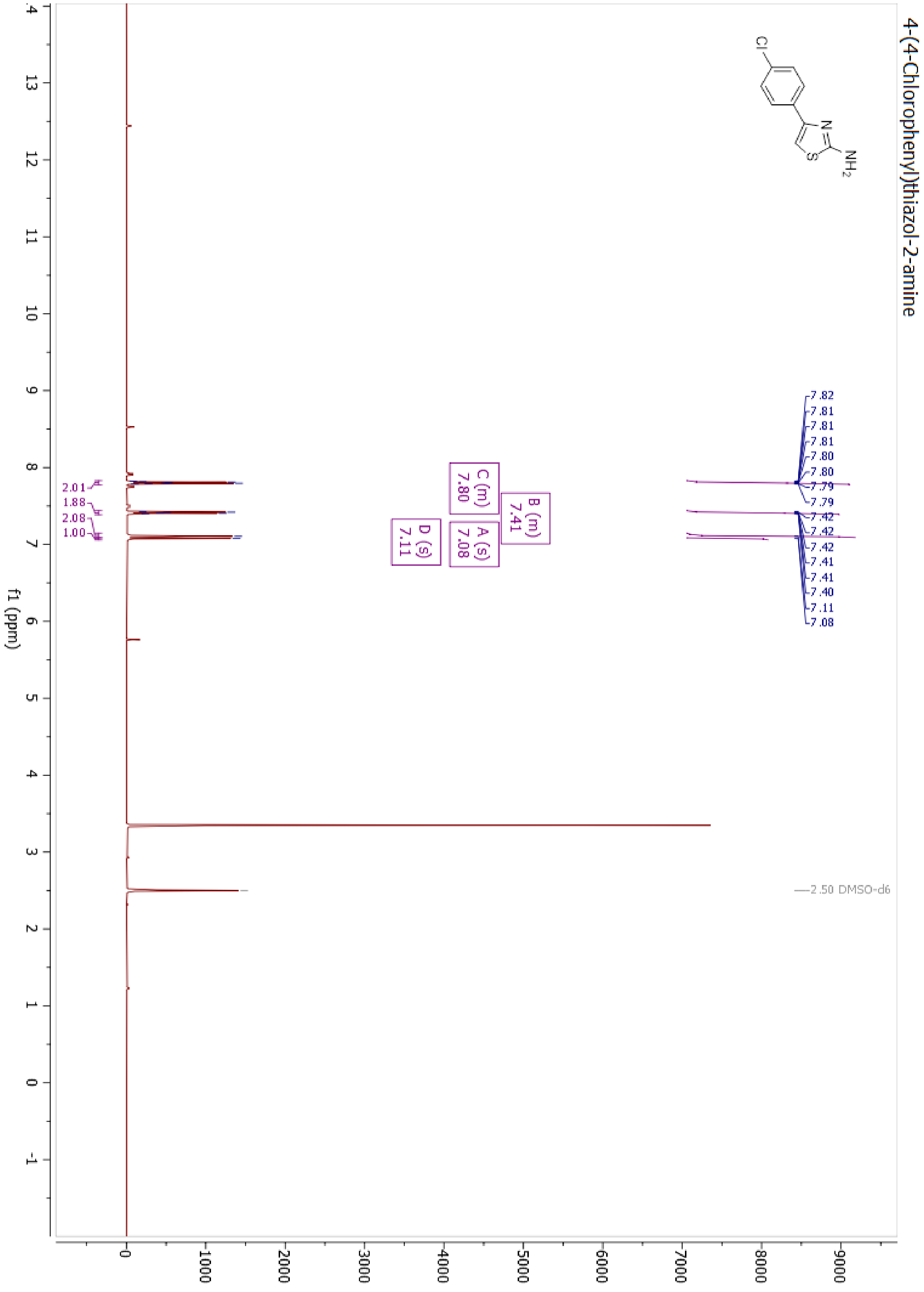

Figure S95. ^{1}H NMR spectrum of **3g’** in d_6-DMSO (600 MHz)

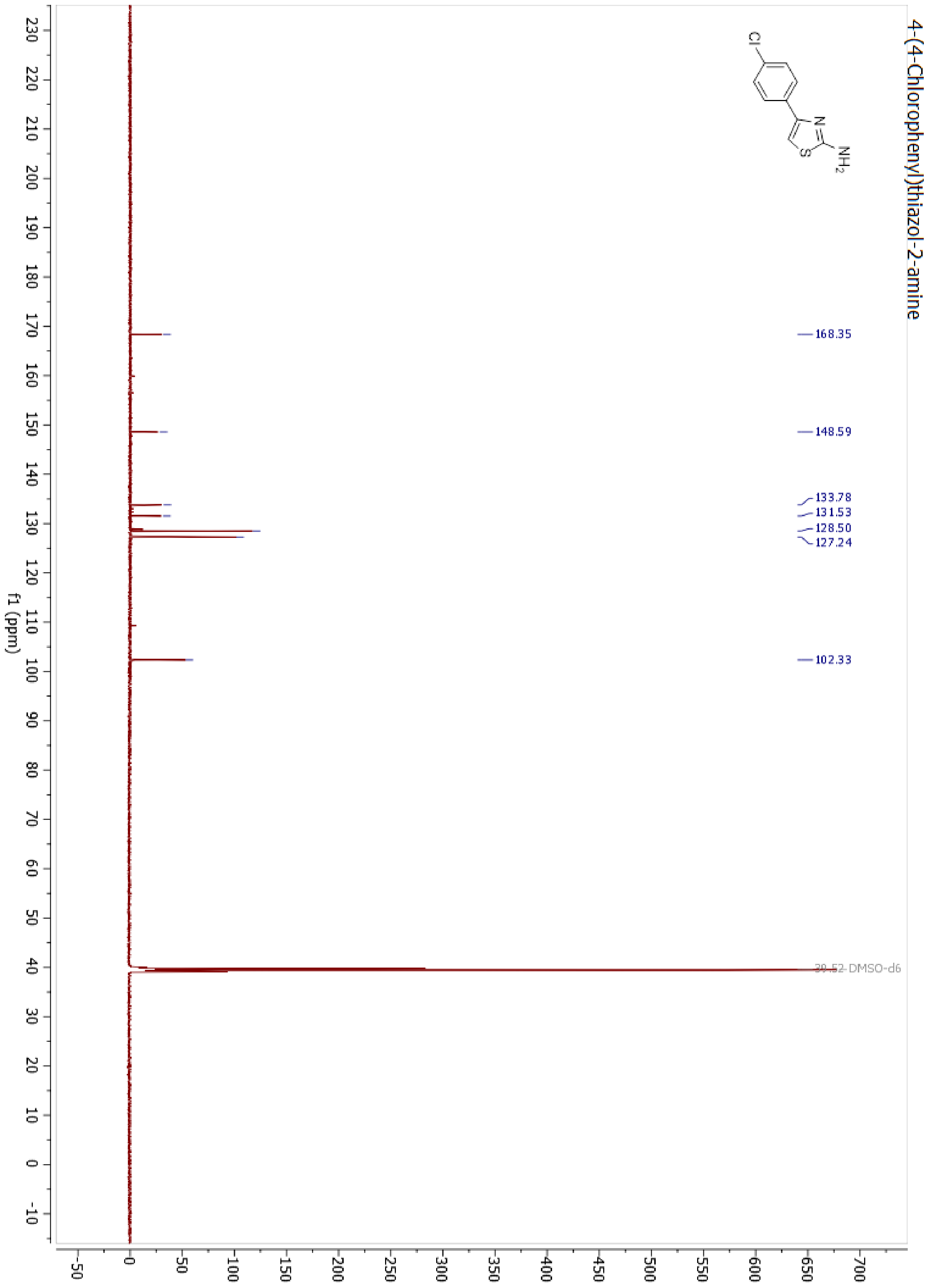

Figure S96. ^{13}C NMR spectrum of **3g’** in d_6-DMSO (151 MHz)

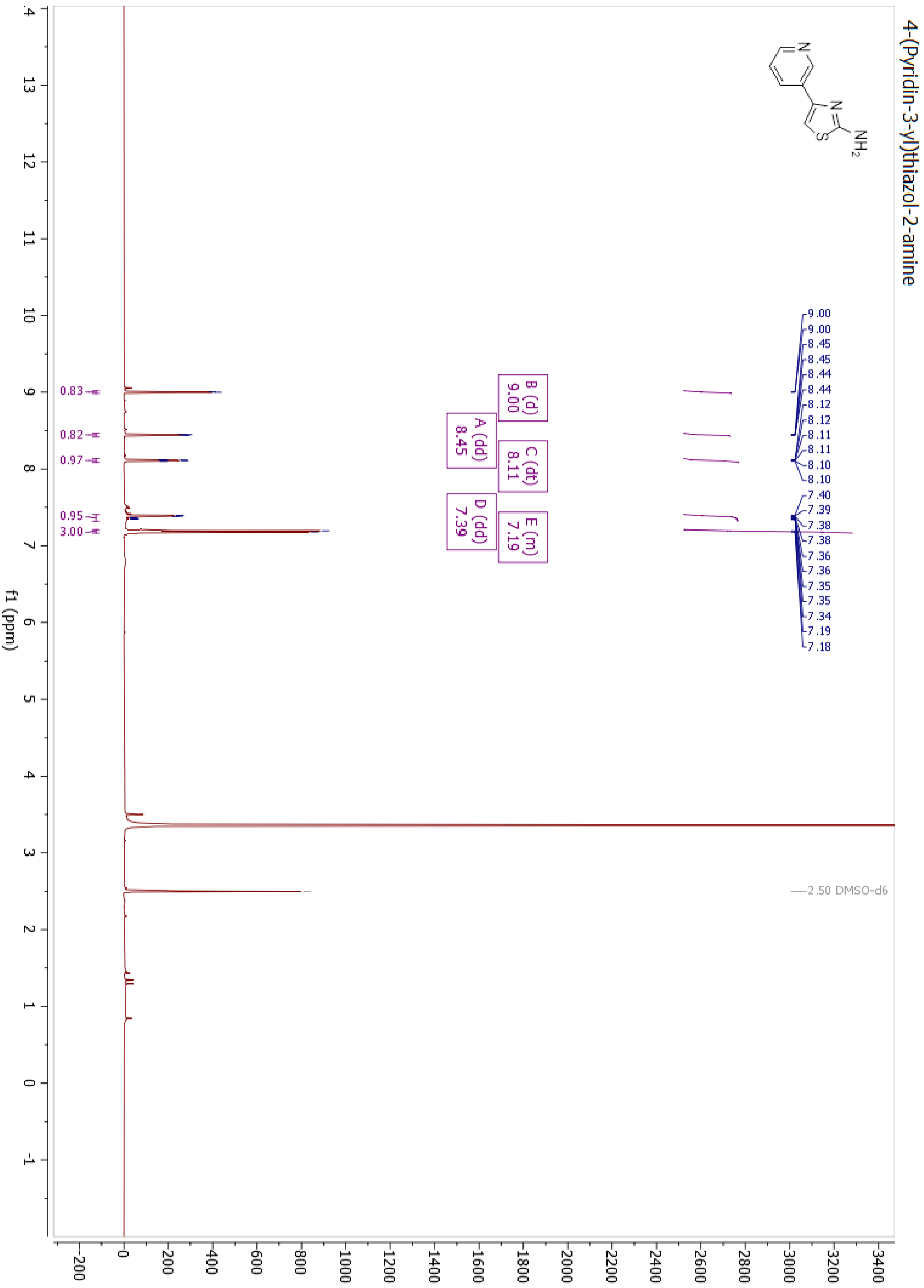

Figure S97. ^{1}H NMR spectrum of **3h'** in d_6-DMSO (600 MHz)

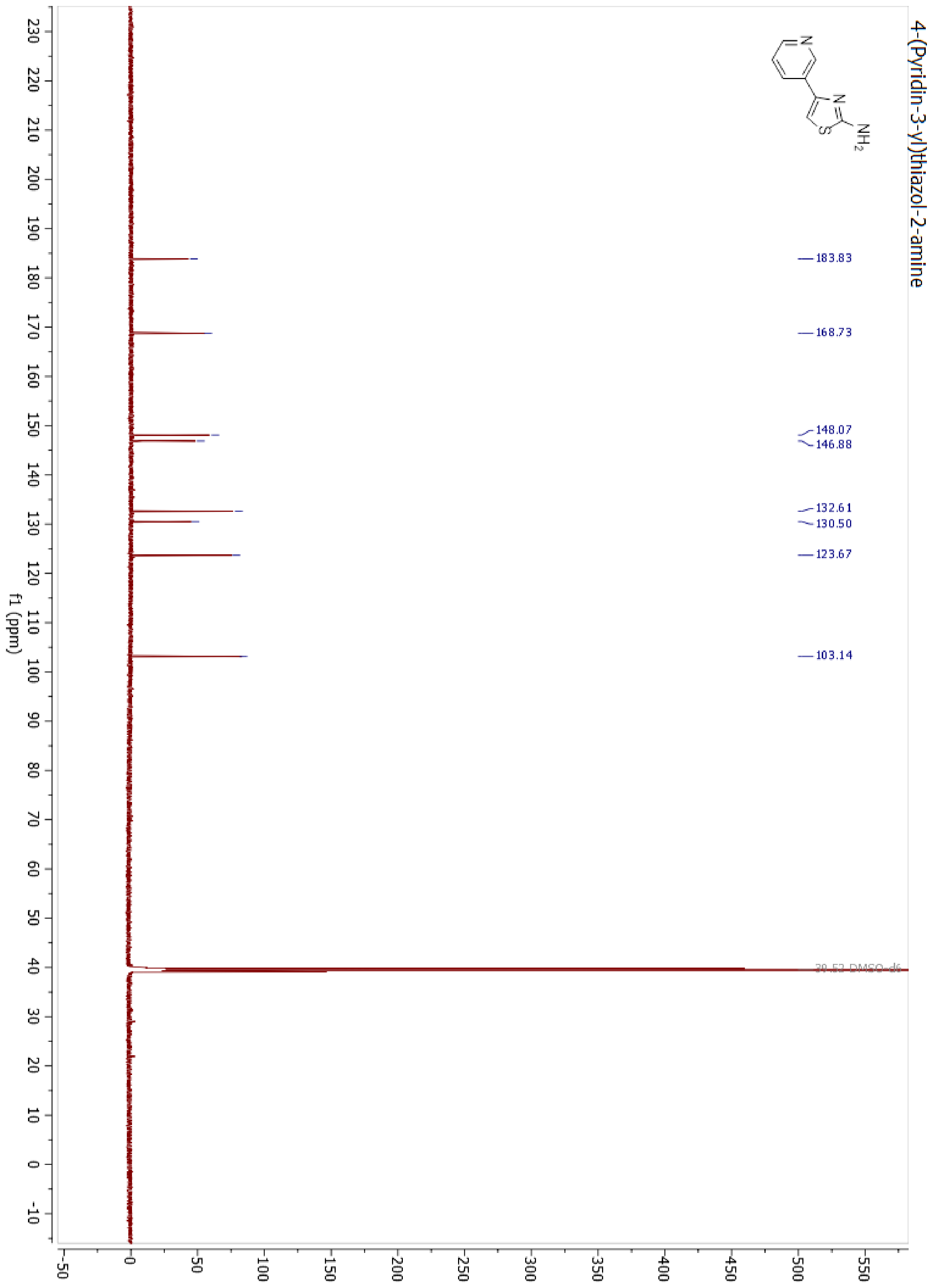

Figure S98. ^{13}C NMR spectrum of **3h’** in d_6-DMSO (151 MHz)

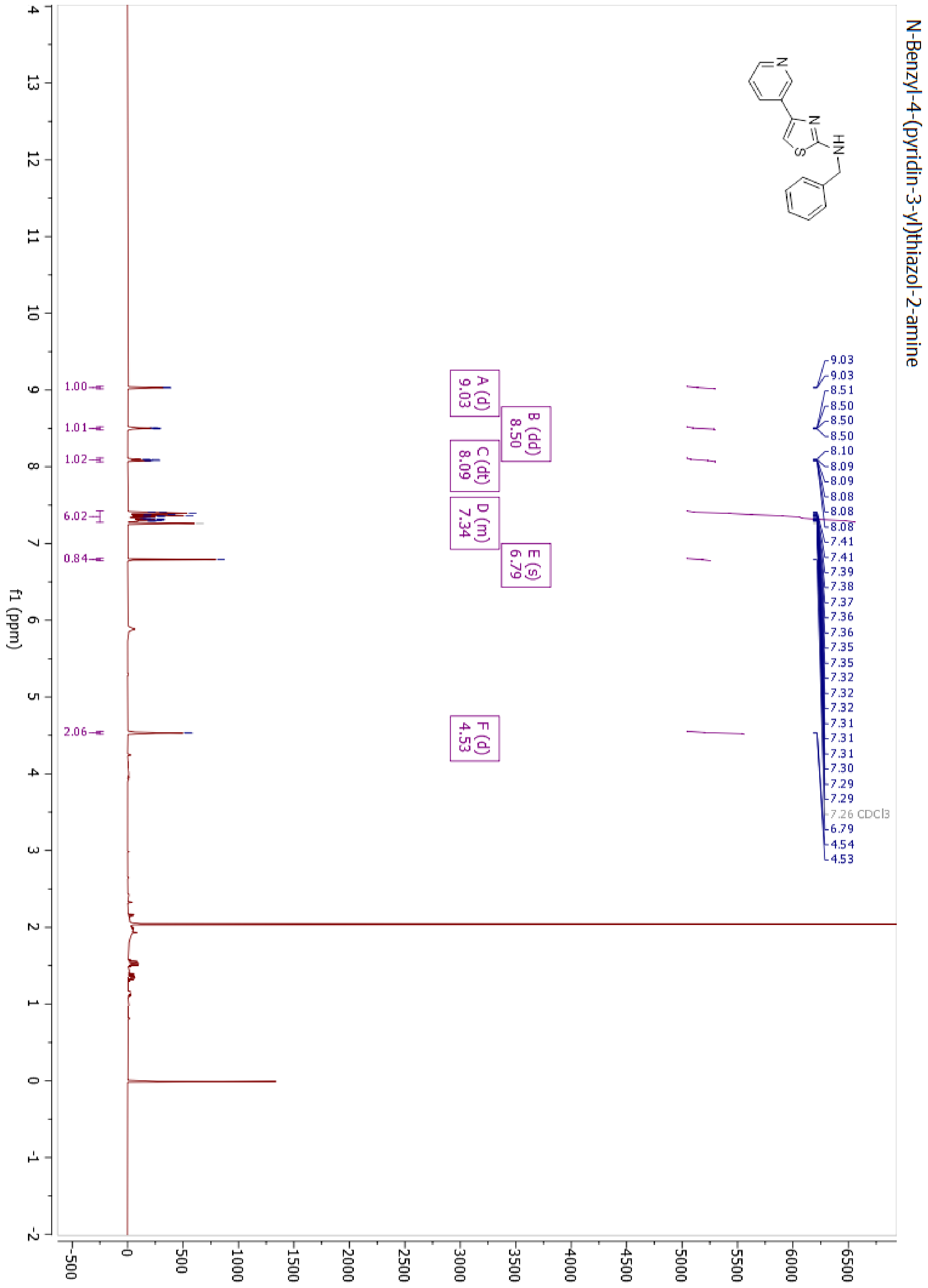

Figure S99. ^{1}H NMR spectrum of **3i’** in d_6-DMSO (600 MHz)

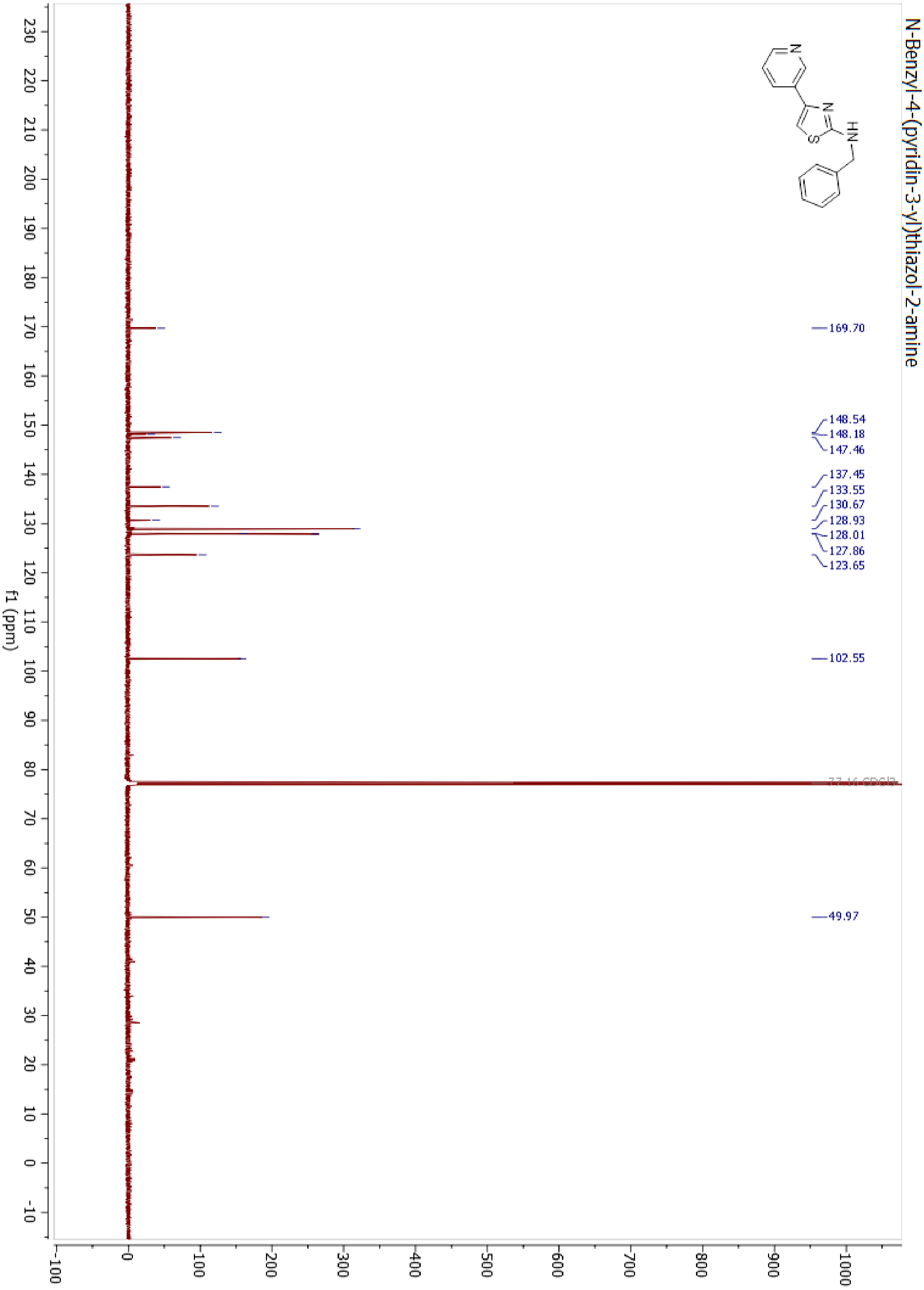

Figure S100. ^{13}C NMR spectrum of **3i’** in d_6-DMSO (151 MHz)

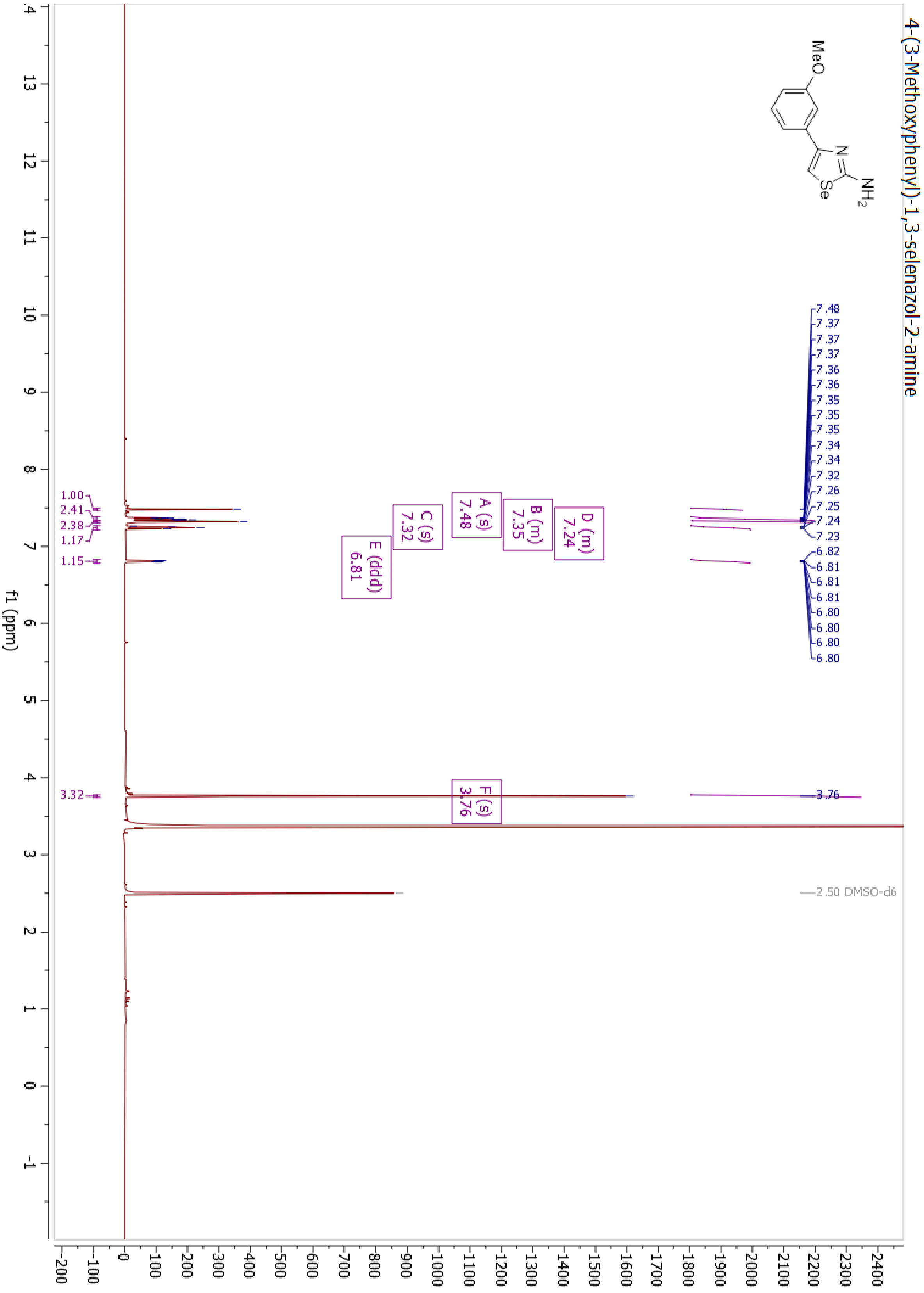

Figure S101. ^{1}H NMR spectrum of **7a** in d_6-DMSO (600 MHz)

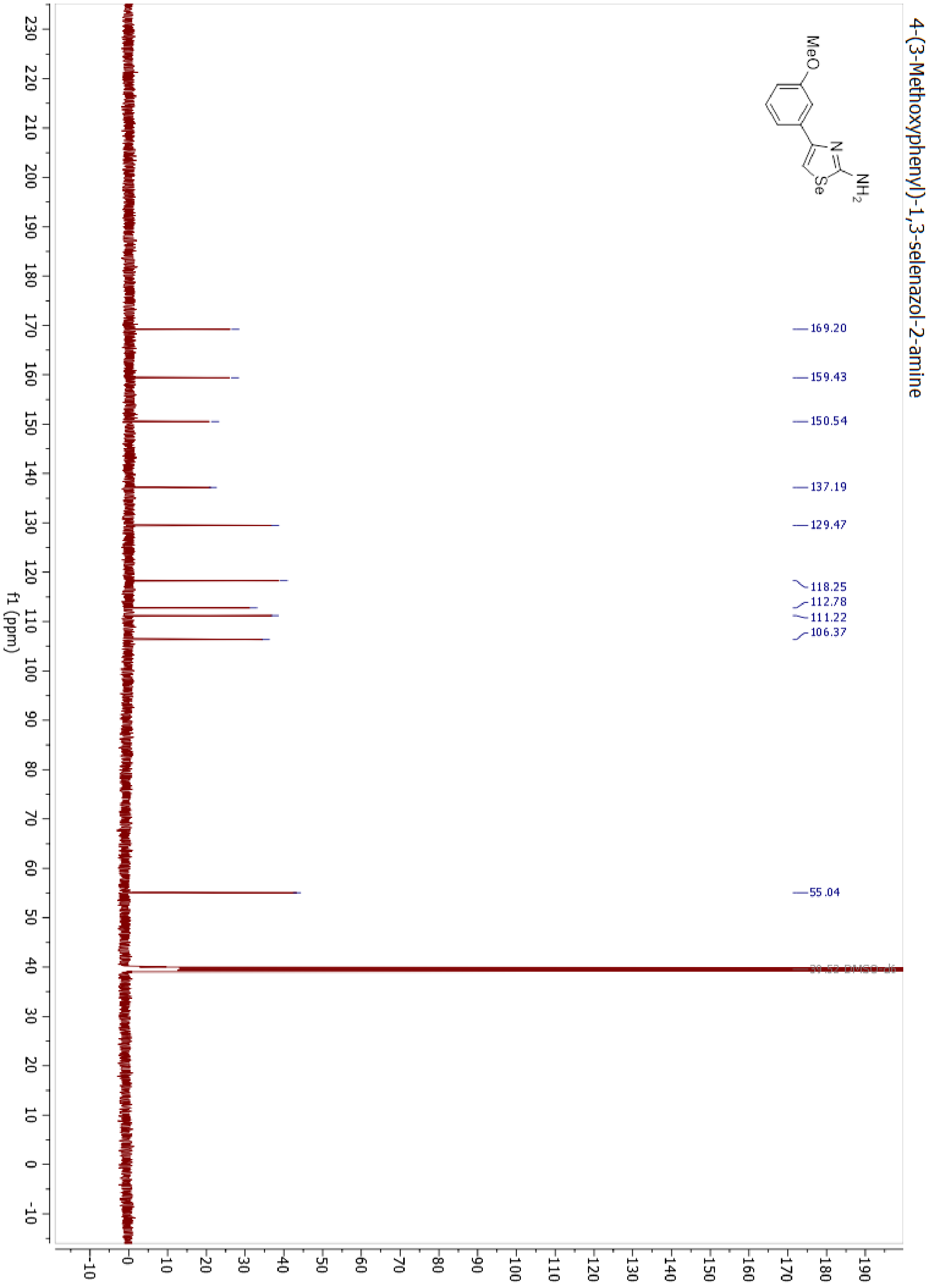

Figure S102. ^{13}C NMR spectrum of **7a** in d_6-DMSO (151 MHz)

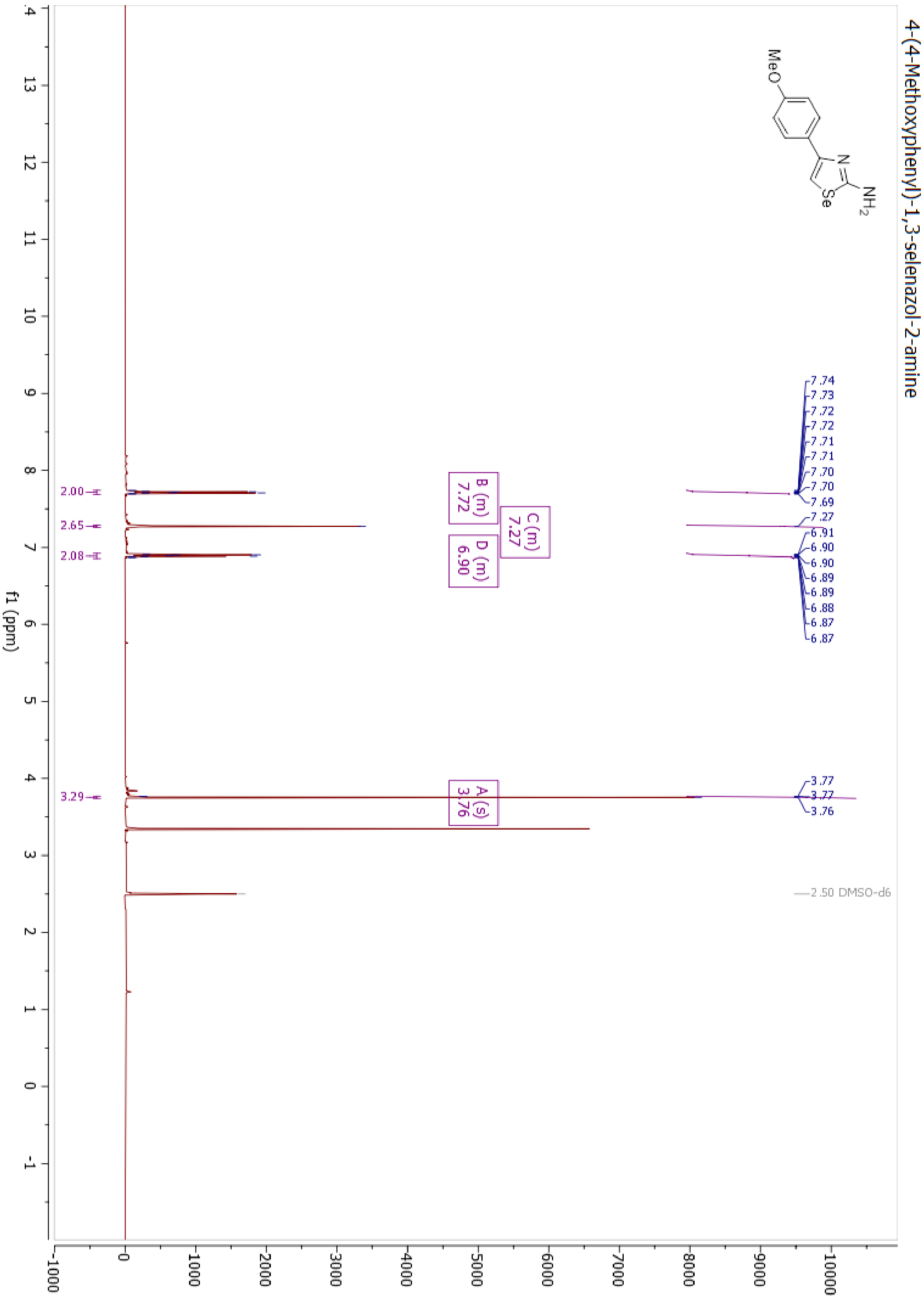

Figure S103. ^{1}H NMR spectrum of **7b** in d_6-DMSO (600 MHz)

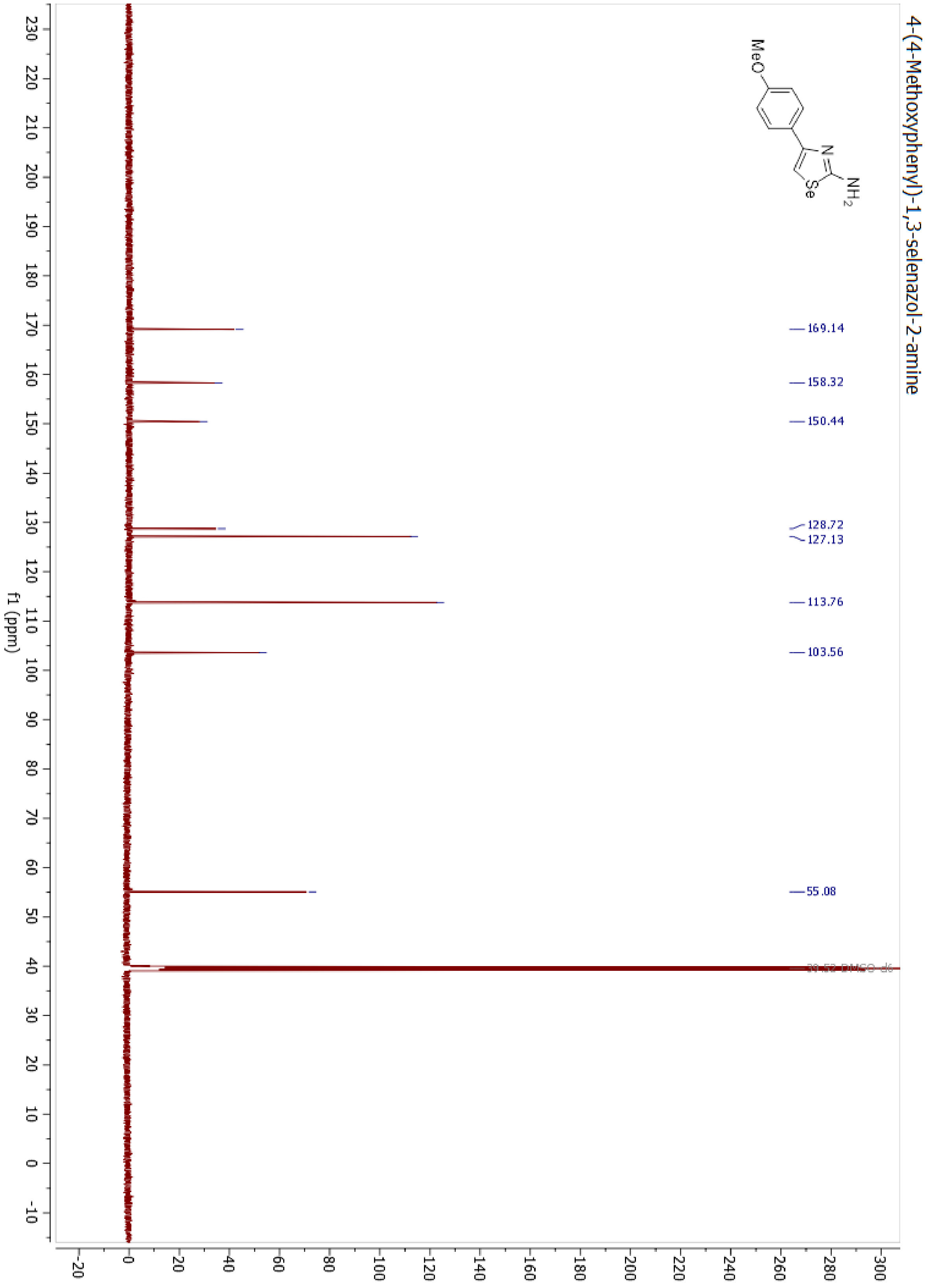

Figure S104. ^{13}C NMR spectrum of **7b** in d_6-DMSO (151 MHz)

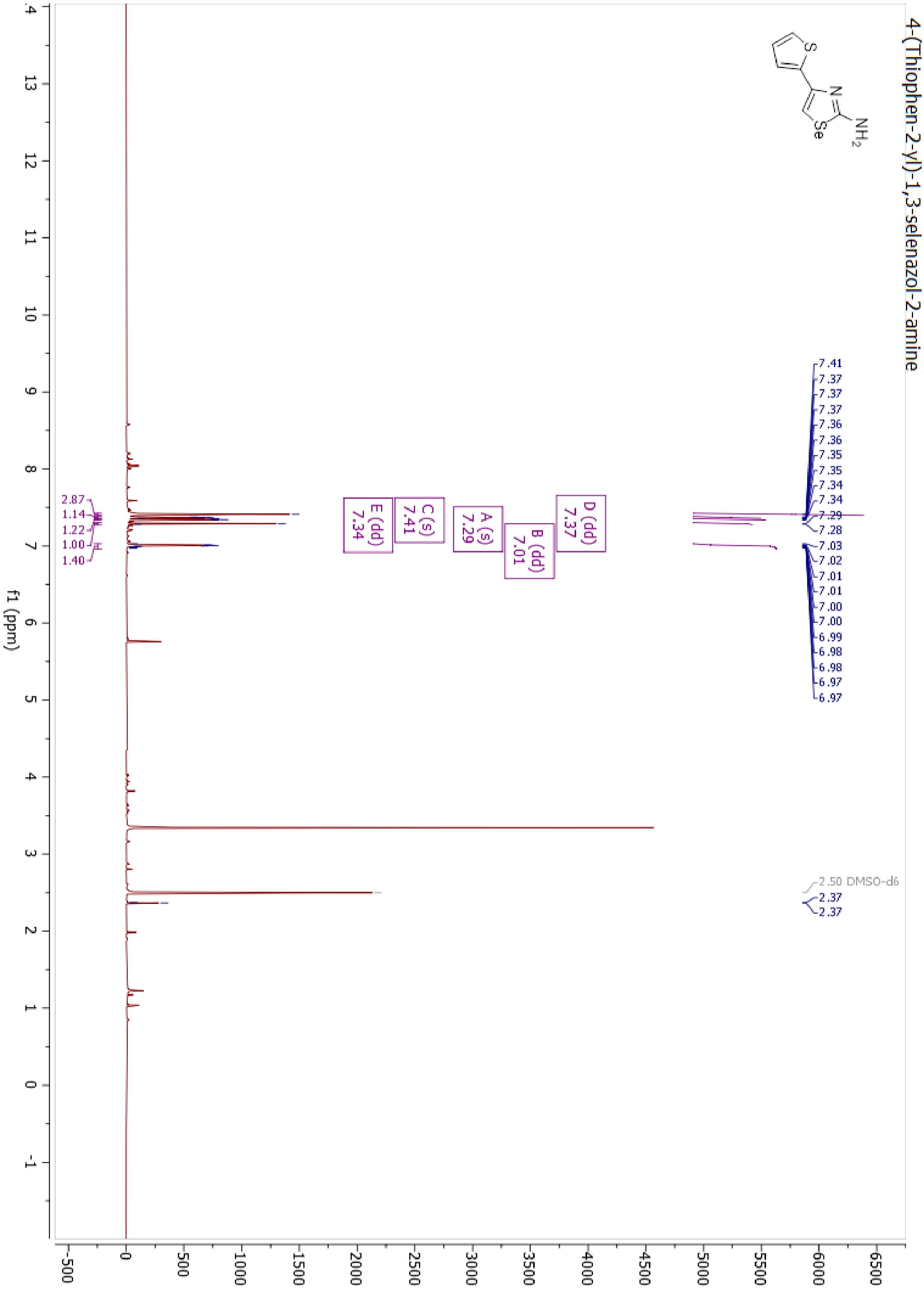

Figure S105. ^{1}H NMR spectrum of **7c** in d_6-DMSO (600 MHz)

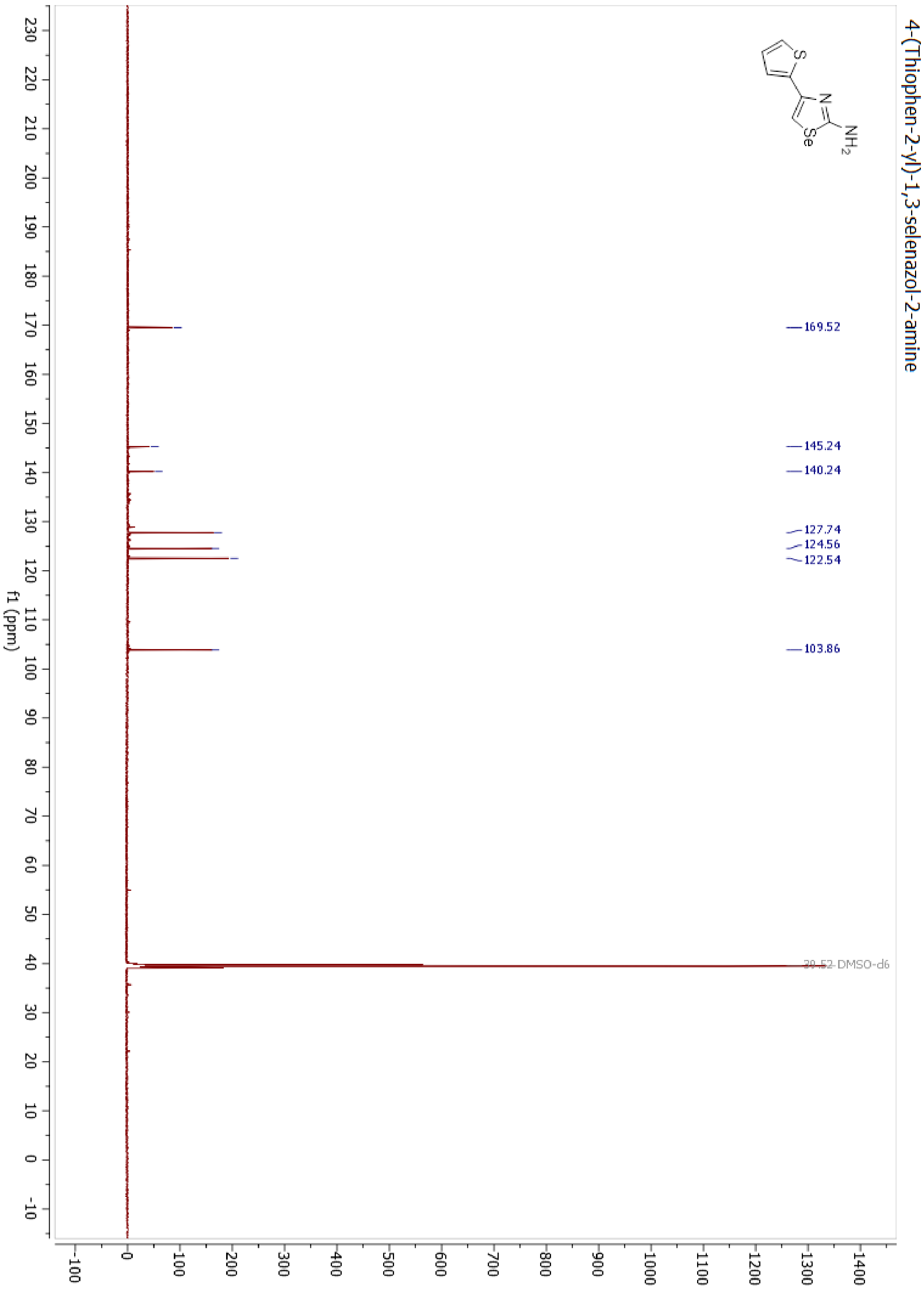

Figure S106. ^{13}C NMR spectrum of **7c** in d_6-DMSO (151 MHz)

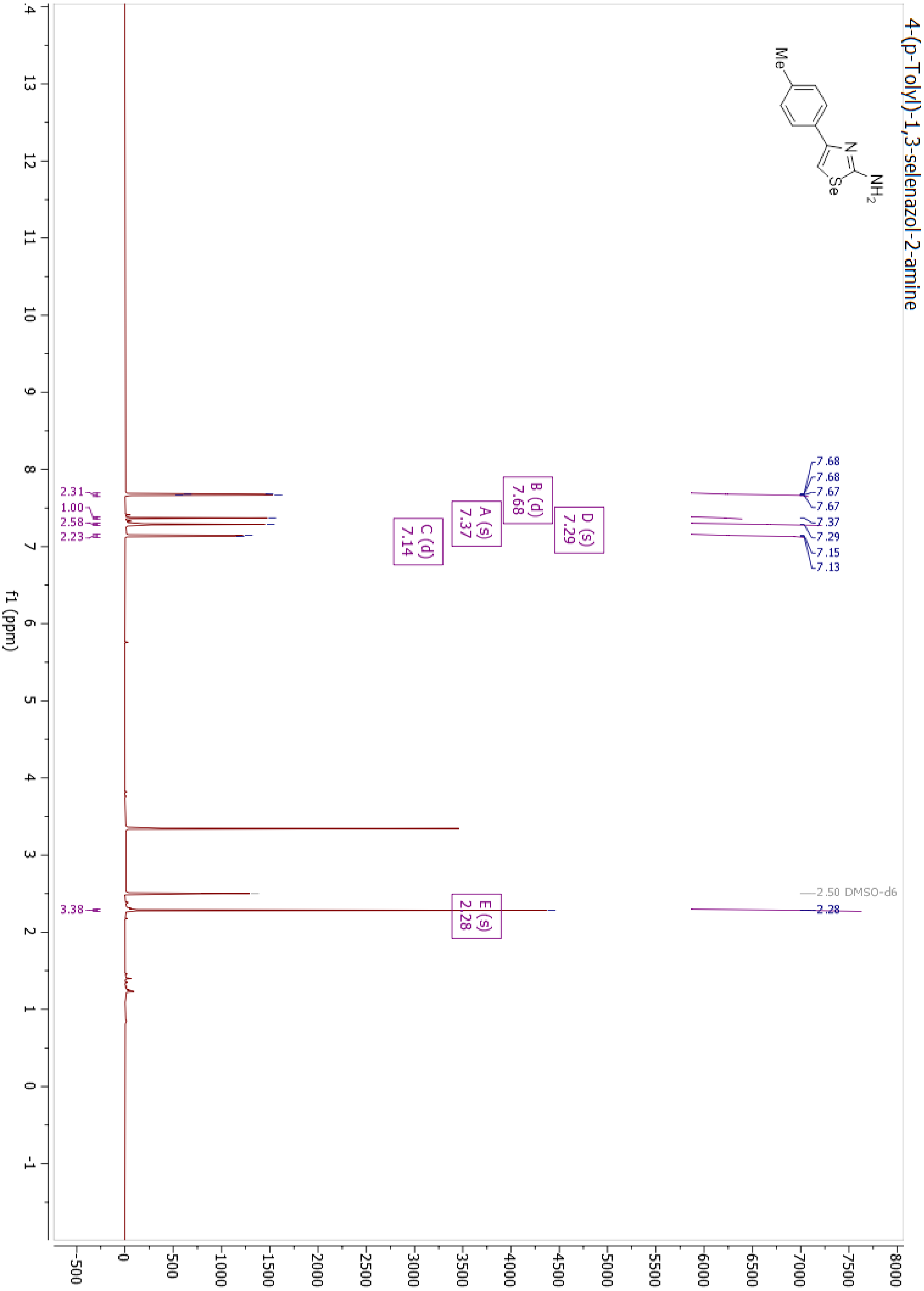

Figure S107. ^{1}H NMR spectrum of **7d** in d_6-DMSO (600 MHz)

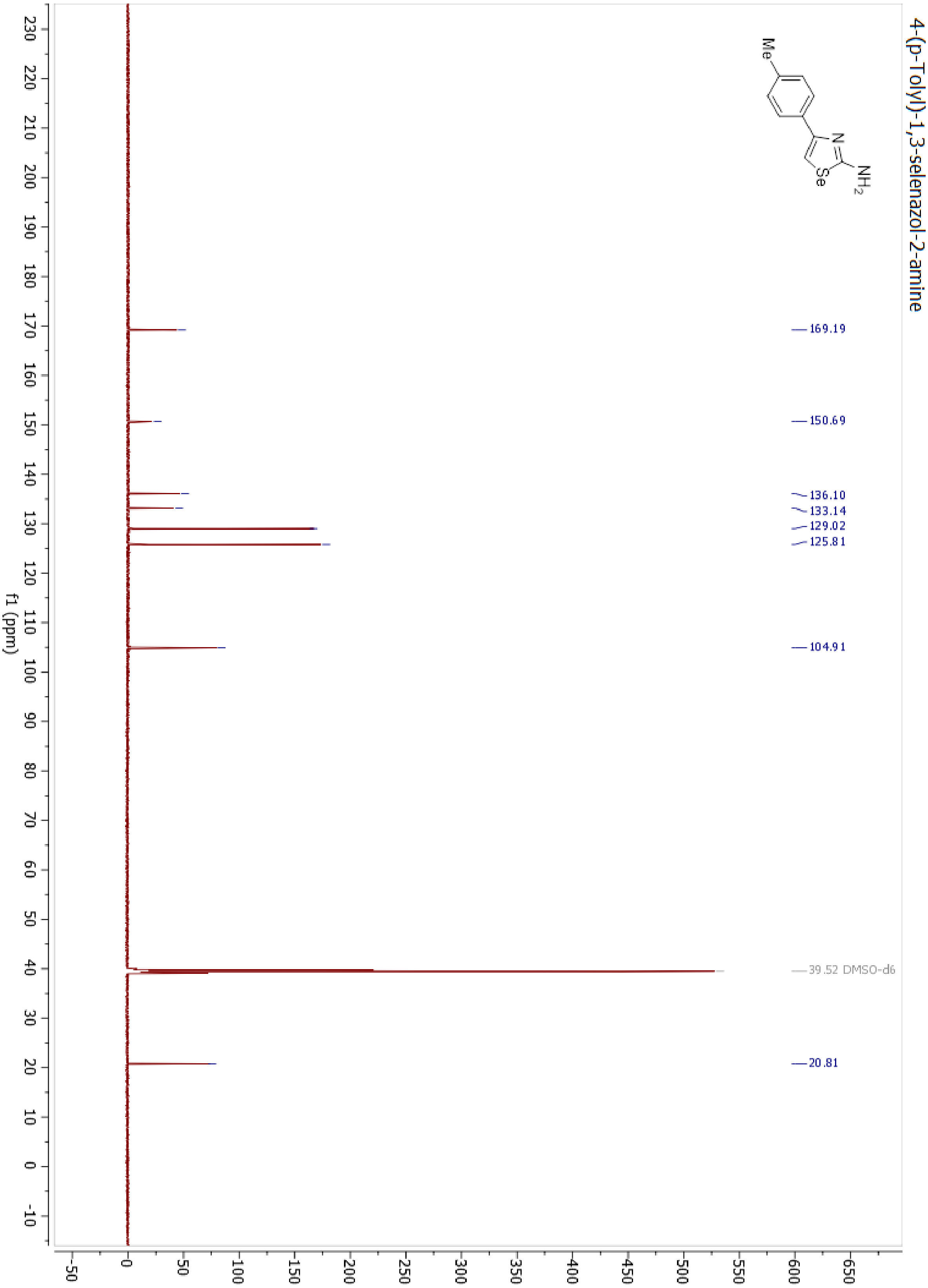

Figure S108. ^{13}C NMR spectrum of **7d** in d_6-DMSO (151 MHz)

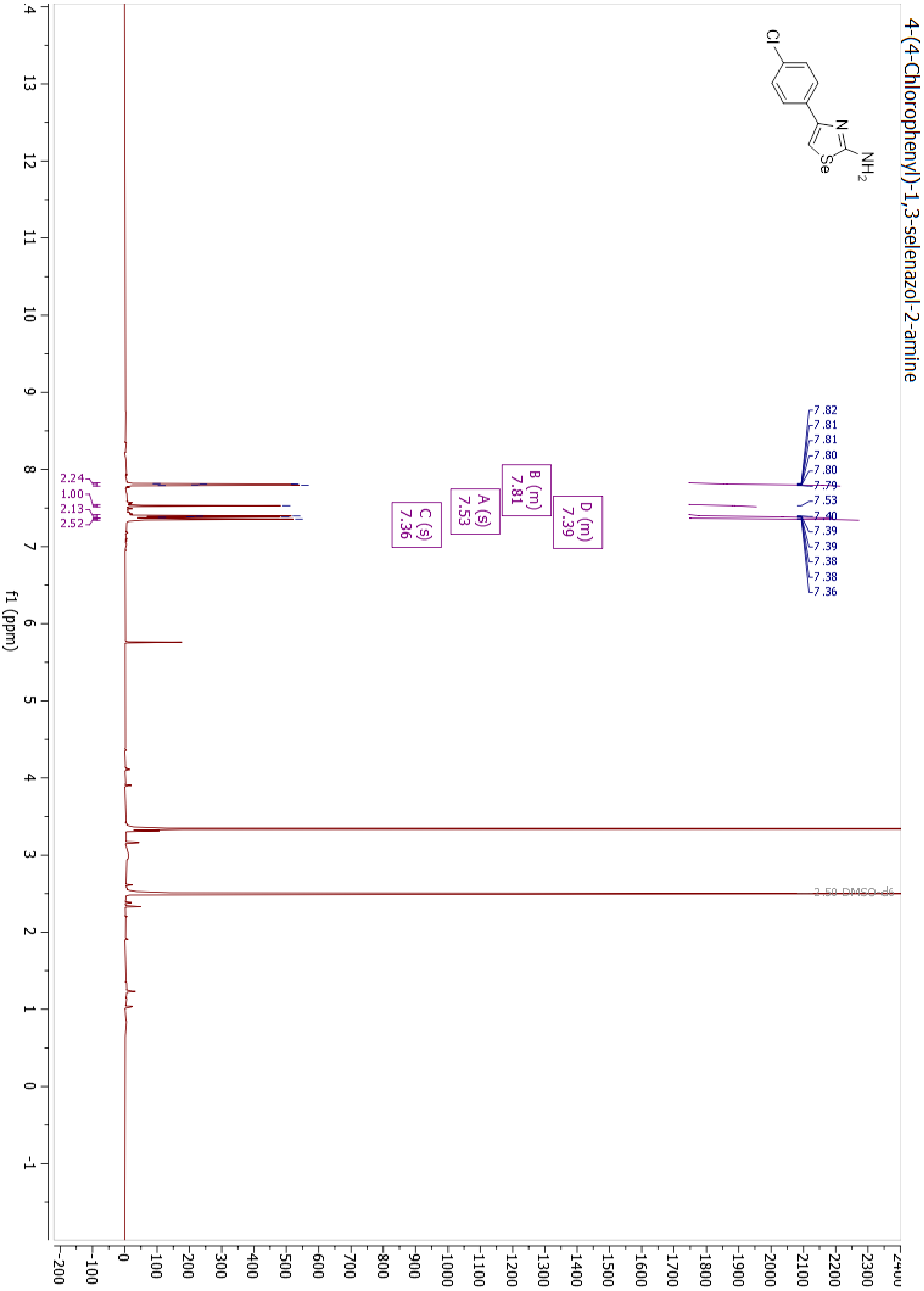

Figure S109. ^{1}H NMR spectrum of **7e** in d_6-DMSO (600 MHz)

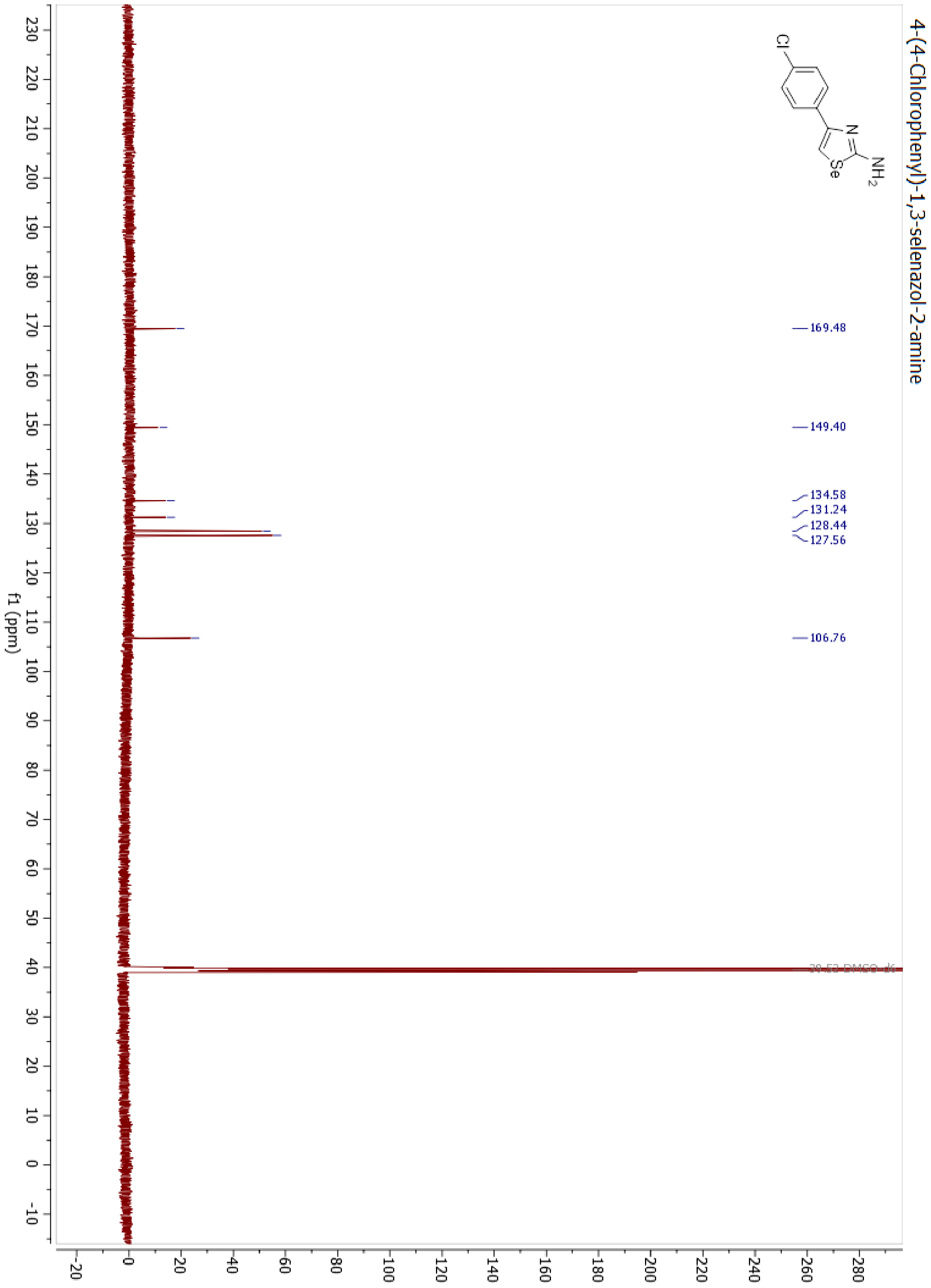

Figure S110. ^{13}C NMR spectrum of **7e** in d_6-DMSO (151 MHz)

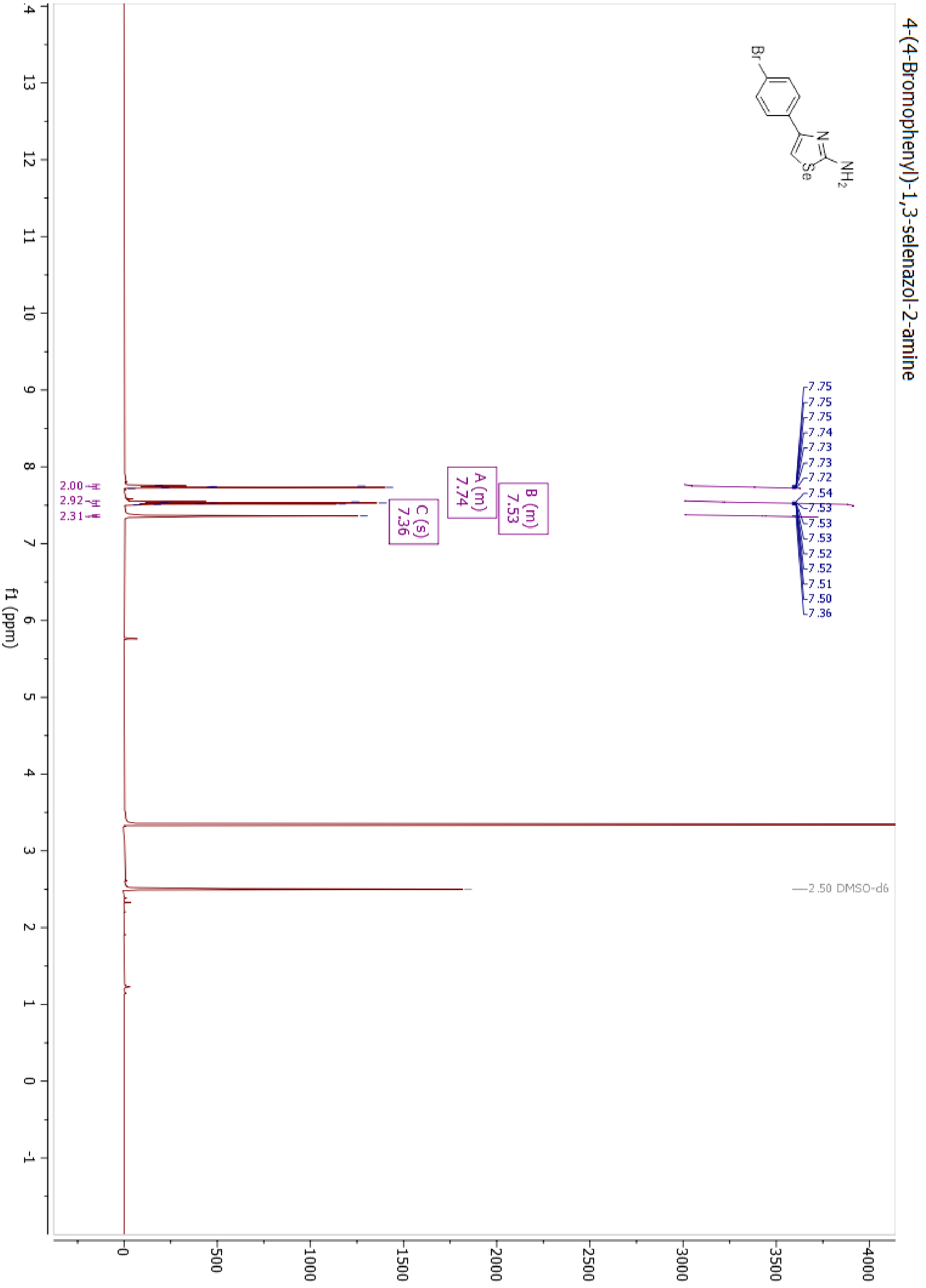

Figure S111. ^{1}H NMR spectrum of **7f** in d_6-DMSO (600 MHz)

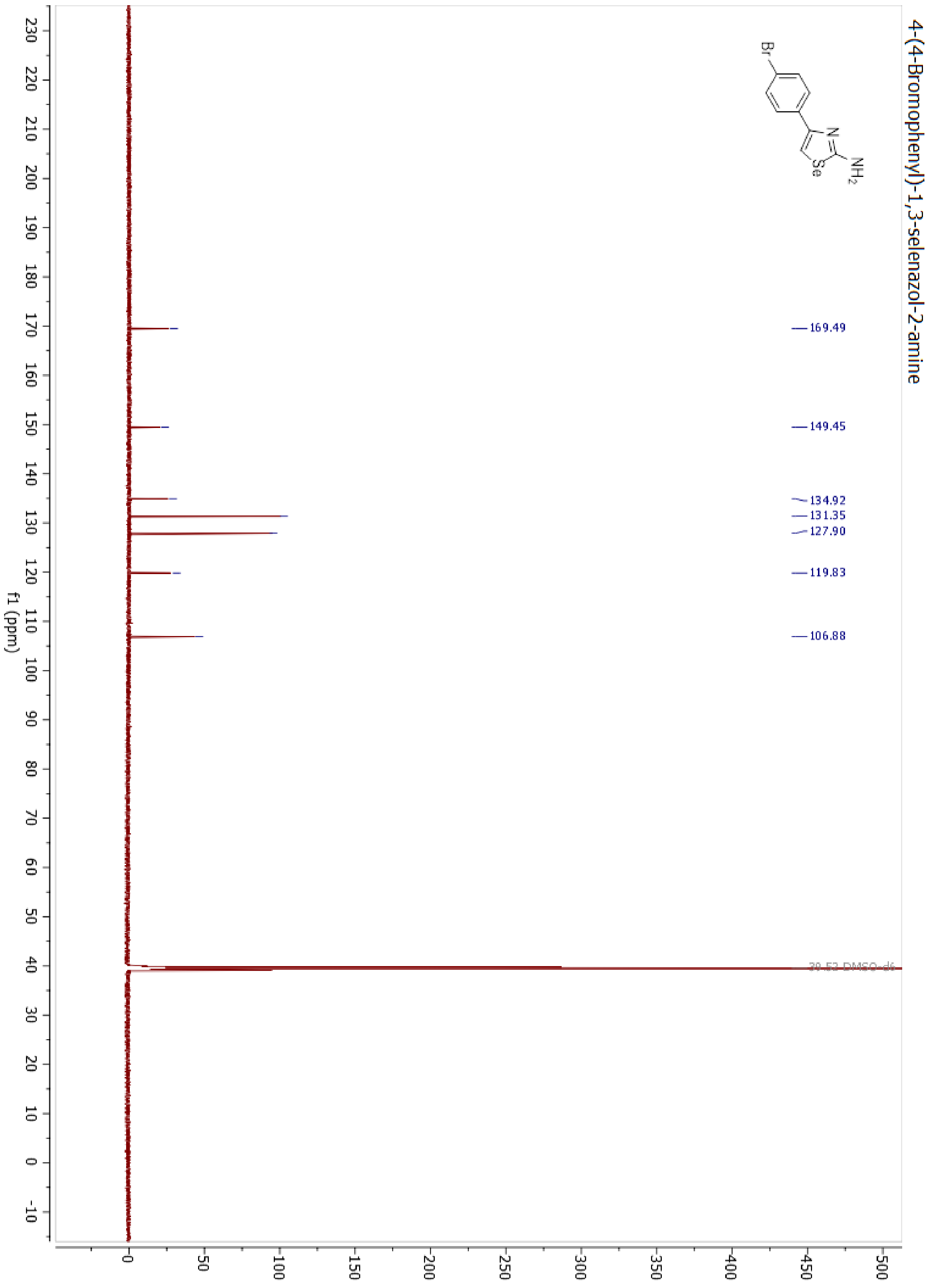

Figure S112. ^{13}C NMR spectrum of **7f** in d_6-DMSO (151 MHz)

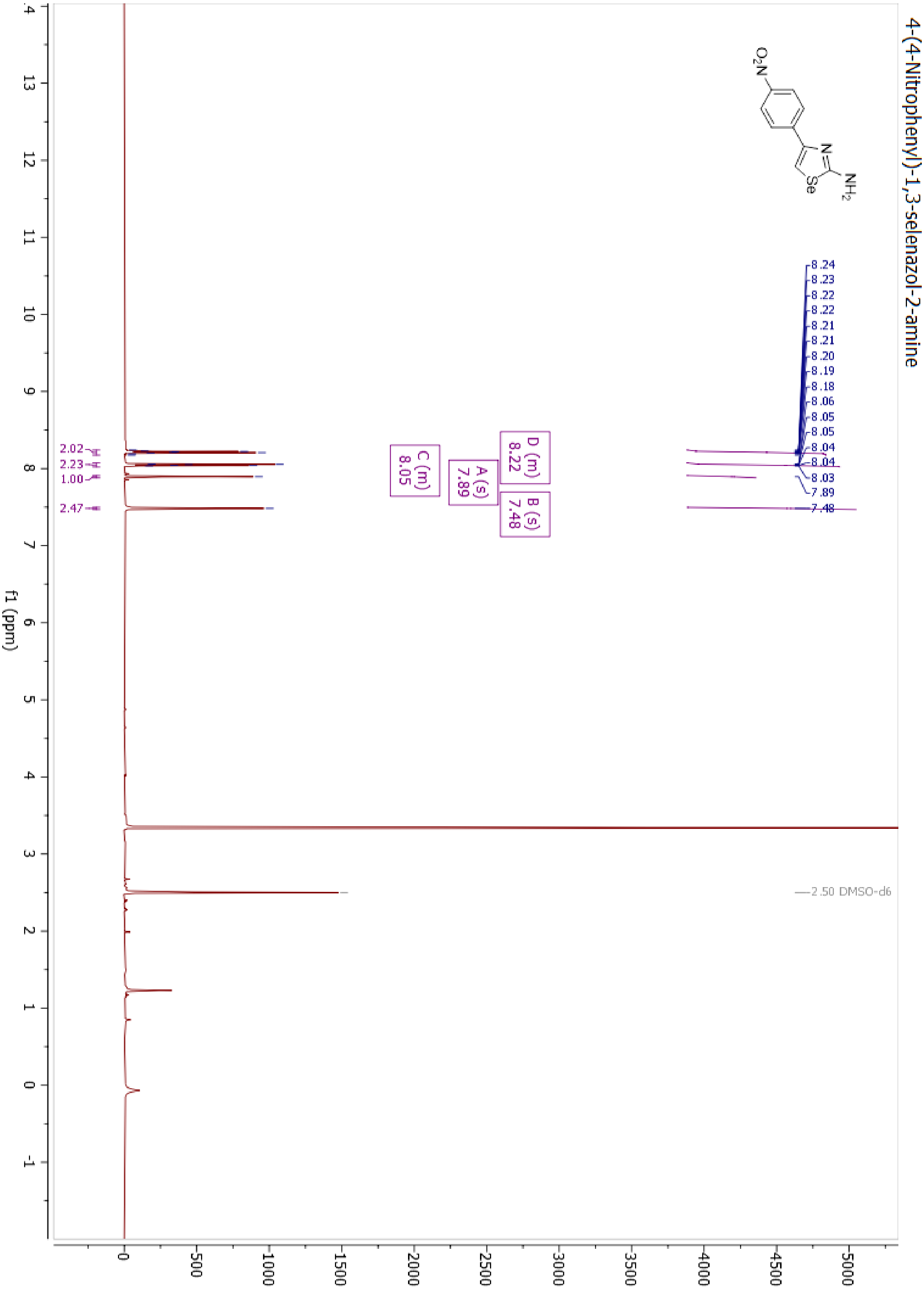

Figure S113. ^{1}H NMR spectrum of **7g** in d_6-DMSO (600 MHz)

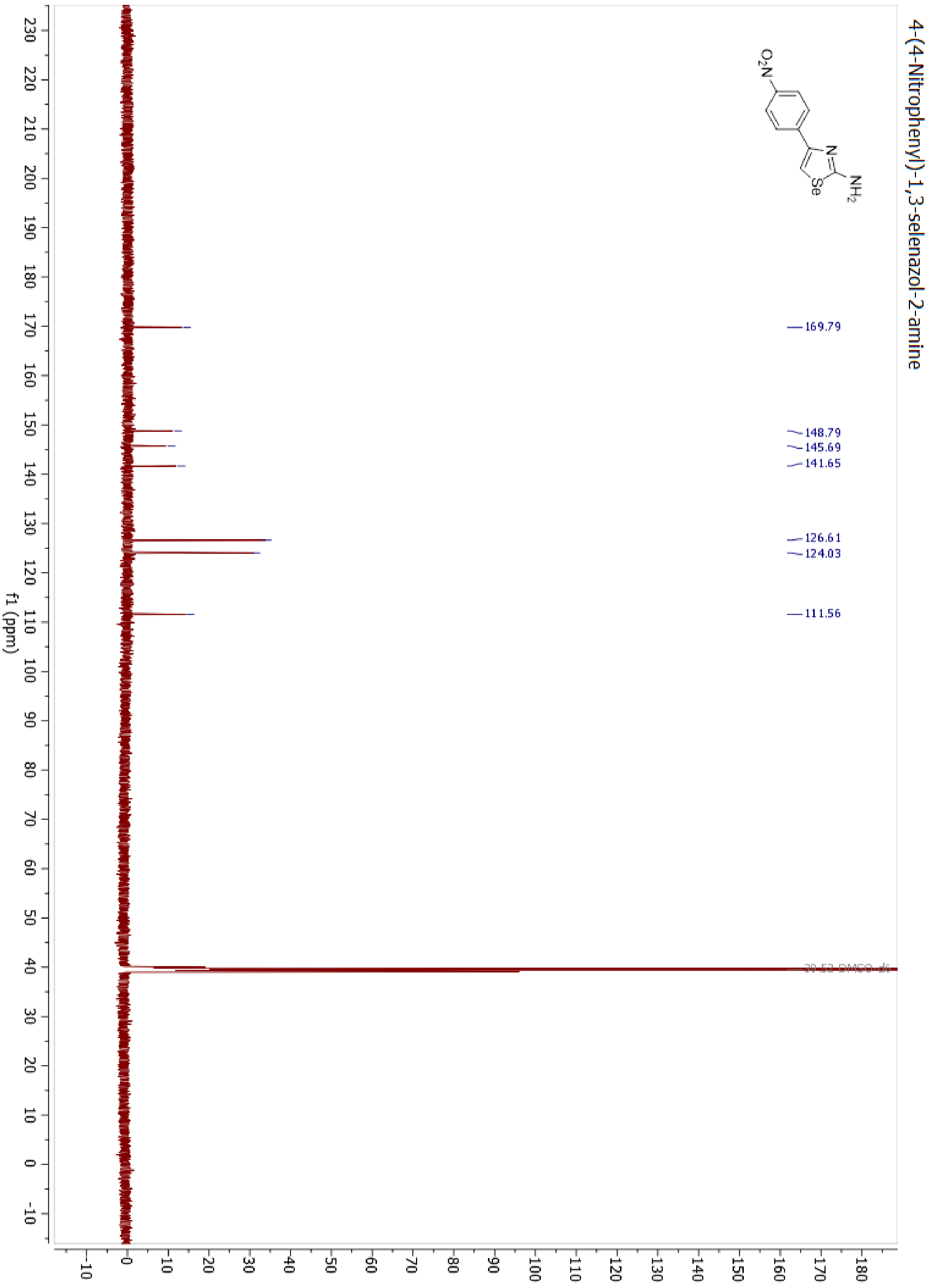

Figure S114. ^{13}C NMR spectrum of **7g** in d_6-DMSO (151 MHz)

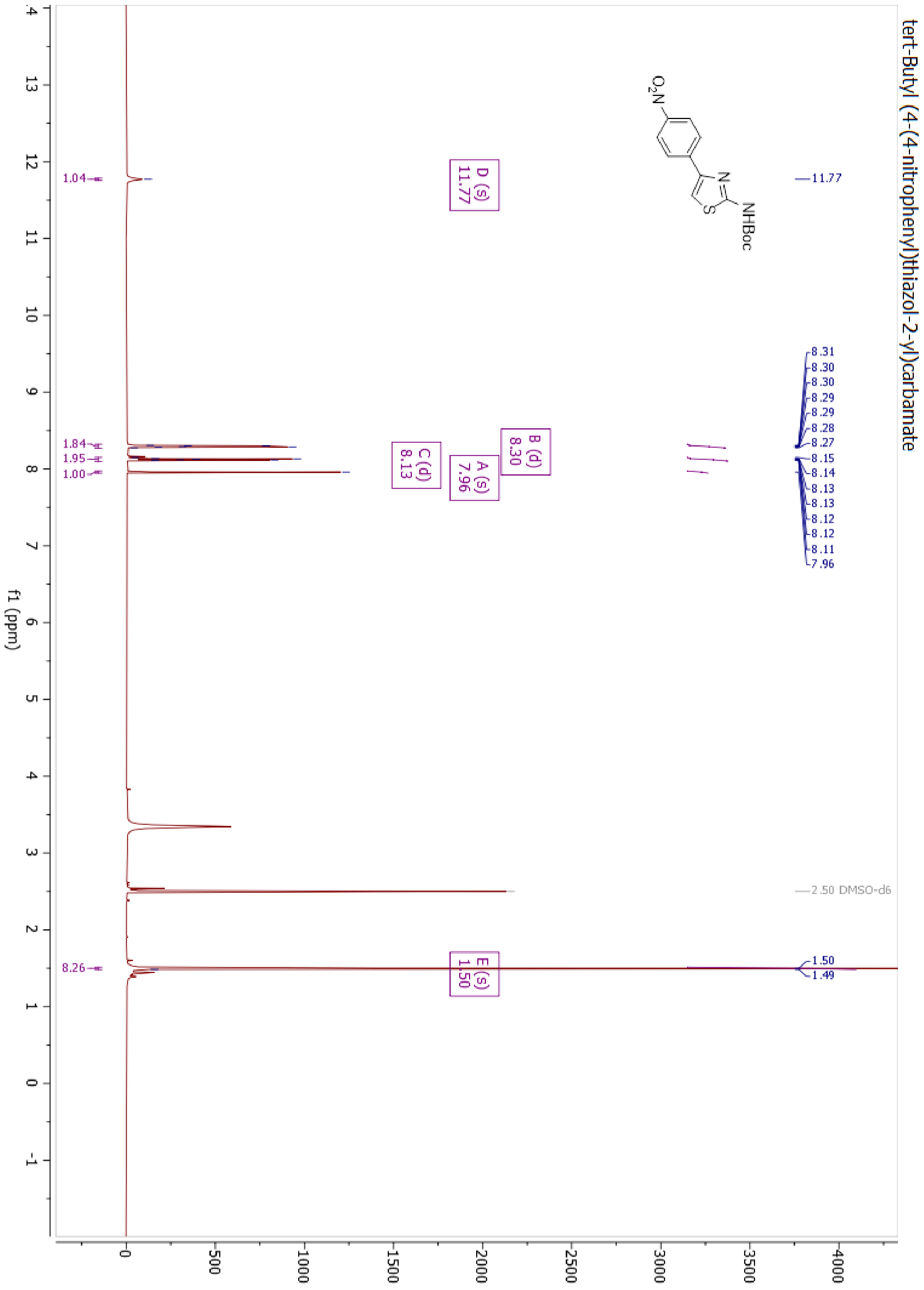

Figure S115. ^{1}H NMR spectrum of **3j’** in d_6-DMSO (600 MHz)

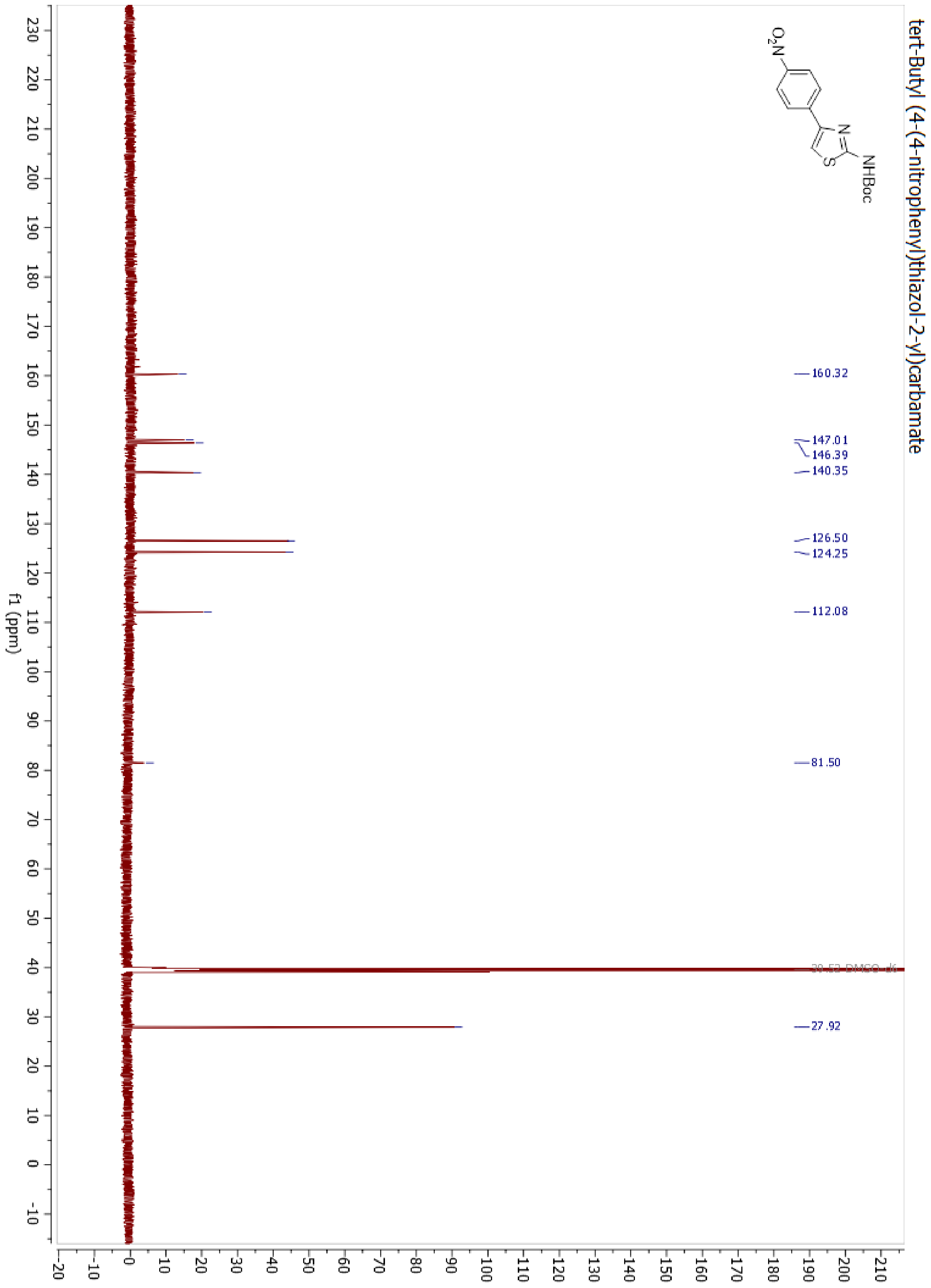

Figure S116. ^{13}C NMR spectrum of **3j’** in d_6-DMSO (151 MHz)

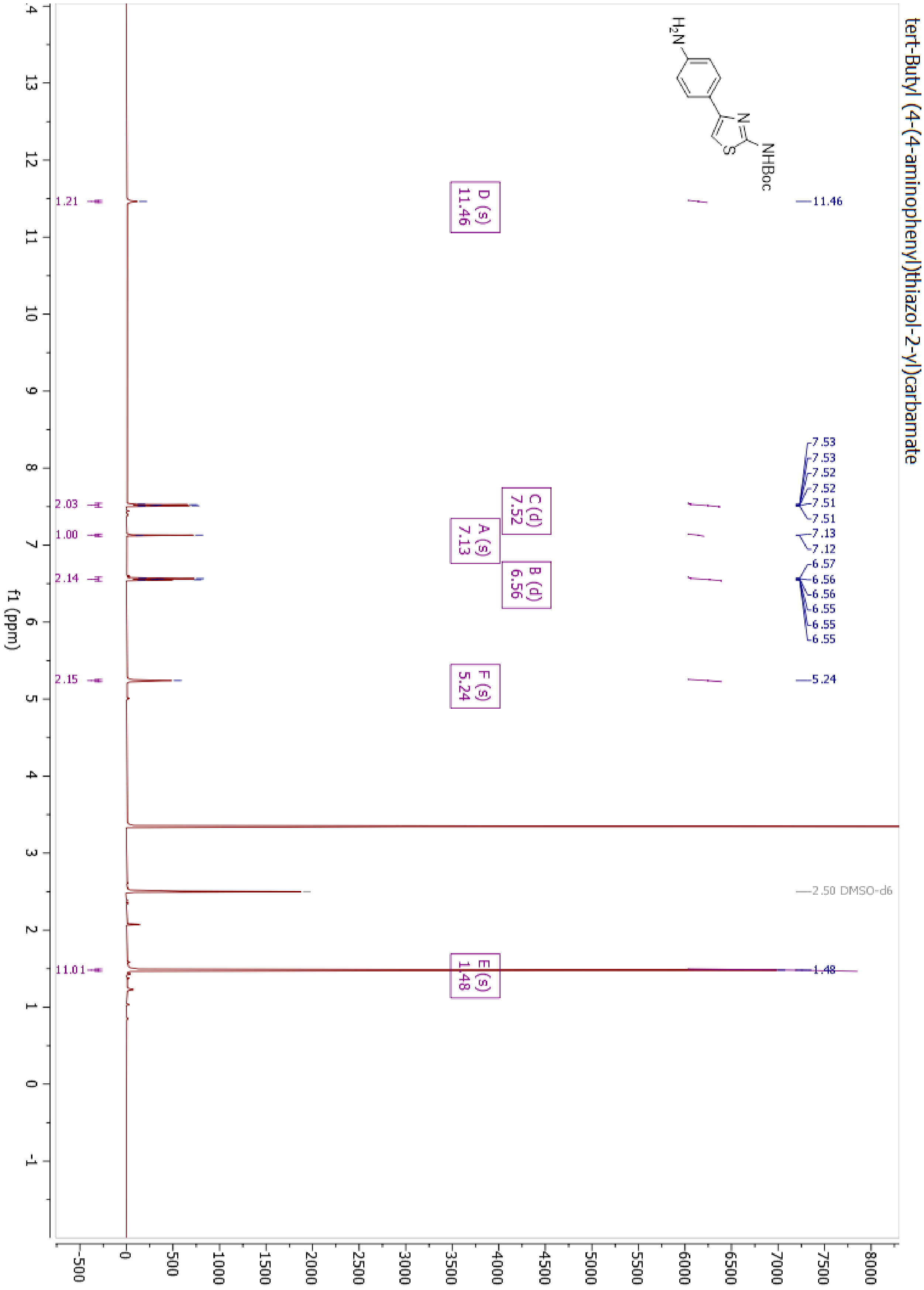

Figure S117. ^{1}H NMR spectrum of **3k’** in d_6-DMSO (600 MHz)

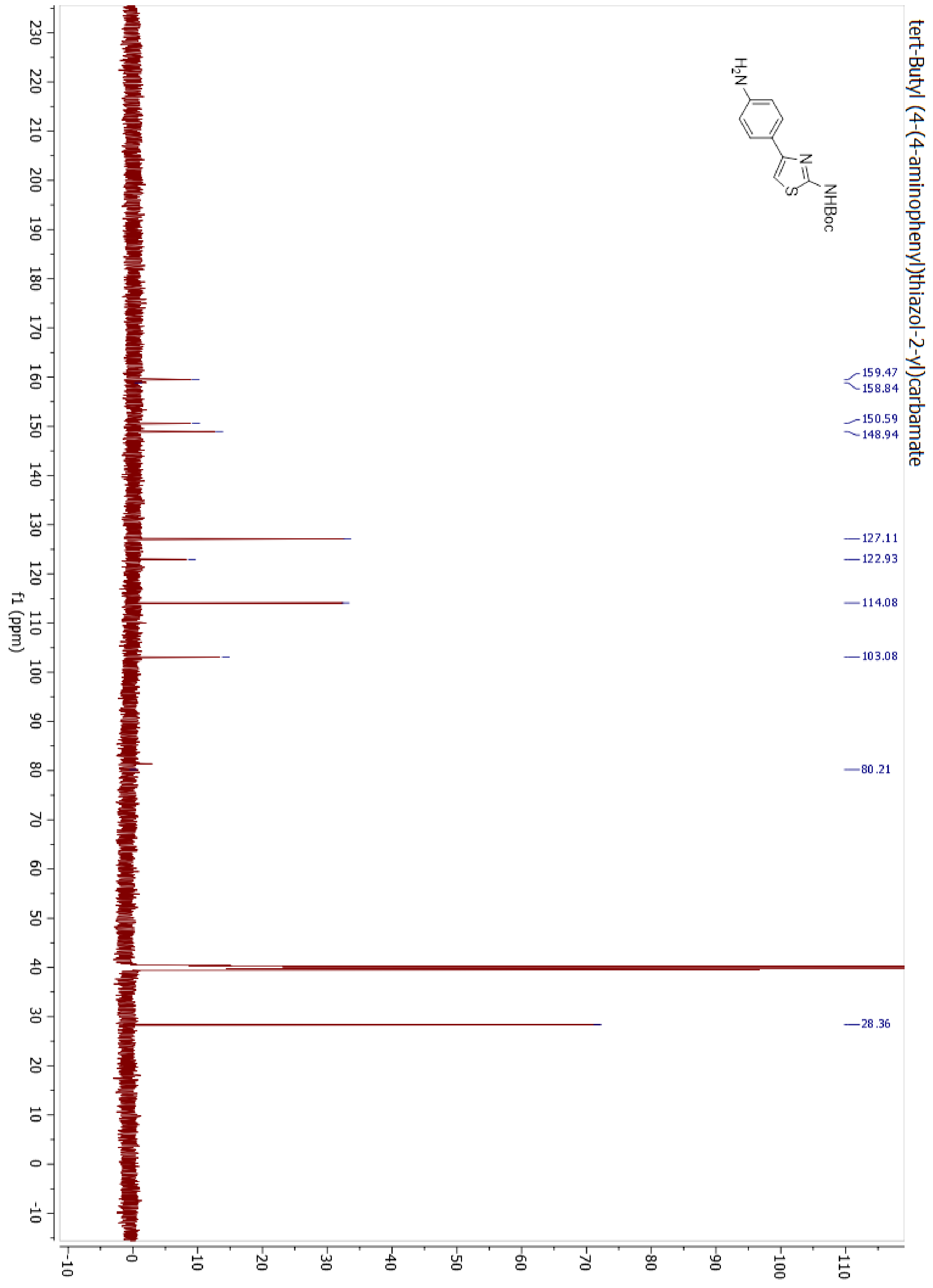

Figure S118. ^{13}C NMR spectrum of **3k’** in d_6-DMSO (151 MHz)

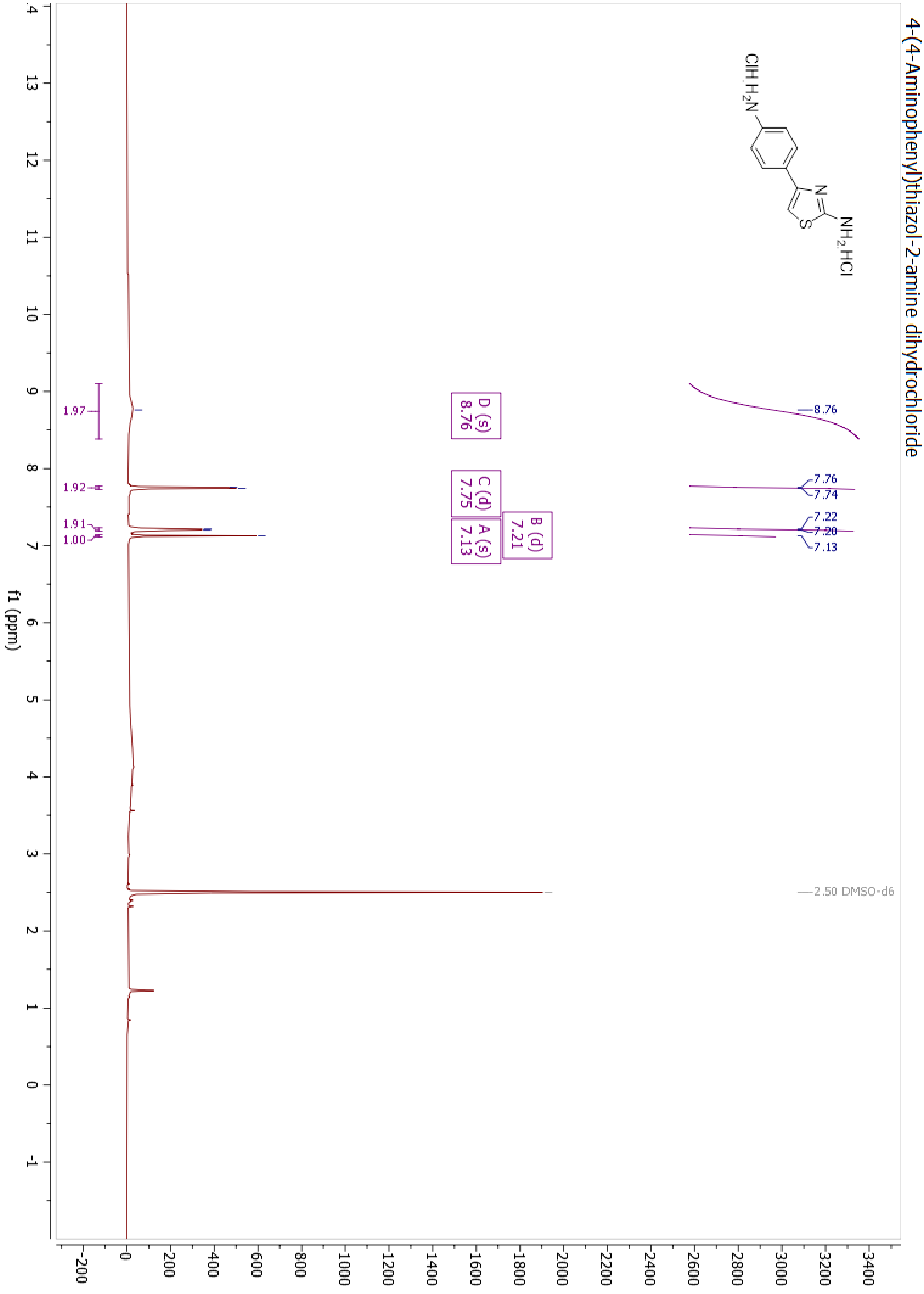

Figure S119. ^{1}H NMR spectrum of **3l’** in d_6-DMSO (600 MHz)

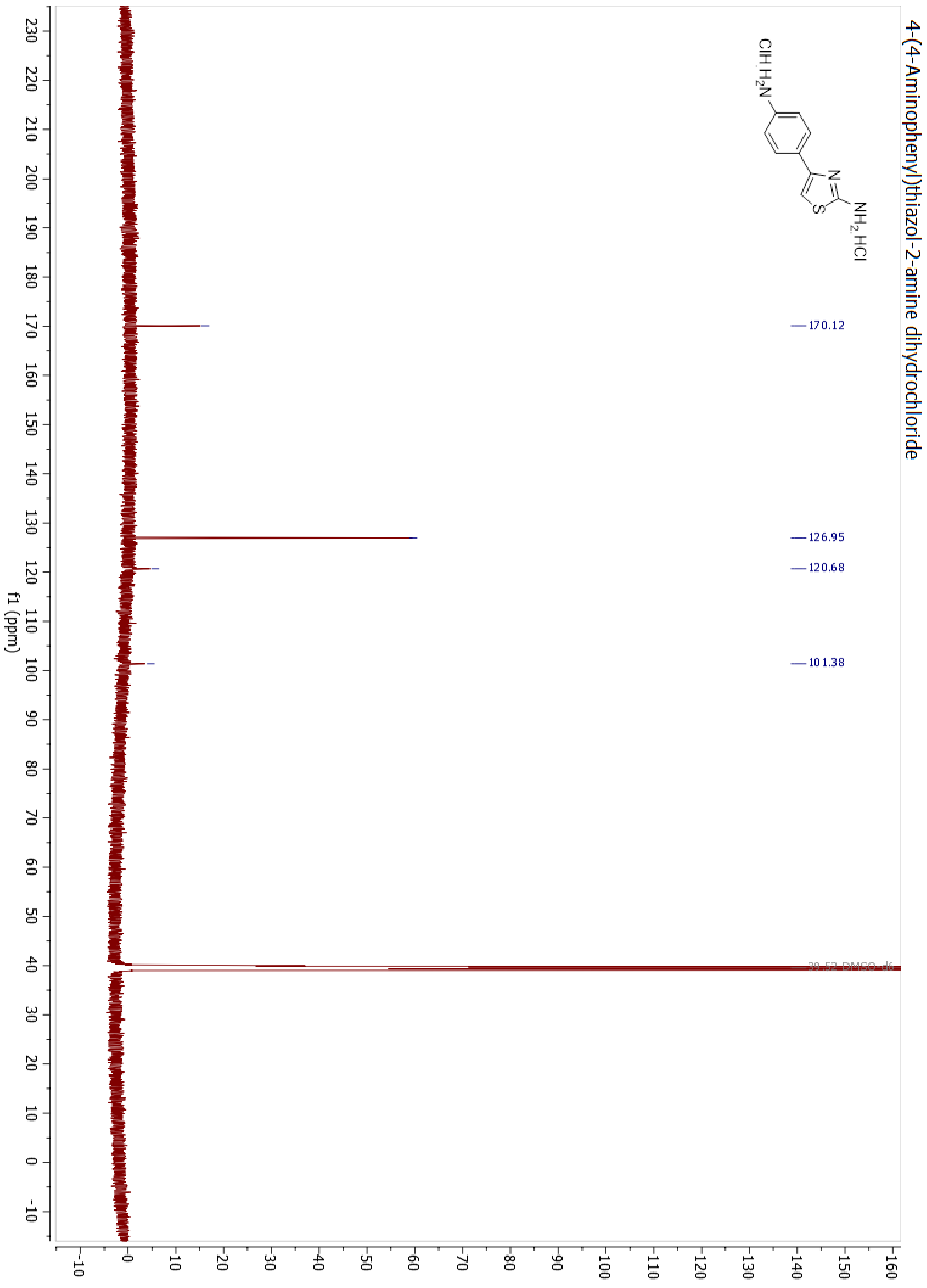

Figure S120. ^{13}C NMR spectrum of **3l’** in d_6-DMSO (151 MHz)

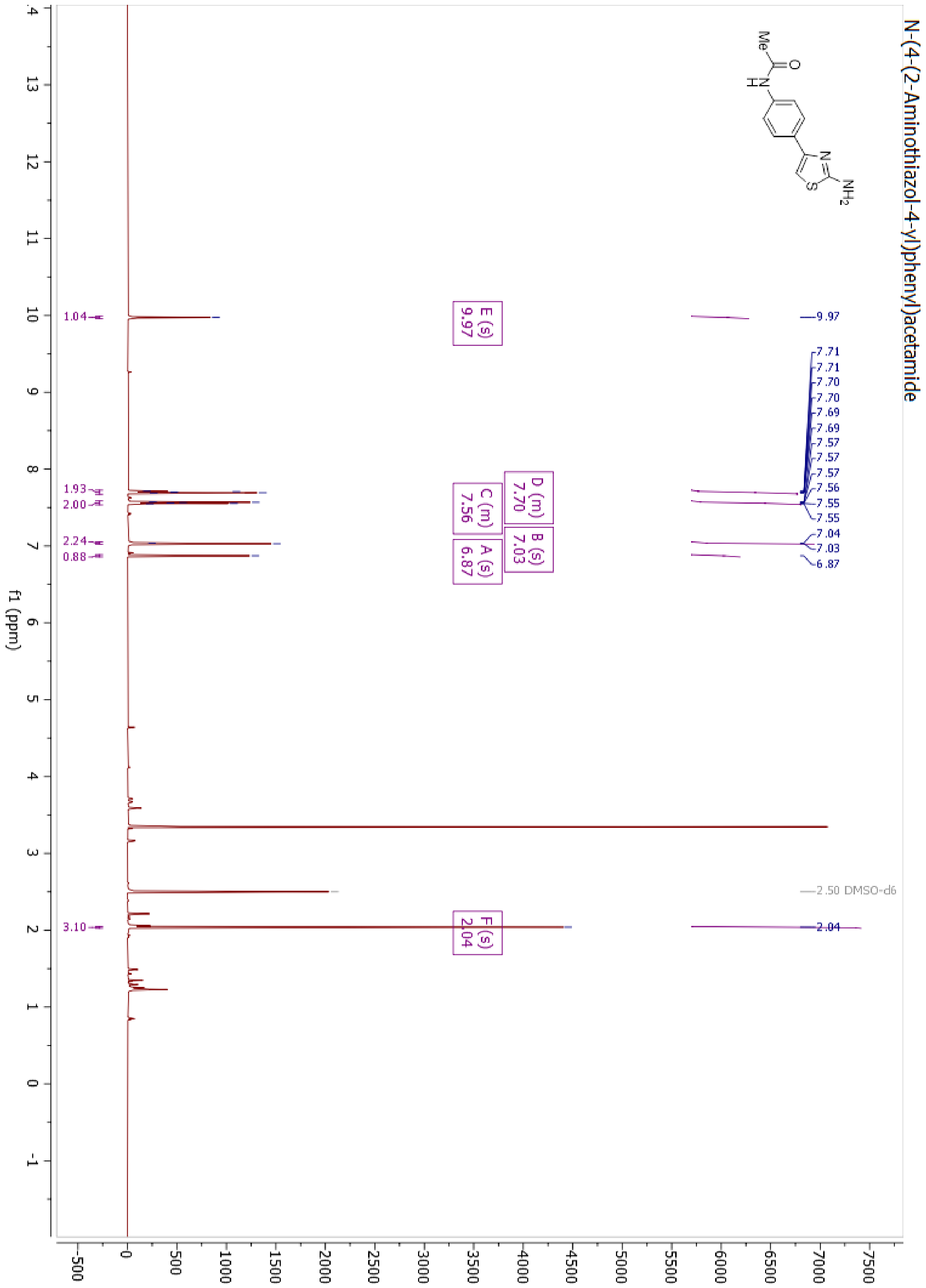

Figure S121. ^{1}H NMR spectrum of **3s'** in d_6-DMSO (600 MHz)

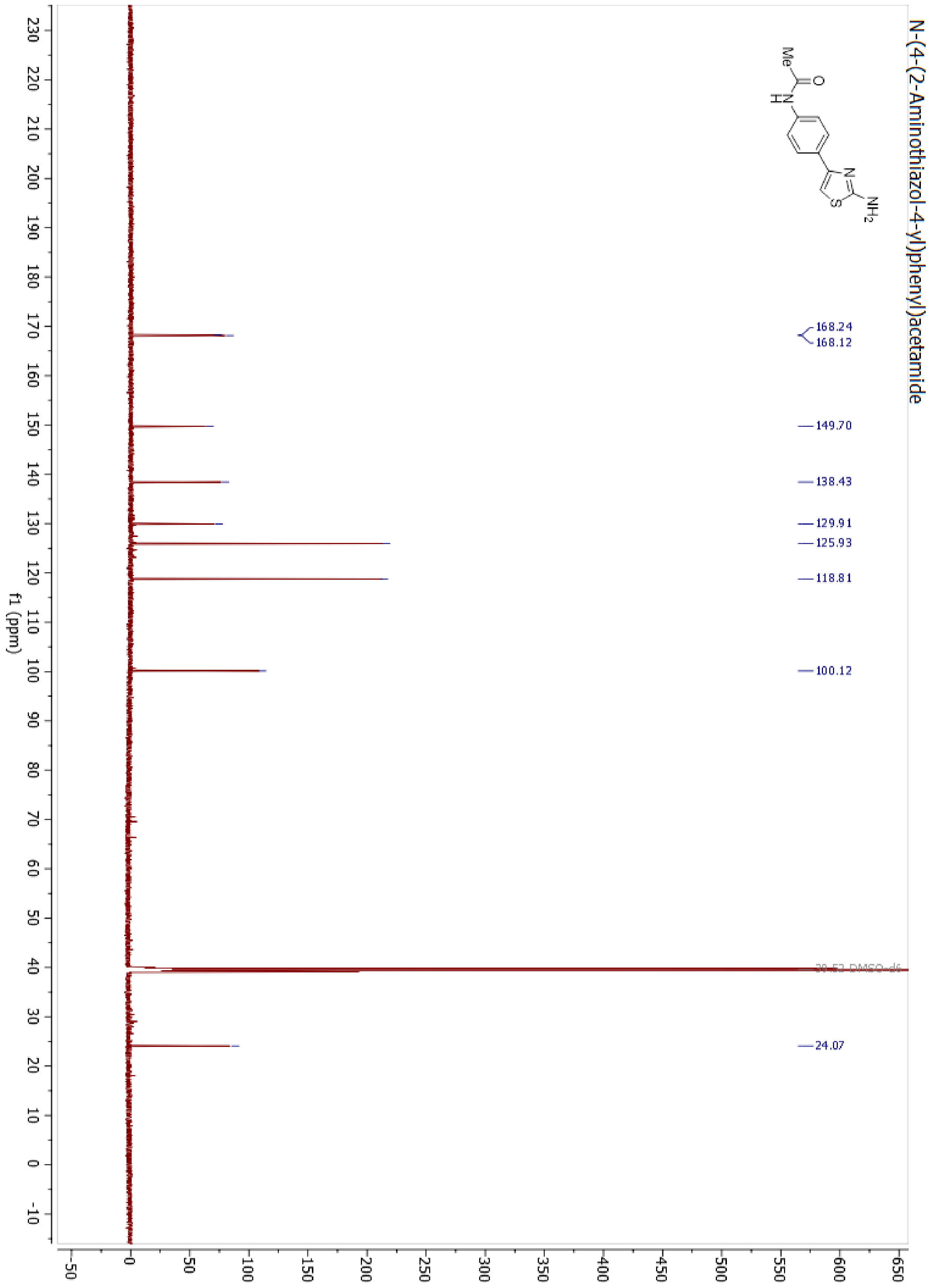

Figure S122. ^{13}C NMR spectrum of **3s’** in d_6-DMSO (151 MHz)

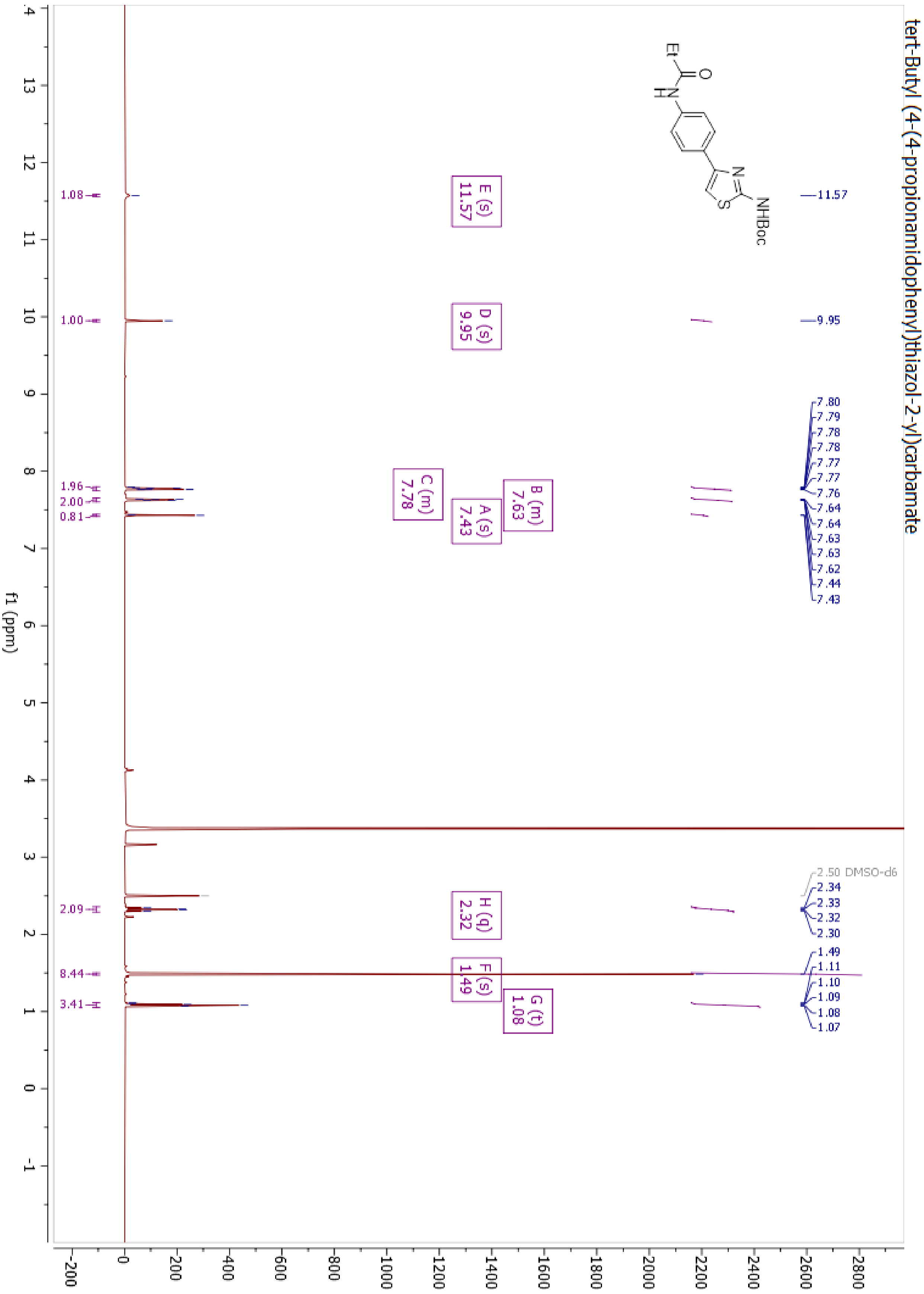

Figure S123. ^{1}H NMR spectrum of **3n’** in d_6-DMSO (600 MHz)

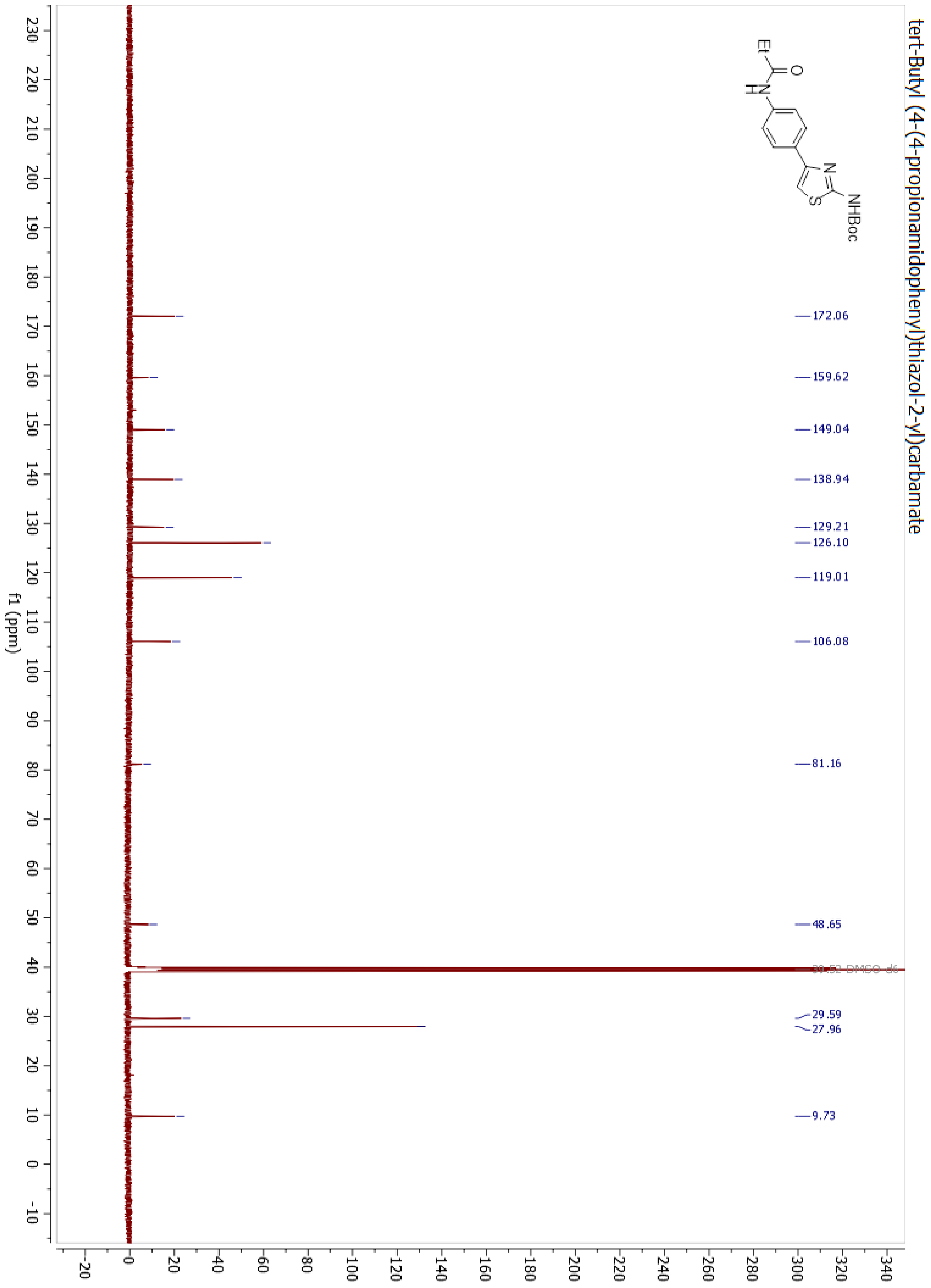

Figure S124. ^{13}C NMR spectrum of **3n’** in d_6-DMSO (151 MHz)

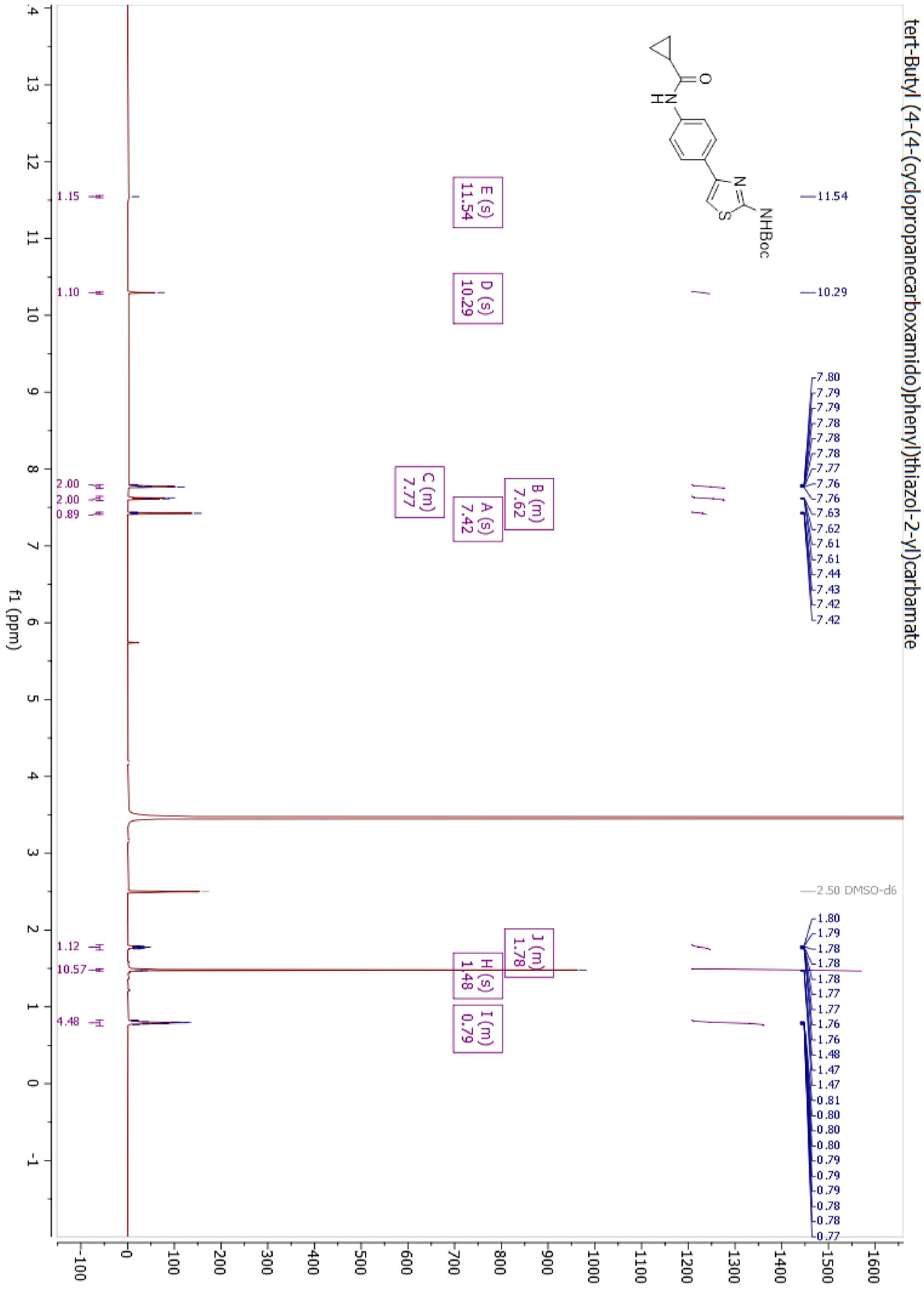

Figure S125. ^{1}H NMR spectrum of **3o’** in d_6-DMSO (600 MHz)

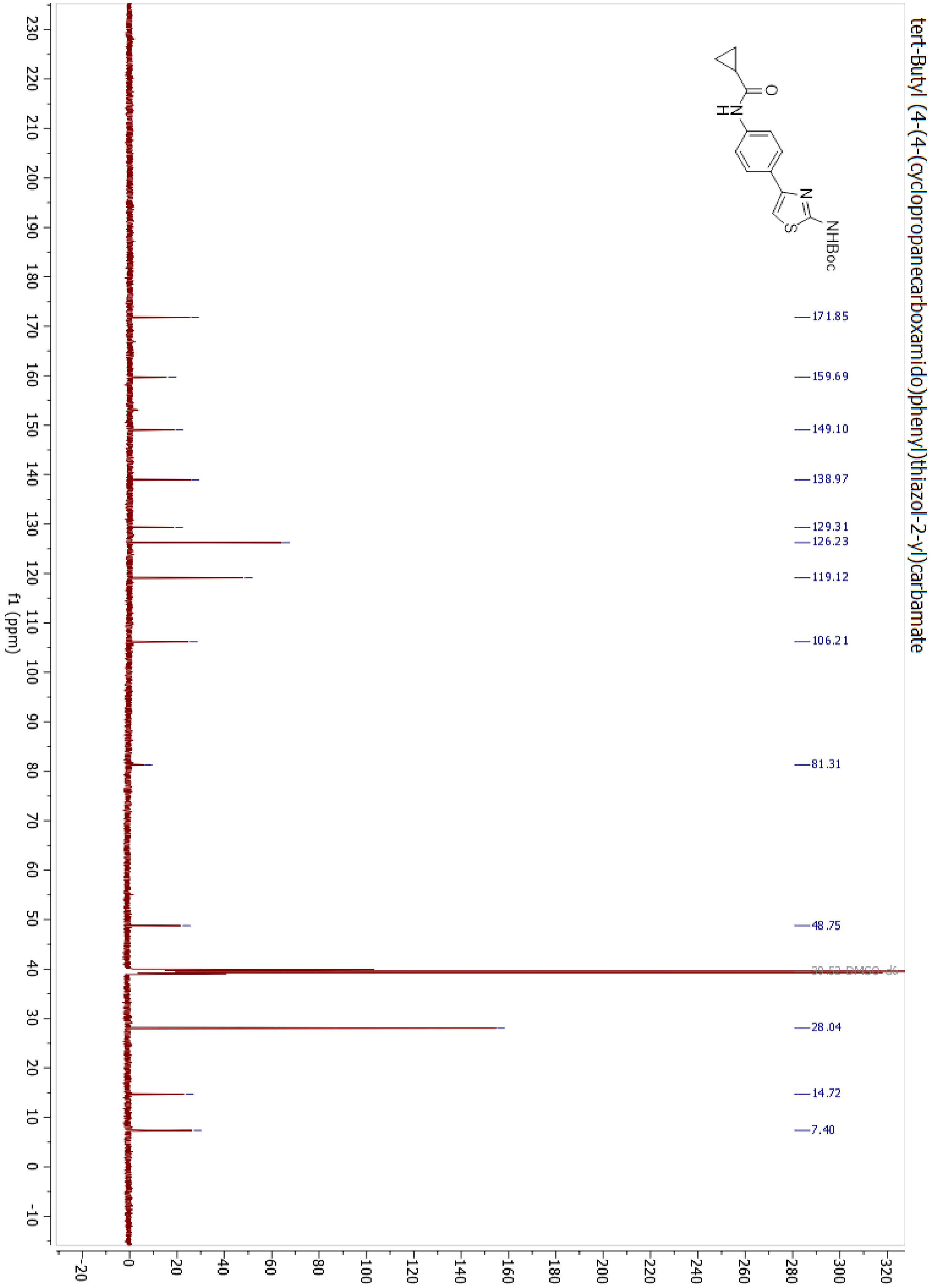

Figure S126. ^{13}C NMR spectrum of **3o'** in d_6-DMSO (151 MHz)

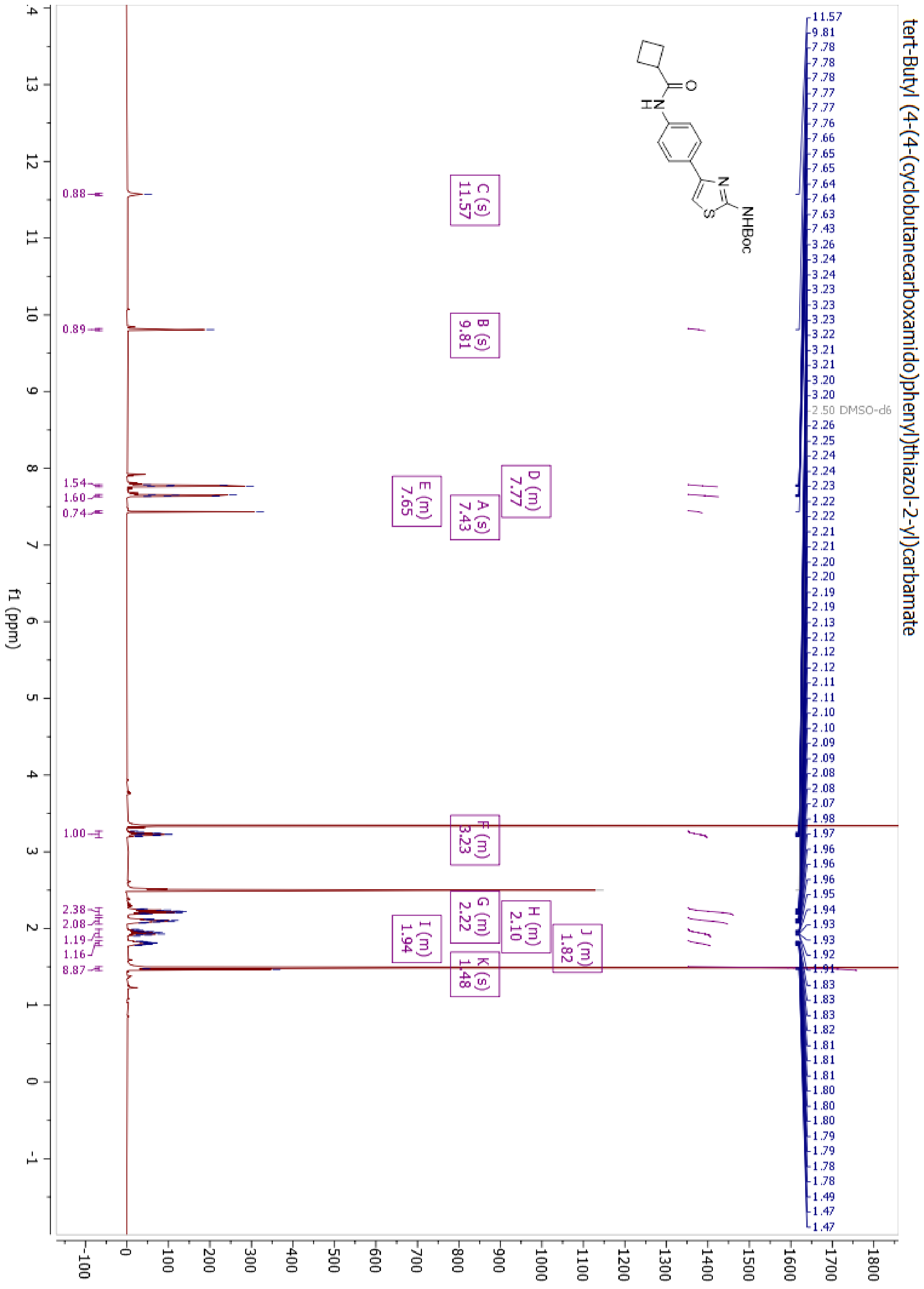

Figure S127. ^{1}H NMR spectrum of **3p’** in d_6-DMSO (600 MHz)

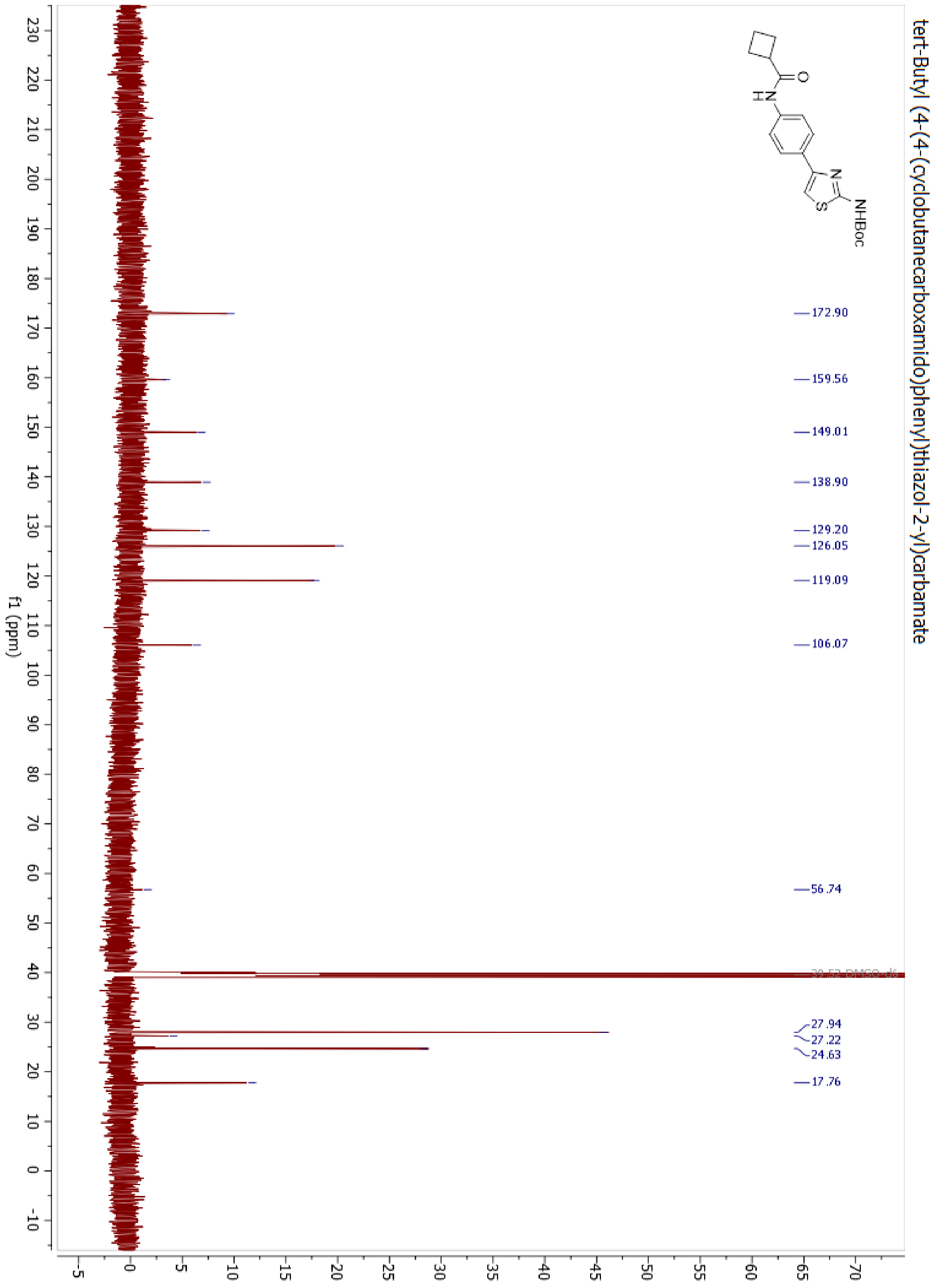

Figure S128. ^{13}C NMR spectrum of **3p’** in d_6-DMSO (151 MHz)

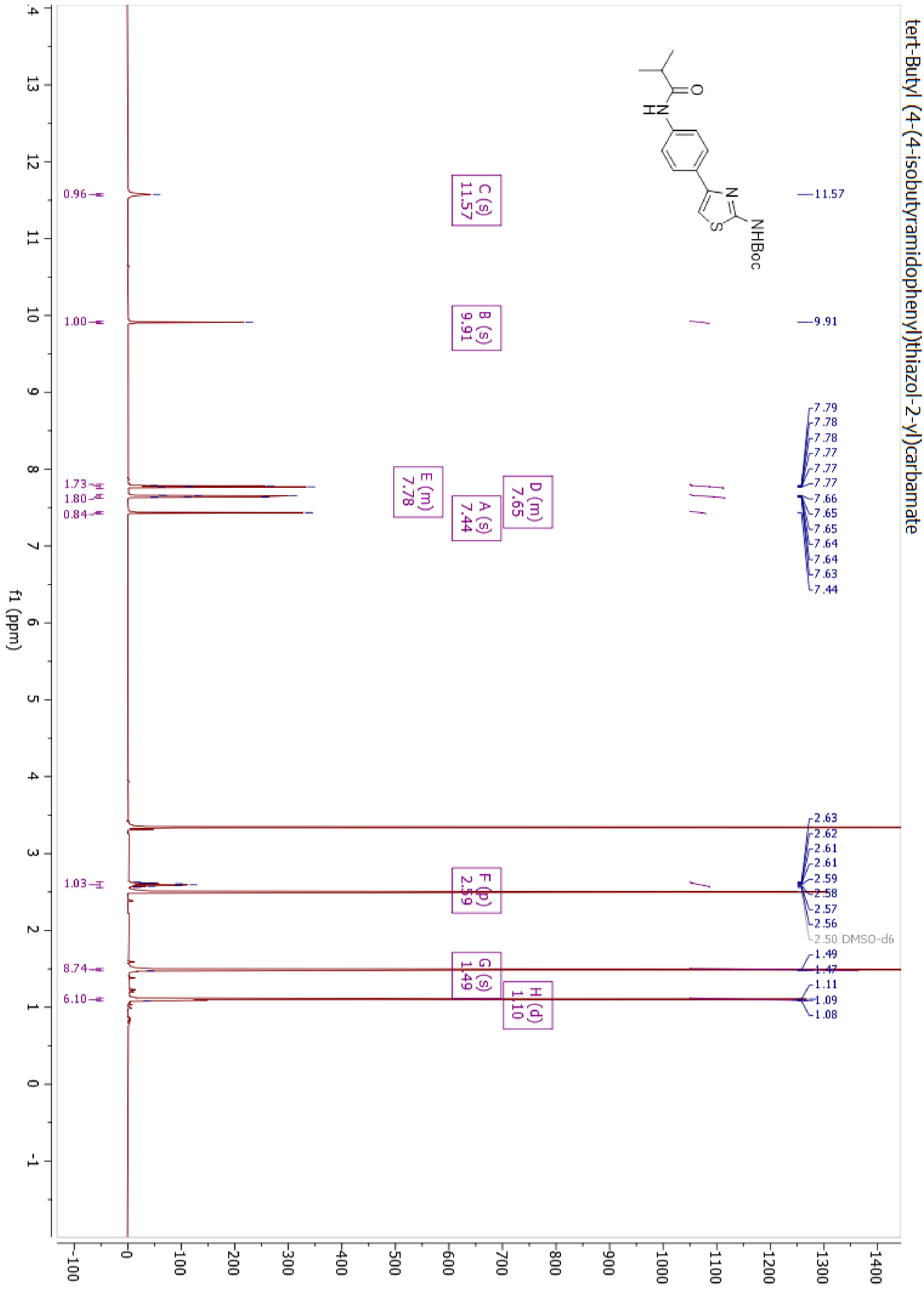

Figure S129. ^{1}H NMR spectrum of **3q'** in d_6-DMSO (600 MHz)

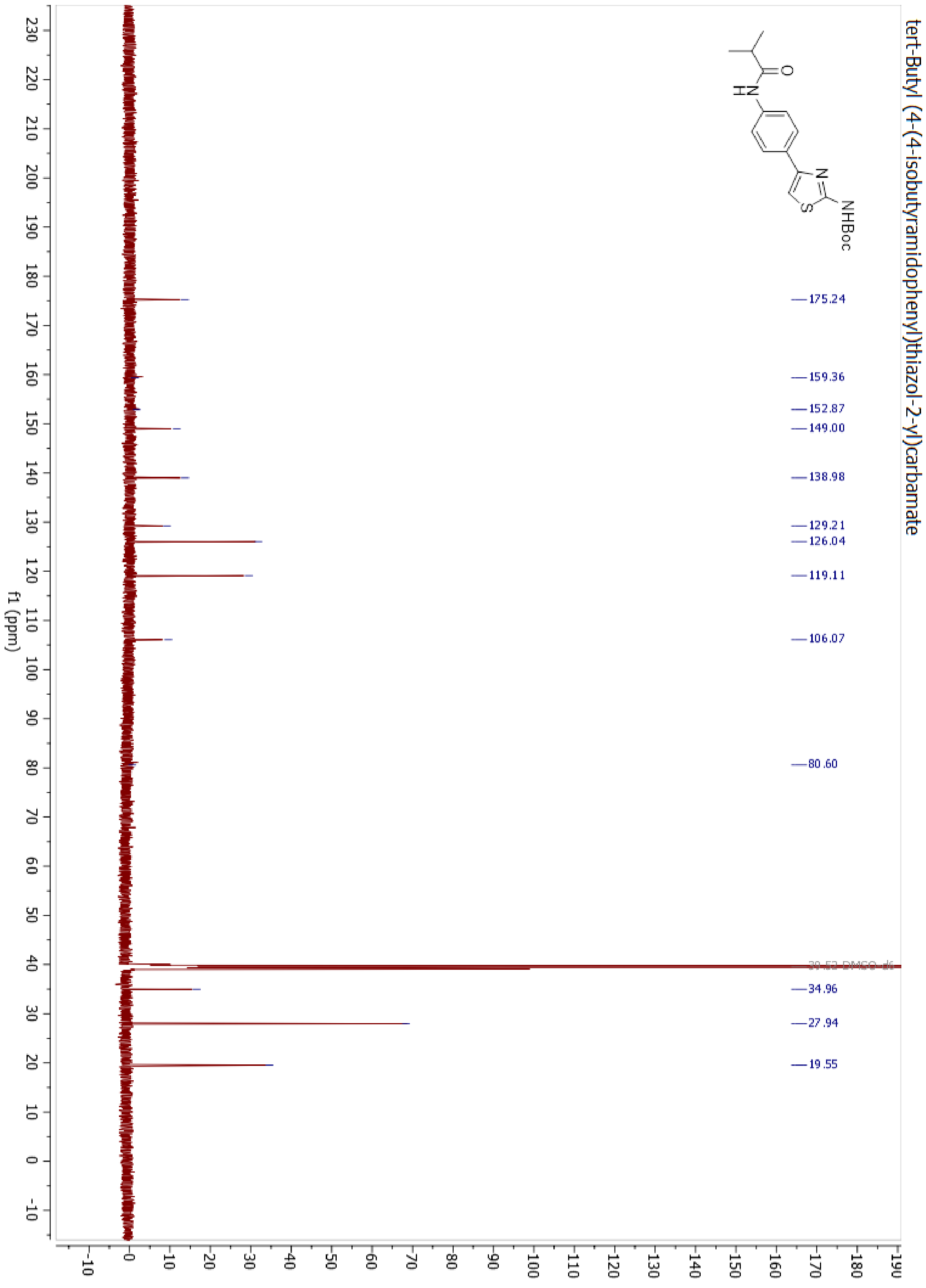

Figure S130. ^{13}C NMR spectrum of **3q'** in d_6-DMSO (151 MHz)

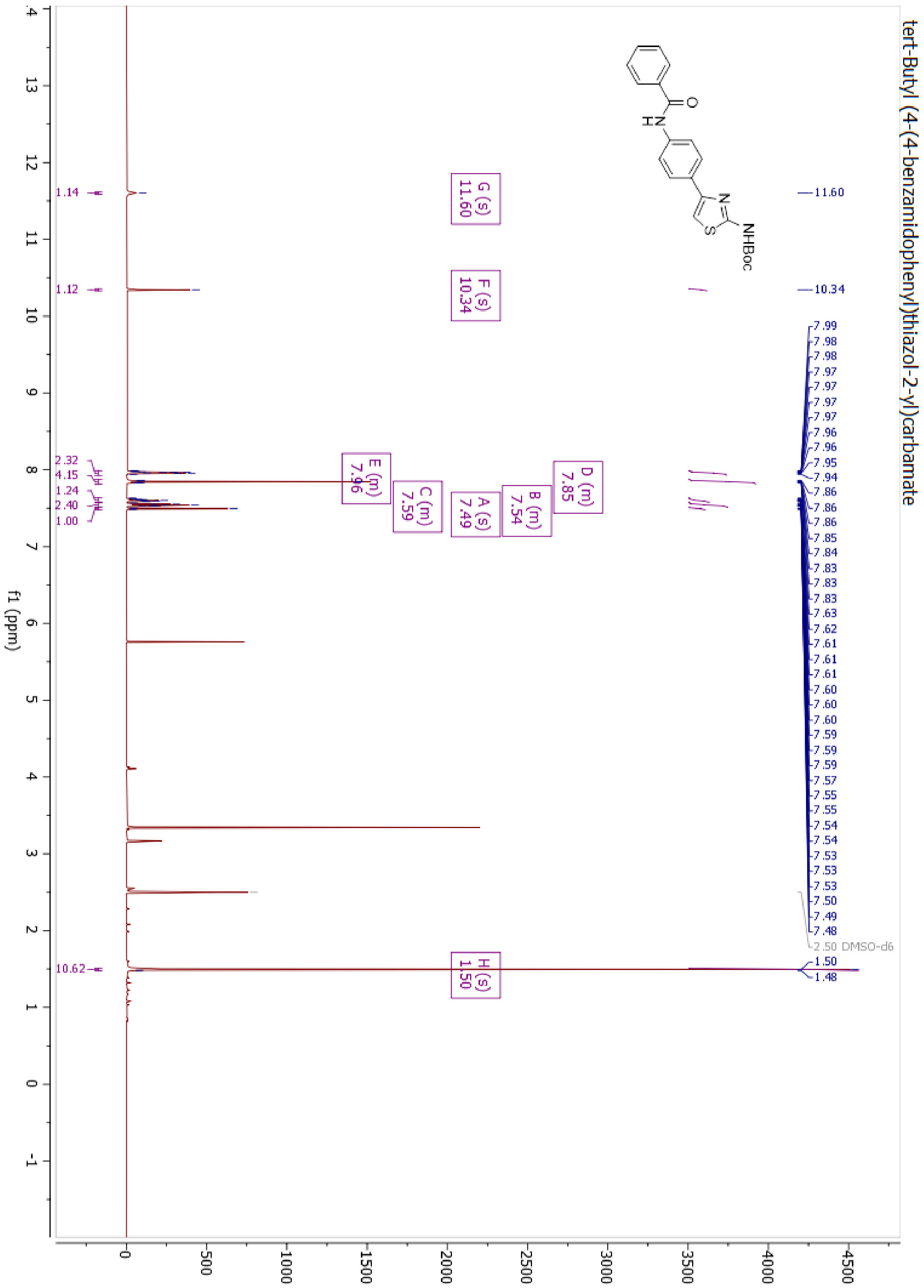

Figure S131. ^{1}H NMR spectrum of **3r’** in d_6-DMSO (600 MHz)

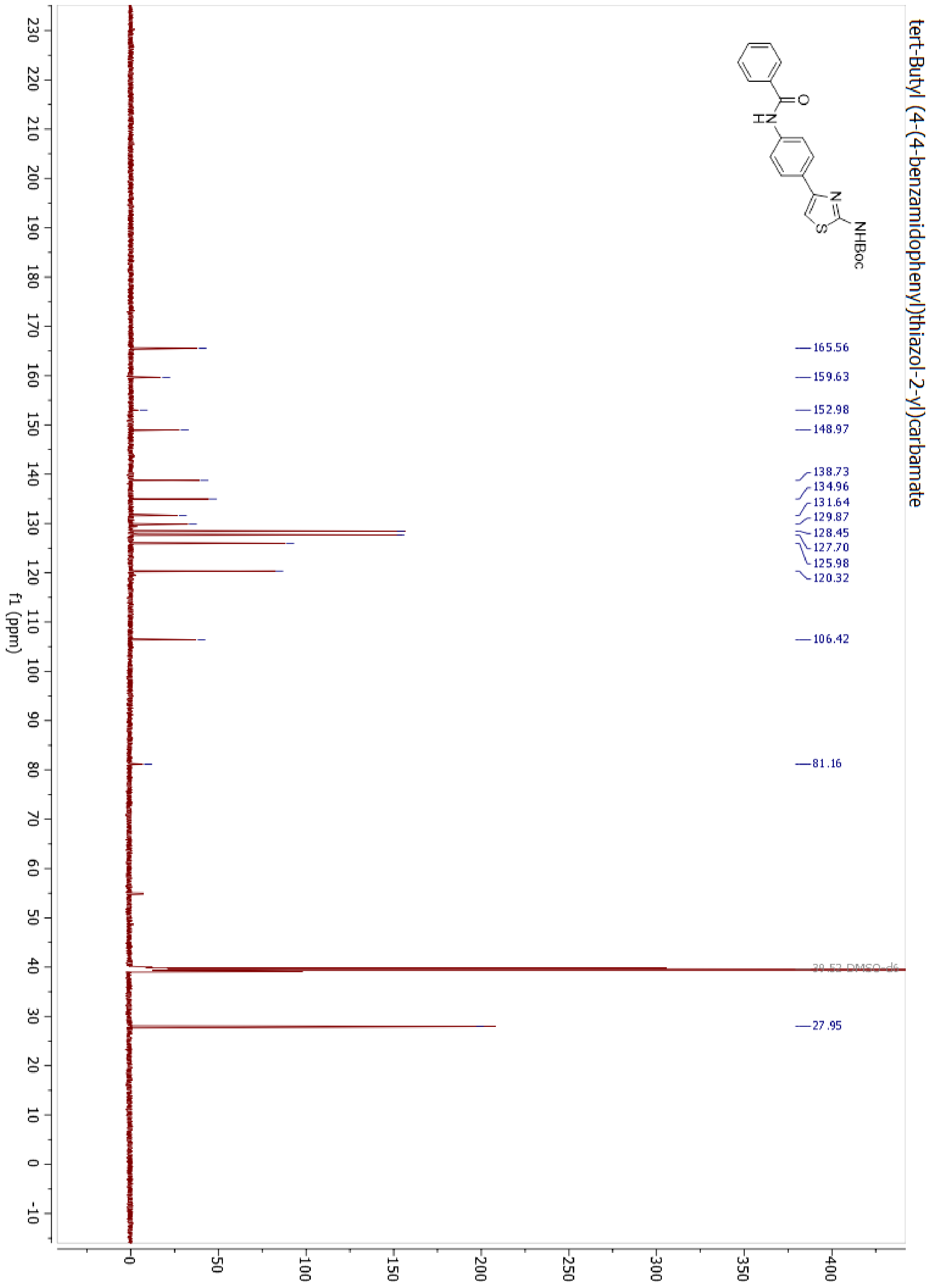

Figure S132. ^{13}C NMR spectrum of **3r’** in d_6-DMSO (151 MHz)

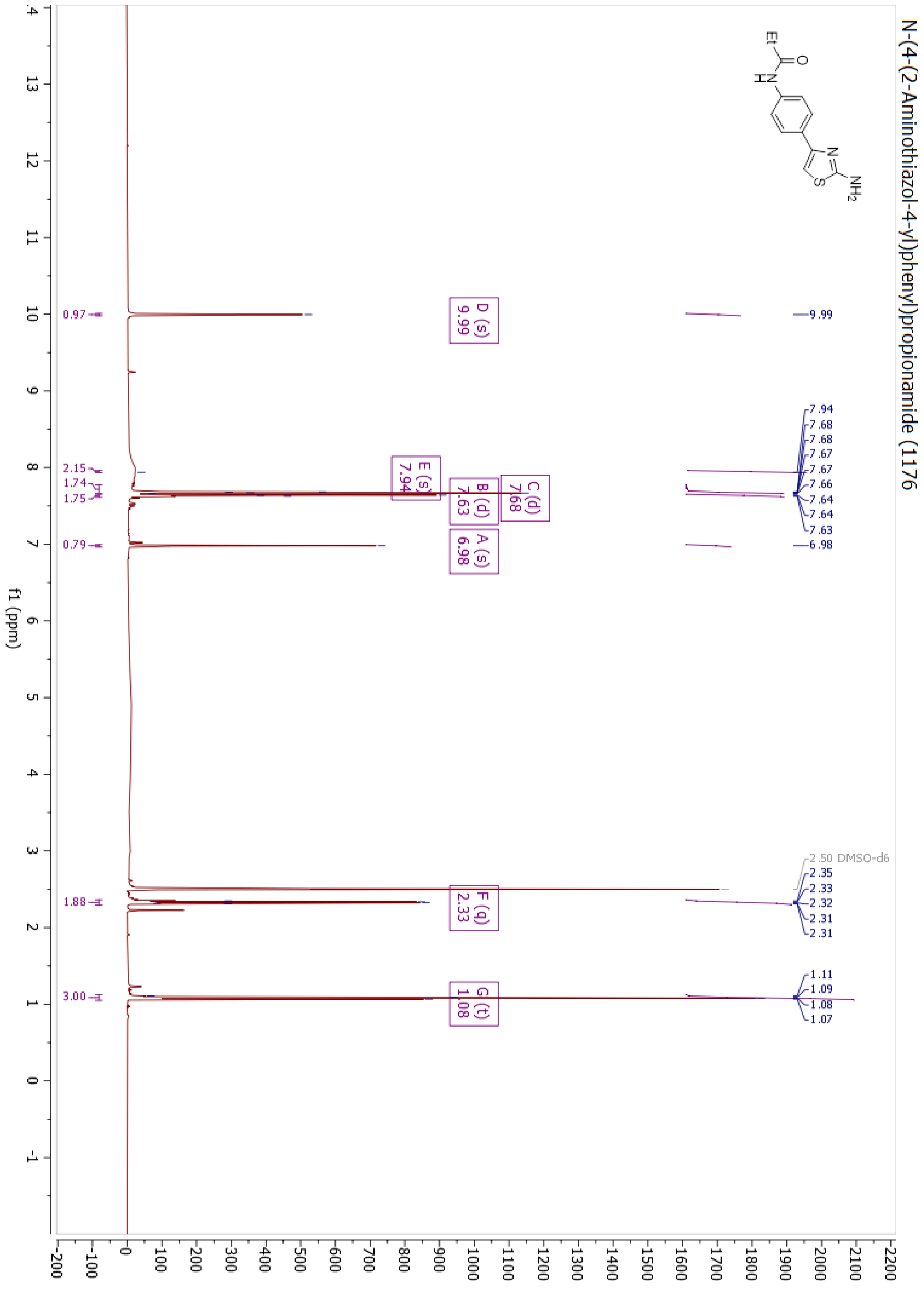

Figure S133. ^{1}H NMR spectrum of **3t'** in d_6-DMSO (600 MHz)

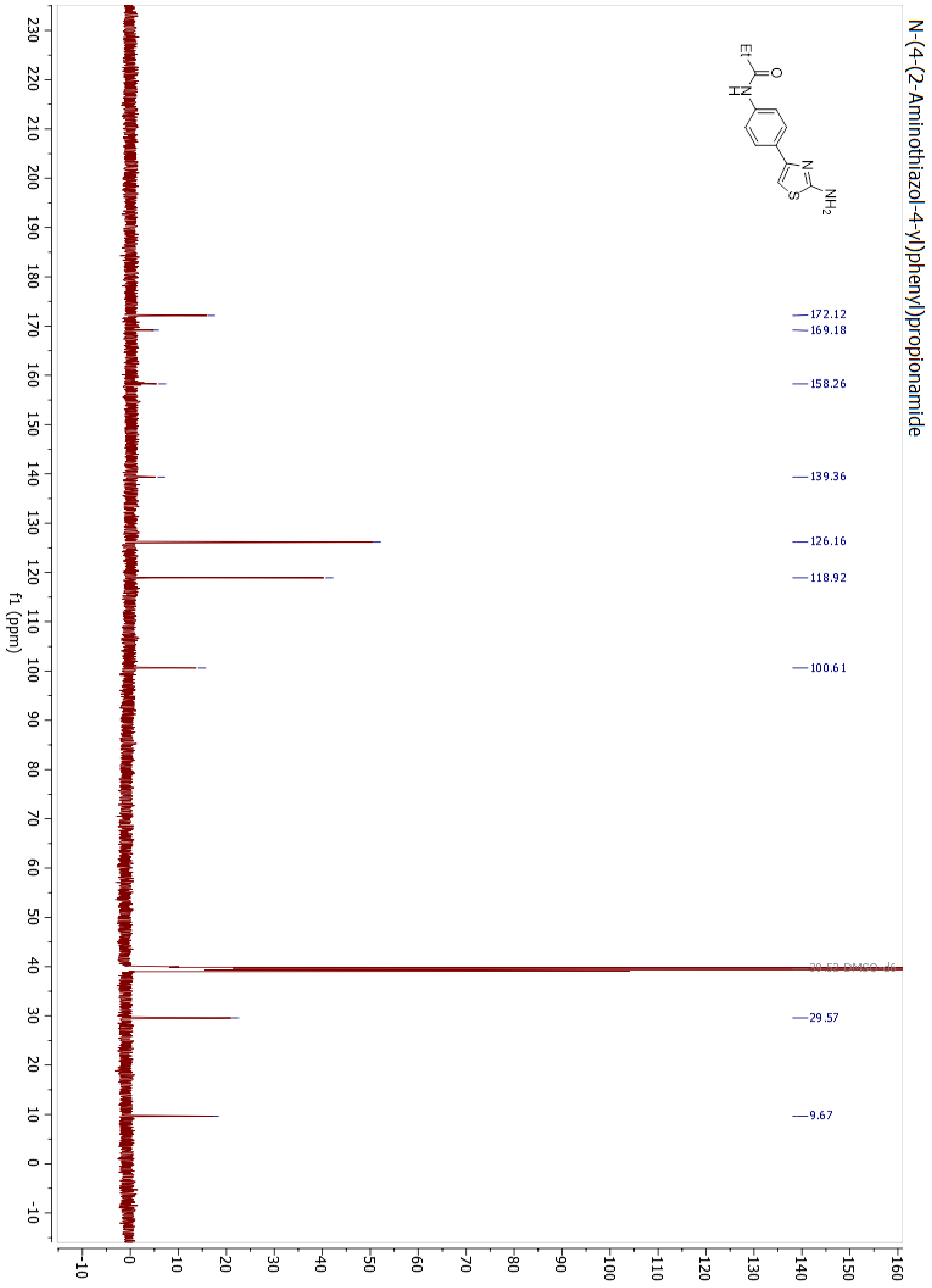

Figure S134. ^{13}C NMR spectrum of **3t'** in d_6-DMSO (151 MHz)

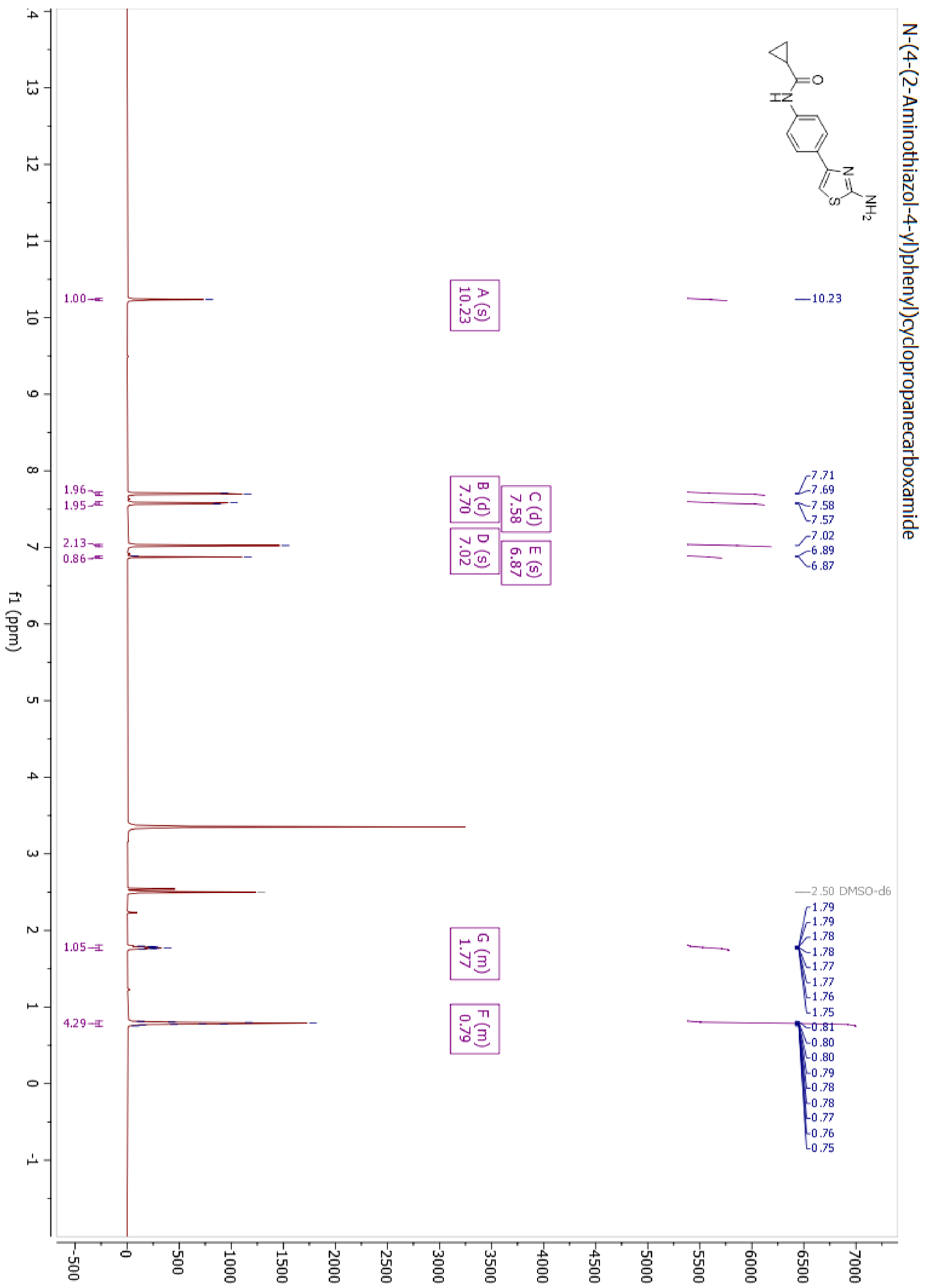

Figure S135. ^{1}H NMR spectrum of **3u’** in d_6-DMSO (600 MHz)

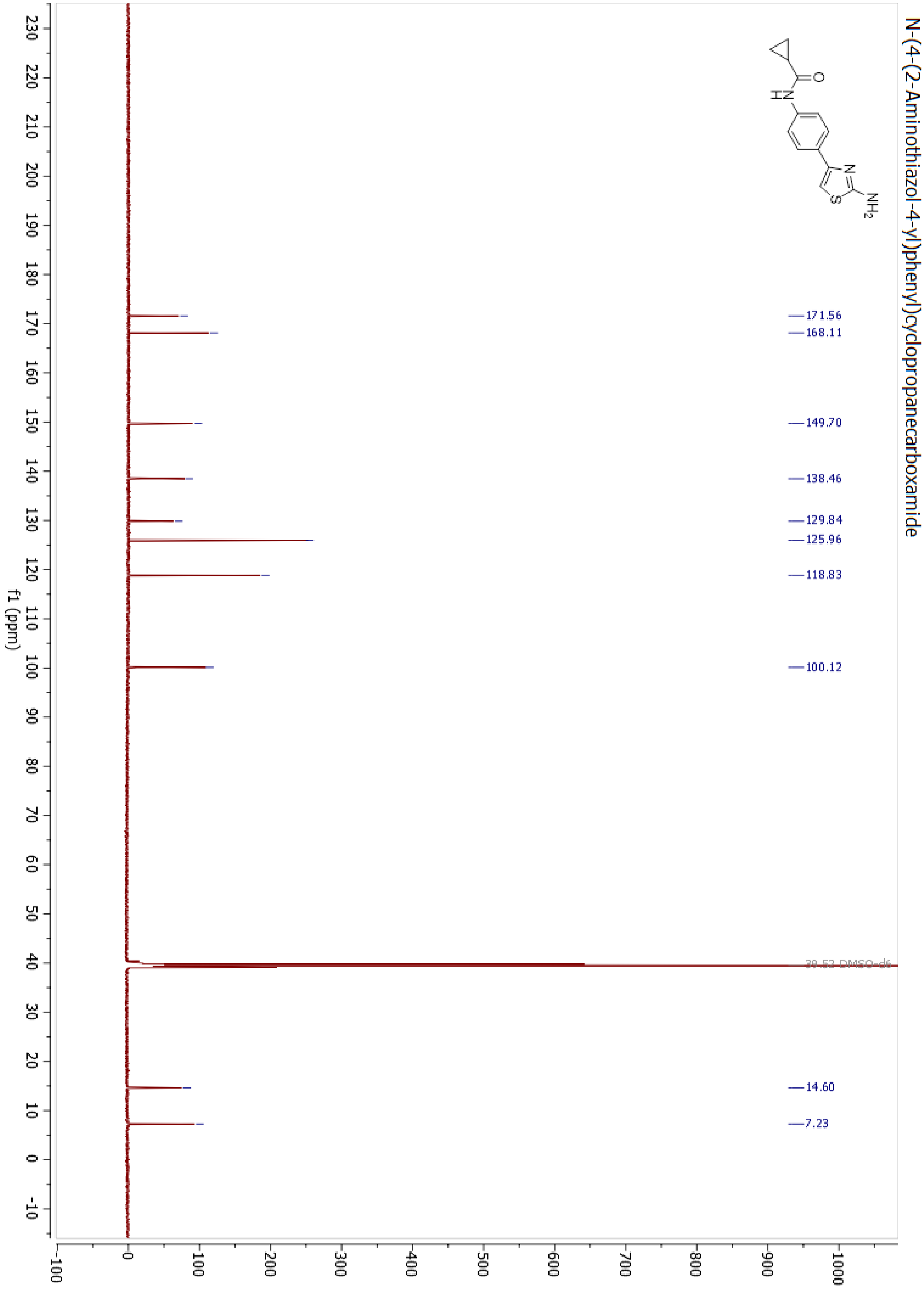

Figure S136. ^{13}C NMR spectrum of **3u’** in d_6-DMSO (151 MHz)

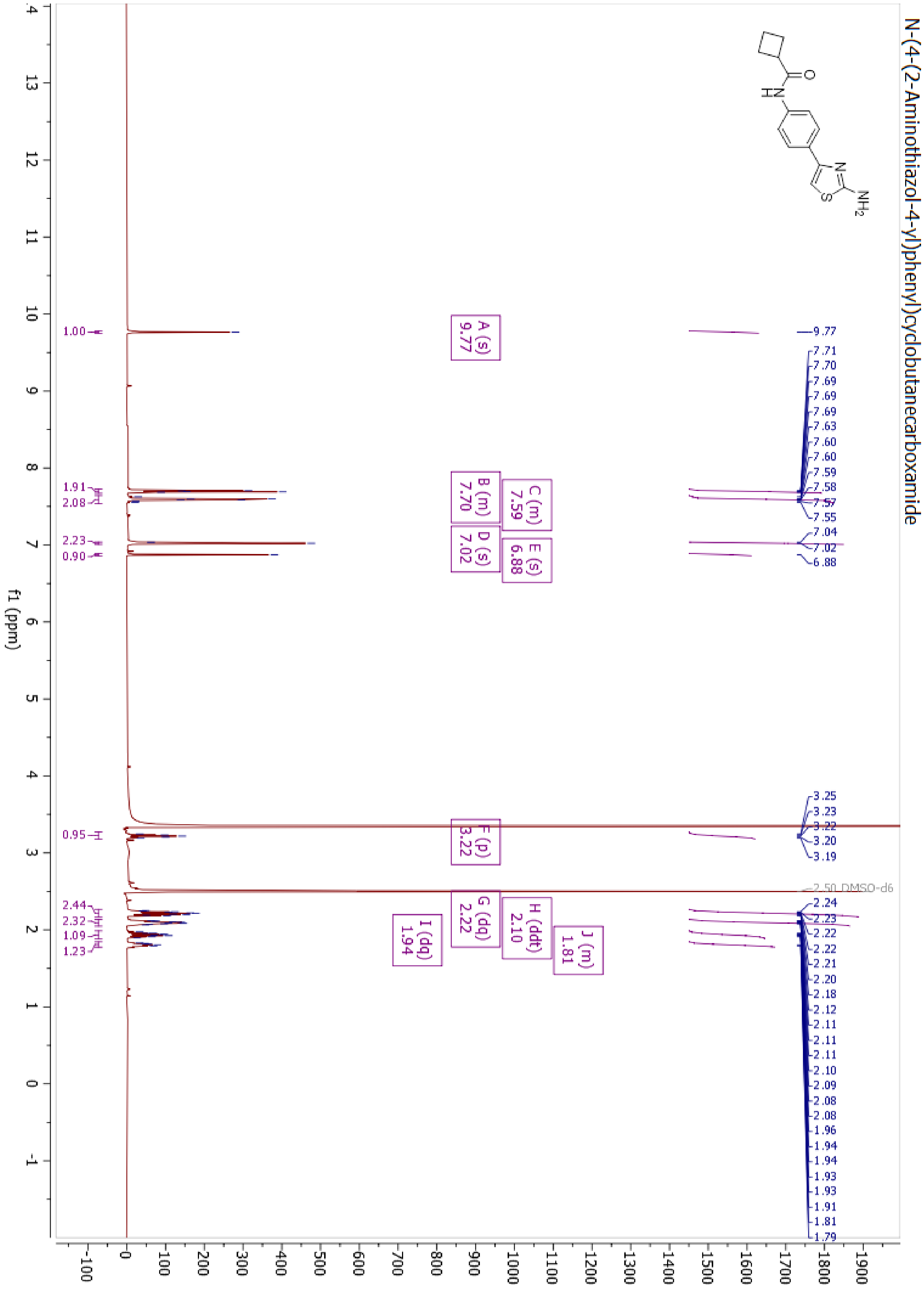

Figure S137. ^{1}H NMR spectrum of **3v’** in d_6-DMSO (600 MHz)

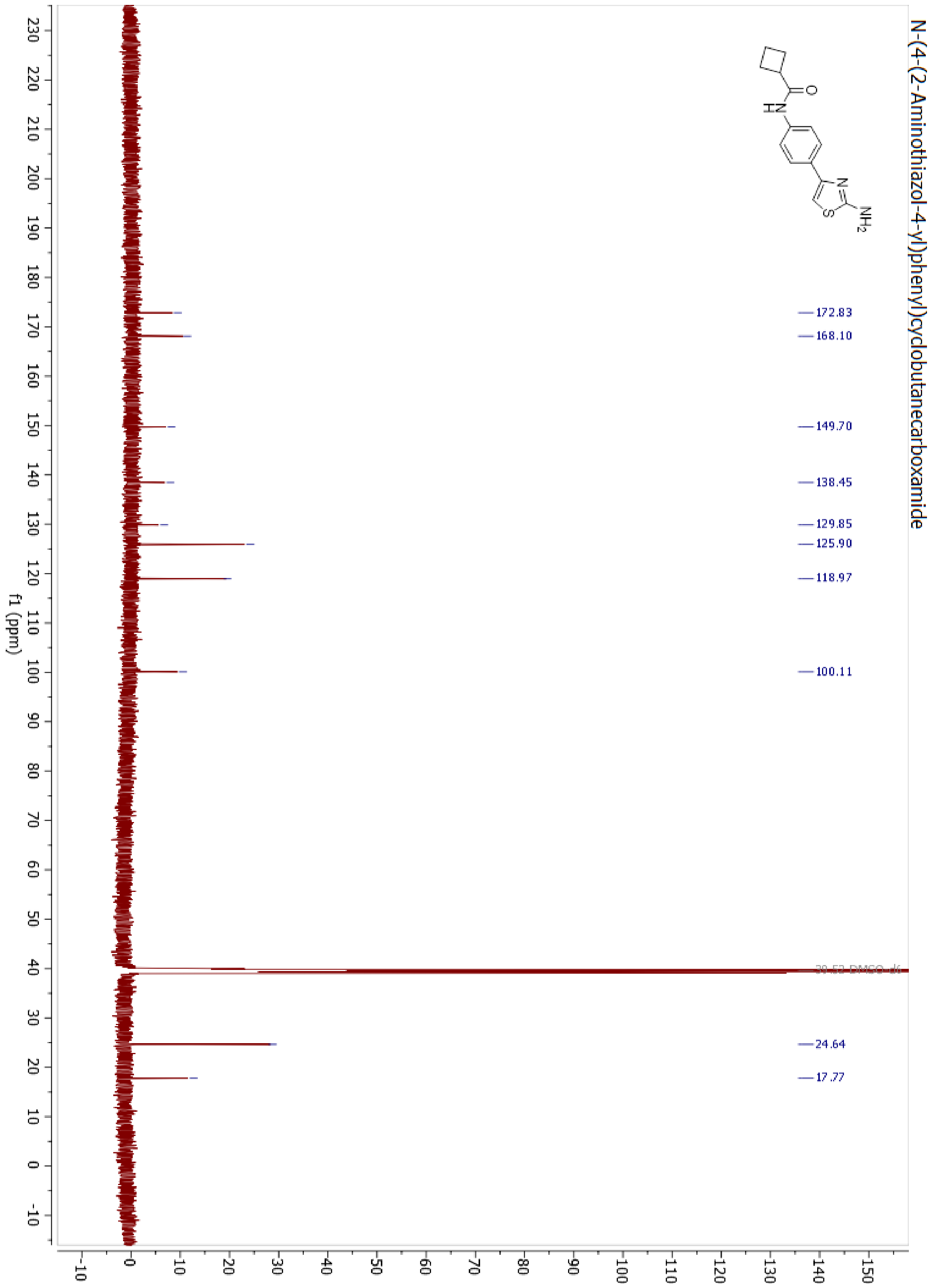

Figure S138. ^{13}C NMR spectrum of **3v’** in d_6-DMSO (151 MHz)

Table S3: Crystallographic Statistics

Data Set<br>(Highest shell in parentheses)	8AXP
a (Å)	43.44
b (Å)	71.51
c (Å)	146.99
α (°)	90.0
β (°)	90.0
γ (°)	90.0
Space Group	$P\ 2_1\ 2_1\ 2_1$
Wavelength (Å)	0.92819
Resolution Limit (Å)	37.40 - 1.83 (1.86 - 1.83)
Number of Unique Obs.	38126 (2032)
Completeness (%)	92.2 (100)
Multiplicity	6.3 (6.8)
Rmerge (I) %	0.072 (2.042)
Rpim(I) %	0.032 (0.841)
$CC_{1/2}$	0.998 (0.669)
I/σI	16.5 (1.1)
Refinement	
Resolution Range (Å)	37.40 - 1.83
Rcryst	0.3345
Rfree	0.3641
Number of protein atoms	2964
Number of ligand atoms	68

Number of solvent atoms	292
Mean B	38.49
Rmsd bond lengths (Å)	0.006
Rmsd bond angles (°)	0.882

Experimental

All crystals were grown by the sitting drop vapour diffusion method in 96-well MRC-2 crystallisation plates. 60 µL of the reservoir solution (100 mM Bis-Tris Propane pH 5.95, 200 mM Li_2SO_4 and 30% PEG 3350) was pipetted into the wells. The drop volume was 0.5 µL, composed of 200 nL of 13.7 mg/mL gonoccocal Pth, 200 nL reservoir solution and 100 nL seed crystals. The plate was sealed quickly and kept in an incubator at 18ºC and the single crystals appeared in 2-7 days. Soaking was performed at the XChem facility at Diamond Light Source where 80nl of the ligand (500mM in DMSO) were added to each drop and left to soak for 3hrs. After cutting and opening the sealing tape, the crystals were immediately fished and placed in liquid nitrogen. Data from all crystals were collected on beamline I04-1 at Diamond Light Source (Oxfordshire, UK) and processed in the xia2 pipeline. The structure was solved by molecular replacement using 4QT4 as the template and refined with Refmac5 in CCP4 and Phenix.refine in Phenix. Manual model building was performed with COOT.
